# Polarity on Demand: Nucleophilic, Electrophilic, and Ambiphilic Reactivity at a 9,10‐Dihydro‐9,10‐Disilaanthracene Platform

**DOI:** 10.1002/chem.71100

**Published:** 2026-05-18

**Authors:** Moritz Schmidt, Alexander Virovets, Eugenia Peresypkina, Hans‐Wolfram Lerner, Matthias Wagner

**Affiliations:** ^1^ Institut für Anorganische und Analytische Chemie Goethe‐Universität Frankfurt am Main Frankfurt am Main Germany

**Keywords:** ambiphiles, disilaanthracenes, Lewis acids, silanides, silicon

## Abstract

9,10‐Dihydro‐9,10‐diheteroanthracenes constitute rigid molecular entities that enable cooperative reactivity between two proximal heteroatoms. While diboraanthracenes act as ditopic Lewis acids and diphosphaanthracenes as ditopic Lewis bases, ambiphilic behavior has so far largely relied on the incorporation of two different heteroelements. Herein, we demonstrate ambiphilicity within a homo‐heteroelement framework based on 9,10‐dihydro‐9,10‐disilaanthracene (DSA). Efficient cyclocondensation of 1,2‐disilyl‐4,5‐dimethylbenzene, promoted by substoichiometric amounts of MeLi, affords DSA **1^2H^** featuring two SiH_2_ groups. Compound **1^2H^** exhibits electrophilic reactivity toward magnesium anthracene, affording a double ‘butterfly’‐type framework **13** reminiscent of the anthracene photodimer. Selective reductive Si–H bond cleavage using 2 equiv. KC_8_ or excess elemental K enables controlled, stepwise access to the monosilanide [**1^H^**]^−^ or the disilanide [**1**]^2−^, respectively. The disilanide [**1**]^2−^, which was structurally characterized by SCXRD, behaves as a ditopic Lewis base toward 9,10‐dihydro‐9,10‐diboraanthracene, forming a cyclic, **13**‐type double adduct K_2_[**10**]. Notably, [**1^H^**]^−^ displays ambiphilic reactivity toward *p*TolC≡C*p*Tol to furnish a tricyclic product containing a deprotonated 1,2‐ethylenediyl bridge between the Si centers. These findings establish that controlled reduction within a homo‐heteroelement platform enables polarity switching, thereby providing a versatile strategy for the design of cooperative main‐group reactivity.

## Graphical abstract

A 9,10‐dihydro‐9,10‐disilaanthracene platform was efficiently synthesized and features intrinsically electrophilic Si sites. Upon stepwise reductive Si─H bond cleavage, these Si sites are converted into nucleophilic centers. This controlled polarity switching also enables ambiphilic reactivity within a homo‐heteroelement framework.

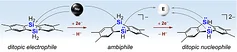

## 1 Introduction

**FIGURE 1:**
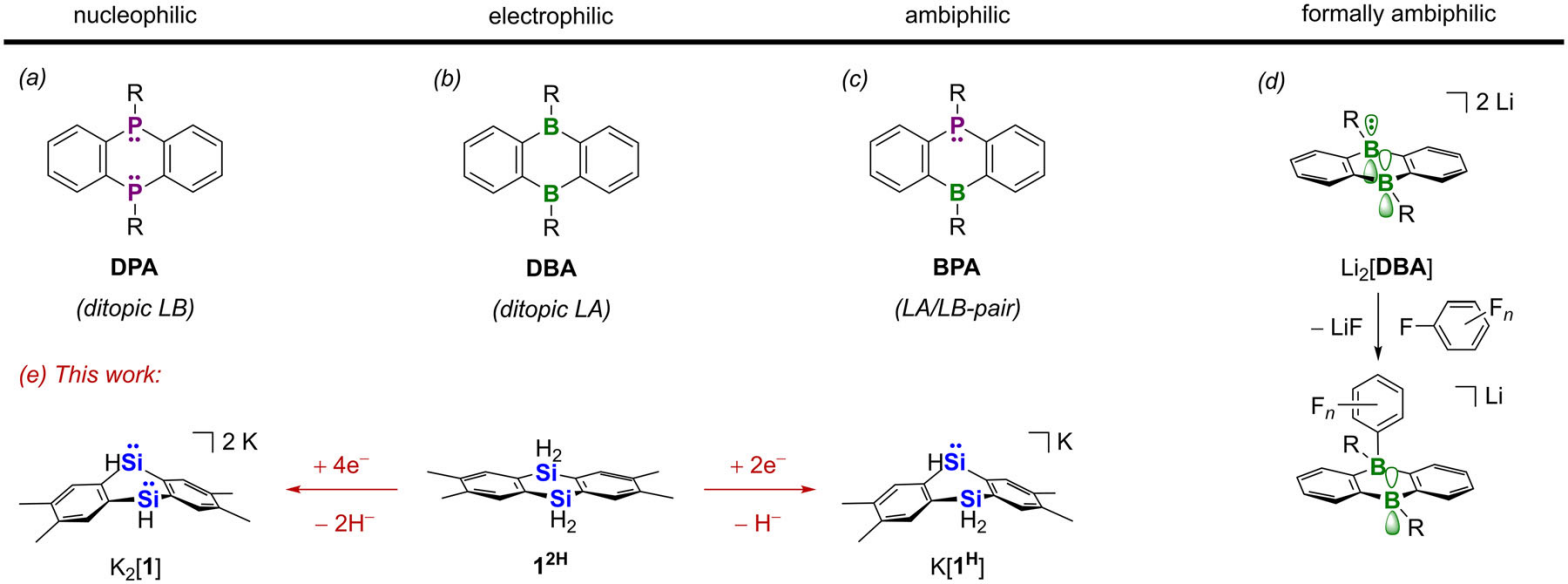
Conceptual overview of Lewis‐basic, ‐acidic, and ambiphilic 9,10‐dihydro‐9,10‐diheteroanthracenes. (a) DPA, (b) DBA, and (c) BPA as representative ditopic Lewis bases (LB), Lewis acids (LA), and ambiphiles, respectively. (d) B‐centered nucleophilic activation of fluoroarenes by [DBA]^2−^. (e) This work: DSA **1^2H^** and its reduced congeners [**1**]^2−^ and [**1^H^**]^−^ as a unified platform for electrophilic, nucleophilic, and ambiphilic reactivity. *Note*: For the series **1^2H^**, [**1^H^**]^−^, and [**1**]^2−^, we retain the common identifier “**1**” to highlight their close relationship; the superscripts indicate that the neutral and monoanionic species possess two and one additional Si‐bound H atom(s), respectively, relative to the disilanide.

9,10‐Dihydro‐9,10‐diheteroanthracenes feature two heteroatoms in close proximity within a rigid molecular framework, with the bridging *o*‐phenylene rings facilitating electronic communication. The resulting cooperative effects give rise to a broad reactivity spectrum. For instance, the corresponding dihydrodiphosphaanthracenes (DPAs; Figure 1a) behave as ditopic Lewis bases, with examples of both P‐alkylation and P‐oxidation documented [1, 2, 3, 4]. The complementary dihydrodiboraanthracenes (DBAs; Figure 1b), by contrast, exhibit the opposite electronic profile and serve as ditopic Lewis acids for various catalytic applications [5, 6, 7, 8]. Complementing the symmetric DPA and DBA systems, ambiphilic B,P‐hybrid congeners (BPAs; Figure 1c) have emerged as a promising extension: [9, 10, 11, 12] although reports on their cooperative reactivity toward more complex substrates are scarce, a pronounced propensity of both heteroatom centers to become tetracoordinate and form cyclic phosphonium borates has already been established [13, 14, 15]. Notably, the exocyclic P‐substituents serve as a key lever to modulate the B center's acceptor properties.

Beyond these hybrid systems, it remains to be explored whether such ambiphilic properties necessitate two disparate heteroatoms or can instead be imprinted onto a ‘homo‐heteroatom’ framework through judicious molecular design. A compelling precedent is provided by the doubly reduced diboraanthracenes, [DBA]^2−^: these highly reactive species not only activate H_2_ via oxidative addition across their two boron sites [16, 17, 18, 19], but also react with fluoroarenes as B‐centered nucleophiles to afford products containing one arylated, tetracoordinate and one Lewis‐acidic, tricoordinate B atom (Figure 1d). In this capacity, [DBA]^2−^ can be viewed as a formal ‘boron‐only’ Lewis pair, which can be rationalized by a resonance contributor featuring one B atom with a lone pair of electrons and the other with a vacant B(p_z_) orbital [20, 21]. Expanding upon the ‘homo‐heteroatom’ approach by exploiting the diagonal relationship in the periodic table, we investigate herein whether the parent 9,10‐dihydro‐9,10‐disilaanthracene (DSA) **1^2H^** (Figure 1e) can serve as a shared molecular platform for (i) ditopic Lewis bases, (ii) ditopic Lewis acids, and (iii) ambiphilic Lewis acid‐base combinations. In formal terms, unmasking Lewis‐basic character would require twofold deprotonation of **1^2H^** to generate lone pairs at both Si centers to afford [**1**]^2−^, whereas a ditopic Lewis acid might be envisaged via the abstraction of two H^−^ ions. However, the resulting disilylium ion would likely represent an extremely unstable and thus experimentally elusive species. At this juncture, a key advantage of silicon as a third‐row p‐block element comes into play: in contrast to B atoms, Si atoms readily expand their coordination number beyond four to reach penta‐ or hexacoordinate states [22, 23]. Consequently, already the saturated DSA **1^2H^** should exhibit a certain Lewis‐acidic or electrophilic behavior toward suitable nucleophiles, obviating the need for delicate silylium intermediates.

**SCHEME 1:**
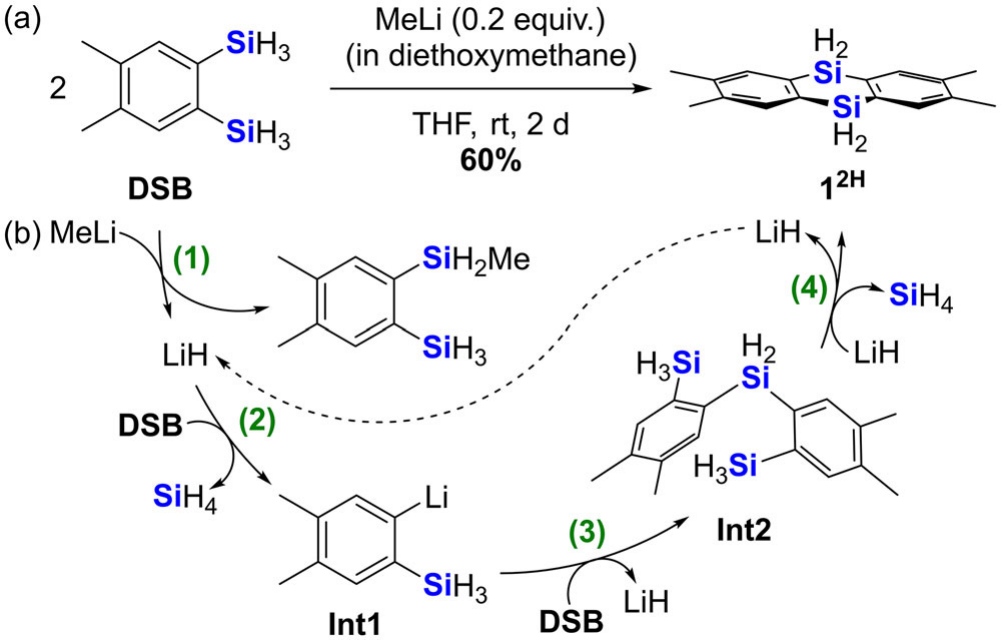
(a) MeLi‐induced cyclocondensation of **DSB** to afford **1^2H^**. (b) Proposed H^−^‐mediated inter‐ and intramolecular condensation pathway; individual steps (1)–(4) are discussed in the main text.

Following this conceptual approach, we herein report an efficient cyclocondensation strategy for the assembly of the parent DSA **1^2H^** (cf. Scheme 1). Selective reductive Si─H bond cleavage converts **1^2H^** into either the disilanide [**1**]^2−^ or the monosilanide [**1^H^**]^−^. The resulting [**1**]^2−^/**1^2H^**/[**1^H^**]^−^ series encompasses all reactivity modes outlined in (i)–(iii): (i) Double adduct formation between [**1**]^2−^ and DBA furnishes a B_2_Si_2_‐doped isostructural analogue of the anthracene photodimer (cf. Scheme 4a) [24, 25, 26]. (ii) Conversely, **1^2H^** plays the role of a dielectrophile toward the polarity‐inverted (relative to DBA) magnesium anthracene, forming the corresponding Si_2_‐doped species (cf. Scheme 4b). (iii) Finally, [**1^H^**]^−^ reacts with alkynes to afford tricyclic 9,10‐dihydro‐9,10‐ethanoanthracenes with bridgehead Si atoms (cf. Scheme 5). Three mechanistic scenarios, at least two of them consistent with a formally ambiphilic Lewis acid‐base character of [**1^H^**]^−^, are discussed.

## 2 Results and Discussion

DSAs emerge as ideal candidates for achieving our objectives, owing to (i) the limited *n*‐π overlap inherent to DSA‐embedded silanide centers, thereby endowing them with Lewis basicity [9, 10], and (ii) the propensity of Si atoms to adopt hypercoordinated states, which facilitates their role as Lewis acids/electrophiles [27, 28]. To minimize steric hindrance that could compromise targeted DSA‐substrate interactions [17] and to avoid complications arising from *cis*/*trans* isomers in the same sample [29], we selected a DSA derivative incorporating sterically undemanding SiH_2_ moieties (cf. **1^2H^**). Beyond these structural considerations, the Si─H functionalities provide a versatile synthetic handle: hydrosilanes are known to undergo reductive cleavage of the Si─H bond (by virtue of its low‐lying σ* orbital) [30] and nucleophilic substitution reactions (with H^−^ acting as a leaving group) [31, 32]. Both features are instrumental for the intended downstream chemistry. Established routes to DSAs primarily rely on salt metathesis reactions between aryllithium or Grignard reagents and (halo)silanes [31, 33, 34, 35, 36, 37]. In 2016, Nishihara, Yamanoi et al. disclosed a Pd‐catalyzed coupling reaction of *o*‐bis(dimethylsilyl)arenes with *o*‐diiodoarenes, furnishing (unsymmetrically substituted) DSAs in moderate yields [38].

### 2.1 Synthesis of DSA **1^2H^** and Its Conversion to Mono‐ and Disilanides

Our target DSA **1^2H^**, featuring exclusively H substituents at the Si sites [39, 40], was synthesized via a distinct cyclocondensation of 1,2‐disilyl‐4,5‐dimethylbenzene (**DSB**) [41, 42, 43], induced by substoichiometric amounts of MeLi (60% yield; Scheme 1a).

Regarding the reaction mechanism (Scheme 1b), we propose that (1) a nucleophilic attack by the Me^−^ anion on an SiH_3_ substituent of **DSB** results in the in situ formation of reactive LiH [44, 45], which (2) adds to another molecule of **DSB** to generate SiH_4_ and the aryllithium intermediate **Int1**. (3) Subsequent reaction of the **Int1** nucleophile with another molecule of **DSB** effects the first, intermolecular condensation, affording intermediate **Int2** with concomitant regeneration of LiH. (4) The sequence concludes with an intramolecular condensation, again triggered by H^−^ coordination to an SiH_3_ group and proceeding analogously to the first condensation step [46]. For simplicity, this discussion considers the extreme case of complete dissociation of LiH and aryllithium within the individual steps. However, substituent exchange at the Si atom may also proceed through a concerted process between penta‐ and tetracoordinate species [arylSi(R)H_3_]^−^ and arylSiH_3_ (R = Me, H).

The overall mechanistic scenario is supported by the following experimental evidence and literature precedent: (1) derivatives of starting material **DSB** bearing partially methylated silyl groups were isolated as byproducts (Table S1). (2) **DSB** readily underwent cyclocondensation to give **1^2H^** upon addition of commercial KH [47, 48] or KC_8_, which is known to generate KH in situ upon reaction with hydrosilanes [49]. Formation of SiH_4_ was revealed by NMR monitoring of the reaction mixture in [D_8_]THF (Figures S5–S7,S14). (3, 4) Side products resulting from overcondensation, namely partially and fully ring‐closed disilatrypticene derivatives, were isolated in trace amounts and characterized by NMR spectroscopy and single‐crystal X‐ray diffraction (SCXRD; Scheme S3, and Figures S104,S105).

Moreover, our observations align with previous reports and mechanistic proposals for the redistribution of the model system PhSiH_3_ induced by *n*BuLi, PhLi, or Na[AlH_4_], furnishing mixtures of SiH_4_, Ph_2_SiH_2_, and small amounts of Ph_3_SiH (< 10%) [50, 51]. Notably, **Int1** does not appear to form directly via the initial attack of MeLi on the starting material **DSB**, as this transformation would liberate MeSiH_3_, which was not observed in any of our in situ NMR monitoring experiments (in contrast to the similarly volatile SiH_4_).

**SCHEME 2:**
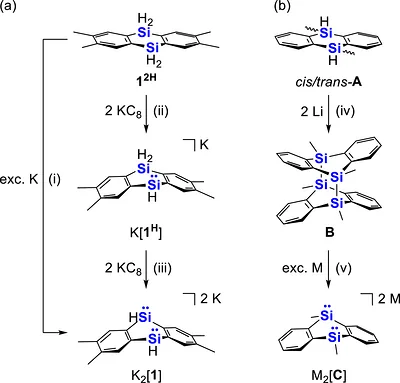
(a) Direct and stepwise reductive Si─H bond cleavage of **1^2H^**, affording K[**1^H^**] and K_2_[**1**]. (b) Dehydrocoupling of *cis*/*trans*‐**A** to afford the cyclic dimer **B**, followed by reductive Si─Si bond cleavage to give M_2_[**C**]. Reagents and conditions: (i) K metal (20 equiv.), THF, rt, 14 h; (ii) KC_8_ (2 equiv.), THF, rt, 2 h; (iii) KC_8_ (2 equiv.), THF, rt, 14 h; (iv) Li metal (2 equiv.), THF/TMEDA, rt, 48 h; (v) excess M metal (M = Li, K), THF, rt.

Reduction of DSA **1^2H^** with a fivefold excess of K metal in THF furnishes the DSA‐based disilanide salt K_2_[**1**] (Scheme 2a). According to NMR monitoring in [D_8_]THF, the conversion is essentially quantitative. For this reason, the deep red solutions of K_2_[**1**] were directly used for subsequent studies after filtration without further workup. The corresponding monosilanide salt K[**1^H^**] is readily accessible through reduction of **1^2H^** with 2 equiv. of KC_8_ in THF. Again, the orange reaction mixture can be used directly for follow‐up derivatizations after filtration. DSA‐based monosilanides such as [**1^H^**]^−^ are unprecedented. The only synthesis of a corresponding disilanide published to date started from *cis*/*trans*‐9,10‐dihydro‐9,10‐dimethyl‐9,10‐disilaanthracene *cis*/*trans*‐**A**, which was subjected to dehydrocoupling with Li metal in THF to give the cyclic dimer **B** (Scheme 2b). In a second step, **B** was converted into the dianion [**C**]^2−^ by cleavage of its Si─Si bonds using excess Li or K [30]. The ^29^Si NMR spectrum of neutral DSA **1^2H^** exhibits a triplet resonance at −49.8 ppm (^1^ *J*_SiH_ = 199 Hz); the corresponding resonance of K_2_[**1**] appears as a doublet at *δ*(^29^Si) = −48.7 (^1^ *J*_SiH_ = 102 Hz). Remarkably, the chemical shift value remains essentially unchanged upon dianion formation, which contrasts with the pronounced downfield shifts typically observed upon phenylsilanide formation in related model systems [for comparison: Ph_3_SiH [52] vs. Ph_3_SiK [53]: −17.9 vs. −7.1 ppm; Ph_2_SiH_2_ [54] vs. K[Ph_2_Si(H)NiL*_n_*] [55]: −33.8 (^1^ *J*_SiH_ = 200 Hz) vs. 21.2 ppm (^1^ *J*_SiH_ = 143 Hz)]. The significantly smaller ^1^ *J*_SiH_ value in K_2_[**1**] reflects an increased p character of the remaining Si─H bond and thus conforms with Bent's rule [56, 57]. In line with these observations, the monosilanide salt K[**1^H^**] gives rise to two signals at *δ*(^29^Si) = −49.8 (t, ^1^ *J*_SiH_ = 181 Hz; SiH_2_) and −40.8 (d, ^1^ *J*_SiH_ = 103 Hz; SiH). The resonances of the Si‐bonded H atoms of **1^2H^** and K_2_[**1**] are detected at *δ*(^1^H) = 4.85 and 4.35, respectively. Two distinct doublets are observed for the SiH_2_ protons of K[**1^H^**], confirming a pyramidalized, configurationally stable silanide center on the NMR timescale, which renders the two SiH_2_ protons diastereotopic.

**FIGURE 2:**
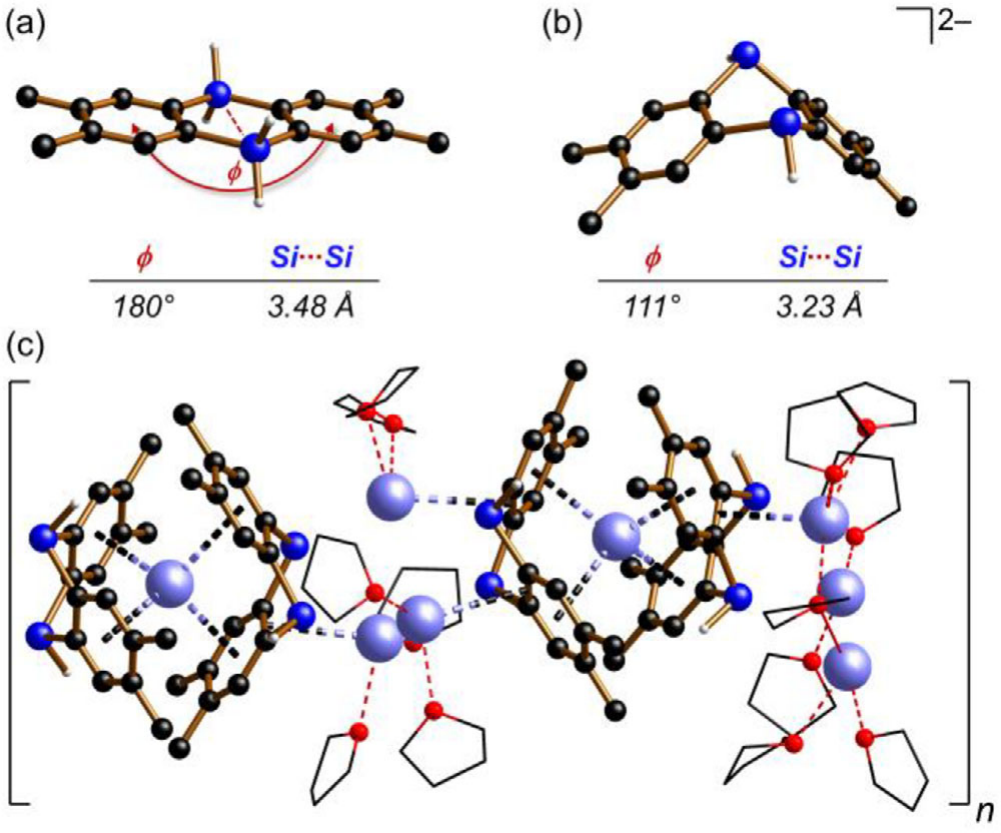
Solid‐state structures of (a) **1^2H^** and (b) one of the eight crystallographically unique dianions [**1**]^2−^. Complex cations, solvent molecules, and H atoms except those bound to Si are omitted for clarity. (c) Section of one of the two crystallographically independent chains in the crystal structure of K_2_[**1**]; H atoms except those bound to Si are omitted for clarity. H: light gray; C: black; Si: blue; K: violet‐blue.

In the solid state, DSA **1^2H^** adopts a planar, *C*_i_‐symmetric structure with average Si─C bond lengths of 1.866[1] Å (Figure 2a) [58, 59]. The crystal structure of its dianion salt K_2_[**1**] is notably more complex, comprising 8 crystallographically unique [**1**]^2−^ anions, 16 K^+^ cations, and 25 THF ligands, all located in general positions of the $P\bar{1}$ space group. As the key geometric parameters of all [**1**]^2−^ moieties are similar, the discussion below is based on average values: each [**1**]^2−^ ion is substantially folded along the intramolecular Si···Si vector, with a dihedral angle between its two phenylene rings of *ϕ* = 111[3]° (Figure 2b) [58]. This folding results in a transannular Si···Si contraction (from 3.481(1) Å in **1^2H^** to 3.234[19] Å in [**1**]^2−^) and a marked compression of the endocyclic C─Si─C angles from 112.6(1)° to 97.1[8]° [58]. The latter indicates a high s character of the electron lone pairs at the silanide centers; accordingly, the average Si─C bond lengths are elongated from 1.866[1] to 1.916[7] Å [58]. The concave face of each dianion interlocks with that of a neighboring dianion, forming a cavity that accommodates one K^+^ ion devoid of THF ligands and interacting solely with the surrounding phenylene rings (Figures 2c and S106). The convex face of each dianion is oriented toward that of a second neighboring dianion so that their two Si···Si vectors subtend an angle of 65[5]° [58]; three THF‐coordinated K^+^ ions are located between the resulting set of four negative charges, thereby forming a coordination polymer (Figures 2c and S107) [60]. According to SCXRD, the central heterocycle of the folded [**1**]^2−^ anion adopts a boat conformation (Figure 2b). Both Si‐bonded H atoms, which were located in the difference electron‐density map and freely refined, reside at equatorial positions, indicating that the electron lone pairs are located at both axial positions and thus point in the same direction. Does this *cis* configuration persist in THF solution? Notably, the isoelectronic, P‐analogue DPA can exist in both its *cis* and *trans* (refcode: NAGLOW) configurations [1, 2], suggesting that a similar configurational diversity cannot be ruled out *a priori* for [**1**]^2−^. However, the NMR spectra of K_2_[**1**] exhibit only a single set of resonances and are therefore inconsistent with the presence of a static mixture of *cis*/*trans* isomers. Consequently, either the *cis* isomer is exclusively present, or a rapid inversion of the silanide centers leads to a higher time‐averaged symmetry. The latter assumption is implausible because: (i) the presence of diastereotopic protons in the monoanion salt K[**1^H^**] has already demonstrated that the chiral silanide ion is configurationally stable – at least on the NMR timescale; (ii) the established inversion barrier for silanides of 26±6 kcal mol^−1^ would, at most, allow for a slow inversion process at room temperature [61, 62]; and (iii) the computed energy for the *trans* configuration of the related Li_2_[(*o*‐C_6_H_4_─SiMe)_2_] is 26 kcal mol^−1^ higher than that of the most stable contact‐ion pair containing a *cis*‐configured dianion [60].

**SCHEME 3:**
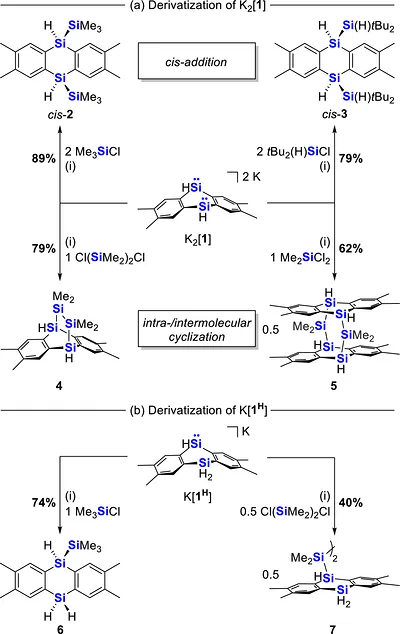
(a) Derivatization of K_2_[**1**]. Silylation with Me_3_SiCl or *t*Bu_2_(H)SiCl affords *cis*‐**2** and *cis*‐**3**, respectively (top), whereas the reaction with Cl(SiMe_2_)_2_Cl or Me_2_SiCl_2_ furnishes **4** and **5** (bottom). (b) Derivatization of K[**1^H^**]. Silylation with Me_3_SiCl affords **6**, while reaction with Cl(SiMe_2_)_2_Cl gives **7**. Conditions: (i) THF, −78 °C to rt, 14 h.

The conclusion that reduction of **1^2H^** with K metal selectively furnishes [**1**]^2−^ was further corroborated by a series of derivatization reactions: (a) Treatment of K_2_[**1**] with Me_3_SiCl and even with the sterically demanding *t*Bu_2_(H)SiCl afforded the bis‐silylation products *cis*‐**2** and *cis*‐**3** in high yields of 89% and 79%, respectively (Scheme 3a, top). (b) Reaction of K_2_[**1**] with Cl(SiMe_2_)_2_Cl furnished the tricyclic species **4** (79%), whereas treatment with Me_2_SiCl_2_ afforded the macrocyclic dimer **5** (62%; Scheme 3a, bottom). The monosilanide nature of K[**1^H^**] was likewise confirmed by derivatization with Me_3_SiCl and Cl(SiMe_2_)_2_Cl (Scheme 3b). In the resulting products **6** and **7**, only one Si atom of the respective DSA unit became silylated, whereas the second remained as an SiH_2_ moiety (more details are provided in the Supporting Information; see Figures S112–S117 for SCXRD results on *cis*‐**2**–**7**) [63].

### 2.2 Disilanide [**1**]^2−^ as a Ditopic Nucleophile and DSA **1^2H^** as Its Complementary Bis‐Electrophile

**SCHEME 4:**
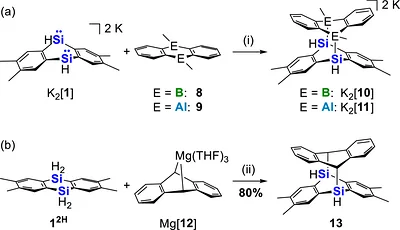
K_2_[**1**] as a ditopic nucleophile and **1^2H^** as a ditopic electrophile. (a) Reaction of K_2_[**1**] with **8** or **9** affords the cyclic adducts K_2_[**10**] or K_2_[**11**], respectively. (b) Reaction of **1^2H^** with Mg[**12**] affords **13**. Reagents and conditions: (i) [D_8_]THF, rt; (ii) Mg[**12**] (4 equiv.), THF, 80 °C, 2 h.

To evaluate the utility of [**1**]^2−^ as a ditopic Lewis base, K_2_[**1**] was treated with its Lewis acid counterparts [64, 65], DBA **8** and its Al congener **9**, hereafter referred to as DAA (Scheme 4a). Twofold Lewis acid‐base pairing afforded the cyclic products K_2_[**10**] and K_2_[**11**], which are structurally related to compound **B** and the classic anthracene photodimer [24, 25, 26]. Unlike the salt metathesis between K_2_[**1**] and Cl(SiMe_2_)_2_Cl to afford **4** (Scheme 3a), the syntheses of K_2_[**10**] and K_2_[**11**] lack the thermodynamic driving force of KCl elimination; nonetheless, both conversions were near quantitative according to NMR spectroscopy. The chemoselectivity of these couplings is of particular interest, as we have no indication of single‐electron transfer (SET) between [**1**]^2−^ and DBA **8**, despite the well‐known radical chemistry of DSAs [29, 39, 66, 67, 68] and markedly high electron affinities of DBAs [19, 69]. In a complementary approach, the dinucleophilic magnesium anthracene Mg[**12**] reacts with the DSA **1^2H^** in THF to form the neutral product **13**, thereby further expanding the class of double ‘butterfly’ frameworks (Scheme 4b) [70]. A fourfold excess of the organomagnesium compound Mg[**12**] and heating at 80 °C for 2 h in a sealed ampoule are required to achieve good selectivity, likely reflecting an only moderate electrophilic character of **1^2H^** [71]. Nevertheless, following chromatographic purification, **13** is obtained in high yields of 80%.

NMR‐spectroscopic analysis of K_2_[**10**], K_2_[**11**], and **13** is facilitated by the four C‐appended Me substituents, which simplify the coupling patterns and allow unambiguous distinction between the DSA and the DBA/DAA/DHA halves (DHA: 9,10‐dihydroanthracenediyl). ^1^H NMR integration confirmed a 1:1 stoichiometry for all three products. The bridgehead B atoms of K_2_[**10**] resonate at −17.3 ppm, well within the characteristic range for tetracoordinate B species [72]; the bridgehead C atoms of **13** give rise to a signal at 40.9 ppm. The ^29^Si NMR signals of all three reaction products are downfield‐shifted relative to K_2_[**1**] (−48.7 ppm), appearing at −35.3 ppm (K_2_[**11**]), −11.3 ppm (**13**), and 2.2 ppm (K_2_[**10**]). A clear trend is observed for the ^1^ *J*_SiH_ coupling constants, which increase across the series [**1**]^2−^ (102 Hz) < [**11**]^2−^ (145 Hz) ≈ [**10**]^2−^ (155 Hz) < **13** (203 Hz), consistent with an increasing s character of the Si─H bonds and concomitant redistribution of p character into the Si–bridgehead bonds [56]. Notably, the ^13^C resonances of the C‐*ipso* atoms in the DSA moieties of K_2_[**10**] and K_2_[**11**] (154.5 and 152.0 ppm, respectively) remain close to that of K_2_[**1**] (156.5 ppm) and are still markedly downfield‐shifted compared to **1^2H^** (135.2 ppm). In contrast, **13** shows a *δ*(^13^C) value of 140.1 ppm, effectively returning to the range observed for the saturated starting material **1^2H^**.

**FIGURE 3:**
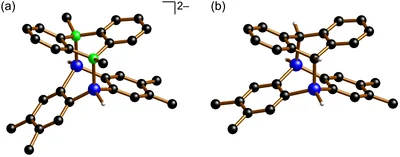
(a) One of the two crystallographically independent [**10**]^2−^ units ([**10**_A_]^2−^) in the crystal structure of [K(18‐c‐6)(THF)_2_][K(18‐c‐6)]_3_[**10**]_2_ and (b) **13**. Complex cations, solvent molecules, and H atoms except for the bridgehead H atoms are omitted for clarity. H: light gray; C: black; Si: blue; B: green.

The molecular structures of the crown‐ether adduct [K(18‐c‐6)(THF)_2_][K(18‐c‐6)]_3_[**10**]_2_ and its neutral congener **13** were confirmed by SCXRD (Figure 3a,b). The crystal lattice of the DBA adduct contains two crystallographically independent [**10**]^2−^ anions: one ([**10**_A_]^2−^) forms contact pairs with two [K(18‐c‐6)]^+^ cations coordinated to the phenylene peripheries of the DBA and DSA fragments, whereas the other ([**10**_B_]^2−^) bears only a single DBA‐coordinated [K(18‐c‐6)]^+^ unit, with a solvent‐separated [K(18‐c‐6)(THF)_2_]^+^ cation completing the charge balance (Figure S109). Contact‐pair formation affects the dihedral angles between the phenylene rings of the DBA/DSA fragments, with values of *ϕ* = 154.6(2)/136.8(3)° and 141.7(2)/122.7(5)° for [**10**_A_]^2−^ and [**10**_B_]^2−^, respectively. In both cases, DSA folding exceeds DBA folding by almost 20°, although the distortion remains less pronounced than in K_2_[**1**] (111[3]°) [58], consistent with a higher degree of p character in the Si─B‐adduct bond compared to the Si lone pair. The Si─B bond lengths in [**10**]^2−^ range from 2.090(9) to 2.106(11) Å and are thus elongated relative to reference adducts of the type X_3_Si─BX_2_·Do (1.977(6)–2.043(4) Å; X = Cl, I; Do = neutral or anionic donor) [73, 74]. The average sum of C─B─C angles amounts to 339[2]° [58], approximately 20° smaller than the 360° diagnostic for planar B atoms, yet still about 10° larger than the 328.5° expected for tetrahedral B centers.

Neutral **13** exhibits the least folded DSA unit (*ϕ* = 141.9(2)°) relative to [**1**]^2−^ and [**10**]^2−^. Although the DHA fragment in **13** (*ϕ* = 146.7(1)°) shows distortion similar to the DBA fragment in [**10**]^2−^, the corresponding C─C─C/H angle sums of 328° now match the value expected for tetrahedral bridgehead atoms. As expected, the Si─C(bridgehead) bonds of 1.942(2) and 1.943(2) Å are shorter than the Si─B bonds in [**10**]^2−^. A summary of key structural and spectroscopic parameters of **1^2H^**, [**1^H^**]^−^, [**1**]^2−^, [**10**]^2−^, and **13** is provided in Table 1.

**TABLE 1:** ^29^Si and ^13^C NMR chemical shift values, ^1^ *J*_SiH_ coupling constants, dihedral angles *ϕ*, and Si···Si interatomic distances for **1^2H^**, [**1^H^**]^−^, [**1**]^2−^, [**10**]^2−^, and **13**. Where two values are given, they correspond to the minimum and maximum observed values.

	*δ*(^29^Si) (ppm)	^1^ *J*_SiH_ (Hz)	*δ*(^13^C)^a^ (ppm)	*ϕ* ^b^ (°)	Si···Si^c^ (Å)
**1^2H^**	−49.8	198.7	135.2	180	3.481(1)
[**1^H^**]^−^	H_2_Si: −49.8 HSi: −40.8	181.4 102.7	130.0 167.8	n.a.	n.a.
[**1**]^2−^	−48.7	102.2	156.5	107.0(1) 115.5(1)	3.203(2) 3.262(2)
[**10**]^2−^	2.2	155.0	154.5	122.7(5) 136.8(3)	3.138(2) 3.167(3)
**13**	−11.3	203.4	140.1	141.9(2)	3.191(1)

^a^ ^13^C NMR shifts of the Si‐bonded aryl‐C atoms (C‐*ipso*).

^b^ Dihedral angle between the DSA phenylene rings.

^c^ Interatomic distance between the transannular DSA Si atoms.

### 2.3 Monosilanide [**1^H^**]^−^ as an Ambiphilic Reagent

**SCHEME 5:**
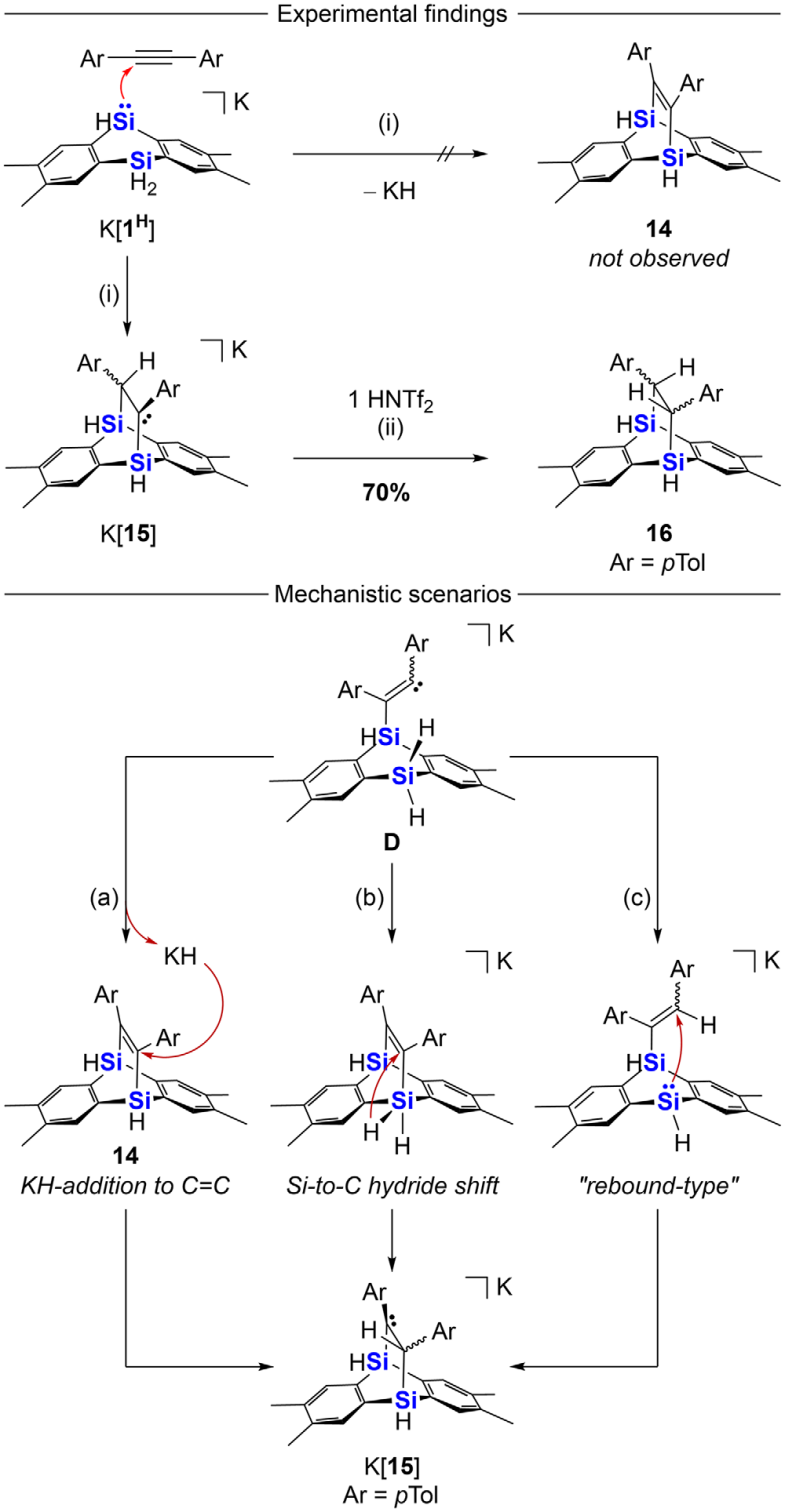
Reaction of K[**1^H^**] with 1,2‐di‐*p*‐tolylethyne and proposed mechanistic scenarios [(a)–(c); Ar = *p*Tol]. Top: Reaction of K[**1^H^**] with *p*TolC≡C*p*Tol affords K[**15**], whereas **14** is not observed; subsequent protonation of K[**15**] with triflimide (HNTf_2_) furnishes **16**. Bottom: Proposed mechanisms for the formation of K[**15**]: (a) KH addition to transiently formed **14**; (b) intramolecular H^−^ migration from pentacoordinate Si to C; and (c) deprotonation of the SiH_2_ unit followed by ‘rebound‐type’ C─Si bond formation. Reagents and conditions: (i) *p*TolC≡C*p*Tol (1 equiv.), THF, rt, 1 h; (ii) HNTf_2_ (1 equiv.), THF, rt, 1 min.

A fundamental challenge in designing molecules featuring concurrent Lewis‐acidic and ‐basic sites is their inherent propensity for head‐to‐tail dimerization, which typically quenches latent reactivity. In the case of monosilanide [**1^H^**]^−^, however, the complementary Si sites are sufficiently attenuated to avoid undesired self‐aggregation. As a result, [**1^H^**]^−^ maintains a monomeric state that is ostensibly dormant yet synthetically accessible—as demonstrated by the reaction of K[**1^H^**] with 1,2‐di‐*p*‐tolylethyne (*p*TolC≡C*p*Tol; Scheme 5). This proof‐of‐principle experiment was inspired by the well‐established addition of Ar_3_P/BR_3_ frustrated Lewis pairs (FLPs) across a C≡C bond via nucleophilic attack of a triarylphosphine at a borane‐activated alkyne [75]. By analogy, the silanide center of [**1^H^**]^−^ could assume the role of the isoelectronic phosphane to generate an Si‐bonded vinyl anion **D** [41, 76, 77]. The resulting intermediate is, in principle, poised for intramolecular cyclization at the SiH_2_ site, with concomitant elimination of KH (cf. the synthesis of **13**), to afford DSA **14** featuring a transannular C═C bridge (Scheme 5, top) [78]. Contrary to *a priori* assumptions, the reaction of K[**1^H^**] with 1 equiv. of *p*TolC≡C*p*Tol in THF at room temperature did not form the neutral **14**, but instead furnished the tricyclic DSA K[**15**], containing a formally deprotonated 1,2‐ethanediyl unit between the Si atoms (Scheme 5, top). Three mechanistic scenarios can be envisaged: (a) the reaction proceeds as intended to produce **14**, but the liberated KH does not remain an innocent byproduct and adds to the transannular C═C bond, affording K[**15**]. (b) In a more plausible intramolecular variant, KH is not released from the SiH_2_ moiety following attack by the vinyl anion, but migrates from the pentacoordinate Si site to a bridging C atom. (c) The vinyl‐anion substituent deprotonates the SiH_2_ unit, and the resulting potassium silanide subsequently attacks the thus‐formed neutral vinyl group in a ‘rebound‐type’ fashion (Scheme 5, bottom) [79, 80].

The primary product K[**15**], which contains a chiral center, was characterized by NMR spectroscopy and SCXRD as a racemic mixture. Following protonation with triflimide (HNTf_2_), separation of the resulting pair of uncharged diastereomers (**16**) was achieved (Scheme 5, top; additionally, the K[**15**]‐analogue bearing *p*‐methoxyphenyl instead of *p*‐tolyl substituents was prepared and successfully quenched with Me_3_SiCl; full details are provided in the Supporting Information).

The ^29^Si NMR spectrum of K[**15**] shows two resonances for the bridgehead Si atoms at *δ* = −44.2 (carbanion‐bonded SiH unit) and −25.0 ppm; the corresponding ^1^ *J*_SiH_ coupling constants are only slightly affected by the presence or absence of the carbanionic substituent, both amounting to approximately 200 Hz. The carbanionic site gives rise to a signal at *δ*(^13^C) = 52.1 ppm. Two sets of ^13^C NMR resonances are observed for the DSA fragment, testifying to the presence of two diastereotopic *o*‐phenylene rings resulting from the saturated, chiral bridging C atom.

**FIGURE 4:**
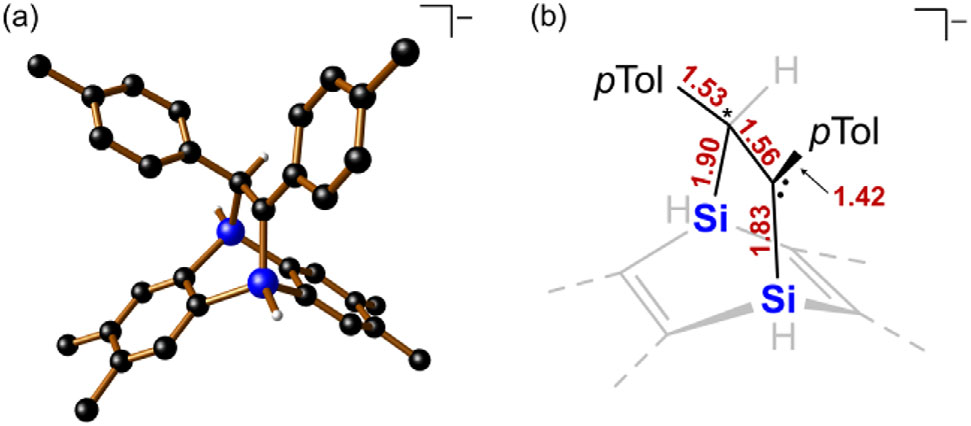
(a) Solid‐state structure of [**15**]^−^. Cations, solvent molecules, and H atoms except those bound to Si and to the interannular C_2_ bridge are omitted for clarity. H: light gray; C: black; Si: blue. (b) Schematic representation of the interannular C_2_ bridge with selected bond lengths indicated in Å.

SCXRD analysis of the 1D coordination polymer [K(THF)_0.5_][**15**] revealed a strongly folded DSA moiety with a dihedral angle *ϕ* = 112.6(2)°, nearly identical to that of the disilanide [**1**]^2−^ (Figure 4a). The bond between the bridgehead Si atom and the carbanionic center measures Si─C^−^ = 1.827(5) Å. Remarkably, the interannular C(sp^2^)─C(sp^3^) bridge (1.556(7) Å) is longer than a typical C(sp^3^)─C(sp^3^) single bond (1.54 Å) [81]. Furthermore, the bond between the carbanion and its *p*Tol substituent (1.423(6) Å) is significantly shorter than the central C(sp^2^)─C(sp^2^) bond in s‐*trans*‐1,3‐butadiene (1.454(1) Å) [82, 83], indicating substantial π character. This is consistent with the restricted rotation of the *p*Tol ring observed on the NMR timescale, due to partial delocalization of the negative charge into the adjacent 6π electron system.

## 3 Conclusion

In this work, we leveraged three distinctive features of aryl(hydro)silanes: (i) their propensity for base‐induced substituent exchange, (ii) the capacity of Si‐bonded H^−^ units to function as leaving groups in nucleophilic substitution reactions, and (iii) the facile reductive cleavage of Si─H bonds by alkali metals to generate nucleophilic silanide sites. Exploiting this reactivity profile, we achieved several main objectives: (i) a high‐yield, one‐pot synthesis of the sterically unencumbered 9,10‐dihydro‐9,10‐disilaanthracene (DSA) **1^2H^** by treating the readily accessible 1,2‐disilylbenzene **DSB** with substoichiometric quantities of MeLi; (ii) the subsequent MgH_2_ elimination with magnesium anthracene to afford the tricyclic framework **13**; and (iii) the reduction of **1^2H^** with excess K metal to furnish a rare disilanide species [80, 84, 85], which was effectively trapped via double adduct formation with the 9,10‐dihydro‐9,10‐diboraanthracene **8**, thereby yielding a **13**‐type species via an umpolung approach. Most significantly, the reduction of **1^2H^** with 2 equiv. of KC_8_ provides a convenient route to the monosilanide [**1^H^**]^−^, which can be described as an ambiphilic species possessing one nucleophilic and one electrophilic Si site. As these sites resist mutual quenching via head‐to‐tail dimerization, they remain accessible for reaction with external substrates. The resulting synthetic utility of [**1^H^**]^−^ was demonstrated by its conversion with *p*TolC≡C*p*Tol, yielding the tricyclic product K[**15**] containing a deprotonated 1,2‐ethanediyl bridge. The carbanionic center of K[**15**] serves as a versatile handle for further derivatization, such as *C*‐silylation. By introducing the DSA **1^2H^** and its anionic derivatives, we have expanded the broadly applicable class of 9,10‐dihydro‐9,10‐diheteroanthracenes by three additional promising members, for which a rich follow‐up chemistry can be anticipated.

## Conflicts of Interest

The authors declare no conflicts of interest.

## Supporting information



The authors have cited additional references within the Supporting Information [86, 87, 88, 89, 90, 91, 92, 93, 94, 95].

## Data Availability

The data that supports the findings of this study are available in the supplementary material of this article.

## References

[1] M. Davis and F. G. Mann , “The Synthesis and Stereochemistry of 5,10‐Disubstituted 5,10‐Dihydrophosphanthrens and Their Derivatives,” Journal of the Chemical Society (1964): 3770.

[2] H. Akutsu , M. Ogasawara , M. Saburi , K. Kozawa , and T. Uchida , “Structure and Characterization of 9,10‐Diethyl‐9,10‐Diphospha‐9,10‐Dihydroanthracene as an Electron Donor,” Bulletin of the Chemical Society of Japan 69 (1996): 1223.

[3] H. Yang , Q. Liang , C. Han , J. Zhang , and H. Xu , “A Phosphanthrene Oxide Host With Close Sphere Packing for Ultralow‐Voltage‐Driven Efficient Blue Thermally Activated Delayed Fluorescence Diodes,” Advanced Materials 29 (2017): 1700553. 10.1002/adma.201700553 28833794

[4] F. Gao , R. Du , C. Han , et al., “High‐Efficiency Blue Thermally Activated Delayed Fluorescence From Donor–Acceptor–Donor Systems via the Through‐Space Conjugation Effect,” Chemical Science 10 (2019): 5556. 31293740 10.1039/c9sc01240k PMC6553033

[5] S. N. Kessler and H. A. Wegner , “Lewis Acid Catalyzed Inverse Electron‐Demand Diels–Alder Reaction of 1,2‐Diazines,” Organic Letters 12 (2010): 4062. 20718452 10.1021/ol101701z

[6] S. N. Kessler , M. Neuburger , and H. A. Wegner , “Domino Inverse Electron‐Demand Diels–Alder/Cyclopropanation Reaction of Diazines Catalyzed by a Bidentate Lewis Acid,” Journal of the American Chemical Society 134 (2012): 17885. 23066957 10.1021/ja308858y

[7] Z. Lu , L. Schweighauser , H. Hausmann , and H. A. Wegner , “Metal‐Free Ammonia–Borane Dehydrogenation Catalyzed by a Bis(borane) Lewis Acid,” Angewandte Chemie International Edition 54 (2015): 15556. 26537288 10.1002/anie.201508360

[8] L. Schweighauser and H. A. Wegner , “Bis‐Boron Compounds in Catalysis: Bidentate and Bifunctional Activation,” Chemistry – A European Journal 22 (2016): 14094. 27490466 10.1002/chem.201602231

[9] T. Agou , J. Kobayashi , and T. Kawashima , “Dibenzophosphaborin: A Hetero‐π‐Conjugated Molecule With Fluorescent Properties Based on Intramolecular Charge Transfer Between Phosphorus and Boron Atoms,” Organic Letters 7 (2005): 4373. 16178536 10.1021/ol051537q

[10] T. Agou , J. Kobayashi , and T. Kawashima , “Tuning of the Optical Properties and Lewis Acidity of Dibenzopnictogenaborins by Modification on Bridging Main Group Elements,” Inorganic Chemistry 45 (2006): 9137. 17054375 10.1021/ic061055q

[11] J. Kobayashi , T. Agou , and T. Kawashima , “Synthesis, Optical Properties, and Reactivities of a Dibenzophosphaborin and Its Derivatives,” Phosphorus, Sulfur, and Silicon and the Related Elements 183 (2008): 389.

[12] P. Xie , F. Zhang , Z. Cai , Y. Zheng , L. Wu , and L. He , “Synthesis of Cyclic Phosphonium‐Borate Compounds Through Reaction of Benzynes and Frustrated Lewis Pairs,” Organic Chemistry Frontiers 12 (2025): 1244.

[13] M. H. Lee , T. Agou , J. Kobayashi , T. Kawashima , and F. P. Gabbaï , “Fluoride Ion Complexation by a Cationic Borane in Aqueous Solution,” Chemical Communications (2007): 1133. 17347716 10.1039/b616814k

[14] T. Agou , J. Kobayashi , Y. Kim , F. P. Gabbaï , and T. Kawashima , “Phase Transfer of Fluoride Ion by Phosphonioborins,” Chemistry Letters 36 (2007): 976.

[15] T. Ito , N. Iwasawa , and J. Takaya , “Photo‐Promoted Skeletal Rearrangement of Phosphine–Borane Frustrated Lewis Pairs Involving Cleavage of Unstrained C–C σ‐Bonds,” Angewandte Chemie International Edition 59 (2020): 11913. 32329933 10.1002/anie.202004444

[16] E. von Grotthuss , M. Diefenbach , M. Bolte , H.‐W. Lerner , M. C. Holthausen , and M. Wagner , “Reversible Dihydrogen Activation by Reduced Aryl Boranes as Main‐Group Ambiphiles,” Angewandte Chemie International Edition 55 (2016): 14067. 27709767 10.1002/anie.201608324

[17] E. von Grotthuss , S. E. Prey , M. Bolte , H.‐W. Lerner , and M. Wagner , “Dual Role of Doubly Reduced Arylboranes as Dihydrogen‐ and Hydride‐Transfer Catalysts,” Journal of the American Chemical Society 141 (2019): 6082. 30875474 10.1021/jacs.9b01998

[18] S. E. Prey and M. Wagner , “Threat to the Throne: Can Two Cooperating Boron Atoms Rival Transition Metals in Chemical Bond Activation and Catalysis?,” Advanced Synthesis & Catalysis 363 (2021): 2290.

[19] S. E. Prey , C. Herok , F. Fantuzzi , et al., “Multifaceted Behavior of a Doubly Reduced Arylborane in B–H‐Bond Activation and Hydroboration Catalysis,” Chemical Science 14 (2023): 849. 36755708 10.1039/d2sc05518j PMC9890859

[20] H. Budy , S. E. Prey , C. D. Buch , M. Bolte , H.‐W. Lerner , and M. Wagner , “Nucleophilic Borylation of Fluorobenzenes With Reduced Arylboranes,” Chemical Communications 58 (2022): 254. 10.1039/d1cc06225e 34881754

[21] C. D. Buch , A. Virovets , E. Peresypkina , et al., “Planarity Is Not Plain: Closed‐ vs Open‐Shell Reactivity of a Structurally Constrained, Doubly Reduced Arylborane toward Fluorobenzenes,” Journal of the American Chemical Society 147 (2025): 20071. 40437997 10.1021/jacs.5c05588 PMC12164271

[22] D. J. Hajdasz and R. R. Squires , “Hypervalent Silicon Hydrides: SiH_5_ ^−^ ,” Journal of the American Chemical Society 108 (1986): 3139.

[23] C. Chuit , R. J. P. Corriu , C. Reye , and J. C. Young , “Reactivity of Penta‐ and Hexacoordinate Silicon Compounds and Their Role as Reaction Intermediates,” Chemical Reviews 93 (1993): 1371.

[24] F. D. Greene , S. L. Misrock , and J. R. Wolfe , “The Structure of Anthracene Photodimers,” Journal of the American Chemical Society 77 (1955): 3852.

[25] K. A. Abboud , S. H. Simonsen , and R. M. Roberts , “Redetermination of the Structure of Bi(9,10‐Dihydro‐9,10‐anthracenediyl) at 198 K,” Acta Crystallographica Section C 46 (1990): 2494.

[26] T. Okutsu , K. Isomura , N. Kakinuma , et al., “Laser‐Induced Morphology Control and Epitaxy of Dipara‐Anthracene Produced From the Photochemical Reaction of Anthracene,” Crystal Growth & Design 5 (2005): 461.

[27] M. Kira , E. Kwon , C. Kabuto , and K. Sakamoto , “X‐Ray Crystal Structure and Dynamic Behavior of Pentafluoro‐9,10‐Disila‐9,10‐Dihydroanthracene Anion Salts Having Transannular 1,4‐Fluorine Bridge,” Chemistry Letters 28 (1999): 1183.

[28] D. Hartmann , M. Schädler , and L. Greb , “Bis(catecholato)Silanes: Assessing, Rationalizing, and Increasing Silicon's Lewis Superacidity,” Chemical Science 10 (2019): 7379. 31489160 10.1039/c9sc02167a PMC6713871

[29] K. Nishiyama , M. Oba , H. Takagi , et al., “Radical Isomerization of 9,10‐Dihydro‐9,10‐Disilaanthracene Derivatives: Stabilization Factor of Silyl Radicals,” Journal of Organometallic Chemistry 626 (2001): 32.

[30] W. Ando , K. Hatano , and R. Urisaka , “Synthesis and Reduction of the 9,10‐Disilaanthracene Dimer,” Organometallics 14 (1995): 3625.

[31] M. Oba , T. Kondo , K. Tanaka , S. Koguchi , and K. Nishiyama , “Synthesis and Structure of 9,10‐Bis(2,4,6‐Triisopropylphenyl)‐9,10‐Dihydro‐9,10‐Disilaanthracene,” Journal of Organometallic Chemistry 696 (2011): 982.

[32] T. Kuribara , S. Ishida , T. Kudo , and S. Kyushin , “Microwave‐Assisted Efficient One‐Pot Synthesis of 9‐Phenyl‐9,10‐Disilatriptycene and Its Bridgehead Functionalization,” Organometallics 32 (2013): 2092.

[33] J. Y. Corey and W. Z. McCarthy , “Simplified Approach to Silaanthrones and Disilaanthracenes,” Journal of Organometallic Chemistry 271 (1984): 319.

[34] W. Z. McCarthy , J. Y. Corey , and E. R. Corey , “Group 4 9,10‐Dihydro‐9,10‐Diheteroanthracenes: Synthesis and Structure,” Organometallics 3 (1984): 255.

[35] K. M. Welsh and J. Y. Corey , “Separation of *Cis* and *Trans* Isomers of 1,4‐Disilacyclohexadienes and Related 9,10‐Dihydro‐9,10‐Disilaanthracenes,” Organometallics 6 (1987): 1393.

[36] A. Kawachi , A. Tani , K. Machida , and Y. Yamamoto , “ *o*‐(Fluorodimethylsilyl)phenyllithium as a Versatile Reagent for Preparation of Unsymmetrical, Silicon‐Functionalized *o*‐Disilylbenzenes,” Organometallics 26 (2007): 4697.

[37] A. Nakamoto , Y. Yamamoto , and A. Kawachi , “Synthesis and Structures of Edge‐Functionalized Dihydrodisilaanthracene and Tetrahydrotetrasilapentacene Compounds,” Chemistry Letters 46 (2017): 1760.

[38] T. Nakashima , M. Shimada , Y. Kurihara , et al., “Fluorescence and Phosphorescence of a Series of Silicon‐Containing Six‐Membered‐Ring Molecules,” Journal of Organometallic Chemistry 805 (2016): 27.

[39] Chatgilialoglu et al. reported the use of 9,10‐dihydro‐9,10‐disilaanthracene, (*o*‐C_6_H_4_–SiH_2_)_2_, as a reducing agent in Barton‐McCombie reactions, but provided no synthetic or analytical details: T. Gimisis , M. Ballestri , C. Ferreri , C. Chatgilialoglu , R. Boukherroub , and G. Manuel , “5,10‐Dihydro‐Silanthrene as a Reagent for the Barton–McCombie Reaction,” Tetrahedron Letters 36 (1995): 3897.

[40] Sasamori et al. detected (*o*‐C_6_H_4_–SiH_2_)_2_ as a side product upon treatment of (*o*‐C_6_H_4_–Si(H)F)_2_ with exc. KF/18‐crown‐6 at 50 °C. They noted: “Although definitive identification of” this compound “was challenging due to its lability toward air, making isolation extremely difficult, the observation of a characteristic triplet signal at δ = −49 ppm (*J* _SiH_ = 201 Hz) in the ^1^H‐coupled ^29^Si NMR spectrum strongly supports the assigned structure”. While this NMR signal is consistent with the NMR data obtained for **1^2H^ **, we did not observe pronounced air sensitivity: M. Kawamoto and T. Sasamori , “9,10‐Difluoro‐9,10‐Disila‐9,10‐Dihydroanthracene,” Inorganics 14 (2026): 23.

[41] I. Georg , J. Teichmann , M. Bursch , et al., “Exhaustively Trichlorosilylated C_1_ and C_2_ Building Blocks: Beyond the Müller–Rochow Direct Process,” Journal of the American Chemical Society 140 (2018): 9696. 29985607 10.1021/jacs.8b05950

[42] M. Schmidt , J. Gilmer , A. Virovets , M. Bolte , H.‐W. Lerner , and M. Wagner , “Adjusting the Number of Functional Groups in Vicinal Bis(trichlorosilylated) Benzenes,” Chemistry – A European Journal 30 (2024): e202402998. 39222351 10.1002/chem.202402998

[43] M. Schmidt , N. H. H. Hürtgen , A. Virovets , H.‐W. Lerner , and M. Wagner , “Complete Series of 1,2‐Bis(trihalogenosilyl)Benzenes (F, Cl, Br, I): A Platform for Cooperating Lewis‐Acidic Sites in Close Quarters,” Zeitschrift für Anorganische und Allgemeine Chemie 652 (2025): e202500154.

[44] B. Wei , H. Li , J. Yin , W.‐X. Zhang , and Z. Xi , “Reaction of Dilithio Reagents With PhSiH_3_: Formation of Siloles and 3‐Silacyclopentenes,” Journal of Organic Chemistry 80 (2015): 8758. 26270413 10.1021/acs.joc.5b01595

[45] For completeness, we note that MeLi can serve as a source of LiH even in the absence of other reaction partners; however, such LiH release has thus far only been reported to occur under thermolytic conditions: J. R. Baran Jr. and R. J. Lagow , “Some Observations on the Pyrolysis of Methyllithium,” Journal of Organometallic Chemistry 427 (1992): 1.

[46] An alternative pathway involving direct cyclocondensation of **Int1** via head‐to‐tail dimerization cannot be excluded. However, the transient nature and presumed low steady‐state concentration of **Int1** render this scenario less probable than the reaction of **Int1** with the more abundant **DSB**. Furthermore, resonances assignable to **Int2** were detected by in situ NMR spectroscopy.

[47] **DSB** remained unreactive toward commercial LiH. This behavior is consistent with the well‐documented lower reactivity of LiH compared to KH: C. A. Brown , “Kaliation. I. Remarkable Fast Reaction of Potassium Hydride With Amines and Other Feeble Organic Acids. Convenient Rapid Route to Elusive New Superbases,” Journal of the American Chemical Society 95 (1973): 982.

[48] We assume that successful cyclocondensation of **DSB** depends on the availability of finely divided, in situ‐generated LiH: Y. Fan , W. Li , Y. Zou , S. Liao , and J. Xu , “Chemical Reactivity and Thermal Stability of Nanometric Alkali Metal Hydrides,” Journal of Nanoparticle Research 8 (2006): 935.

[49] M. A. Ring and D. M. Ritter , “Preparation and Reactions of Potassium Silyl,” Journal of the American Chemical Society 83 (1961): 802.

[50] B. Becker , R. J. P. Corriu , C. Guérin , and B. J. L. Henner , “Hypervalent Silicon Hydrides: Evidence for Their Intermediacy in the Exchange Reactions of Di‐ and Tri‐Hydrogenosilanes Catalysed by Hydrides (NaH, KH, and LiAlH_4_),” Journal of Organometallic Chemistry 369 (1989): 147.

[51] M. Itoh , K. Inoue , J. Ishikawa , and K. Iwata , “Disproportionation Reactions of Organohydrosilanes in the Presence of Base Catalysts,” Journal of Organometallic Chemistry 629 (2001): 1.

[52] C. Appelt , H. Westenberg , F. Bertini , et al., “Geminal Phosphorus/Aluminum‐Based Frustrated Lewis Pairs: C–H Versus C = C Activation and CO_2_ Fixation,” Angewandte Chemie International Edition 50 (2011): 3925. 21425419 10.1002/anie.201006901

[53] E. Alig , I. Georg , I. Sänger , L. Fink , M. Wagner , and H.‐W. Lerner , “Synthesis and Structure of the Donor‐Free Potassium Silanide K[SiPh_3_],” Zeitschrift für Naturforschung B 74 (2019): 153.

[54] J. Beckmann , D. Dakternieks , A. Duthie , and E. R. T. Tiekink , “Synthesis and Reactivity Towards DIBAL‐H of Cyclo‐Siloxanes Cyclo‐[R_2_SiOSi(O*t*‐Bu)_2_O]_2_, Cyclo‐(*t*‐BuO)_2_Si(OSiR_2_)_2_O, and Cyclo‐R_2_Si[OSi(O*t*‐Bu)_2_]_2_O (R = Me, Ph),” Zeitschrift für Anorganische und Allgemeine Chemie 628 (2002): 2948.

[55] B. Pribanic , M. Trincado , F. Eiler , M. Vogt , A. Comas‐Vives , and H. Grützmacher , “Hydrogenolysis of Polysilanes Catalyzed by Low‐Valent Nickel Complexes,” Angewandte Chemie International Edition 59 (2020): 15603. 32049402 10.1002/anie.201907525

[56] J. P. Foster and F. Weinhold , “Natural Hybrid Orbitals,” Journal of the American Chemical Society 102 (1980): 7211.

[57] The qualitative ^13^C chemical shift differences upon conversion of **1^2H^ ** to K_2_[**1**] are in agreement with published trends for the transition from phenylsilanes to phenylsilanides: E. Buncel , T. K. Venkatachalam , B. Eliasson , and U. Edlund , “NMR Studies of Phenylsilyl Anions: Evaluation of Ion Pairing and Charge Distribution,” Journal of the American Chemical Society 107 (1985): 303.

[58] The average values with standard deviations in square brackets were calculated from the individual values stated in the cif files.

[59] Deposition numbers 2539949 (for **1^2H^ **), 2539950 (for K_2_[**1**]), 2539953 (for *cis*‐**2**), 2539954 (for *cis*‐**3**), 2539955 (for **4**), 2539956 (for **5**), 2539957 (for **6**), 2539958 (for **7**), 2540095 (for K_2_[**10**]), 2539952 (for [K(18‐c‐6)(THF)_2_][K(18‐c‐6)]_3_[**10**]_2_), 2539959 (for **13**), 2539960 (for K[**15**]), 2539961 (for **VI**), and 2539962 (for **VII**) contain the supplementary crystallographic data for this paper. These data are provided free of charge by the joint Cambridge Crystallographic Data Centre and Fachinformationszentrum Karlsruhe Access Structures service.

[60] Consistent with our crystallographic findings for K_2_[**1**], a low‐temperature NMR study on Li_2_[(*o*‐C_6_H_4_–SiMe)_2_], augmented by quantum‐chemical calculations, suggested a *C* _2v_‐symmetric dianion. In this model, one Li^+^ ion bridges the silanide sites on the convex face, while the second Li^+^ ion resides on the opposite, concave face: K. Hatano , K. Morihashi , O. Kikuchi , and W. Ando , “Lithium‐Bridged Bis(silyl Anion) of 9,10‐Dimethyl‐9,10‐Disilaanthracene,” Chemistry Letters 26 (1997): 293.

[61] J. B. Lambert and M. Urdaneta‐Pérez , “Diastereotopic Groups in Silyllithium and Germyllithium Compounds. Slow Inversion About Silicon and Germanium,” Journal of the American Chemical Society 100 (1978): 157.

[62] M. R. Nimlos and G. B. Ellison , “Photoelectron Spectroscopy of Silicon Trihydride and Trideuteride Anions,” Journal of the American Chemical Society 108 (1986): 6522.

[63] Comparable silylation reactions have previously been reported by Ando et al. (cf. Ref. [30]). However, as the disilanide [**C**]^2−^ in their study was generated via reductive cleavage of precursor **B** featuring *cis*‐positioned Si–Si bonds, the formation of *cis*/*trans* isomers was not a matter of concern. Moreover, this study did not provide definitive proof of the proposed product configurations by SCXRD.

[64] E. von Grotthuss , S. E. Prey , M. Bolte , H.‐W. Lerner , and M. Wagner , “Selective CO_2_ Splitting by Doubly Reduced Aryl Boranes to Give CO and [CO_3_]^2−^ ,” Angewandte Chemie International Edition 57 (2018): 16491. 30320950 10.1002/anie.201811135

[65] P. L. Lückert , J. Gilmer , A. Virovets , H.‐W. Lerner , and M. Wagner , “Donor‐Free 9,10‐Dihydro‐9,10‐Dialuminaanthracenes,” Chemical Science 16 (2025): 147. 10.1039/d4sc06940d PMC11609976 39629489

[66] M. Oba and K. Nishiyama , “9,10‐Dihydro‐9,10‐Disilaanthracenes as a New Radical‐Based Reducing Agent: Importance of Transannular Interaction Between Silyl Radical and Silicon Atom,” Journal of the Chemical Society, Chemical Communications (1994): 1703.

[67] M. Oba , Y. Kawahara , R. Yamada , H. Mizuta , and K. Nishiyama , “9,10‐Dihydro‐9‐Sila‐10‐Heteroanthracenes as New Radical‐Based Reducing Agents,” Journal of the Chemical Society, Perkin Transactions 2 (1996): 1843.

[68] S. Kyushin , T. Shinnai , T. Kubota , and H. Matsumoto , “Synthesis, Structures, and Isomerization of 9,10‐Di‐*tert*‐butyl‐9,10‐Dihydro‐9,10‐Disilaanthracenes,” Organometallics 16 (1997): 3800.

[69] A. Lorbach , M. Bolte , H.‐W. Lerner , and M. Wagner , “Dilithio 9,10‐Diborataanthracene: Molecular Structure and 1,4‐Addition Reactions,” Organometallics 29 (2010): 5762.

[70] J. D. Rich and R. West , “Evidence for the Intermediacy of Hexamethyl‐1,4‐Disilabenzene,” Journal of the American Chemical Society 104 (1982): 6884.

[71] Note that a continuum exists between polar (S_N_1, S_N_2) and electron‐transfer reactions, as ‘all so‐called two‐electron (or polar) pathways actually involve the shift of a single electron and are far more closely related to the established SET pathways than has been recognized till now’: A. Pross, “The Single Electron Shift as a Fundamental Process in Organic Chemistry: The Relationship Between Polar and Electron‐Transfer Pathways,” Accounts of Chemical Research 18 (1985): 212. Beyond this general consideration, Mg[**12**] may in principle serve not only as a source of the dinucleophile [C_14_H_10_]^2−^, but also as a reducing system capable of generating highly reactive Mg(0) in situ, which, similar to [C_14_H_10_]^2−^, could induce parallel radical pathways via reductive activation: S. Harvey, C. L. Raston, “Polymer Supported ‘Magnesium(Anthracene)’: Effective in Forming Benzylic Grignard Reagents (via Electron Transfer Reactions)” Journal of the Chemical Society, Chemical Communications (1988): 652. However, the use of THF as solvent argues against a pronounced radical character in the reaction between **1^2H^ ** and Mg[**12**]. THF is an efficient H‐atom donor and would therefore be expected to partially quench a putative radical pair. Experimentally, we observe neither incorporation of additional H atoms at the anthracene unit or at the Si sites, nor any evidence for competing radical–radical coupling involving a THF‐derived radical species, as reported in other contexts in the literature (cf. Ref. [21]). Moreover, control reactions of **1^2H^ ** with 2 and 4 equiv. MeLi in [D_8_]THF afforded the corresponding DSAs with two Si(H)Me and two SiMe_2_ groups, respectively (NMR‐spectroscopic control). While MeLi is a strong nucleophile, it is significantly less prone to radical reactivity than Mg[**12**].Taken together, a predominantly closed‐shell mechanism, in which **1^2H^ ** acts as a twofold electrophile, appears most plausible at present.

[72] H. Nöth and B. Wrackmeyer , Nuclear Magnetic Resonance Spectroscopy of Boron Compounds, in NMR Basic Principles and Progress, eds. P. Diehl , E. Fluck , and R. Kosfeld , (Springer, 1978), 74–101.

[73] J. Teichmann , M. Bursch , B. Köstler , et al., “Trapping Experiments on a Trichlorosilanide Anion: A Key Intermediate of Halogenosilane Chemistry,” Inorganic Chemistry 56 (2017): 8683. 28318239 10.1021/acs.inorgchem.7b00216

[74] J. Heller , C. D. Buch , A. V. Virovets , et al., “Synthesis of Perhalogenated Silylboranes (X = Cl, I) and Their Application in Regiodivergent Alkene Silaboration,” Chemical Science 16 (2025): 20329. 41064321 10.1039/d5sc06234a PMC12501792

[75] J. Guo , M. Yan , and D. W. Stephan , “Frustrated Lewis Pair Chemistry of Alkynes,” Organic Chemistry Frontiers 11 (2024): 2375.

[76] N. F. McKinley and D. F. O'Shea , “Carbolithiation of Diphenylacetylene as a Stereoselective Route to (*Z*)‐Tamoxifen and Related Tetrasubstituted Olefins,” Journal of Organic Chemistry 71 (2006): 9552. 17137396 10.1021/jo061949s

[77] J. Maddaluno , C. Fressigné , and M. Durandetti , “Intramolecular Carbolithiation of Alkynes: Observation, Mechanism, Applications,” Synlett 37 (2025): 440.

[78] M. Oba , Y. Watanabe , K. Iwai , H. Ohtaki , and K. Nishiyama , “Reaction of 9,10‐Dimethyl‐9,10‐Dihydro‐9,10‐Disilaanthracene With Alkyne in the Presence of Palladium,” Journal of Organometallic Chemistry 629 (2001): 44.

[79] E. N. Gladyshev , E. A. Fedorova , G. A. Razuvaev , L. O. Yuntila , and N. S. Vyazankin , “Reactions of Triethylsilylpotassium and Related Compounds With Vinyltrimethylsilane in Benzene Solution,” Journal of Organometallic Chemistry 97 (1975): 25.

[80] C. Präsang and D. Scheschkewitz in Functional Molecular Silicon Compounds II, Structure and Bonding, ed. D. Scheschkewitz , (Springer, 2013), 1–47.

[81] J. Clayden , N. Greeves , and S. Warren , Organic Chemistry, 2nd ed (Oxford University Press, 2012), 295.

[82] N. C. Craig , P. Groner , and D. C. McKean , “Equilibrium Structures for Butadiene and Ethylene: Compelling Evidence for π‐Electron Delocalization in Butadiene,” Journal of Physical Chemistry A 110 (2006): 7461. 16759136 10.1021/jp060695b

[83] P. D. Jarowski , M. D. Wodrich , C. S. Wannere , P. V. R. Schleyer , and K. N. Houk , “How Large is the Conjugative Stabilization of Diynes?,” Journal of the American Chemical Society 126 (2004): 15036. 15547994 10.1021/ja046432h

[84] Y. Heider , P. Willmes , V. Huch , M. Zimmer , and D. Scheschkewitz , “Boron and Phosphorus Containing Heterosiliconoids: Stable p‐ and n‐Doped Unsaturated Silicon Clusters,” Journal of the American Chemical Society 141 (2019): 19498. 31726826 10.1021/jacs.9b11181

[85] T. Iwamoto , D. Yin , A. Kobayashi , et al., “Lithiated 1,3‐Disilabicyclo[1.1.0]Butanes Synthesized via Selective Cleavage of Exocyclic Si–Si Bonds on Bridgehead Silicon Atoms,” Organometallics 40 (2021): 2557.

[86] B. Bogdanović , S.‐T. Liao , R. Mynott , K. Schlichte , and U. Westeppe , “Rate of Formation and Characterization of Magnesium Anthracene,” Chemische Berichte 117 (1984): 1378.

[87] G. R. Fulmer , A. J. M. Miller , N. H. Sherden , et al., “NMR Chemical Shifts of Trace Impurities: Common Laboratory Solvents, Organics, and Gases in Deuterated Solvents Relevant to the Organometallic Chemist,” Organometallics 29 (2010): 2176.

[88] E. A. Chernyshev , N. G. Komalenkova , L. N. Shamshin , and S. A. Shchepinov , “Silicon‐Containing Heterocyclic Compounds. IX. Preparation of Silicon‐Containing Heterocycles by the Pyrolysis of Phenylchlorosilanes,” Zhurnal Obshchei Khimii 41 (1971): 843.

[89] E. A. Chernyshev and N. G. Komalenkova , “The Thermal Gas‐Phase Synthesis of Heterocyclic Compounds With One or Several Silicon Atoms in the Ring,” Russian Chemical Reviews 58 (1989): 559.

[90] K. Thorshaug , O. Swang , I. M. Dahl , and A. Olafsen , “An Experimental and Theoretical Study of Spin–Spin Coupling in Chlorosilanes,” Journal of Physical Chemistry A 110 (2006): 9801. 16898680 10.1021/jp060704g

[91] P. Molinillo , A. Gálvez Del Postigo , M. Puyo , et al., “Selective H/D Exchange in E–H (E = Si, Ge, Sn) Bonds Catalyzed by 1,2,3‐Triazolylidene‐Stabilized Nickel Nanoparticles,” Inorganic Chemistry 64 (2025): 8125. 40240314 10.1021/acs.inorgchem.5c00216 PMC12135044

[92] S. Bourg , B. Boury , F. H. Carré , and R. J. P. Corriu , “Synthesis of New Silicon–Cobalt Clusters,” Organometallics 17 (1998): 167.

[93] G. M. Sheldrick , “A Short History of SHELX,” Acta Crystallographica Section A: Foundations of Crystallography 64 (2008): 112. 18156677 10.1107/S0108767307043930

[94] G. M. Sheldrick , “SHELXT – Integrated Space‐Group and Crystal‐Structure Determination,” Acta Crystallographica Section A: Foundations and Advances 71 (2015): 3. 25537383 10.1107/S2053273314026370 PMC4283466

[95] G. M. Sheldrick , “Crystal Structure Refinement With SHELXL,” Acta Crystallographica Section C: Structural Chemistry 71 (2015): 3. 25567568 10.1107/S2053229614024218 PMC4294323

