# Supplementary material for: Polarity on Demand: Nucleophilic, Electrophilic, and Ambiphilic Reactivity at a 9,10‐Dihydro‐9,10‐Disilaanthracene Platform
Source: Chemistry. 2026 May 18;32(33):e71100. doi: 10.1002/chem.71100 (PMC13532117; doi:10.1002/chem.71100)
Supplement: Supplementary file 1 [file CHEM-32-e71100-s001.pdf]

Supporting Information

Polarity on Demand: Nucleophilic, Electrophilic, and Ambiphilic Reactivity at a 9,10-Dihydro-9,10-disilaanthracene Platform

Moritz Schmidt, Alexander Virovets, Eugenia Peresypkina, Hans-Wolfram Lerner, and Matthias Wagner*

Institut für Anorganische und Analytische Chemie, Goethe-Universität Frankfurt, Max-von-Laue-Straße 7, D-60438 Frankfurt (Main), Germany

*To whom correspondence should be addressed. Email: matthias.wagner@chemie.uni-frankfurt.de

Table of Contents

1. Experimental details and characterization data ... 3
    1.1. Synthesis of **1**^{2H} ... 4
        1.1.1. Notes on byproduct formation ... 4
    1.2. Synthesis of K_2[**1**] ... 9
    1.3. Synthesis of K[**1**^{H}] ... 12
    1.4. Reactivity of K_2[**1**] toward mono- and difunctional organochlorosilanes ... 13
        1.4.1. Synthesis of *cis*-**2** ... 13
        1.4.2. Synthesis of *cis*-**3** ... 14
        1.4.3. Synthesis of **4** ... 15
        1.4.4. Synthesis of **5** ... 16
    1.5. Reactivity of K[**1**^{H}] toward mono- and difunctional organochlorosilanes ... 17
        1.5.1. Synthesis of **6** ... 17
        1.5.2. Synthesis of **7** ... 18
    1.6. Synthesis of **13** ... 19
    1.7. Reactivity of K[**1**^{H}] toward diaryl alkynes ... 20
        1.7.1. Protonation of K[**15**] with $HNTf_2$ (preparative scale) ... 22
        1.7.2. Trapping of K[**15**^{Ans}] with Me_3SiCl (preparative scale) ... 24
2. Plots of NMR spectra ... 26
3. Single-crystal X-ray structure analyses ... 78
    3.1. Crystal structure of **1**^{2H} ... 86
    3.2. Crystal structure of **VI** ... 86
    3.3. Crystal structure of **VII** ... 87
    3.4. Crystal structure of K_2[**1**] ... 88
    3.5. Crystal structure of K_2[**10**] ... 90
    3.6. Crystal structure of [K(18-c-6)$(THF)_2$][K(18-c-6)]$_3$[**10**]$_2$ ... 91
    3.7. Crystal structure of *cis*-**2** ... 93
    3.8. Crystal structure of *cis*-**3** ... 94
    3.9. Crystal structure of **4** ... 95
    3.10. Crystal structure of **5** ... 95
    3.11. Crystal structure of **6** ... 96
    3.12. Crystal structure of **7** ... 97
    3.13. Crystal structure of **13** ... 98
    3.14. Crystal structure of K[**15**] ... 98
    3.15. Selected geometrical characteristics of the DSA fragment ... 100
4. References ... 102

1. Experimental details and characterization data

General Considerations. All reactions, manipulations, and analyses of air- and moisture-sensitive compounds were carried out under an atmosphere of dry argon or nitrogen using Schlenk techniques or in an argon- or nitrogen-filled glovebox. *n*-Hexane and benzene (C_6H_6) were distilled from Na metal, diethyl ether (Et_2O) and tetrahydrofuran (THF) were distilled from Na/benzophenone, and dichloromethane (CH_2Cl_2) was distilled from calcium hydride (CaH_2) prior to use. THF-d_8 was purchased from Eurisotop, dried over Na/K alloy (without benzophenone, 1–2 d), degassed by applying three freeze-pump-thaw cycles, and stored over molecular sieves (3 Å). $CDCl_3$ (Eurisotop) and CD_2Cl_2 (Deutero GmbH) were dried over molecular sieves (3 Å). KC_8 was prepared by combining potassium metal and finely ground graphite in a 1:8 molar ratio in a Schlenk flask and stirring the mixture vigorously at 100 °C for 4 h. The resulting bronze-colored solid was used as obtained. Me_3SiCl and Me_2SiCl_2 were purchased commercially, dried over CaH_2, and distilled prior to use. All other commercially available reagents were used as received. The following compounds were synthesized according to literature procedures: 1,2-bis(silyl)-4,5-dimethylbenzene (**DSB**),[S1] 9,10-dimethyl-9,10-dihydro-9,10-diboraanthracene (**8**),[S2] 9,10-dimethyl-9,10-dihydro-9,10-dialuminaanthracene (**9**),[S3] and (9,10-dihydro-9,10-anthracenediyl)-tris(tetrahydrofuran)magnesium (Mg[**12**]).[S4]

NMR Experiments. NMR spectra were recorded at 298 K using the following spectrometers: Bruker Avance™ III 500 HD and Bruker Avance™ DRX 600. Chemical shift values are reported in ppm and referenced to (residual) solvent signals ($^1H/^{13}C\{^1H\}$; $CDCl_3$: δ = 7.26/77.16; CD_2Cl_2: δ = 5.32/53.84; THF-d_8: δ = 3.58/67.21)[S5] or to an external standard ($^{29}Si/^{29}Si\{^1H\}$: $SiMe_4$). Abbreviations: s = singlet, d = doublet, t = triplet, tt = triplet of triplets, m = multiplet, dm = doublet of multiplets, tm = triplet of multiplets, br = broad. Unless stated otherwise, resonance assignments were supported by ^1H–^{13}C HMBC, ^1H–^{13}C HSQC, ^1H–^{29}Si HMBC, and ^1H–^{29}Si HSQC NMR experiments. The numbering schemes correspond to the nomenclature given in the reaction schemes.

GC-MS Measurements. GC–MS data were recorded on a Shimadzu GCMS-QP2010 SE system. Separations were performed on a Restek Rtx-200 capillary column (60 m × 0.32 mm i.d., 1.50 µm film thickness). Samples were prepared by dissolving the analyte in $CHCl_3$. To avoid detector overloading, a solvent cut was applied. The injector temperature was set to 230 °C, and injections were performed in split mode (1:10). He was used as the carrier gas at a flow rate of 1.86 mL min^{-1}. The oven temperature program was as follows: initial temperature 50 °C (held for 1 min), ramped at steps of 10 °C min^{-1} to 250 °C (held for 40 min), and then ramped at 25 °C min^{-1} to 270 °C (held for 5 min). Mass spectra were acquired in positive-ion electron ionization mode (EI$^+$, 70 eV) over the range *m/z* 30–800. Retention times are reported in minutes; peak areas are given as relative values (area%) and were not corrected for response factors.

HRMS Measurements. High-resolution mass spectra were recorded at TU Darmstadt using a Bruker Orbitrap Exploris mass spectrometer (EI) and at Heidelberg University using a JEOL AccuTOF GCx mass spectrometer (EI).

Elemental Analysis. Elemental analyses (%CHN) were performed at the University of Gießen using a Thermo Fisher Scientific FlashEA 1112 elemental analyzer.

1.1. Synthesis of 1^{2H}

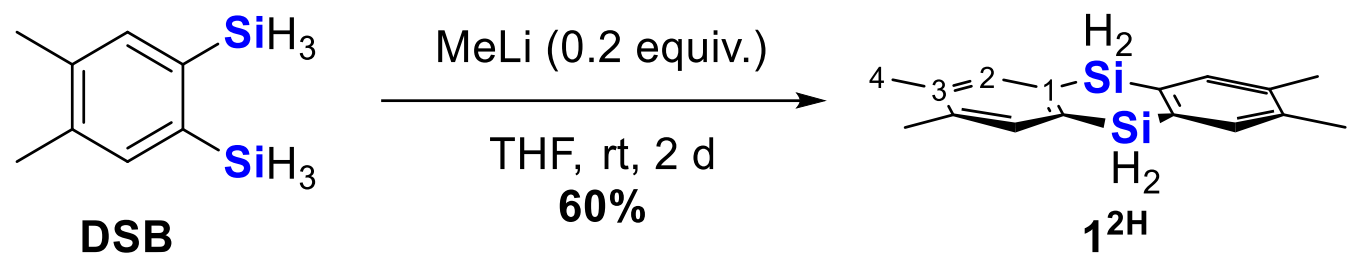

Safety Considerations

The synthesis of **1^{2H}** involves the formation of silane (SiH_4), a pyrophoric and highly flammable gas that can ignite spontaneously upon contact with air. Appropriate precautions must therefore be taken to avoid the accumulation of SiH_4 and to ensure its safe handling under inert conditions.

DSB (2.50 g, 15.0 mmol) was placed in a reaction flask and dissolved in THF (20 mL). A solution of MeLi in diethoxymethane (3.1 M, 0.97 mL, 3.0 mmol) was added dropwise with stirring at room temperature, resulting in the formation of a colorless, turbid mixture. After stirring the mixture for 2 d, volatile components were removed under reduced pressure. The residue was dried at 100 °C under dynamic vacuum (10^{-3} mbar) and subsequently sublimed at 150 °C/10^{-3} mbar. The sublimate was washed with *n*-pentane (2 × 2 mL), and the resulting colorless solid was dried under oil-pump vacuum to afford **1^{2H}** as a colorless solid. Yield: 1.21 g (4.51 mmol, 60% based on 0.5 equiv. of **DSB**).

Single crystals of **1^{2H}** suitable for X-ray diffraction were grown by slow evaporation of a solution of **1^{2H}** in *n*-hexane under ambient atmosphere.

^{1}H NMR (500.2 MHz, $CDCl_3$): δ = 7.55 (s, 4H, H-2), 4.85 (s, 4H, SiH_2), 2.31 (s, 12H, H-4).

^{13}C{^{1}H} NMR (125.8 MHz, $CDCl_3$): δ = 137.9 (C-3), 136.9 (C-2), 135.2 (C-1), 19.9 (C-4).

^{29}Si{^{1}H} NMR (99.4 MHz, $CDCl_3$): δ = –49.8.

^{29}Si NMR (99.4 MHz, $CDCl_3$): δ = –49.8 (tt, $^{1}J_{SiH}$ = 198.7 Hz, $^{3}J_{SiH}$ = 6.1 Hz).

HRMS (EI): *m/z* calcd for $C_{16}H_{20}Si_2$ ($[M]^{\bullet+}$) 268.1098; found: 268.1099.

EA (%): calcd for $C_{16}H_{20}Si_2$ [268.50]: C 71.57, H 7.51; found: C 70.38, H 7.46.

Notes on literature syntheses of parent 9,10-dihydro-9,10-disilaanthracene

The first synthesis of the parent (unsubstituted) 9,10-dihydro-9,10-disilaanthracene (DSA) proceeded via 9,9,10,10-tetrachloro-9,10-dihydro-9,10-disilaanthracene, accessed by cyclization of dichloro(phenyl)(*o*-trichlorosilylphenyl)silane at 680 °C. Under these demanding experimental conditions, low yields of about 15–30% were obtained.[S6,S7] Subsequent reduction with $LiAlH_4$ afforded DSA.[S6] Although ring-closure strategies via dilithio or di-Grignard intermediates derived from bis(*o*-halophenyl)silanes provide a versatile entry to 9,10-dihydro-9,10-disilaanthracenes,[S8] the parent DSA has not been reported via this approach.

1.1.1. Notes on byproduct formation

Detection of SiH_4

In an argon-filled glovebox, **DSB** (20 mg, 0.12 mmol) was placed in a glass vial and dissolved in THF-d_8 (0.5 mL). A solution of MeLi in diethoxymethane (3.1 M, 7.6 µL, 0.024 mmol) was added cautiously with stirring at room temperature, resulting in the formation of a colorless, turbid mixture. The mixture was transferred to an NMR tube, which was flame-sealed. In addition to **1^{2H}**, SiH_4 was detected by ^{1}H, ^{29}Si{^{1}H}, and ^{29}Si NMR spectroscopy after the sample had been stored for 14 h (Figure S5–Figure S7); cf. ref[S9] for published chemical shift values of SiH_4.

Detection of hydride-transfer side products

(a) Partial methylation products detected in the vacuum distillate

After evaporation of the reaction mixture of **1^{2H}**, the resulting crude residue was distilled under reduced pressure (100–120 °C/10^{-3} mbar), furnishing a colorless liquid. GC–MS analysis of this liquid revealed a mixture of compounds **I**–**V** (Table S1 and Chart S1). Further characterization by ^{1}H, ^{29}Si{^{1}H}, and ^{29}Si NMR spectroscopy confirmed the presence of mono- and dimethylated silyl groups. Because the sample comprised a mixture of five compounds (**I**–**V**), significant signal overlap precluded an unambiguous assignment of all resonances to individual species (see Figure S8–Figure S10). The observed chemical shift ranges for the Si-bound H and Me substituents are consistent with literature data reported for related partially methylated hydrosilanes.[S1,S10,S11]

Table S1. GC–MS data for compounds **I**–**V** detected in the liquid fraction obtained by vacuum distillation of the crude residue after evaporation of the reaction mixture in the synthesis of **1^{2H}** (vacuum distillation instead of the drying step). Retention times (t_R), *m/z* values (calcd and found for [M]$^{\bullet+}$), and relative peak areas (area%) are given; peak areas are reported as relative values and were not corrected for response factors.

Compound		t_R (min)	*m/z* calcd ([M]$^{\bullet+}$)	*m/z* found ([M]$^{\bullet+}$)	area (%)
**I**	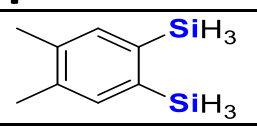	9.19	166.1	166.0	3.7
**II**	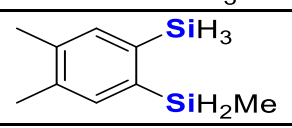	9.84	180.1	180.1	10.5
**III**	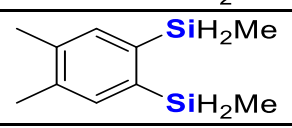	10.38	194.1	194.0	33.7
**IV**	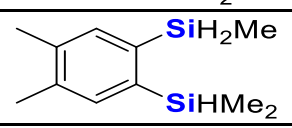	10.68	208.1	208.0	29.0
**V**	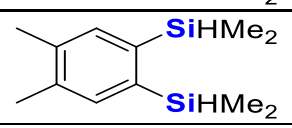	10.89	222.1	222.0	23.1

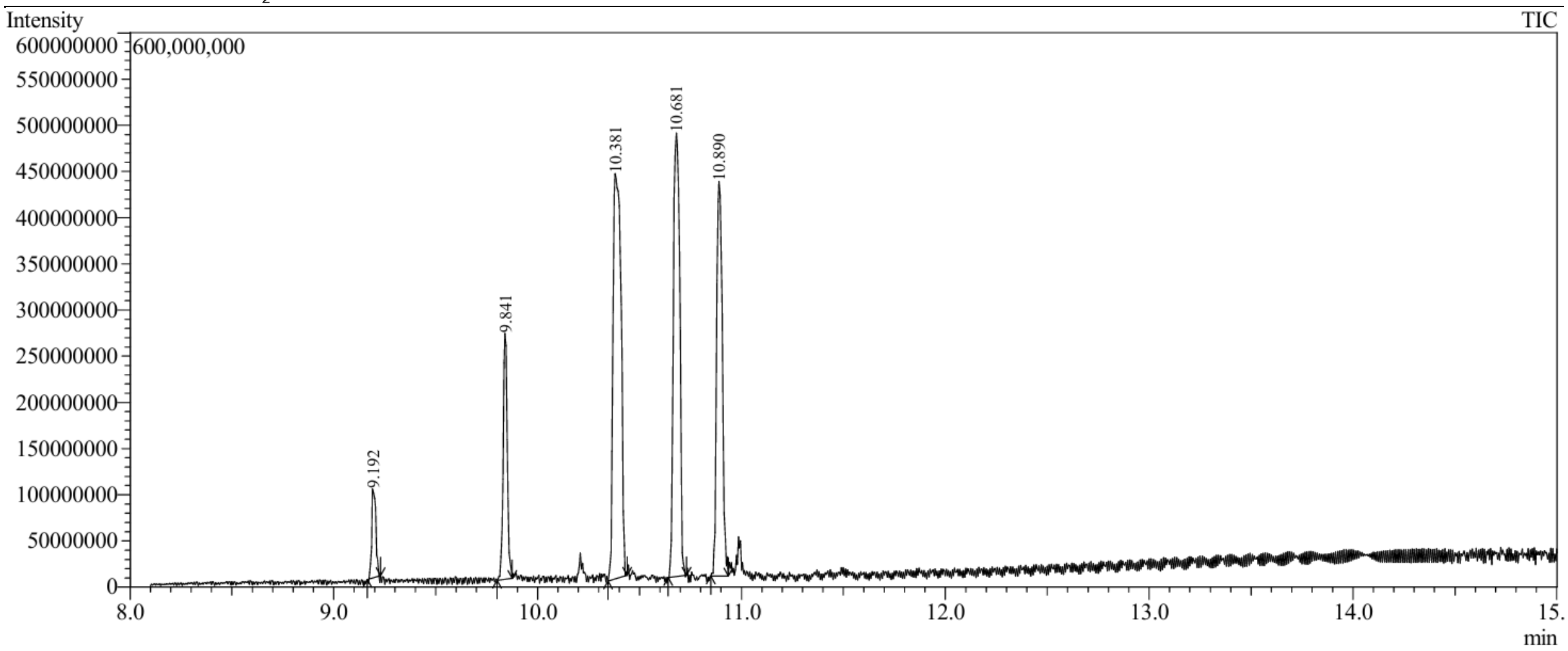

Chart S1. GC–MS chromatogram of the liquid fraction collected by vacuum distillation of the crude residue obtained by evaporation of the reaction mixture in the synthesis of **1^{2H}**. Peaks assignable to compounds **I**–**V** were detected (intensity (a.u.) vs t_R (min)).

The detected partially methylated species likely result from the reaction of **DSB** with MeLi under the applied conditions. Their formation provides indirect evidence for *in situ* LiH formation, which is known to play a key role in hydrosilane redistribution reactions (Scheme S1).[S12]

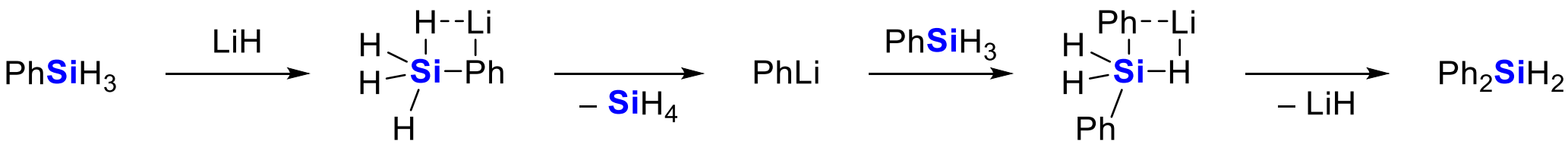

Scheme S1. Postulated mechanism for the LiH-mediated disproportionation of phenylsilane to diphenylsilane.[S12]

(b) Control experiments with deliberately added alkali metal hydrides

Since *in situ*-generated LiH is a plausible active species in the cyclocondensation of **DSB** to $\mathbf{1^{2H}}$, control experiments were conducted, in which **DSB** (10 mg, 0.060 mmol) was treated directly with 1 equiv. of MH (M = Li, K) in THF-d_8 (0.5 mL; flame-sealed NMR tubes). Under these conditions, no conversion to $\mathbf{1^{2H}}$ was observed after 3 d. The absence of reactivity may reflect limited hydride availability, due to poor solubility in THF and/or surface passivation of the metal hydrides (in contrast to the added MH, *in situ*-generated LiH should be extremely finely divided).

As KH, in contrast to LiH, is generally known to promote hydrosilane redistribution reactions, an additional control experiment was conducted under modified conditions. In an argon-filled glovebox, **DSB** (10 mg, 0.060 mmol) was placed in a glass vial and dissolved in THF-d_8 (0.5 mL), and solid KH (1 equiv.) was added. The resulting suspension was stirred at room temperature for 14 h. In contrast to the experiments performed in flame-sealed NMR tubes, complete conversion of **DSB** to $\mathbf{1^{2H}}$ was observed under these conditions, as judged by ^{1}H NMR spectroscopy of the reaction mixture.

(c) Cyclocondensation in the presence of in situ-generated KH

To assess whether the disproportionation could also be promoted by *in situ*-generated KH, KC_8 (2 mg, 0.02 mmol) was added to a solution of **DSB** (11 mg, 0.066 mmol) in THF-d_8 (0.5 mL), as reduction of hydrosilanes is known to afford KH.[S13] ^{1}H NMR analysis of the resulting reaction mixture showed the formation of $\mathbf{1^{2H}}$ as the major product, indicating that the cyclocondensation of **DSB** to $\mathbf{1^{2H}}$ can proceed also in the presence of *in situ*-generated KH (Figure S11).

Detection and isolation of minor side products

Besides **1^2H^**, additional intermediates/species consistent with a stepwise cyclocondensation of **DSB** were detected/isolated.

(a) **DSB** (11 mg, 0.066 mmol) was placed in a glass vial and dissolved in THF-d_8 (0.5 mL). KC_8 (1 mg, 0.007 mmol) was added with stirring. The resulting mixture was transferred to an NMR tube, flame-sealed, and analyzed by ^{1}H NMR spectroscopy. Substantial amounts of starting material remained, while the singly condensed intermediate **Int2** and only minor amounts of **1^{2H}** were detected. Notably, after 1 d, no further conversion was observed, indicating that the reaction stalled at this stage under these conditions. Intermediate **Int2** was identified by both ^{1}H and ^{29}Si{^{1}H} NMR spectroscopy (Figure S12 and Figure S13). Increasing the amount of KC_8 to ≥ 0.2 equiv. resulted in complete consumption of **DSB** and formation of **1^{2H}** as the major product.

(b) **DSB** (10 mg, 0.060 mmol) was placed in a glass vial and dissolved in THF-d_8 (0.5 mL). A solution of MeLi in diethoxymethane (3.1 M, 4.0 µL, 0.012 mmol) was added with stirring. The resulting mixture was transferred to an NMR tube, flame-sealed, and analyzed by ^{1}H NMR spectroscopy. After 30 min, resonances assignable to **Int2** (Scheme S2, middle) were observed alongside unconsumed starting material (Figure S14). After 1 d, the spectrum was dominated by signals corresponding to **1^{2H}** (Figure S15).

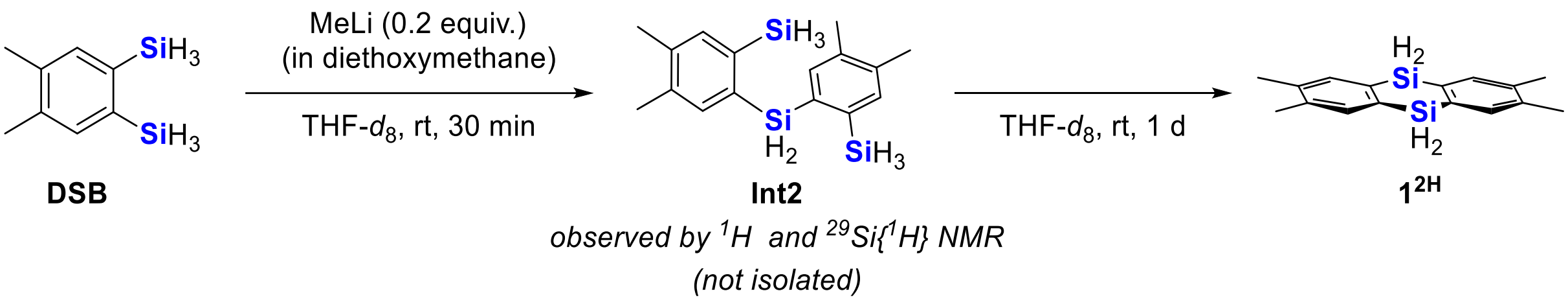

Scheme S2. Stepwise cyclocondensation of **DSB** to **1^{2H}** upon treatment with MeLi (solution in diethoxymethane) in THF-d_8 (flame-sealed NMR tube); intermediate **Int2** was observed after 30 min (not isolated).

On the NMR scale, ^{1}H NMR spectroscopy indicated that the above reaction (b) largely terminates at the stage of **1^{2H}**, as no significant amounts of additional products were observed. However, upon scale-up (Section 1.1.), column chromatography of the sublimation residue obtained during the purification of **1^{2H}** (silica gel, *n*-hexane/$CHCl_3$ 8:1, R_f = 0.54) followed by recrystallization from *n*-hexane afforded **VI** as a colorless crystalline solid (102 mg, 0.253 mmol, 5%). In addition, the disilatriptycene derivative **VII** was isolated in very small, non-quantified (n.q.) amounts and was not obtained in analytically pure form. Its formation indicates that further Si–H/Si–C^{Ar} redistribution processes may occur to a minor extent under these reaction conditions (Scheme S3).

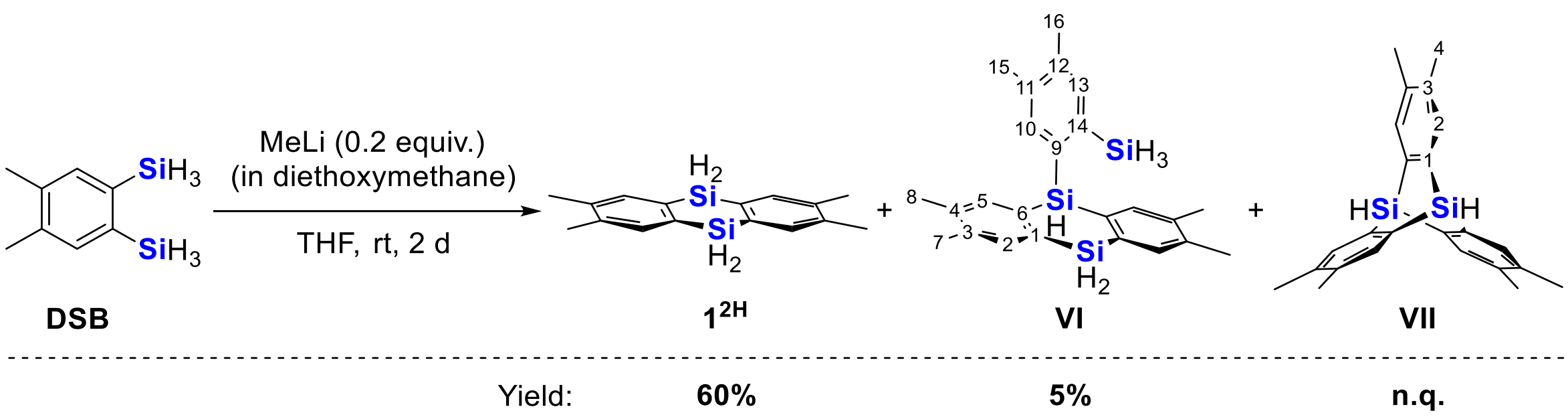

Scheme S3. Isolated products obtained from the reaction of **DSB** with MeLi.

VI:

Single crystals of **VI** suitable for X-ray diffraction were obtained by recrystallization from *n*-hexane.

^{1}H NMR (500.2 MHz, $CDCl_3$): δ = 7.56 (s, 2H, H-2), 7.48 (s, 1H, H-13), 7.42 (s, 1H, H-10), 7.31 (s, 2H, H-5), 5.56 (s, 1H, SiH), 4.96 (d, $^2J_{HH}$ = 6.0 Hz, 1H, SiH_2), 4.91 (d, $^2J_{HH}$ = 6.0 Hz, 1H, SiH_2), 4.11 (s, 3H, SiH_3), 2.30 (s, 6H, H-8), 2.29 (s, 3H, H-15), 2.24 (s, 3H, H-16), 2.21 (s, 6H, H-7).

^{13}C{^{1}H} NMR (125.8 MHz, $CDCl_3$): δ = 139.6 (C-13), 139.6 (C^{Ar}), 138.9 (C-10), 138.2 (C^{Ar}), 138.2 (C^{Ar}), 137.9 (C^{Ar}), 137.9 (C^{Ar}), 137.7 (C^{Ar}), 137.4 (C-5), 136.8 (C-2), 134.7 (C^{Ar}), 133.5 (C^{Ar}), 19.9 (CH_3), 19.9 (CH_3), 19.9 (CH_3), 19.8 (CH_3).

^{29}Si{^{1}H} NMR (99.4 MHz, $CDCl_3$): δ = –33.0 (SiH), –50.8 (SiH_2), –59.3 (SiH_3).

^{29}Si NMR (99.4 MHz, $CDCl_3$): δ = –33.0 (dm, $^1J_{SiH}$ = 195.5 Hz, SiH), –50.8 (tm, $^1J_{SiH}$ = 198.4 Hz, SiH_2), –59.3 (qm, $^1J_{SiH}$ = 200.5 Hz, SiH_3).

HRMS (EI): *m/z* calcd for $C_{24}H_{29}Si_3$ ($[M–H]^{\bullet+}$) 401.1572; found: 401.1580.

EA (%): calcd for $C_{24}H_{30}Si_3$ [402.75]: C 71.57, H 7.51; found: C 70.87, H 7.79.

VII:

Single crystals of **VII** suitable for X-ray diffraction were obtained upon evaporation of the combined eluates (*n*-hexane/$CHCl_3$ 8:1) under ambient atmosphere.

^{1}H NMR (500.2 MHz, CD_2Cl_2): δ = 7.55 (s, 6H, H-2), 5.43 (s, 2H, SiH), 2.21 (s, 18H, H-4).

^{13}C{^{1}H} NMR (125.8 MHz, CD_2Cl_2): δ = 141.6 (C-1), 136.3 (C-3), 134.6 (C-2), 19.8 (C-4).

^{29}Si{^{1}H} NMR (99.4 MHz, CD_2Cl_2): δ = –38.1.

^{29}Si NMR (99.4 MHz, CD_2Cl_2): δ = –38.1 (d, $^1J_{SiH}$ = 217.0 Hz).

HRMS (EI): *m/z* calcd for $C_{24}H_{26}Si_2$ ($[M]^{\bullet+}$) 370.1568; found: 370.1566.

1.2. Synthesis of K_2[**1**]

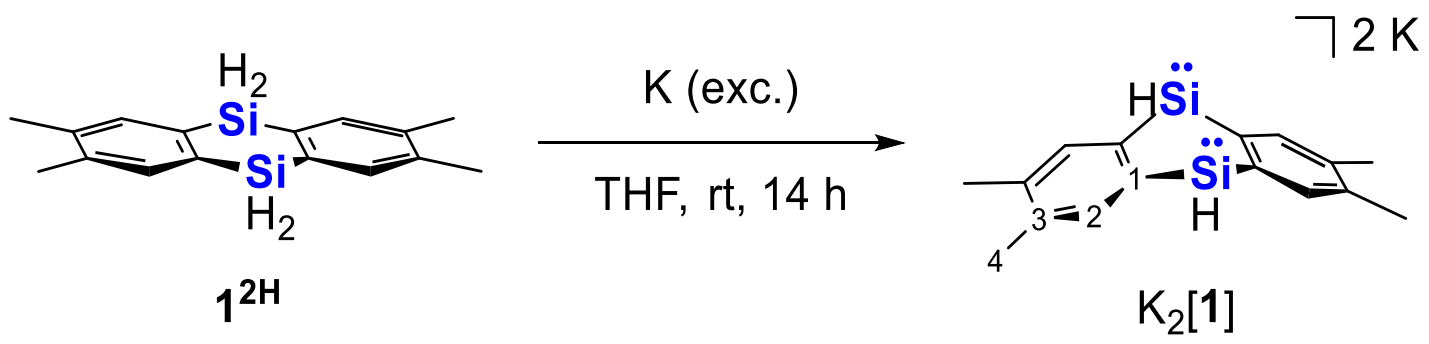

In an argon-filled glovebox, a Schlenk flask was charged with $\mathbf{1^{2H}}$ (100 mg, 0.372 mmol) and K metal (291 mg, 7.44 mmol). THF (10 mL) was added at room temperature with stirring, and the reaction mixture was stirred for 14 h. After filtration of the reaction mixture through a syringe filter, K_2[**1**] was obtained as a red solution in THF and used directly.

Single crystals of K_2[**1**] suitable for X-ray diffraction were grown in an argon-filled glovebox by vapor diffusion of Et_2O into a THF solution of K_2[**1**], followed by partial solvent evaporation.

^{1}H NMR (500.2 MHz, THF-*d*$_8$): δ = 7.07 (s, 4H, H-2), 4.35 (s, 2H, SiH), 2.04 (s, 12H, H-4).

^{13}C{^{1}H} NMR (125.8 MHz, THF-*d*$_8$): δ = 156.5 (C-1), 132.9 (C-2), 128.4 (C-3), 19.7 (C-4).

^{29}Si{^{1}H} NMR (99.4 MHz, THF-*d*$_8$): δ = –48.7.

^{29}Si NMR (99.4 MHz, THF-*d*$_8$): δ = –48.7 (d, $^1J_{SiH}$ = 102.2 Hz).

Notes on alternative access routes to K_2[**1**]

While our standard procedure employs an excess of K metal, replacing the K metal with KC_8 (≥ 4 equiv.) under otherwise identical conditions also afforded K_2[**1**] in near-quantitative conversion.

Synthesis of M_2[**1**] *with alkali-metal counterions M^+ other than K^+*

Preliminary experiments with lighter alkali metals indicated that clean access to M_2[**1**] is not readily achieved for M = Li or Na. Treatment of $\mathbf{1^{2H}}$ (10 mg, 0.037 mmol) with Li metal (6 mg, 0.9 mmol) in THF for 1 d resulted in an unselective reaction, giving rise to multiple species. Although signals tentatively attributable to Li[$\mathbf{1^{H}}$] were observed by ^{1}H NMR spectroscopy, unambiguous assignment was not possible (Figure S28). In contrast, reaction of $\mathbf{1^{2H}}$ with Na metal under analogous conditions predominantly afforded Na[$\mathbf{1^{H}}$], as discussed in Section 1.3. In both cases, prolonged reaction times did not lead to the formation of the corresponding M_2[**1**] (M = Li, Na) species; instead, extended stirring resulted in gradual decomposition, as indicated by the appearance of multiple ill-defined NMR signals.

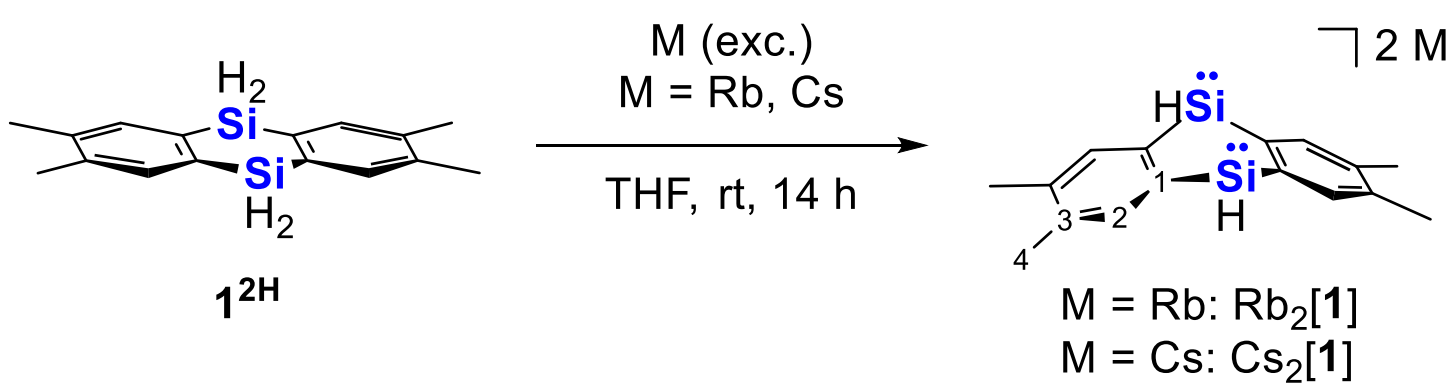

Application of the above-described protocol using the heavier alkali metals Rb or Cs in place of K similarly afforded the corresponding M_2[**1**] (M = Rb, Cs) species: In an argon-filled glovebox, $\mathbf{1^{2H}}$ (10 mg, 0.037 mmol) was placed in a glass vial and dissolved in THF-*d*$_8$ (0.5 mL). Rb (65 mg, 0.76 mmol) or Cs (100 mg, 0.752 mmol) was added, and the respective mixtures were stirred at room temperature for 14 h. The resulting suspensions were filtered through syringe filters to afford THF-*d*$_8$ solutions of Rb_2[**1**] or Cs_2[**1**], respectively.

Rb_2[**1**]:

^{1}H NMR (500.2 MHz, THF-*d*$_8$): δ = 7.13 (s, 4H, H-2), 4.47 (s, 2H, SiH), 2.03 (s, 12H, H-4).

^{13}C{^{1}H} NMR (125.8 MHz, THF-*d*$_8$): δ = 157.1 (C-1), 132.9 (C-2), 128.3 (C-3), 19.7 (C-4).

^{29}Si{^{1}H} NMR (99.4 MHz, THF-*d*$_8$): δ = –44.8.

^{29}Si NMR (99.4 MHz, THF-*d*$_8$): δ = –44.8 (d, $^1J_{SiH}$ = 99.2 Hz, SiH).

Cs_2[**1**]:

^{1}H NMR (500.2 MHz, THF-*d*$_8$): δ = 7.11 (s, 4H, H-2), 4.43 (s, 2H, SiH), 2.04 (s, 12H, H-4).

^{13}C{^{1}H} NMR (125.8 MHz, THF-*d*$_8$): δ = 157.1 (C-1), 133.1 (C-2), 128.5 (C-3), 19.7 (C-4).

^{29}Si{^{1}H} NMR (99.4 MHz, THF-*d*$_8$): δ = –43.9.

^{29}Si NMR (99.4 MHz, THF-*d*$_8$): δ = –43.9 (d, $^1J_{SiH}$ = 98.9 Hz, SiH).

The ^{1}H and ^{13}C{^{1}H} NMR chemical shifts of M_2[**1**] (M = K, Rb, Cs) vary only slightly, whereas the ^{29}Si resonance shows a small downfield shift with increasing cation size (Table S2).

Table S2. Selected ^{1}H, ^{13}C{^{1}H}, and ^{29}Si NMR chemical shifts for M_2[**1**] (M = K, Rb, Cs) in THF-*d*$_8$ (δ in ppm).

**M_2[1]**	***δ*(^{1}H): SiH**	***δ*(^{13}C{^{1}H}): C-1**	***δ*(^{29}Si)**
K_2[**1**]	4.35	156.5	–48.7
Rb_2[**1**]	4.47	157.1	–44.8
Cs_2[**1**]	4.43	157.1	–43.9

Adducts of K_2[**1**] *with **8** or **9***

A THF-d_8 solution of K_2[**1**] (0.037 mmol in 0.5 mL THF-d_8) was prepared according to the protocol described above. At room temperature, this solution was added with stirring to a solution of **8** (7.6 mg, 0.037 mmol) or **9** (8.8 mg, 0.037 mmol) in THF-d_8 (0.1 mL) in a glass vial. The resulting mixture was transferred into an NMR tube, which was flame-sealed.

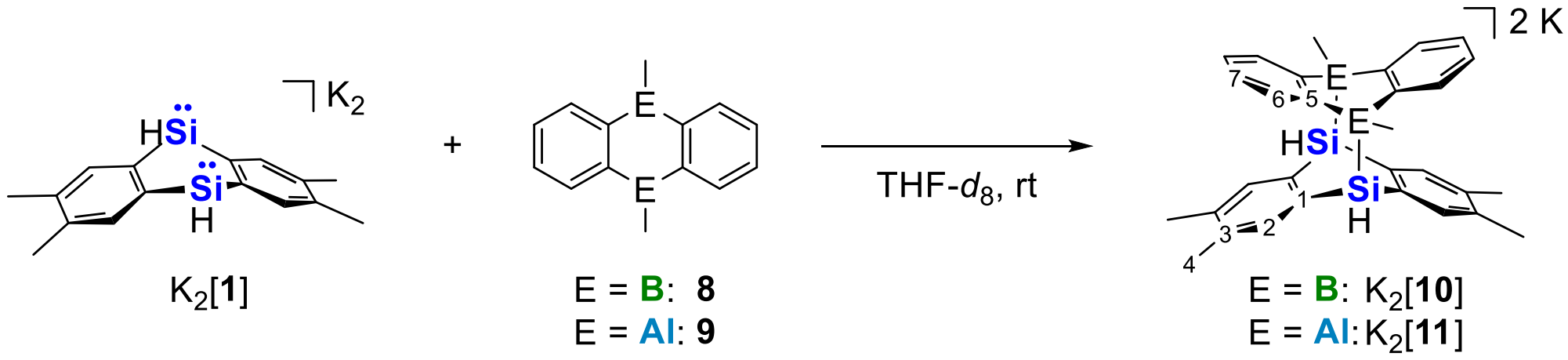

K_2[**10**] (E = B)

Single crystals of K_2[**10**] suitable for X-ray diffraction were grown in an argon-filled glovebox by vapor diffusion of *n*-hexane into a THF-d_8 solution of K_2[**10**], followed by partial solvent evaporation.

Single crystals of [K(18-c-6)(THF)$_2$][K(18-c-6)]$_3$[**10**]$_2$ were grown under an argon atmosphere by carefully layering a 0.1 mL THF solution of 18-c-6 (2.0 equiv. relative to K_2[**10**]) onto a THF-d_8 solution of K_2[**10**]. Subsequent vapor diffusion of *n*-pentane and slow evaporation (without drying to completeness) afforded crystals suitable for X-ray diffraction.

^{1}H NMR (500.2 MHz, THF-*d*$_8$): δ = 7.24–7.21 (m, 4H, H-6), 7.18 (s, 4H, H-2), 6.50–6.47 (m, 4H, H-7), 4.43 (s, 2H, SiH), 2.00 (s, 12H, H-4), 0.57 (s, 6H, BMe).

^{11}B NMR (160.5 MHz, THF-*d*$_8$): δ = –17.3 (s).

^{13}C{^{1}H} NMR (125.8 MHz, THF-*d*$_8$): δ = 170.0 (C-5), 154.5 (C-1), 135.1 (C-2), 131.7 (C-3), 128.0 (C-6), 120.7 (C-7), 19.2 (C-4), 10.8 (BMe).

^{29}Si NMR (99.4 MHz, THF-*d*$_8$): δ = 2.2 ($^1J_{SiH}$ = 155.0 Hz, SiH) ($^1J_{SiH}$ determined from ^{1}H NMR; chemical shift assigned by ^{1}H–^{29}Si HSQC/HMBC experiments).

K_2[**11**] (E = Al)

^{1}H NMR (500.2 MHz, THF-*d*$_8$): δ = 7.54–7.51 (m, 4H, H-6), 7.34 (s, 4H, H-2), 6.71–6.67 (m, 4H, H-7), 4.64 (s, 2H, SiH), 2.03 (s, 12H, H-4), –0.37 (s, 6H, AlMe).

^{13}C{^{1}H} NMR (125.8 MHz, THF-*d*$_8$): δ = 174.7 (C-5), 152.0 (C-1), 135.6 (C-6), 135.3 (C-2), 132.2 (C-3), 123.2 (C-7), 19.1 (C-4), –10.5 (AlMe).

^{29}Si NMR (99.4 MHz, THF-*d*$_8$): δ = –35.3 ($^1J_{SiH}$ = 144.6 Hz, SiH) ($^1J_{SiH}$ determined from ^{1}H NMR; chemical shift assigned by ^{1}H–^{29}Si HSQC/HMBC experiments).

1.3. Synthesis of K[1^H]

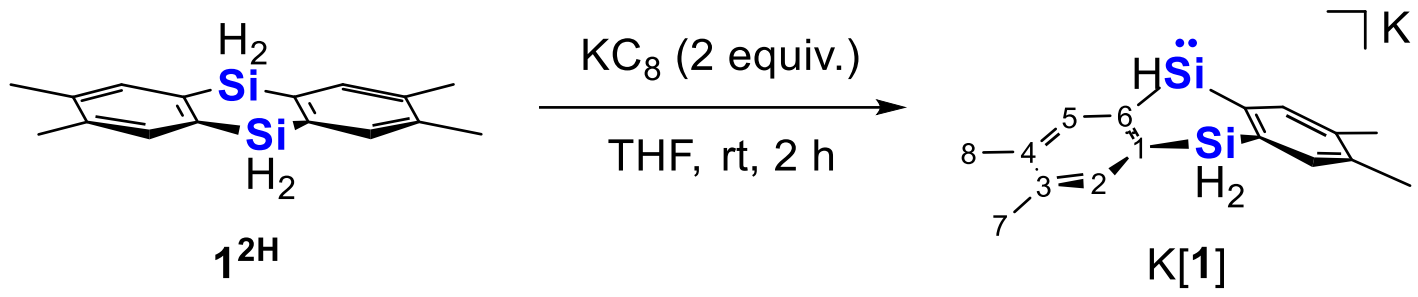

In an argon-filled glovebox, a Schlenk flask was charged with **1^{2H}** (100 mg, 0.372 mmol) and KC_8 (101 mg, 0.747 mmol). THF (10 mL) was added at room temperature, and the reaction mixture was stirred for 2 h. After filtration of the reaction mixture through a syringe filter, K[1^H] was obtained as an orange solution in THF and used directly.

^{1}H NMR (500.2 MHz, THF-d_8): δ = 7.32 (s, 2H, H-2), 7.17 (s, 2H, H-5), 4.73 (d, $^2J_{HH}$ = 6.6 Hz, 1H, SiH_2), 4.59 (s, 1H, SiH), 4.25 (d, $^2J_{HH}$ = 6.6 Hz, 1H, SiH_2), 2.12 (s, 6H, H-7), 2.10 (s, 6H, H-8).

^{13}C{^{1}H} NMR (125.8 MHz, THF-d_8): δ = 167.8 (C-6), 135.4 (C-2), 133.9 (C-5), 133.6 (C-4), 130.0 (C-1), 128.9 (C-3), 19.8 (C-7), 19.3 (C-8).

^{29}Si{^{1}H} NMR (99.4 MHz, THF-d_8): δ = –40.8 (SiH), –49.8 (SiH_2).

^{29}Si NMR (99.4 MHz, THF-d_8): δ = –40.8 (d, $^1J_{SiH}$ = 102.7 Hz, SiH), –49.8 (tm, $^1J_{SiH}$ = 181.4 Hz, SiH_2).

Notes on alternative access routes to [1^H]$^-$*:*

(a) Treatment of **1^{2H}** (10 mg, 0.037 mmol) with an excess of Na metal (17 mg, 0.74 mmol) in THF-d_8 (0.5 mL) and stirring for 1 d resulted predominantly, but not exclusively, in the formation of Na[1^H], which was identified as the major species by ^{1}H NMR spectroscopy (Figure S50a). Prolonged stirring did not improve the selectivity of the reaction; instead, gradual decomposition, accompanied by the formation of multiple ill-defined species, was observed.

(b) **1^{2H}** (10 mg, 0.037 mmol) was dissolved in C_6D_6 (0.5 mL) and treated with an excess of K metal (30 mg, 0.77 mmol). After stirring for 1 d, the reaction mixture was filtered through a syringe filter. ^{1}H NMR spectroscopic analysis of the C_6D_6 filtrate revealed the presence of unconsumed **1^{2H}**. The filter cake was extracted with THF-d_8 (0.5 mL), and NMR spectroscopic examination of the extract confirmed the formation of K[1^H] (Figure S50b).

(c) The feasibility of selectively forming K[1^H] via formal H^+-transfer from **1^{2H}** to K_2[**1**] was investigated. **1^{2H}** (10 mg, 0.037 mmol) was dissolved in THF-d_8 (0.5 mL), treated with an excess of K metal (30 mg, 0.77 mmol), and stirred for 1 d. After filtration, NMR spectroscopy of the filtrate confirmed the clean formation of K_2[**1**] (see below). Subsequent addition of **1^{2H}** (10 mg, 0.037 mmol) to the freshly prepared THF-d_8 solution of K_2[**1**] led to the formation of K[1^H] as the major species according to ^{1}H NMR spectroscopy, albeit with lower selectivity than observed for the KC_8-based approach (Figure S50c).

Overall, the KC_8-based protocol (Figure S50d) provided the fastest, cleanest, and most complete conversion to K[1^H] and was therefore employed in subsequent experiments.

1.4. Reactivity of K_2[**1**] toward mono- and difunctional organochlorosilanes

1.4.1. Synthesis of *cis*-**2**

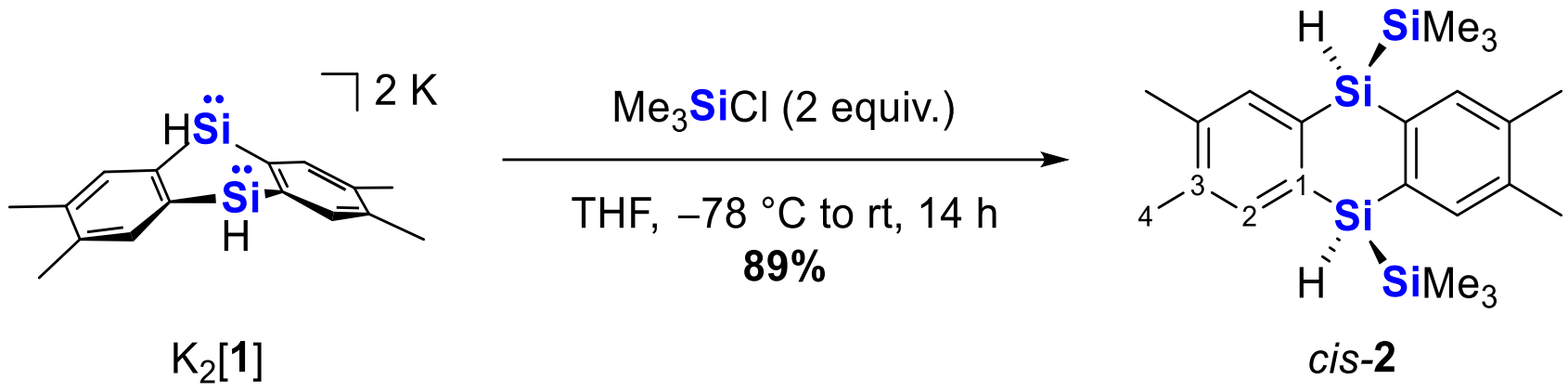

A THF solution of K_2[**1**] (0.19 mmol in 5 mL THF) was prepared according to the protocol described in Section 1.2. At –78 °C, a solution of Me_3SiCl (47 µL, 0.37 mmol) in THF (5 mL) was added dropwise via a dropping funnel. The reaction mixture was stirred for 14 h while warming to room temperature. Volatile components were removed under reduced pressure, and the residue was extracted into *n*-hexane (4 × 5 mL). Removal of the solvent from the combined extracts under dynamic vacuum afforded *cis*-**2** as a colorless solid. Yield: 70 mg (0.17 mmol, 89%).

Single crystals of *cis*-**2** suitable for X-ray diffraction were grown in an argon-filled glovebox by slow evaporation of a solution of *cis*-**2** in *n*-hexane.

^{1}H NMR (500.2 MHz, $CDCl_3$): δ = 7.34 (s, 4H, H-2), 4.85 (s, 2H, SiH), 2.29 (s, 12H, H-4), 0.13 (s, 18H, $SiMe_3$).

^{13}C{^{1}H} NMR (125.8 MHz, $CDCl_3$): δ = 136.7 (C-2), 136.5 (C-1/3), 136.4 (C-1/3), 19.9 (C-4), –1.0 ($SiMe_3$).

^{29}Si{^{1}H} NMR (99.4 MHz, $CDCl_3$): δ = –17.9 ($SiMe_3$), –46.7 (SiH).

^{29}Si NMR (99.4 MHz, $CDCl_3$): δ = –17.9 (m, $SiMe_3$), –46.7 (d, $^{1}J_{SiH}$ = 180.6 Hz, SiH).

HRMS (EI): *m/z* calcd for $C_{22}H_{35}Si_4$ ([M–H]$^{\bullet+}$) 411.1816; found: 411.1759.

EA (%): calcd for $C_{22}H_{36}Si_4$ [412.86]: C 64.00, H 8.79; found: C 63.67, H 8.84.

1.4.2. Synthesis of *cis*-**3**

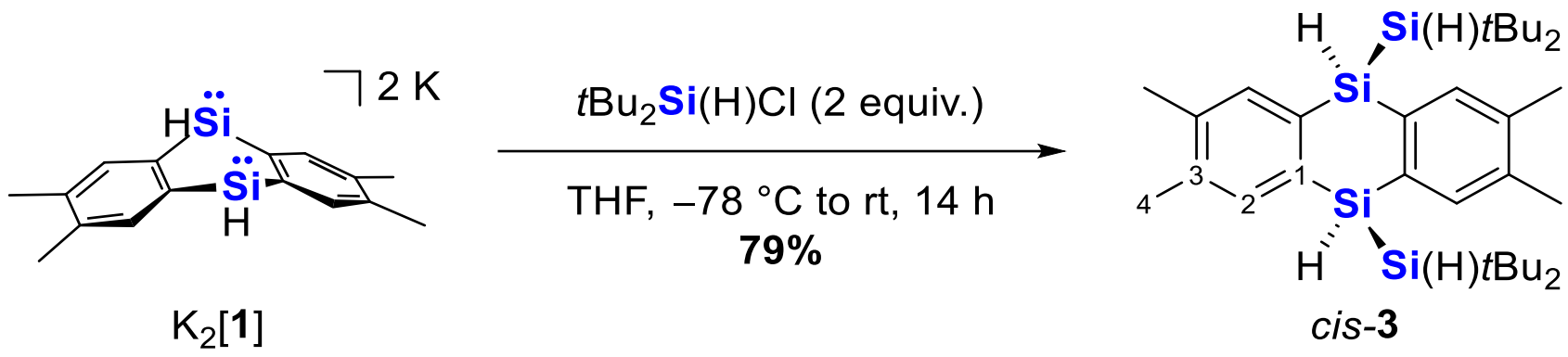

A THF solution of K_2[**1**] (0.19 mmol in 5 mL THF) was prepared according to the protocol described in Section 1.2. At –78 °C, a THF solution of tBu$_2$Si(H)Cl (75 µL, 0.37 mmol in 5 mL THF) was added dropwise via a dropping funnel. The reaction mixture was stirred for 14 h while warming to room temperature. Volatile components were removed under reduced pressure, and the residue was extracted into *n*-hexane (4 × 5 mL). Removal of the solvent from the combined extracts under dynamic vacuum afforded a colorless residue. *Cis*-**3** was isolated after column chromatography (silica gel, *n*-hexane/$CHCl_3$ 8:1, R_f = 0.75) as a colorless solid. Yield: 81 mg (0.15 mmol, 79%).

Single crystals of *cis*-**3** suitable for X-ray diffraction were grown by slow evaporation of a solution of *cis*-**3** in *n*-hexane under ambient atmosphere.

^{1}H NMR (500.2 MHz, $CDCl_3$): δ = 7.54 (s, 4H, H-2), 5.07 (s, 2H, SiH), 3.79 (s, 2H, SitBu$_2$*H*), 2.27 (s, 12H, H-4), 1.10 (s, 36H, *t*Bu).

^{13}C{^{1}H} NMR (125.8 MHz, $CDCl_3$): δ = 137.8 (C-2), 137.8 (C-1), 136.5 (C-3), 30.7 (C(*C*H$_3$)$_3$), 20.9 (*C*(CH$_3$)$_3$), 19.9 (C-4).

^{29}Si{^{1}H} NMR (99.4 MHz, $CDCl_3$): δ = 1.3 (SitBu$_2$H), –50.5 (SiH).

^{29}Si NMR (99.4 MHz, $CDCl_3$): δ = 1.3 (dm, $^{1}J_{SiH}$ = 173.0 Hz, SitBu$_2$H), –50.5 (dm, $^{1}J_{SiH}$ = 181.8 Hz, SiH).

HRMS (EI): *m/z* calcd for $C_{28}H_{47}Si_4$ ([M–*t*Bu]$^{\bullet+}$) 495.2749; found: 495.2756.

1.4.3. Synthesis of **4**

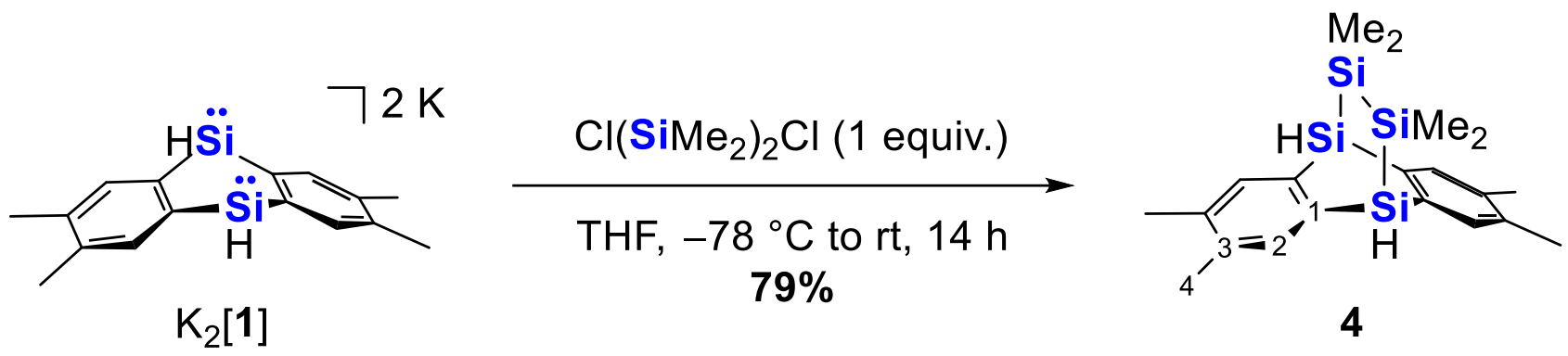

A THF solution of K_2[**1**] (0.19 mmol in 5 mL THF) was prepared according to the protocol described in Section 1.2. At –78 °C, a solution of $Cl(SiMe_2)_2Cl$ (35 µL, 0.19 mmol) in THF (5 mL) was added dropwise via a dropping funnel. The reaction mixture was stirred for 14 h while warming to room temperature. Volatile components were removed under reduced pressure, and the residue was extracted into *n*-hexane (4 × 5 mL). Removal of the solvent from the combined extracts under dynamic vacuum afforded a colorless residue. **4** was isolated after column chromatography (silica gel, *n*-hexane/$CHCl_3$ 8:1, R_f = 0.48) as a colorless solid. Yield: 58 mg (0.15 mmol, 79%).

Single crystals of **4** suitable for X-ray diffraction were grown by slow evaporation of a solution of **4** in *n*-hexane under ambient atmosphere.

^{1}H NMR (500.2 MHz, $CDCl_3$): δ = 7.43 (s, 4H, H-2), 4.90 (s, 2H, SiH), 2.23 (s, 12H, H-4), 0.03 (s, 12H, $SiMe_2$).

^{13}C{^{1}H} NMR (125.8 MHz, $CDCl_3$): δ = 139.3 (C-1), 136.2 (C-3), 135.4 (C-2), 19.8 (C-4), –6.6 ($SiMe_2$).

^{29}Si{^{1}H} NMR (99.4 MHz, $CDCl_3$): δ = –41.7 (SiH), –59.4 ($SiMe_2$).

HRMS (EI): *m/z* calcd for $C_{20}H_{30}Si_4$ ($[M]^{\bullet+}$) 382.1419; found: 382.1407.

1.4.4. Synthesis of **5**

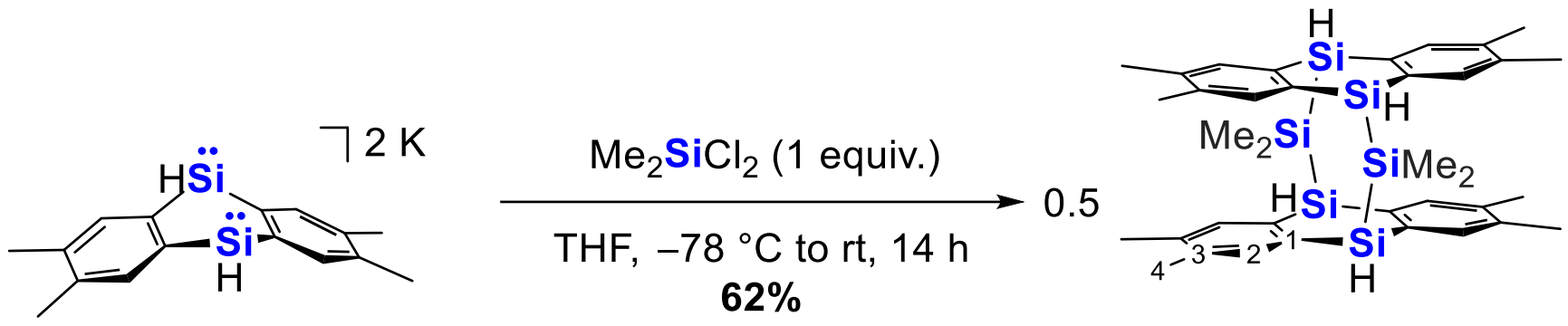

A THF solution of K_2[**1**] (0.19 mmol in 5 mL THF) was prepared according to the protocol described in Section 1.2. At –78 °C, a solution of Me_2SiCl_2 (23 µL, 0.19 mmol) in THF (5 mL) was added dropwise via a dropping funnel. The reaction mixture was stirred for 14 h while warming to room temperature. Volatile components were removed under reduced pressure. The residue was extracted into *n*-hexane (4 × 10 mL). The combined extracts were concentrated under dynamic vacuum to afford **5** as a colorless solid. Yield: 38 mg (0.059 mmol, 62%).

Single crystals of **5** suitable for X-ray diffraction were grown in an argon-filled glovebox by slow evaporation of a solution of **5** in $CHCl_3$.

^{1}H NMR (500.2 MHz, $CDCl_3$): δ = 7.03 (s, 8H, H-2), 4.81 (s, 4H, SiH), 2.13 (s, 24H, H-4), 0.66 (s, 12H, $SiMe_2$).

^{13}C{^{1}H} NMR (125.8 MHz, $CDCl_3$): δ = 136.5 (C-2), 135.9 (C-1), 135.7 (C-3), 19.8 (C-4), –3.4 ($SiMe_2$).

^{29}Si{^{1}H} NMR (99.4 MHz, $CDCl_3$): δ = –46.4 (SiH), –49.8 ($SiMe_2$).

HRMS (EI): *m/z* calcd for $C_{36}H_{48}Si_6$ ($[M]^{\bullet+}$) 648.2372; found: 648.2380.

1.5. Reactivity of K[**1^H^**] toward mono- and difunctional organochlorosilanes

1.5.1. Synthesis of **6**

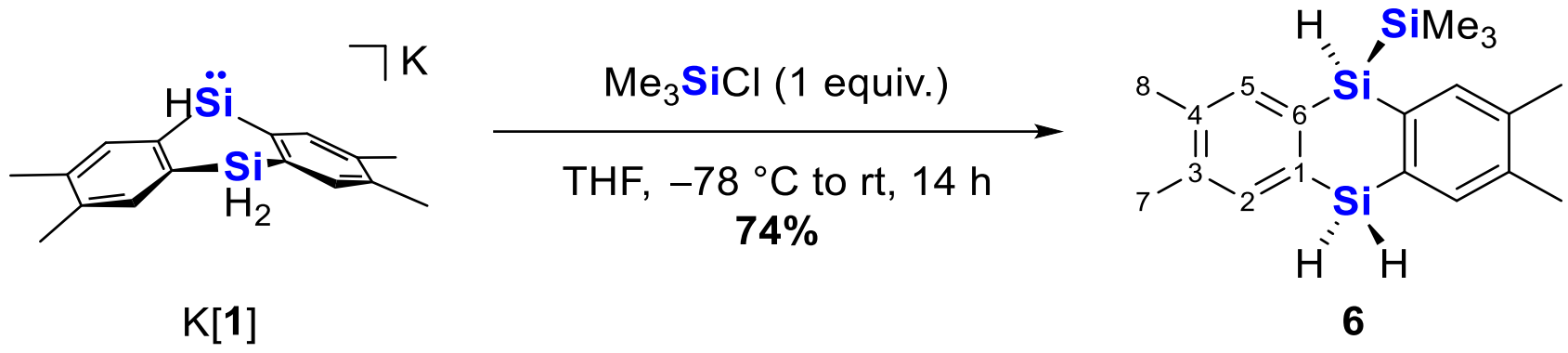

A THF solution of K[**1^{H}**] (0.19 mmol in 5 mL THF) was prepared according to the protocol described in Section 1.3. At –78 °C, a solution of Me_3SiCl (24 µL, 0.19 mmol) in THF (5 mL) was added dropwise via a dropping funnel. The reaction mixture was stirred for 14 h while warming to room temperature. Volatile components were removed under reduced pressure, and the residue was extracted into *n*-hexane (4 × 5 mL). The combined extracts were concentrated under reduced pressure, and the resulting residue was sublimed at 120 °C/10^{-3} mbar to afford **6** as a colorless solid. Yield: 48 mg (0.14 mmol, 74%).

Single crystals of **6** suitable for X-ray diffraction were grown in an argon-filled glovebox by slow evaporation of a solution of **6** in *n*-hexane.

^{1}H NMR (500.2 MHz, $CDCl_3$): δ = 7.51 (s, 2H, H-2), 7.39 (s, 2H, H-5), 4.83 (d, $^{2}J_{HH}$ = 6.2 Hz, 1H, SiH_2), 4.79 (d, $^{2}J_{HH}$ = 6.2 Hz, 1H, SiH_2), 4.77 (s, 1H, SiH), 2.29 (s, 12H, H-7 and H-8), 0.06 (s, 9H, $SiMe_3$).

^{13}C{^{1}H} NMR (125.8 MHz, $CDCl_3$): δ = 138.2 (C-6), 137.4 (C-3), 136.9 (C-2), 136.7 (C-4), 136.4 (C-5), 134.1 (C-1), 20.0 (C-8), 19.8 (C-7), –1.6 ($SiMe_3$).

^{29}Si{^{1}H} NMR (99.4 MHz, $CDCl_3$): δ = –16.5 ($SiMe_3$), –43.9 (SiH), –48.8 (SiH_2).

^{29}Si NMR (99.4 MHz, $CDCl_3$): δ = –16.5 (m, $SiMe_3$), –43.9 (d, $^{1}J_{SiH}$ = 182.3 Hz, SiH), –48.8 (tm, $^{1}J_{SiH}$ = 196.8 Hz, SiH_2).

HRMS (EI): *m/z* calcd for $C_{19}H_{27}Si_3$ ($[M–H]^{\bullet+}$) 339.1415; found: 339.1423.

EA (%): calcd for $C_{19}H_{28}Si_3$ [340.68]: C 66.98, H 8.28; found: C 65.45, H 8.24.

1.5.2. Synthesis of **7**

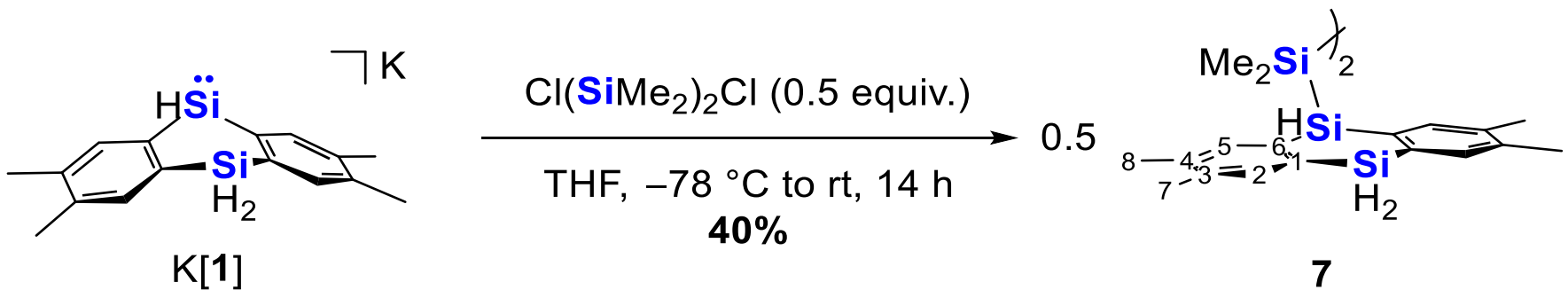

A THF solution of K[**1**[H]] (0.19 mmol in 5 mL THF) was prepared according to the protocol described in Section 1.3. At –78 °C, a solution of $Cl(SiMe_2)_2Cl$ (17 µL, 0.092 mmol) in THF (5 mL) was added dropwise via a dropping funnel. The reaction mixture was stirred for 14 h while warming to room temperature. Volatile components were removed under reduced pressure. The residue was washed with *n*-hexane (4 × 5 mL) and subsequently extracted into CH_2Cl_2 (4 × 5 mL). The combined extracts were concentrated under dynamic vacuum to afford **7** as a colorless solid. Yield: 25 mg (0.038 mmol, 40%).

Single crystals of **7** suitable for X-ray diffraction were grown in an argon-filled glovebox by slow evaporation of a solution of **7** in CH_2Cl_2.

^{1}H NMR (500.2 MHz, CD_2Cl_2): δ = 7.50 (s, 4H, H-2), 7.34 (s, 4H, H-5), 4.89 (s, 2H, SiH), 4.80 (d, $^2J_{HH}$ = 6.1 Hz, 2H, SiH_2), 4.76 (d, $^2J_{HH}$ = 6.1 Hz, 2H, SiH_2), 2.27 (s, 12H, H-8), 2.26 (s, 12H, H-7), –0.09 (s, 12H, $SiMe_2$).

^{13}C{^{1}H} NMR (125.8 MHz, CD_2Cl_2): δ = 138.5 (C-6), 137.9 (C-3), 137.2 (C-4), 137.1 (C-2), 136.8 (C-5), 134.2 (C-1), 19.8 (C-7/8), 19.8 (C-7/8), –5.5 ($SiMe_2$).

^{29}Si NMR (99.4 MHz, CD_2Cl_2): δ = –41.5 ($SiMe_2$), –42.4 (SiH), –49.2 (SiH_2) (assigned by ^{1}H–^{29}Si HSQC/HMBC experiments).

HRMS (EI): *m/z* calcd for $C_{36}H_{49}Si_6$ ($[M–H]^{•+}$) 649.2450; found: 649.2391.

EA (%): calcd for $C_{36}H_{50}Si_6$ [651.30]: C 66.39, H 7.74; found: C 65.68, H 7.92.

1.6. Synthesis of **13**

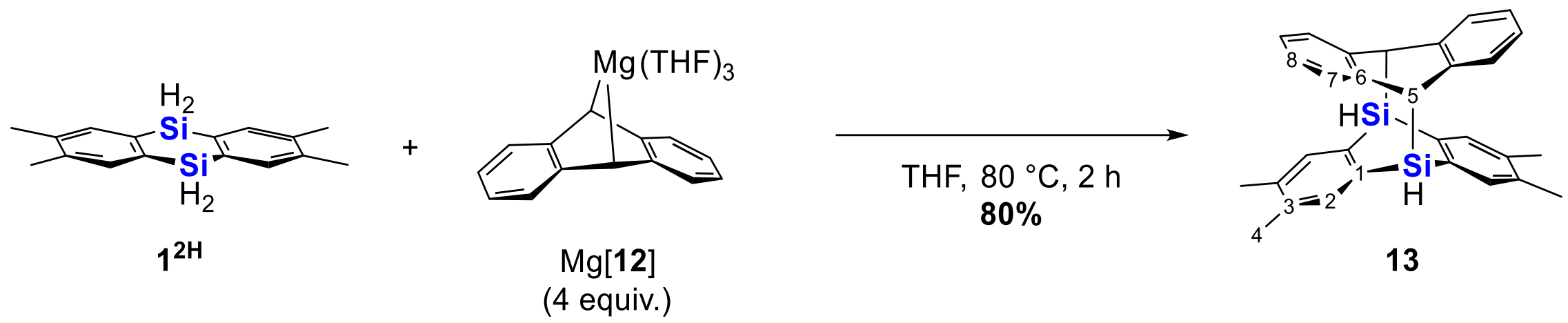

In an argon-filled glovebox, an ampoule was charged with Mg[**12**] (562 mg, 1.34 mmol), **1^{2H}** (90 mg, 0.34 mmol), and THF (5 mL). The ampoule was flame-sealed and heated at 80 °C for 2 h. After cooling to room temperature, the ampoule was opened and the reaction mixture was transferred to a flask. Volatiles were removed under a dynamic vacuum, and the residue was extracted into *n*-hexane (4 × 10 mL). The combined extracts were concentrated under reduced pressure and the residue purified by column chromatography (silica gel, *n*-hexane/$CHCl_3$ = 8:1, R_f = 0.31) to afford **13** as a colorless solid. Yield: 121 mg (0.272 mmol, 80%).

Single crystals of **13** suitable for X-ray diffraction were grown by slow evaporation of a solution of **13** in *n*-hexane under ambient atmosphere.

^{1}H NMR (500.2 MHz, THF-d_8): δ = 7.13 (s, 4H, H-2), 6.91–6.87 (m, 4H, H-7), 6.69–6.65 (m, 4H, H-8), 5.19 (d, $^{3}J_{HH}$ = 5.1 Hz, 2H, SiH), 4.25 (d, $^{3}J_{HH}$ = 5.1 Hz, 2H, H-5), 2.06 (s, 12H, H-4).

^{13}C{^{1}H} NMR (125.8 MHz, THF-d_8): δ = 140.1 (C-1), 139.6 (C-6), 136.4 (C-3), 135.8 (C-2), 126.2 (C-7), 125.0 (C-8), 40.9 (C-5), 19.4 (C-4).

^{29}Si{^{1}H} NMR (99.4 MHz, THF-d_8): δ = –11.3.

^{29}Si NMR (99.4 MHz, THF-d_8): δ = –11.3 (dm, $^{1}J_{SiH}$ = 203.4 Hz).

HRMS (EI): *m/z* calcd for $C_{30}H_{28}Si_2$ ($[M]^{\bullet+}$) 444.1724; found: 444.1727.

1.7. Reactivity of K[$\mathbf{1}^{\mathbf{H}}$] toward diaryl alkynes

The reactivity of K[$\mathbf{1}^{\mathbf{H}}$] toward diaryl alkynes was investigated using 1,2-bis(4-methoxyphenyl)ethyne (AnsC≡CAns) and 1,2-di-*p*-tolylethyne (*p*TolC≡C*p*Tol).

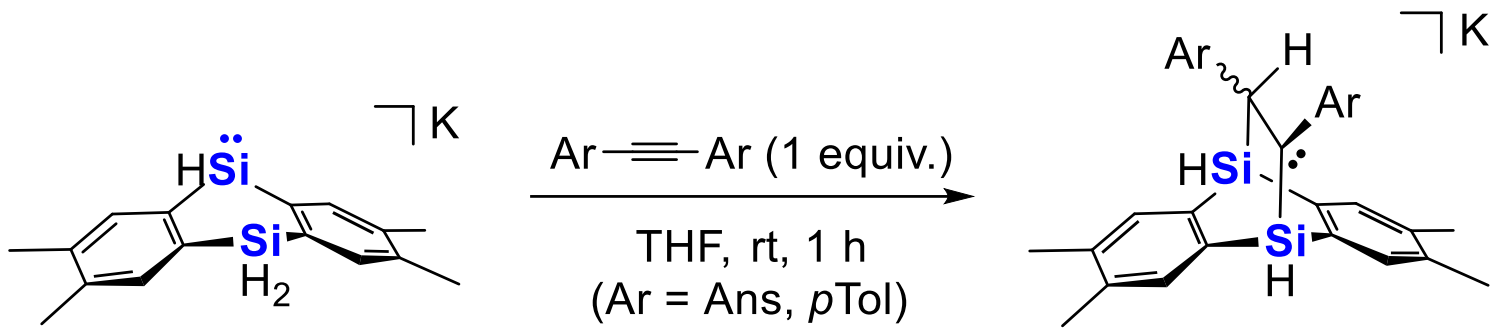

K[**1**]   Ar = Ans: K[$\mathbf{15}^{\mathbf{Ans}}$]
Ar = *p*Tol: K[**15**]

A THF solution of K[$\mathbf{1}^{\mathbf{H}}$] (0.19 mmol in 5 mL THF) was prepared according to the protocol described in Section 1.3. At room temperature, this solution was added dropwise with stirring to a THF solution (5 mL) of the respective diaryl alkyne (AnsC≡CAns: 44 mg, 0.18 mmol; *p*TolC≡C*p*Tol: 38 mg, 0.18 mmol), resulting in an immediate color change of the reaction mixture from orange to deep red. Stirring was continued for 1 h, affording THF solutions of K[$\mathbf{15}^{\mathbf{Ans}}$] (Ar = Ans) and K[**15**] (Ar = *p*Tol), respectively, which were used directly in subsequent reactions.

K[$\mathbf{15}^{\mathbf{Ans}}$]:

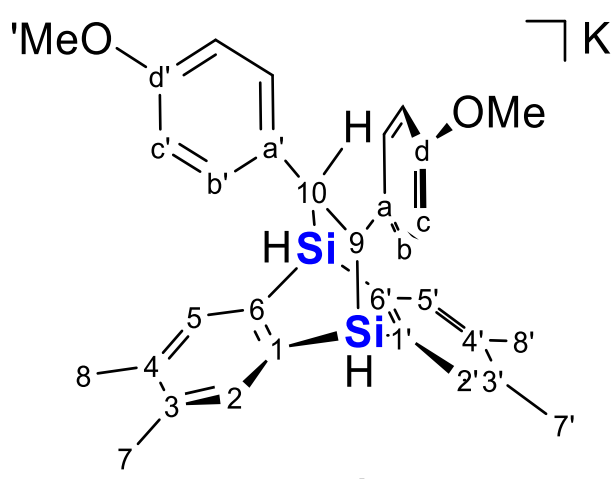

K[$\mathbf{15}^{\mathbf{Ans}}$]

^{1}H NMR (500.2 MHz, THF-*d*$_8$): δ = 7.30 (s, 1H, H-5/5'), 7.26 (s, 1H, H-2/2'), 7.18 (s, 1H, H-2/2'), 6.98 (s, 1H, H-5/5'), 6.89 (d, $^3J_{HH}$ = 8.6 Hz, 2H, H-b'), 6.47 (d, $^3J_{HH}$ = 8.6 Hz, 2H, H-c'), 6.12 (br, 2H, H-b/c)*, 5.71 (s, 1H, C^{-}SiH), 4.72 (s, 1H, HCSi*H*), 3.61 (s, 3H, OMe), 3.40 (s, 3H, OMe'), 2.66 (s, 1H, H-10), 2.18 (s, 3H, H-7/7'/8/8'), 2.15 (s, 6H, H-7/7'/8/8'), 2.06 (s, 3H, H-7/7'/8/8').

*Note 1: The measured integral value of this signal is too small to account for all four C*H* protons of this Ans ring. We assume a partial double bond between the carbanionic C-9 and C-a, resulting in hindered rotation and concomitant motional broadening. Either the resonance at 6.12 ppm is so severely broadened that a reliable integral cannot be obtained, or the remaining two C*H* protons give rise to a second signal (cf. the situation encountered for the *p*Tol derivative K[**15**]) that is broadened beyond detection.

^{13}C{^{1}H} NMR (125.8 MHz, THF-*d*$_8$): δ = 157.0 (C-d'), 151.3 (C-a), 150.8 (C-1/1'), 150.0 (C-1/1'), 143.9 (C-d), 142.8 (C-a'), 142.7 (C-6/6'), 142.0 (C-6/6'), 135.1 (C-3/3'), 134.7 (C-5/5'), 134.6 (C-3/3'), 133.9 (C-5/5'), 133.2 (C-4/4'), 132.6 (C-4/4'), 131.5 (C-2/2'), 130.5 (C-2/2'), 128.7 (C-b'), 117.3 (C-b/c), 113.3 (C-c'), 57.2 (OMe'), 54.8 (OMe), 52.1 (C-9), 36.2 (C-10), 19.7 (C-7/7'/8/8'), 19.7 (C-7/7'/8/8'), 19.5 (C-7/7'/8/8'), 19.4 (C-7/7'/8/8'); n.o. C-b/c (cf. Note 1 above).

^{29}Si{^{1}H} NMR (99.4 MHz, THF-*d*$_8$): δ = –25.5 (HCSiH)*, –44.2 (C^{-}SiH).

*Note 2: This signal was assigned based on the ^{29}Si{^{1}H} NMR spectrum of the protonation product $\mathbf{16}^{\mathbf{Ans}}$.

^{29}Si NMR (99.4 MHz, THF-*d*$_8$): δ = –25.5 (dm, $^1J_{SiH}$ = 195.8 Hz, HCSiH), –44.2 (dm, $^1J_{SiH}$ = 188.2 Hz, C^{-}SiH).

K[**15**]:

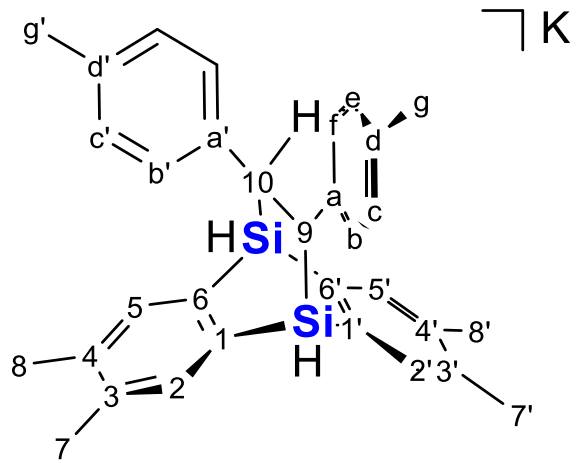

K[**15**]

Single crystals of K[**15**] suitable for X-ray diffraction were grown in an argon-filled glovebox by vapor diffusion of Et_2O into a THF solution of K[**15**], followed by partial solvent evaporation.

^{1}H NMR (500.2 MHz, THF-*d*$_8$): δ = 7.30 (s, 1H, H-5/5’), 7.25 (s, 1H, H-2/2’), 7.18 (s, 1H, H-2/2’), 6.97 (s, 1H, H-5/5’), 6.88 (d, $^3J_{HH}$ = 8.0 Hz, 2H, H-b’), 6.83 (br, 1H, H-b/c/e/f), 6.69 (d, $^3J_{HH}$ = 8.0 Hz, 2H, H-c’), 6.24 (br, 1H, H-b/c/e/f), 6.09 (br, 1H, H-b/c/e/f), 5.70 (s, 1H, C^-SiH), 5.61 (br, 1H, H-b/c/e/f), 4.72 (s, 1H, HCSi*H*), 2.76 (s, 1H, H-10), 2.17 (s, 3H, H-7/7’/8/8’), 2.15 (s, 6H, H-7/7’/8/8’), 2.14 (s, 3H, H-g’), 2.06 (s, 3H, H-7/7’/8/8’), 1.83 (s, 3H, H-g).

^{13}C{^{1}H} NMR (125.8 MHz, THF-*d*$_8$): δ = 153.1 (C-a), 150.6 (C-1/1’), 149.7 (C-1/1’), 148.4 (C-a’), 142.6 (C-6/6’), 141.9 (C-6/6’), 135.1 (C-3/3’), 134.7 (C-5/5’), 134.6 (C-3/3’), 133.9 (C-5/5’), 133.3 (C-4/4’), 132.8 (C-4/4’), 132.2 (C-d’), 131.6 (C-2/2’), 130.7 (C-2/2’), 130.5 (C-b/c/e/f), 129.2 (C-b/c/e/f), 128.6 (C-c’), 128.1 (C-b’), 117.7 (C-b/c/e/f), 110.9 (C-b/c/e/f), 109.9 (C-d), 55.2 (C-9), 36.5 (C-10), 20.9 (C-g’), 20.3 (C-g), 19.7 (C-7/7’/8/8’), 19.7 (C-7/7’/8/8’), 19.5 (C-7/7’/8/8’), 19.4 (C-7/7’/8/8’).

^{29}Si{^{1}H} NMR (99.4 MHz, THF-*d*$_8$): δ = –25.0 (HCSiH)*, –44.2 (C^-SiH).

*Note 3: This signal was assigned based on the ^{29}Si{^{1}H} NMR spectrum of the protonation product **16**.

^{29}Si NMR (99.4 MHz, THF-*d*$_8$): δ = –25.0 (dm, $^1J_{SiH}$ = 203.2 Hz, HCSiH), –44.2 (dm, $^1J_{SiH}$ = 190.4 Hz, C^-SiH).

*Protonation of K[**15^{Ans}**] and K[**15**] with HNTf$_2$ (NMR scale)*

A THF-*d*$_8$ solution of K[**1^H**] (0.037 mmol in 0.5 mL THF-*d*$_8$) was prepared according to the protocol described in Section 1.3. At room temperature, this solution was added with stirring to the respective diaryl alkyne ArC≡CAr (Ar = Ans: 9 mg, 0.04 mmol; Ar = *p*Tol: 8 mg, 0.04 mmol) in a glass vial. ^{1}H NMR spectroscopic analysis of the resulting reaction mixtures in sealed NMR tubes for each alkyne indicated formation of K[**15^{Ans}**] and K[**15**], respectively, as the major species, accompanied in each case by minor resonances of a second species (Figure S85a (Ar = Ans) and Figure S86a (Ar = *p*Tol)). Upon addition of $HNTf_2$ (10 mg, 0.036 mmol) to the respective THF-*d*$_8$ solution with stirring, immediate decolorization was observed. Subsequent ^{1}H NMR spectroscopy showed complete conversion of K[**15^{Ans}**] and K[**15**] into the second species (Figure S85b (Ar = Ans) and Figure S86b (Ar = *p*Tol)). Based on the observed spectroscopic changes upon addition of $HNTf_2$, **16a^{Ans}** and **16a** are assigned as the protonated forms of K[**15^{Ans}**] and K[**15**], respectively.

1.7.1. Protonation of K[**15**] with $HNTf_2$ (preparative scale)

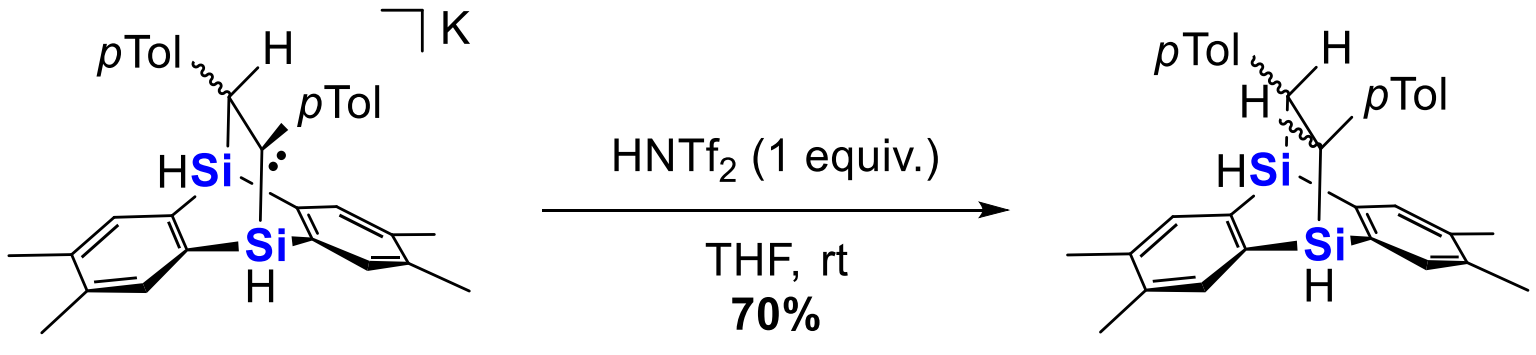

A THF solution of K[**15**] (0.372 mmol in 10 mL THF) was prepared according to the protocol described above. At room temperature, this solution was treated with $HNTf_2$ (104 mg, 0.369 mmol) in one portion with stirring. Immediate decolorization of the reaction mixture was observed. After stirring for 1 min at room temperature, volatile components were removed under reduced pressure, and the residue was extracted into *n*-hexane (4 × 10 mL). The combined extracts were concentrated under reduced pressure, and the crude product was purified by column chromatography (silica gel, *n*-hexane/$CHCl_3$ 2:1). Yield: 123 mg (0.259 mmol, 70%).

In addition to purification, column chromatography allowed the separation of the individual diastereomers, **16a** and **16b**, in small amounts for NMR characterization (R_f(**16a**) = 0.62; R_f(**16b**) = 0.57).

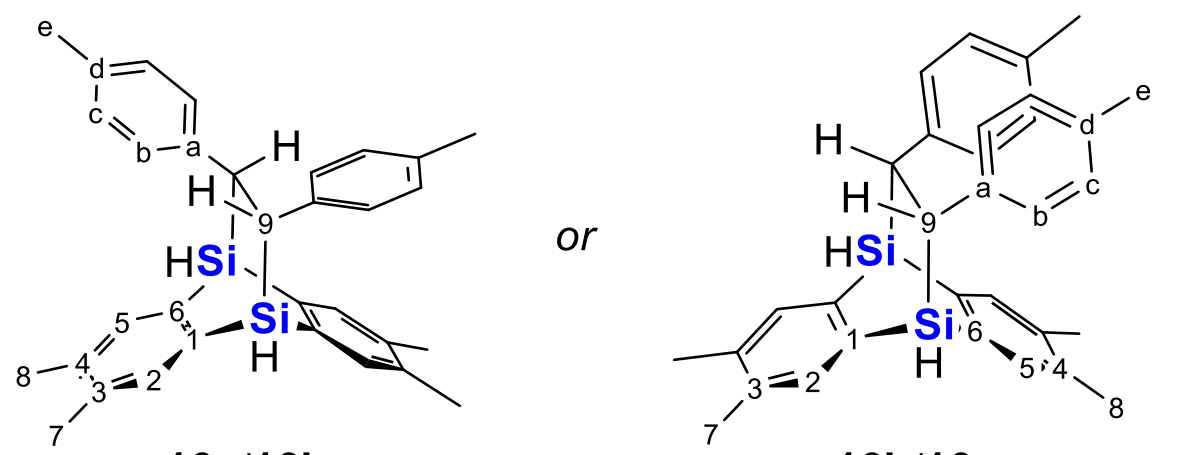

16a/16b *or* 16b/16a

16a:

^{1}H NMR (500.2 MHz, CDCl$_3$): δ = 7.57 (s, 2H, H-2/5), 7.49 (s, 2H, H-2/5), 6.92 (d, $^{3}J_{HH}$ = 8.0 Hz, 4H, H-c), 6.64 (d, $^{3}J_{HH}$ = 8.0 Hz, 4H, H-b), 4.89 (s, 2H, SiH), 2.48 (s, 2H, H-9), 2.31 (s, 6H, H-7/8), 2.28 (s, 6H, H-7/8), 2.25 (s, 6H, H-e).

^{13}C{^{1}H} NMR (125.8 MHz, CDCl$_3$): δ = 142.5 (C-1/6), 142.3 (C-a), 138.7 (C-1/6), 137.0 (C-3/4), 137.0 (C-3/4), 135.5 (C-2/5), 134.3 (C-d), 133.8 (C-2/5), 129.1 (C-c), 127.6 (C-b), 36.9 (C-9), 21.1 (C-e), 20.0 (C-7/8), 19.9 (C-7/8).

^{29}Si{^{1}H} NMR (99.4 MHz, CDCl$_3$): δ = –28.9.

^{29}Si NMR (99.4 MHz, CDCl$_3$): δ = –28.9 (d, $^{1}J_{SiH}$ = 221.7 Hz).

HRMS (EI): *m/z* calcd for $C_{32}H_{34}Si_2$ ($[M]^{\bullet+}$) 474.2194; found: 474.2199.

EA (%): calcd for $C_{32}H_{34}Si_2$ [474.79]: C 80.95, H 7.22; found: C 79.77, H 7.12.

16b:

^{1}H NMR (500.2 MHz, CDCl$_3$): δ = 7.55 (s, 2H, H-2/5), 7.54 (s, 2H, H-2/5), 6.67 (d, $^{3}J_{HH}$ = 8.0 Hz, 4H, H-c), 6.50 (d, $^{3}J_{HH}$ = 8.0 Hz, 4H, H-b), 5.02 (s, 2H, SiH), 3.19 (s, 2H, H-9), 2.29 (s, 6H, H-7/8), 2.27 (s, 6H, H-7/8), 2.09 (s, 6H, H-e).

^{13}C{^{1}H} NMR (125.8 MHz, CDCl$_3$): δ = 142.1 (C-1/6), 140.9 (C-1/6), 138.0 (C-a), 137.1 (C-3/4), 136.8 (C-3/4), 135.3 (C-2/5), 134.2 (C-2/5), 133.9 (C-d), 130.5 (C-b), 128.1 (C-c), 30.8 (C-9), 21.0 (C-e), 20.0 (C-7/8), 19.9 (C-7/8).

^{29}Si{^{1}H} NMR (99.4 MHz, CDCl$_3$): δ = –31.4.

^{29}Si NMR (99.4 MHz, CDCl$_3$): δ = –31.4 (d, $^{1}J_{SiH}$ = 219.3 Hz).

HRMS (EI): *m/z* calcd for $C_{32}H_{34}Si_2$ ($[M]^{\bullet+}$) 474.2194; found: 474.2199.

EA (%): calcd for $C_{32}H_{34}Si_2$ [474.79]: C 80.95, H 7.22; found: C 79.43, H 7.01.

1.7.2. Trapping of K[**15**^{Ans}] with Me_3SiCl (preparative scale)

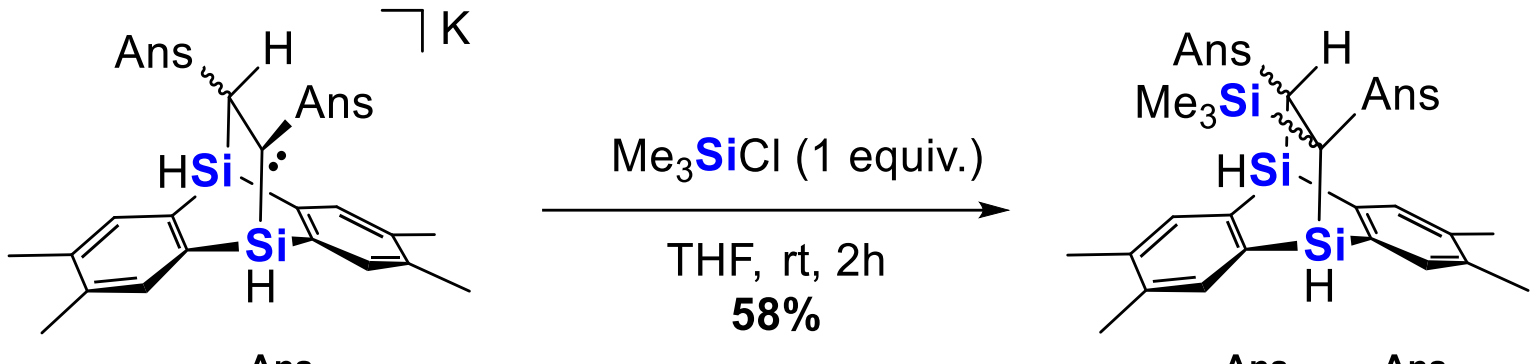

A THF solution of K[**15**^{Ans}] (0.19 mmol in 5 mL THF) was prepared according to the protocol described above. At room temperature, a solution of Me_3SiCl (24 µL, 21 mg, 0.19 mmol) in THF (5 mL) was added dropwise and the reaction mixture was stirred at room temperature for 2 h, during which time it gradually decolorized. Volatile components were removed under reduced pressure, and the residue was extracted into *n*-hexane (4 × 5 mL). The combined extracts were concentrated under reduced pressure, and the crude product was purified by column chromatography (silica gel, *n*-hexane/$CHCl_3$ 8:1). Yield: 64 mg (0.11 mmol, 58%).

In addition to purification, column chromatography allowed the separation of the individual diastereomers, **16a**^{Ans} and **16b**^{Ans}, in small amounts for NMR characterization (R_f(**16a**^{Ans}) = 0.44; R_f(**16b**^{Ans}) = 0.38).

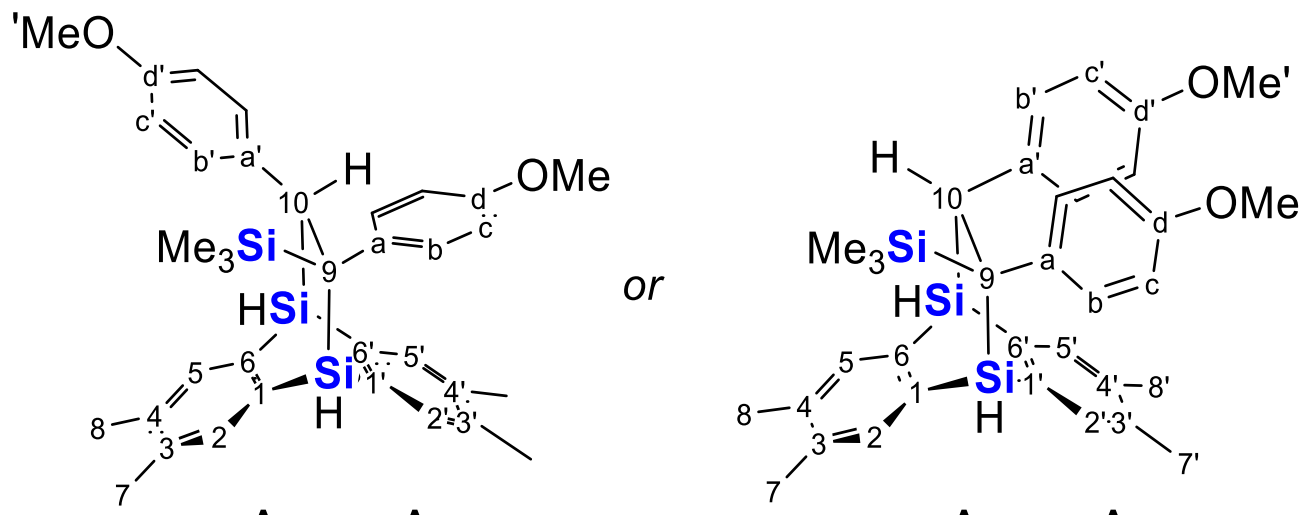

16a^{Ans}:

^{1}H NMR (500.2 MHz, $CDCl_3$): δ = 7.63 (s, 1H, H-5/5′), 7.51 (s, 1H, H-2/2′/5/5′), 7.49 (s, 1H, H-2/2′/5/5′), 7.39 (d, $^3J_{HH}$ = 8.8 Hz, 2H, H-b), 7.29 (s, 1H, H-2/2′), 7.02 (d, $^3J_{HH}$ = 8.7 Hz, 2H, H-b′), 6.76 (d, $^3J_{HH}$ = 8.7 Hz, 2H, H-c′), 6.71 (d, $^3J_{HH}$ = 8.8 Hz, 2H, H-c), 5.51 (s, 1H, HCSi*H*), 4.70 (s, 1H, SiCSiH), 3.78 (s, 3H, OMe′), 3.73 (s, 3H, OMe), 3.09 (s, 1H, H-10), 2.30 (s, 3H, H-7/7′/8/8′), 2.28 (s, 3H, H-7/7′/8/8′), 2.17 (s, 3H, H-7/7′/8/8′), 2.12 (s, 3H, H-7/7′/8/8′), –0.50 (s, 9H, $SiMe_3$).

^{13}C{^{1}H} NMR (125.8 MHz, $CDCl_3$): δ = 157.9 (C-d′), 156.1 (C-d), 143.0 (C-a), 142.2 (C-1/1′/6/6′), 142.1 (C-1/1′/6/6′), 141.9 (C-1/1′/6/6′), 140.7 (C-1/1′/6/6′), 137.2 (C-3/3′/4/4′), 136.8 (C-3/3′/4/4′), 136.8 (C-a′), 136.4 (C-3/3′/4/4′), 136.1 (C-3/3′/4/4′), 135.9 (C-5/5′), 135.3 (C-2/2′/5/5′), 134.5 (C-2/2′/5/5′), 133.7 (C-2/2′), 131.1 (C-b′), 130.9 (C-b), 113.9 (C-c′), 112.9 (C-c), 55.3 (OMe/OMe′), 55.2 (OMe/OMe′), 39.7 (C-10), 30.4 (C-9), 20.0 (C-7/7′/8/8′), 20.0 (C-7/7′/8/8′), 19.9 (C-7/7′/8/8′), 19.8 (C-7/7′/8/8′), 1.2 ($SiMe_3$).

^{29}Si{^{1}H} NMR (99.4 MHz, $CDCl_3$): δ = 2.6 ($SiMe_3$), –31.7 (HCSi*H*), –31.9 (SiC*Si*H).

^{29}Si NMR (99.4 MHz, $CDCl_3$): δ = 2.7–2.5 (m, $SiMe_3$), –31.7 (dm, HCSi*H*), –31.9 (dm, SiC*Si*H).

HRMS (EI): *m/z* calcd for $C_{35}H_{42}O_2Si_3$ ($[M]^{\bullet+}$) 578.2487; found: 578.2474.

16b^{Ans}:

^{1}H NMR (500.2 MHz, $CDCl_3$): δ = 7.62 (s, 1H, H-5/5'), 7.56 (s, 1H, H-5/5'), 7.48 (s, 1H, H-2/2'), 7.16 (s, 1H, H-2/2'), 6.74 (d, $^{3}J_{HH}$ = 8.9 Hz, 2H, H-b), 6.46 (d, $^{3}J_{HH}$ = 8.9 Hz, 2H, H-c'), 6.44 (d, $^{3}J_{HH}$ = 8.9 Hz, 2H, H-c), 6.05 (d, $^{3}J_{HH}$ = 8.9 Hz, 2H, H-b'), 5.57 (s, 1H, HCSi*H*), 4.69 (s, 1H, SiCSiH), 3.69 (s, 3H, OMe'), 3.66 (s, 3H, OMe), 3.17 (s, 1H, H-10), 2.27 (s, 3H, H-7/7'/8/8'), 2.26 (s, 3H, H-7/7'/8/8'), 2.25 (s, 3H, H-7/7'/8/8'), 2.11 (s, 3H, H-7/7'/8/8'), –0.32 (s, 9H, $SiMe_3$).

^{13}C{^{1}H} NMR (125.8 MHz, $CDCl_3$): δ = 156.9 (C-d'), 155.9 (C-d), 142.6 (C-1/1'/6/6'), 141.9 (C-1/1'/6/6'), 141.2 (C-1/1'/6/6'), 141.2 (C-1/1'/6/6'), 137.1 (C-3/3'/4/4'), 136.8 (C-3/3'/4/4'), 136.6 (C-3/3'/4/4'), 136.4 (C-3/3'/4/4'), 135.6 (C-2/2'), 135.2 (C-5/5'), 135.0 (C-5/5'), 134.4 (C-a), 133.8 (C-2/2'), 133.7 (C-b), 133.1 (C-a'), 131.3 (C-b'), 112.8 (C-c), 111.5 (C-c'), 55.2 (OMe/OMe'), 55.0 (OMe/OMe'), 31.8 (C-10), 26.7 (C-9), 20.0 (C-7/7'/8/8'), 19.9 (C-7/7'/8/8'), 19.9 (C-7/7'/8/8'), 19.7 (C-7/7'/8/8'), –0.8 ($SiMe_3$).

^{29}Si{^{1}H} NMR (99.4 MHz, $CDCl_3$): δ = 7.1 ($SiMe_3$), –29.9 (SiC*Si*H), –33.5 (HCSiH).

^{29}Si NMR (99.4 MHz, $CDCl_3$): δ = 7.3–7.0 (m, $SiMe_3$), –29.9 (dm, $^{1}J_{SiH}$ = 216.9 Hz, SiC*Si*H), –33.5 (dm, $^{1}J_{SiH}$ = 208.0 Hz, HCSiH).

HRMS (EI) *m/z*: calcd. for $[C_{35}H_{42}O_2Si_3]^{\bullet+}$ ($[M]^{\bullet+}$): 578.2487; found: 578.2478.

2. Plots of NMR spectra

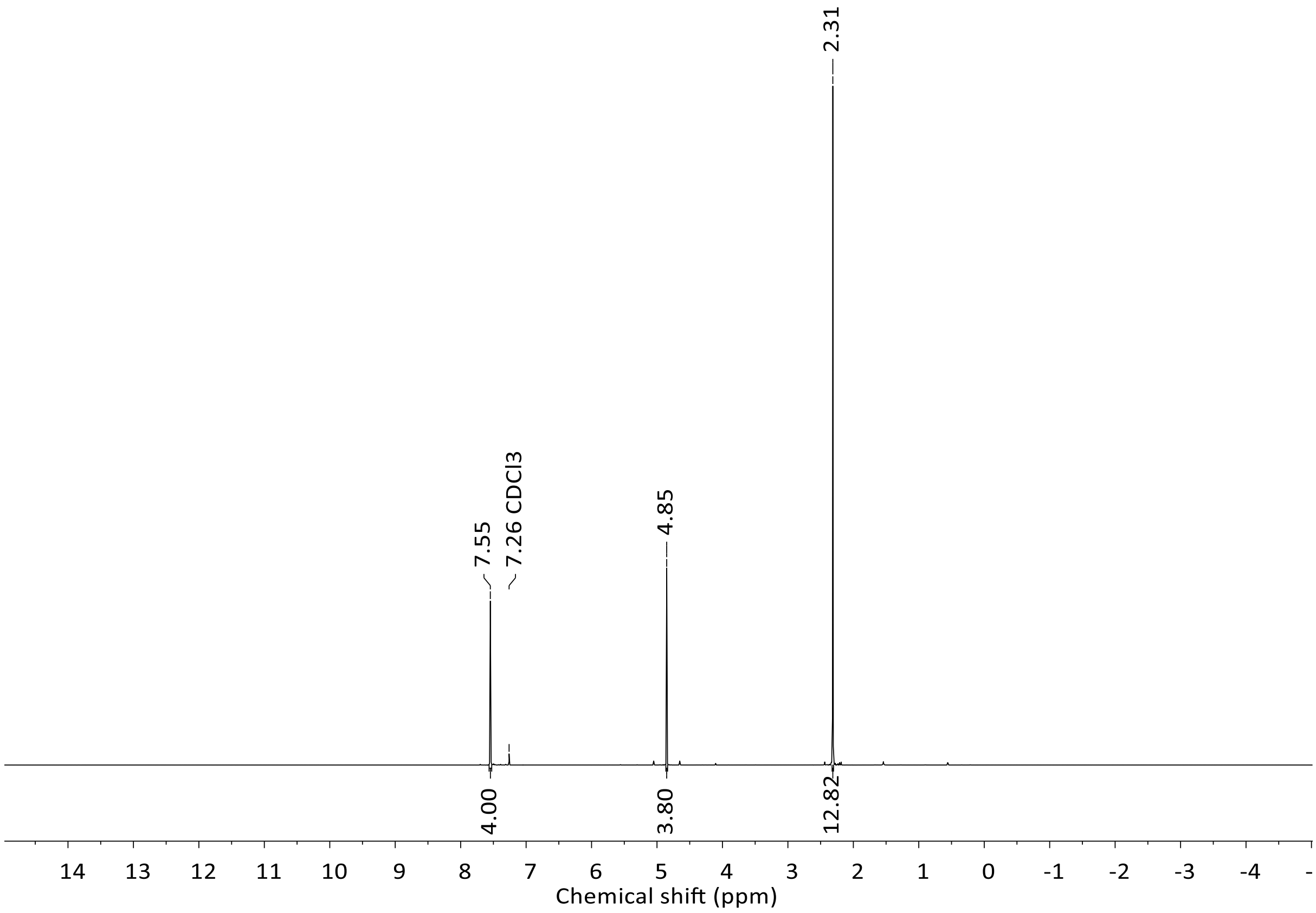

Figure S1. ^{1}H NMR spectrum (500.2 MHz, $CDCl_3$) of **1^{2H}**.

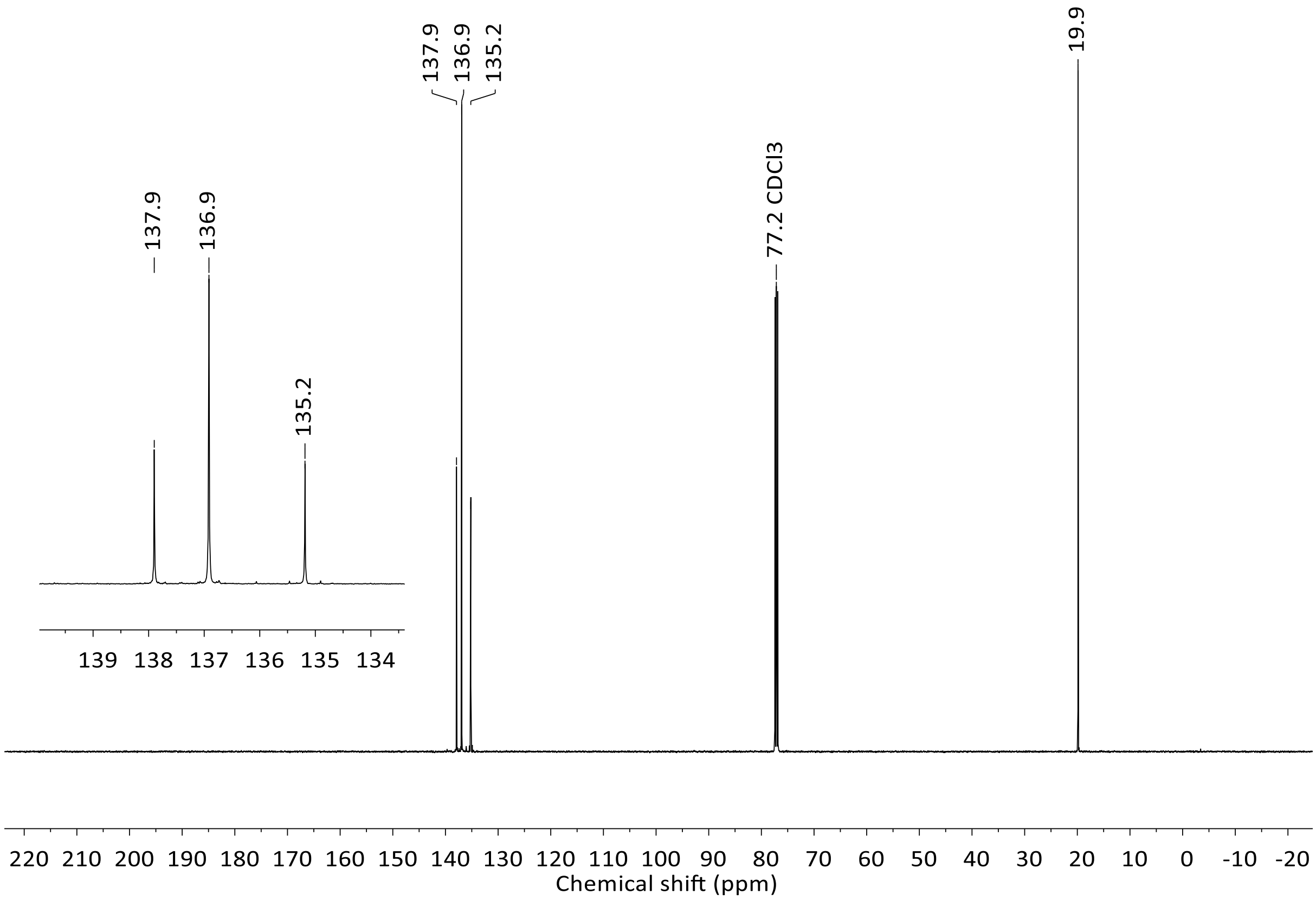

Figure S2. $^{13}C\{^{1}H\}$ NMR spectrum (125.8 MHz, $CDCl_3$) of **1^{2H}**.

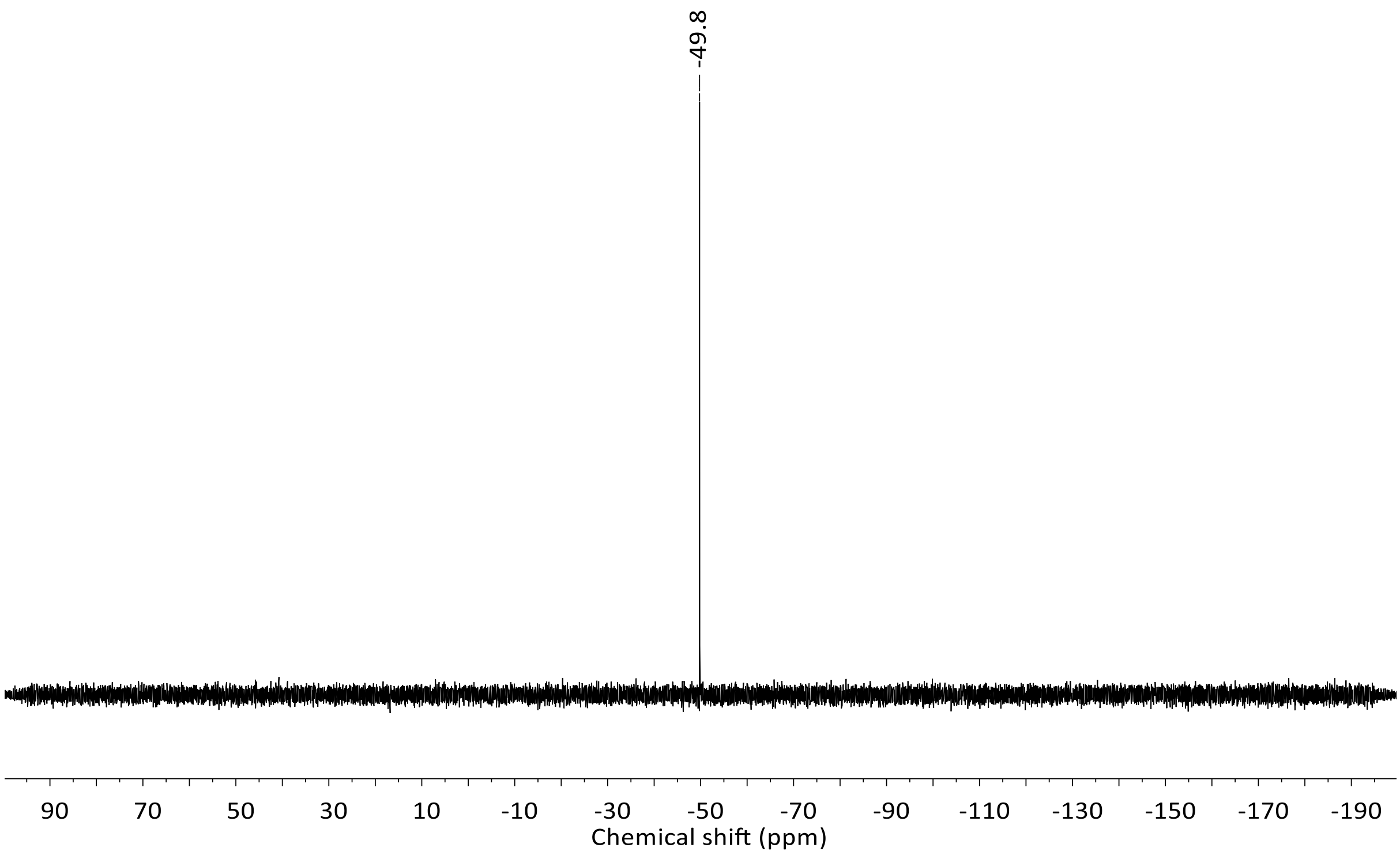

Figure S3. $^{29}Si\{^{1}H\}$ NMR spectrum (99.4 MHz, $CDCl_3$) of **1^{2H}**.

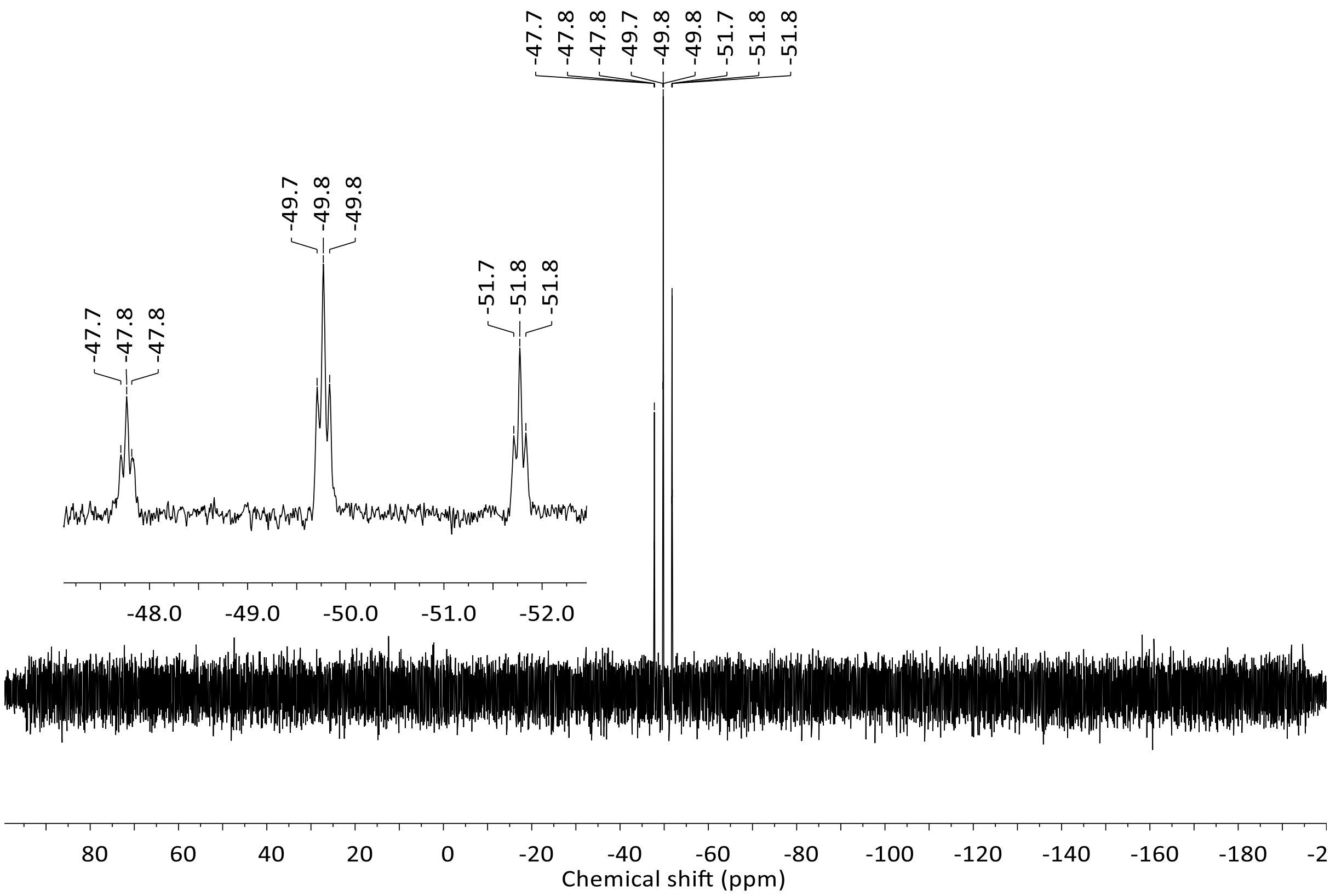

Figure S4. ^{29}Si NMR spectrum (99.4 MHz, $CDCl_3$) of **1^{2H}**.

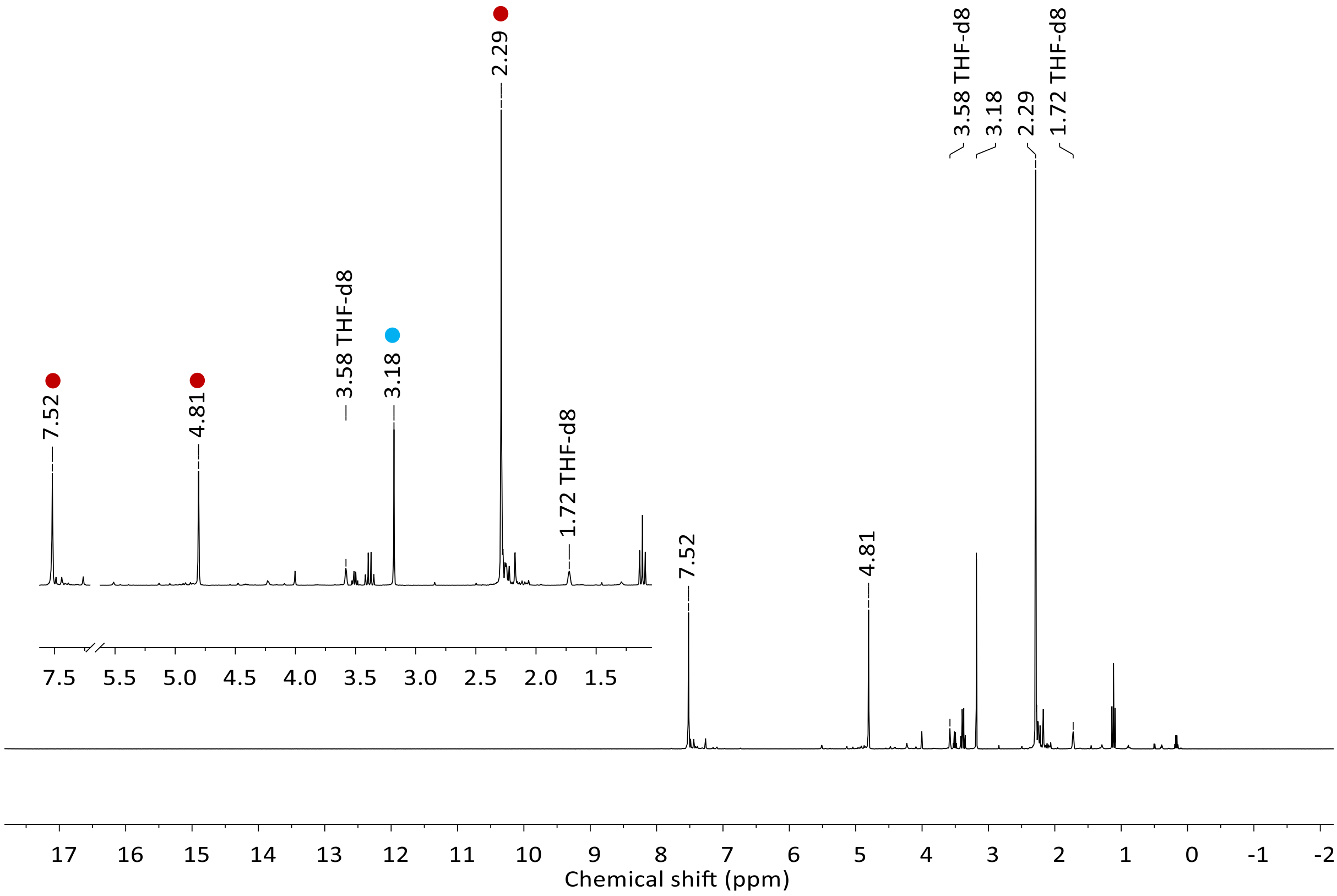

Figure S5. ^{1}H NMR spectrum (500.2 MHz, THF-d_8) of the sealed reaction mixture obtained from **DSB** and MeLi in THF-d_8, showing signals assignable to $\mathbf{1}^{\mathbf{2H}}$ (red dots) and SiH_4 (blue dot).

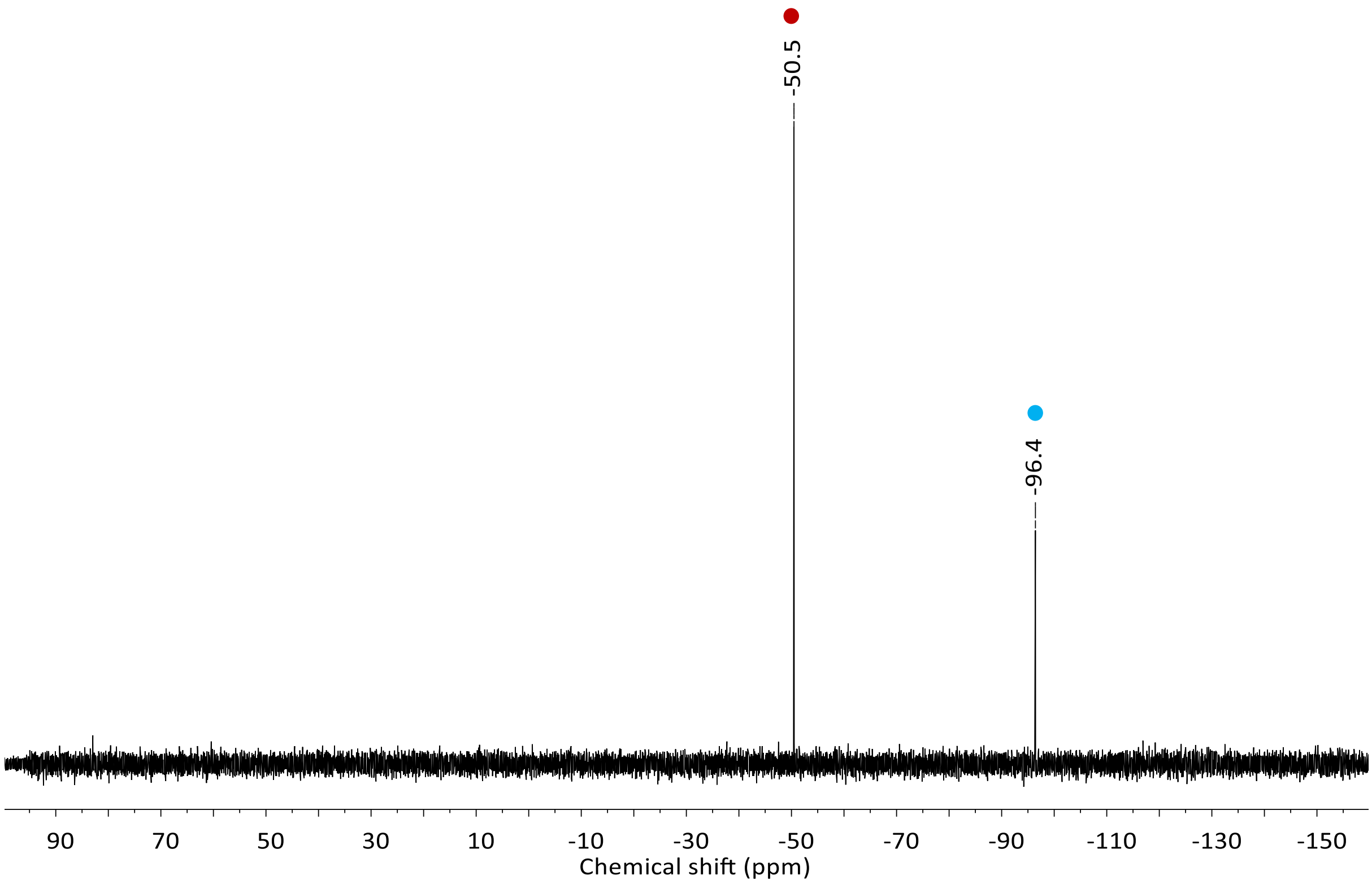

Figure S6. $^{29}Si\{^{1}H\}$ NMR spectrum (99.4 MHz, THF-d_8) of the sealed reaction mixture obtained from **DSB** and MeLi in THF-d_8, showing signals assignable to $\mathbf{1}^{\mathbf{2H}}$ (red dot) and SiH_4 (blue dot).

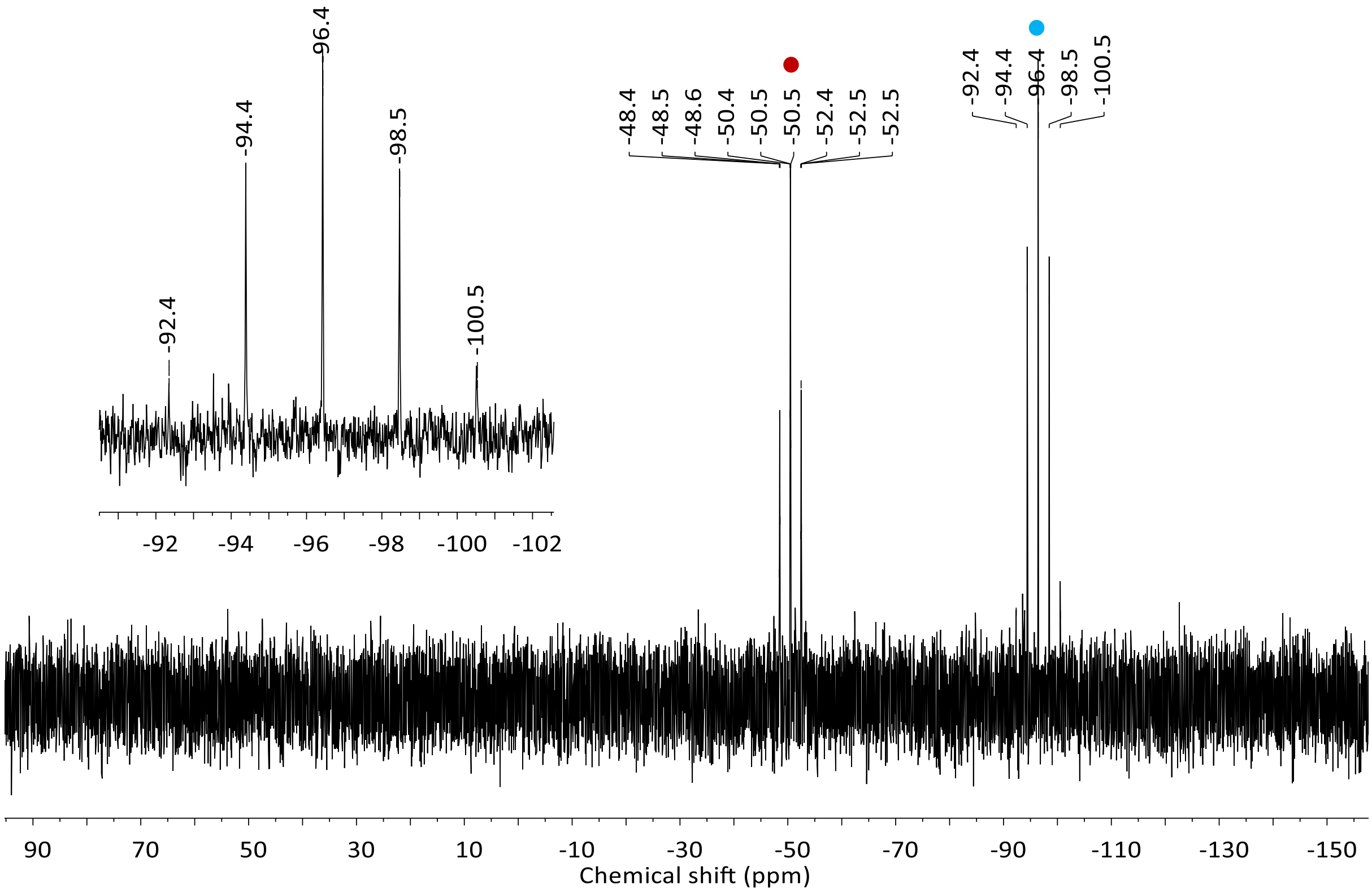

Figure S7. ^{29}Si NMR spectrum (99.4 MHz, THF-d_8) of the sealed reaction mixture obtained from **DSB** and MeLi in THF-d_8, showing signals assignable to $\mathbf{1}^{\mathbf{2H}}$ (red dot) and SiH_4 (blue dot).

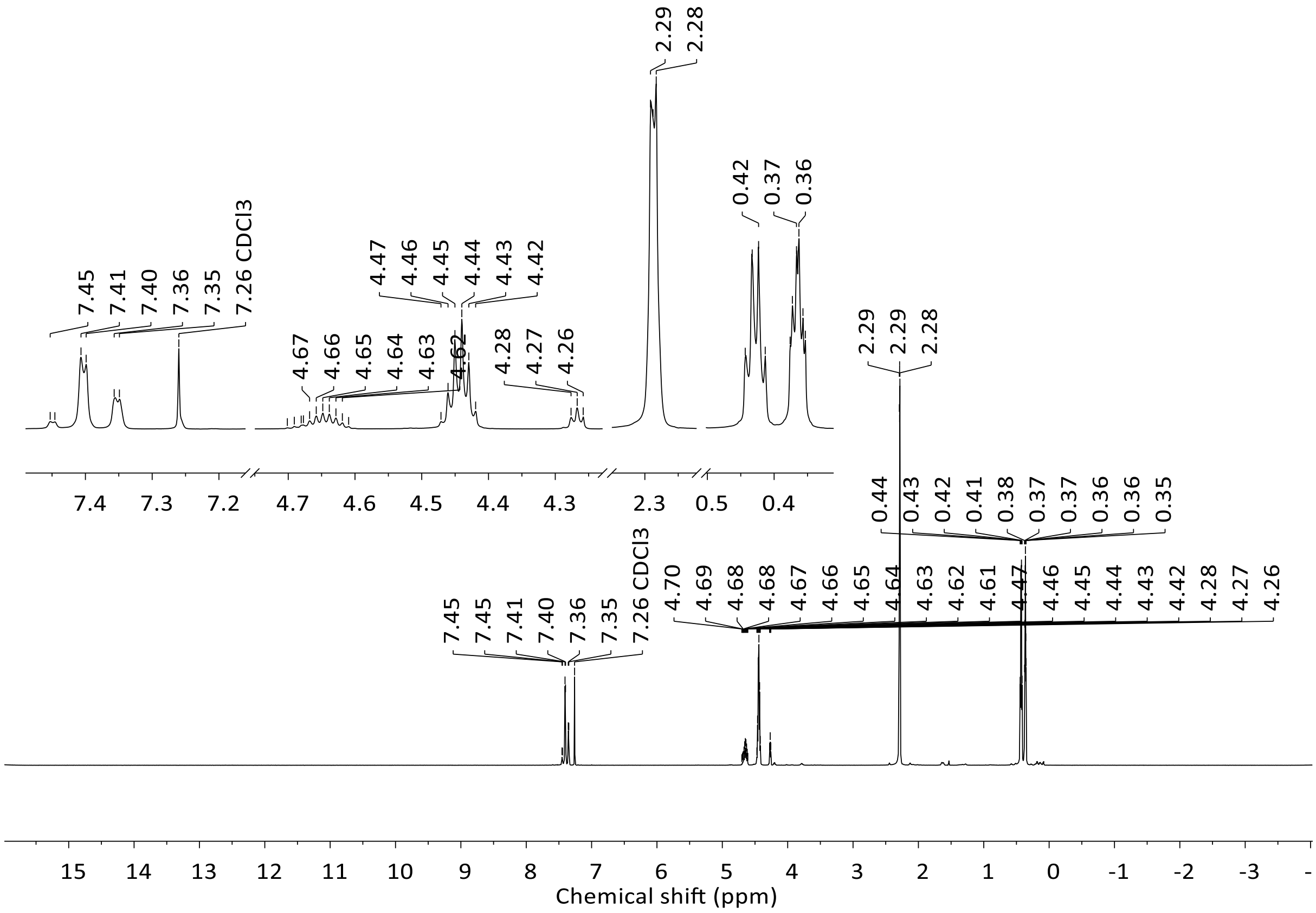

Figure S8. ^{1}H NMR spectrum (500.2 MHz, $CDCl_3$) of the liquid collected by vacuum distillation (10^{-3} mbar, 100–120 °C) of the crude residue obtained by evaporation of the reaction mixture in the synthesis of $\mathbf{1}^{\mathbf{2H}}$.

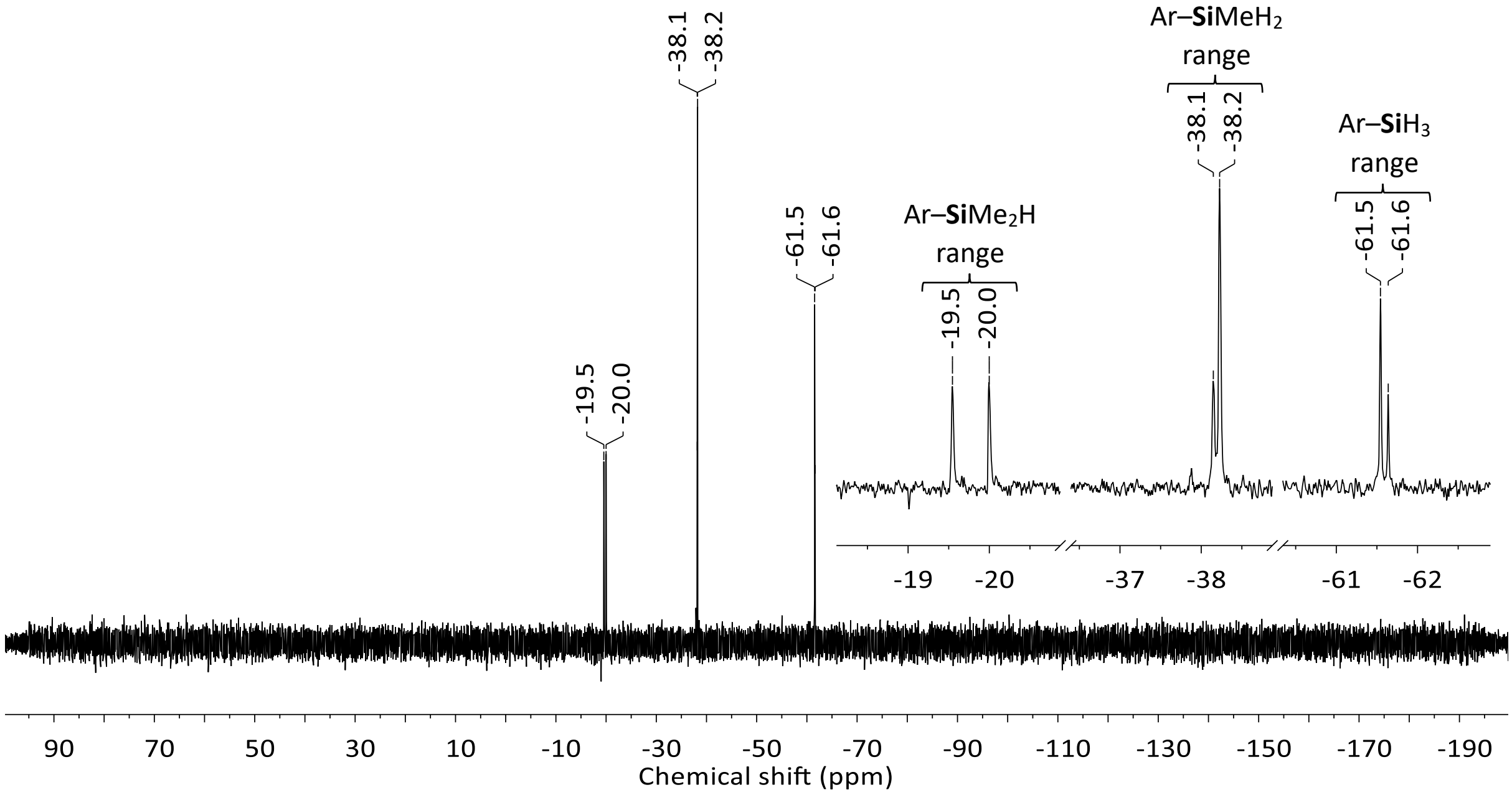

Figure S9. $^{29}Si\{^1H\}$ NMR spectrum (99.4 MHz, $CDCl_3$) of the liquid collected by vacuum distillation (10^{-3} mbar, 100–120 °C) of the crude residue obtained by evaporation of the reaction mixture in the synthesis of **1^{2H}**. Resonances are observed in chemical shift regions characteristic of Ar–SiMe$_2$H ($\delta \approx -20$ ppm), Ar–SiMeH$_2$ ($\delta \approx -38$ ppm), and Ar–SiH$_3$ ($\delta \approx -61$ ppm). For the proposed mixture **I–V**, seven ^{29}Si resonances are expected; six signals are resolved. If two resonances overlap (likely) in the Ar–SiMeH$_2$ region, the observed number and distribution of signals across these regions are consistent with a mixture of partially methylated arylhydrosilanes **I–V**.

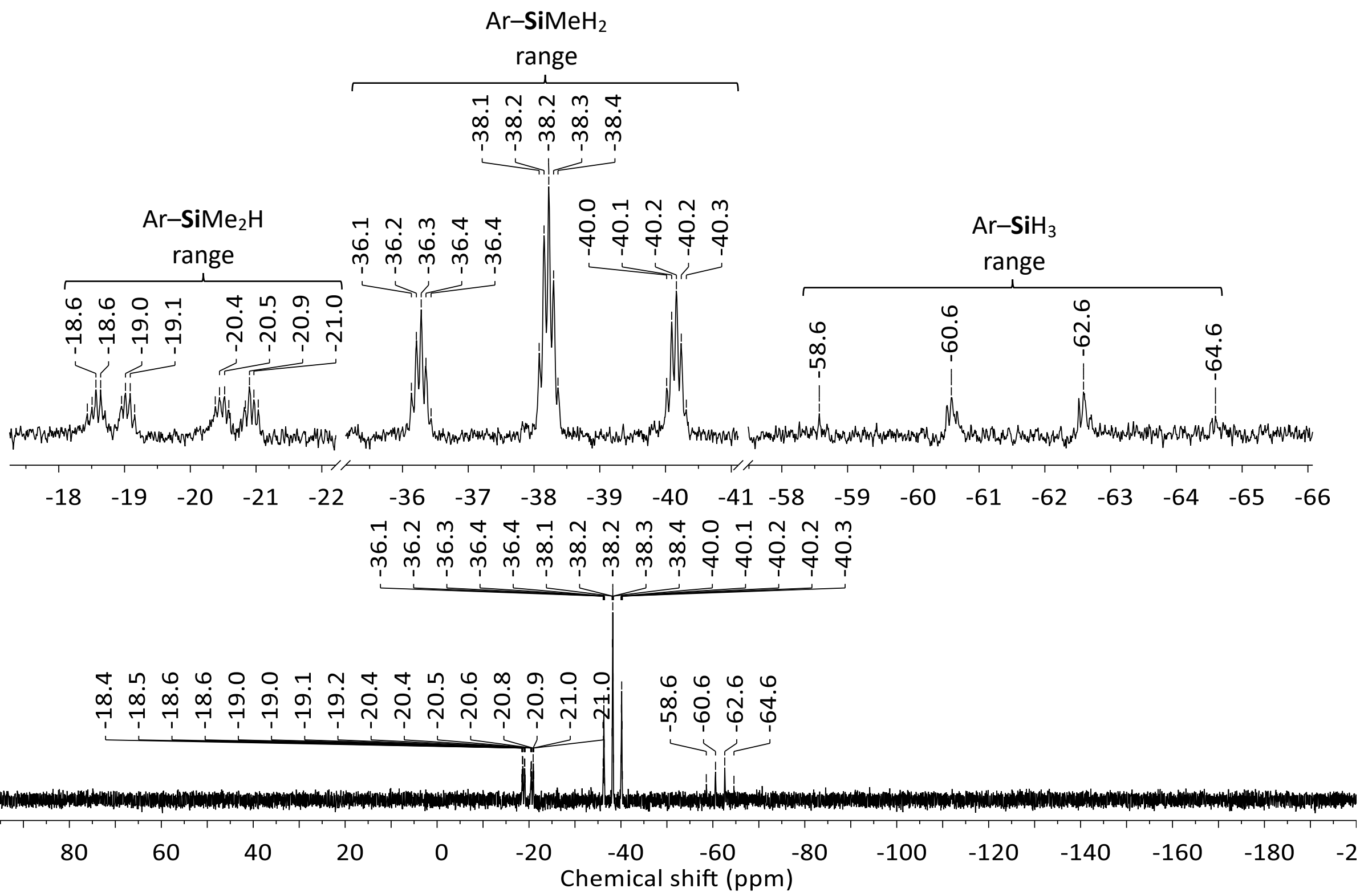

Figure S10. ^{29}Si NMR spectrum (99.4 MHz, $CDCl_3$) of the liquid collected by vacuum distillation (10^{-3} mbar, 100–120 °C) of the crude residue obtained by evaporation of the reaction mixture in the synthesis of **1^{2H}**. Resonances are observed in chemical shift regions characteristic of Ar–SiMe$_2$H ($\delta \approx -20$ ppm), Ar–SiMeH$_2$ ($\delta \approx -38$ ppm), and Ar–SiH$_3$ ($\delta \approx -61$ ppm). For the proposed mixture **I–V**, seven ^{29}Si resonances are expected; six signals are resolved. If two resonances overlap (likely) in the

Ar–SiMeH_2 region, the observed number and distribution of signals across these regions are consistent with a mixture of partially methylated arylhydrosilanes **I–V**.

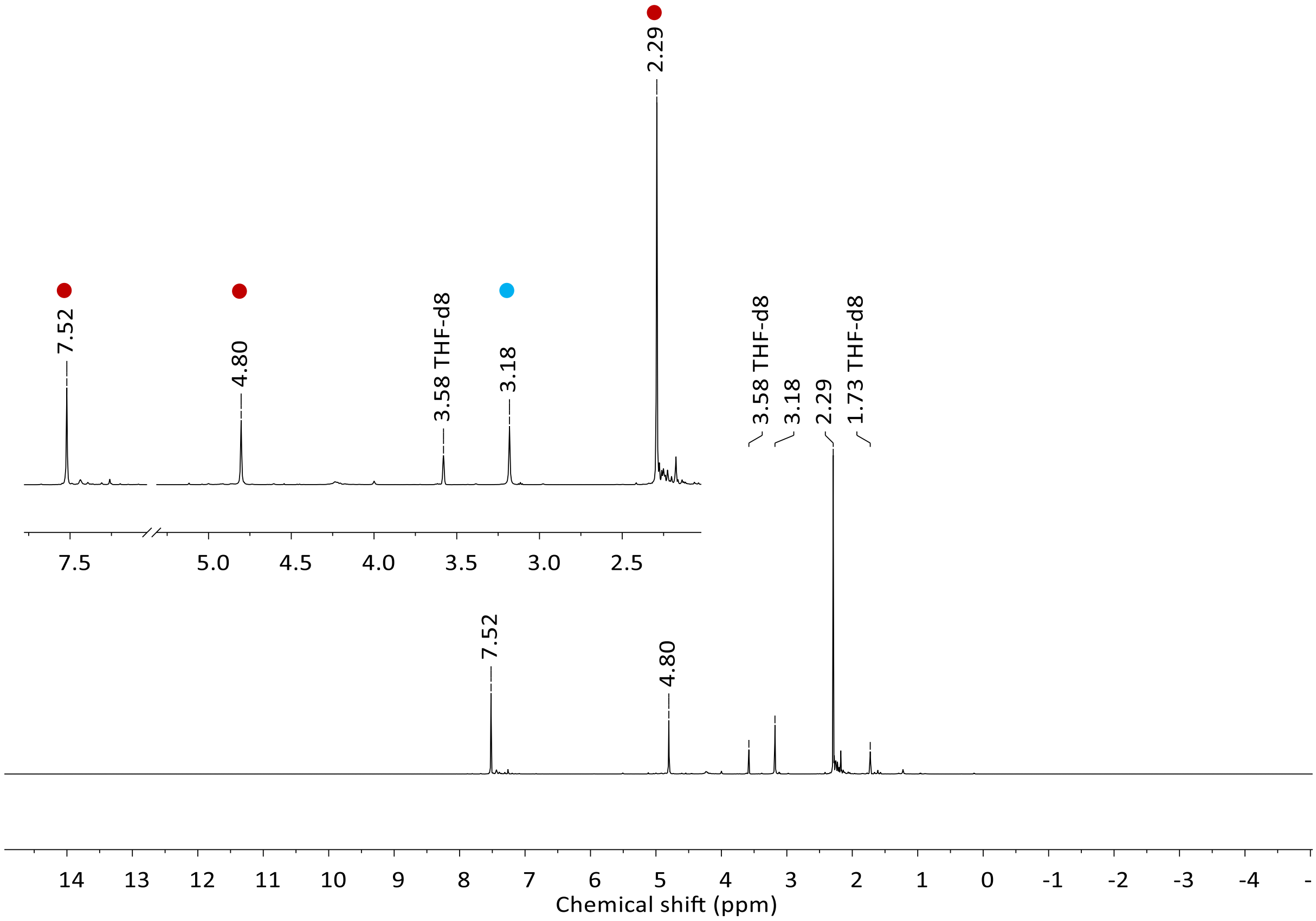

Figure S11. ^{1}H NMR spectrum (500.2 MHz, THF-d_8) of the reaction mixture obtained from **DSB** and KC_8 in THF-d_8, showing signals of $\mathbf{1}^{\mathbf{2H}}$ (red dots) and SiH_4 (blue dot).

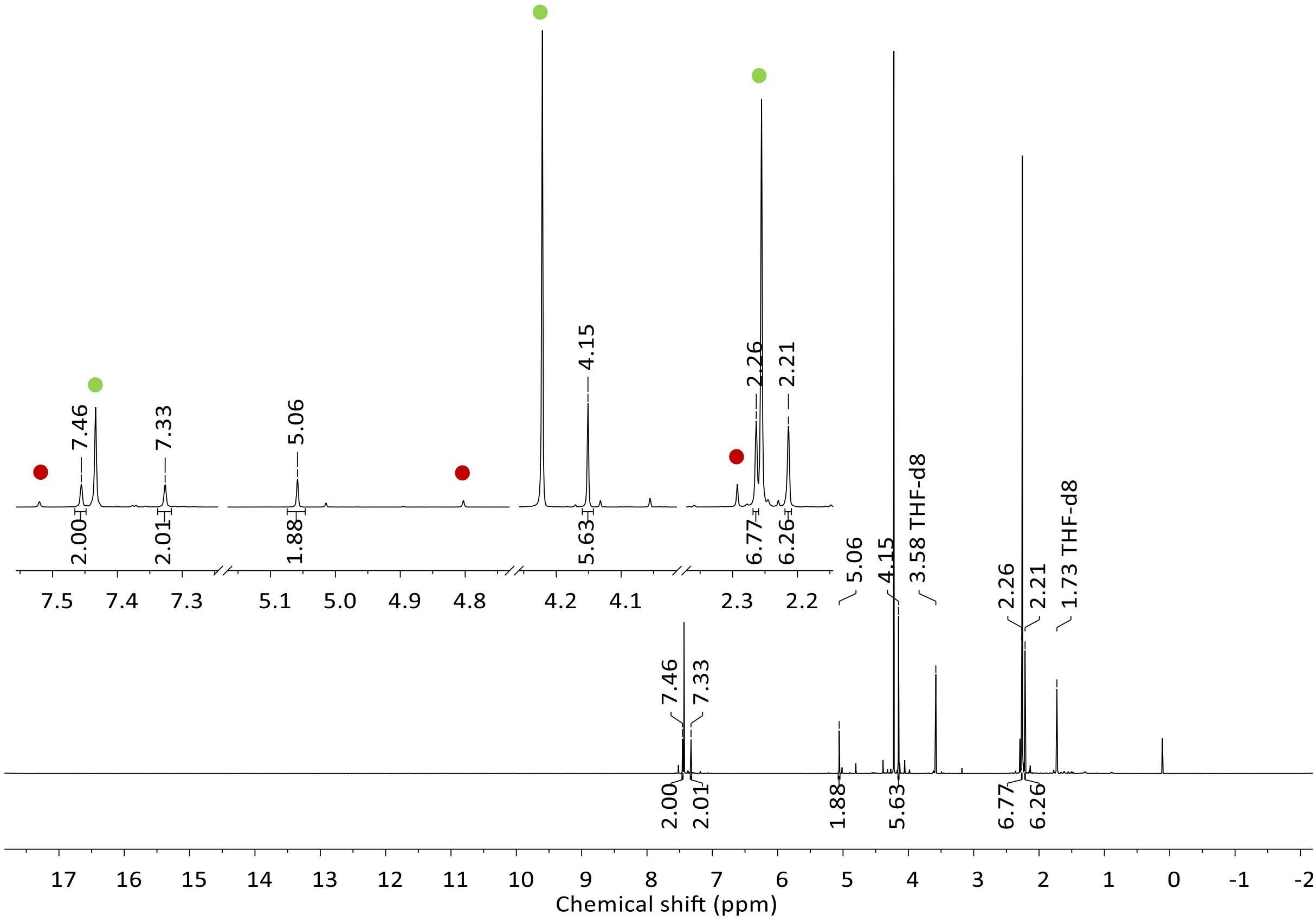

Figure S12. ^{1}H NMR spectrum (500.2 MHz, THF-d_8) of the reaction mixture obtained from **DSB** (green dots) and 0.1 equiv. KC_8 in THF-d_8 after 1 d, showing signals of intermediate **Int2** (picked) and minor amounts of **1^{2H}** (red dots).

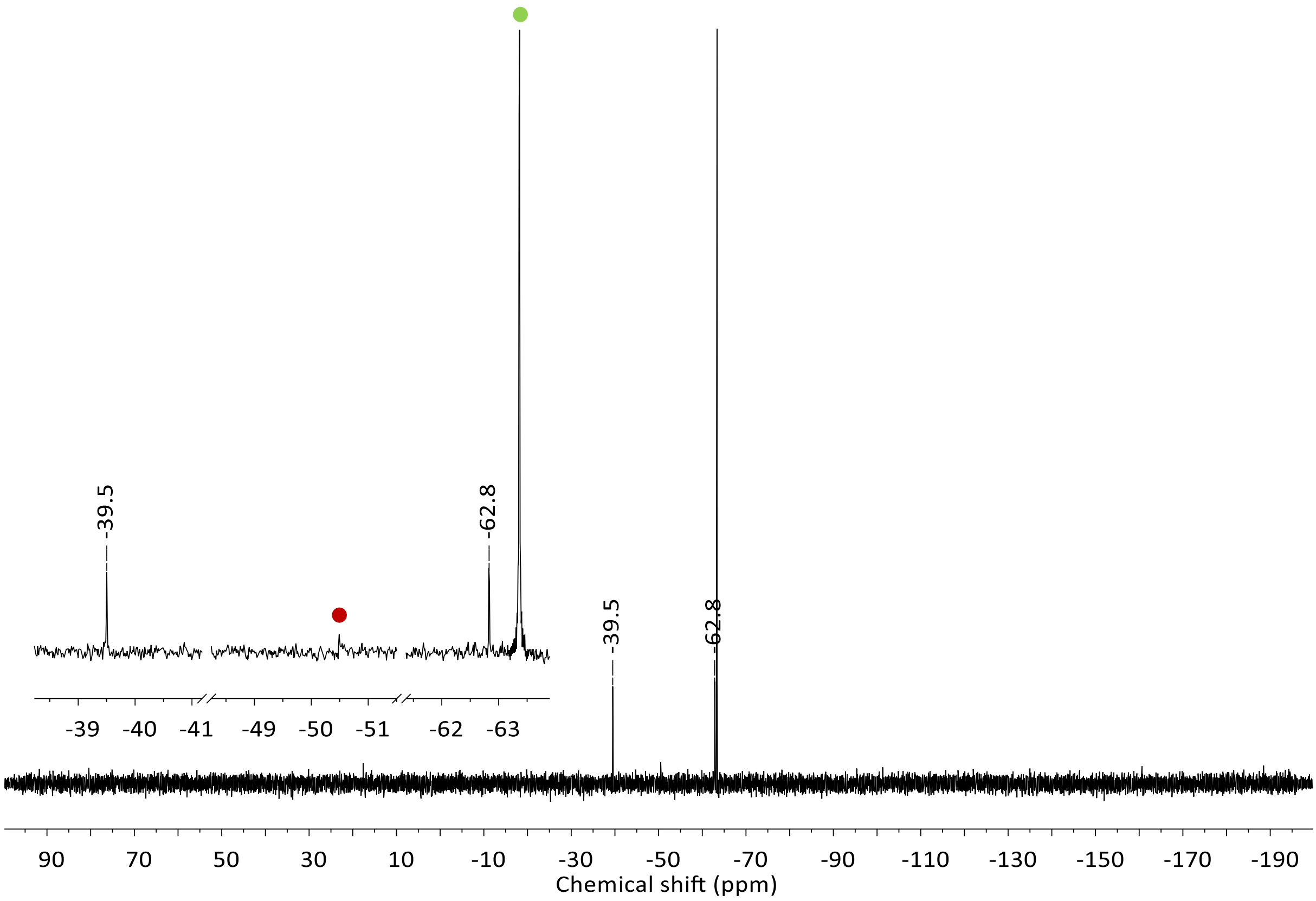

Figure S13. ^{29}Si{^{1}H} NMR spectrum (99.4 MHz, THF-d_8) of the reaction mixture obtained from **DSB** (green dot) and 0.1 equiv. KC_8 in THF-d_8 after 1 d, showing signals of intermediate **Int2** (picked) and minor amounts of **1^{2H}** (red dot).

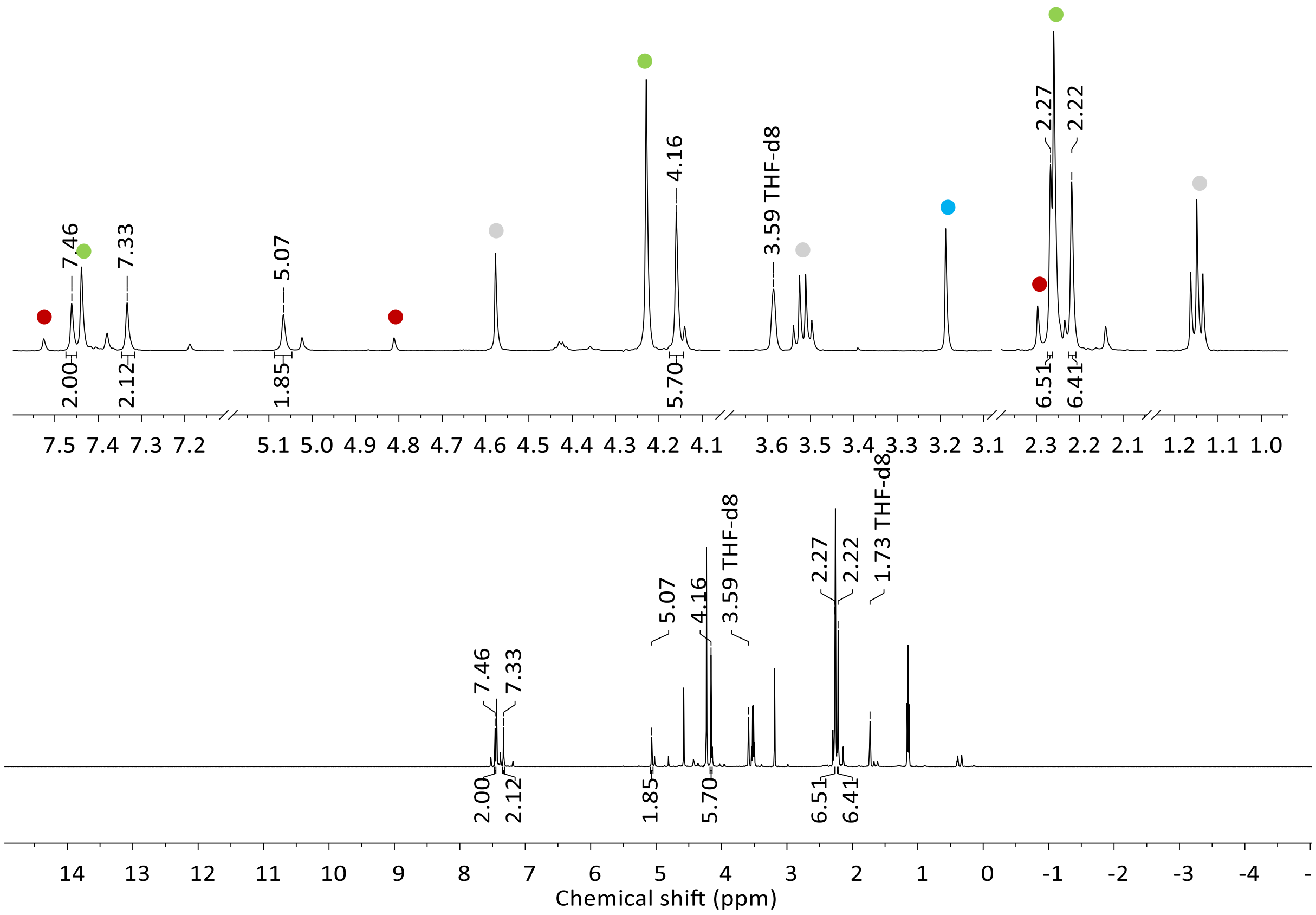

Figure S14. ^1H NMR spectrum (500.2 MHz, THF-d_8) of the reaction mixture obtained from **DSB** and MeLi (solution in diethoxymethane; grey dots) after 30 min. Resonances assignable to **Int2** are picked, while signals of the starting material **DSB** (green dots), the final product $\mathbf{1^{2H}}$ (red dots), and SiH_4 (blue dot) are marked accordingly.

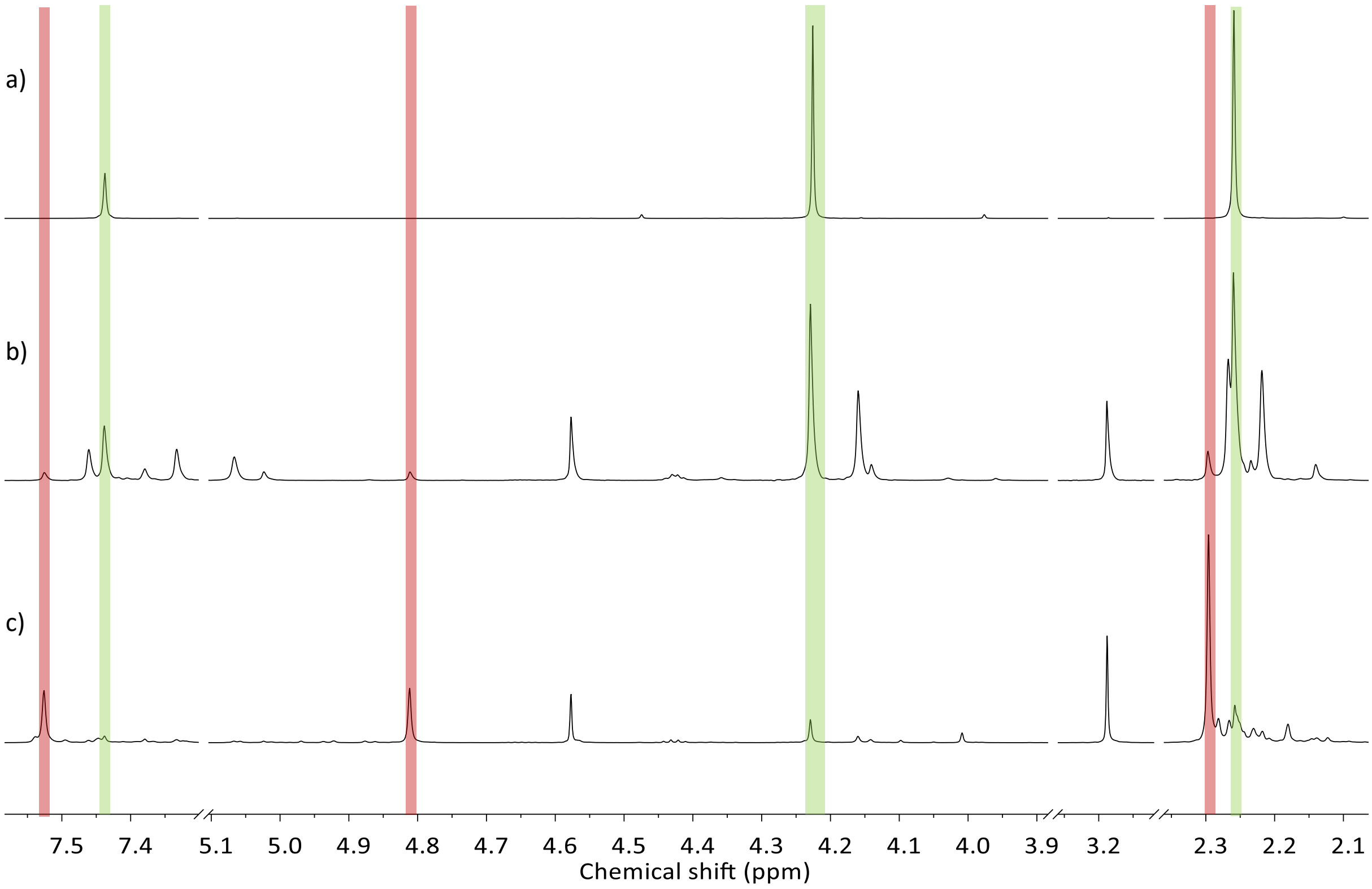

Figure S15. Stacked ^{1}H NMR spectra (500.2 MHz, THF-d_8) of the reaction mixture obtained from **DSB** and MeLi (solution in diethoxymethane) after different reaction times: a) **DSB** (green) prior to MeLi addition, b) 30 min after MeLi addition, and c) 1 d after MeLi addition, showing $\mathbf{1}^{\mathbf{2H}}$ (red) as the major product. Only selected spectral regions are shown for clarity.

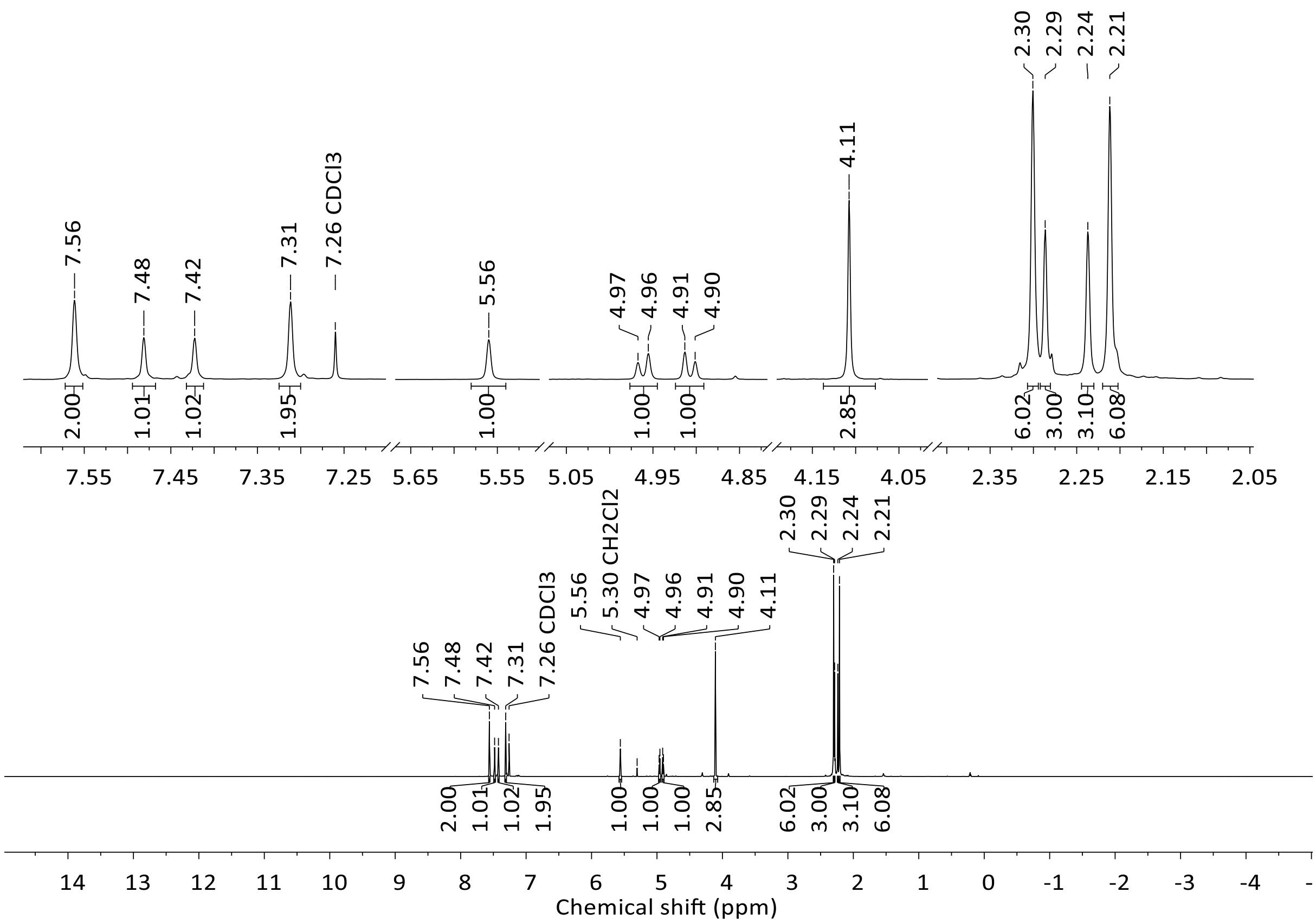

Figure S16. ^{1}H NMR spectrum (500.2 MHz, $CDCl_3$) of **VI**.

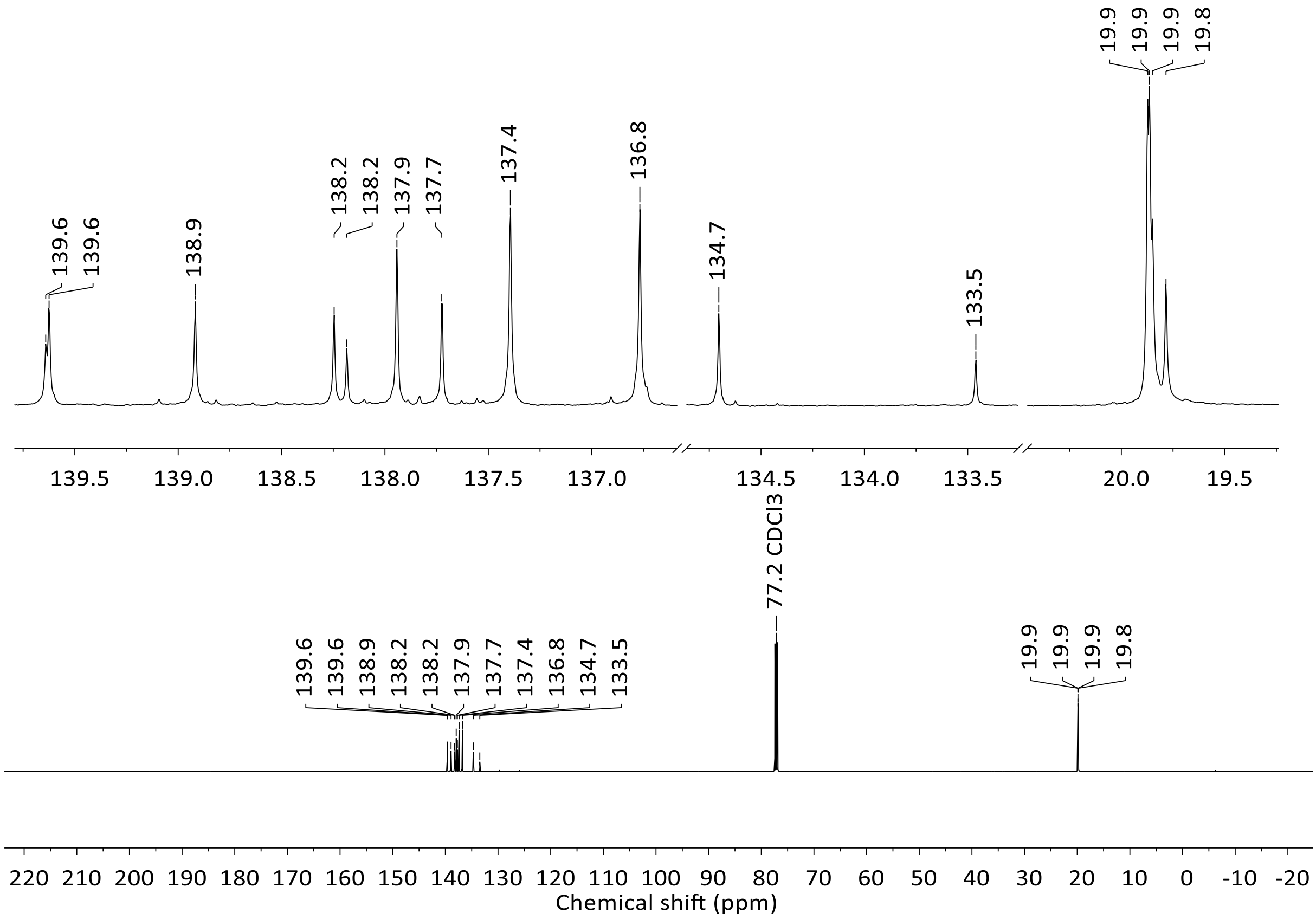

Figure S17. $^{13}C\{^{1}H\}$ NMR spectrum (125.8 MHz, $CDCl_3$) of **VI**.

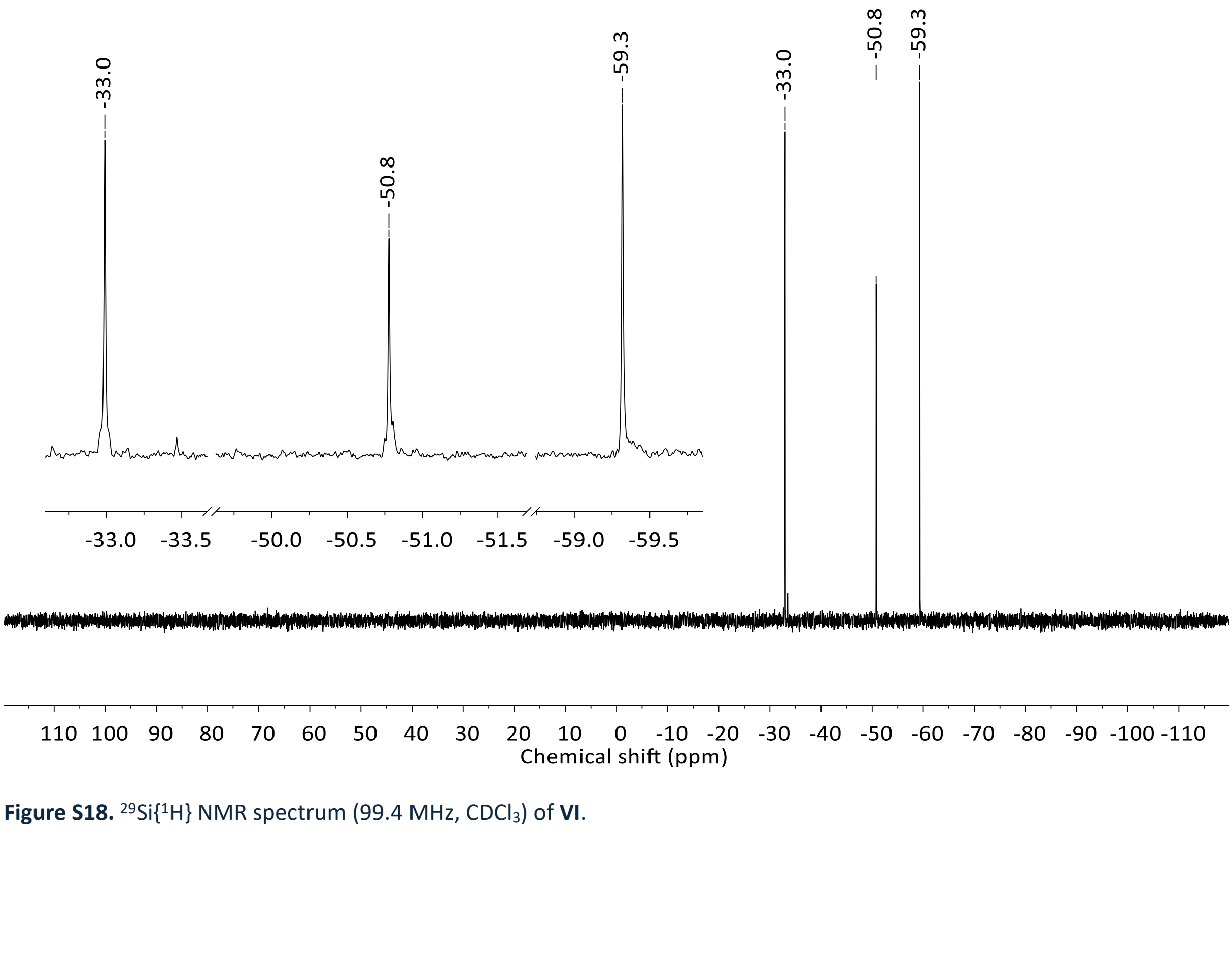

Figure S18. $^{29}Si\{^{1}H\}$ NMR spectrum (99.4 MHz, $CDCl_3$) of **VI**.

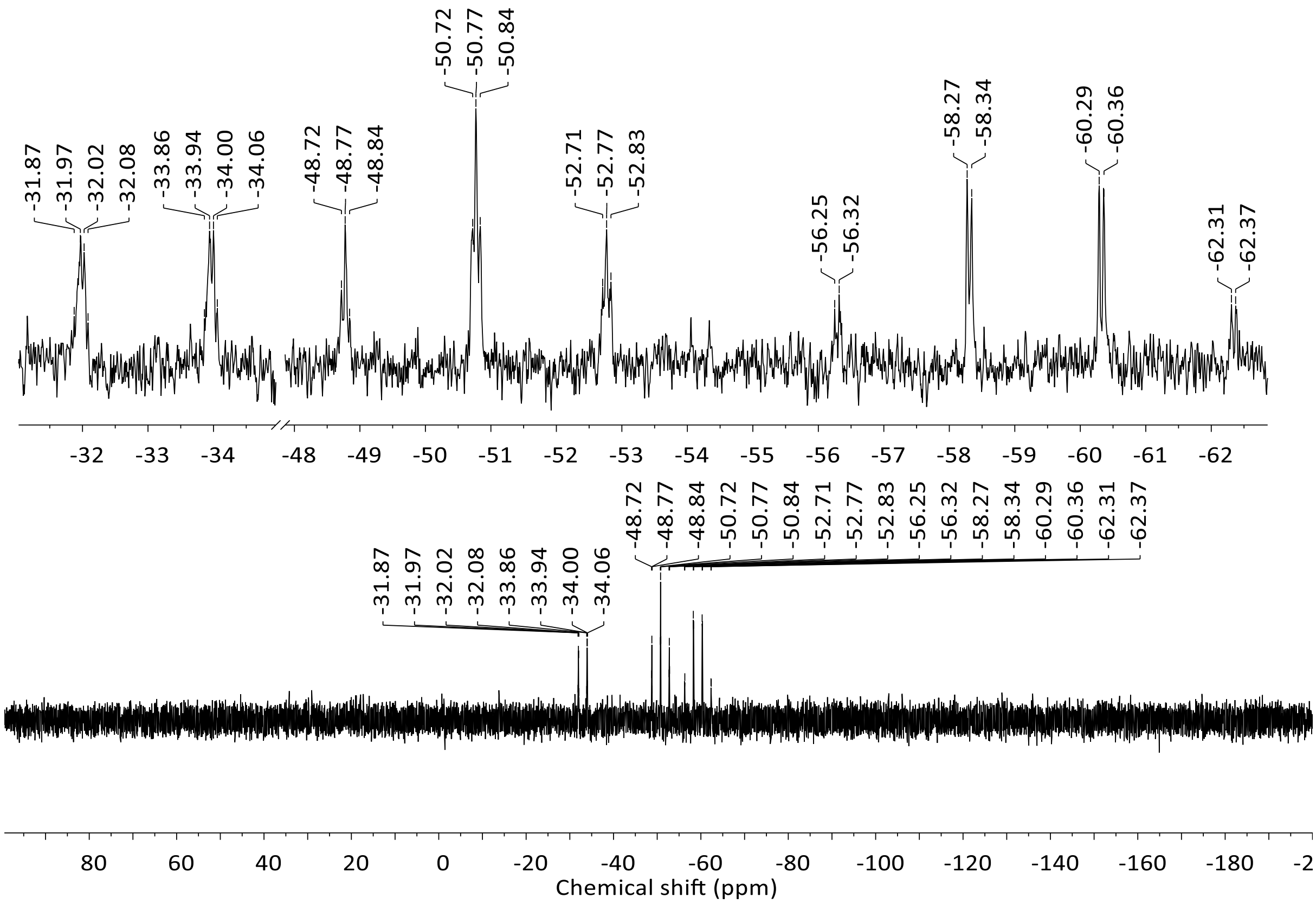

Figure S19. ^{29}Si NMR spectrum (99.4 MHz, $CDCl_3$) of **VI**.

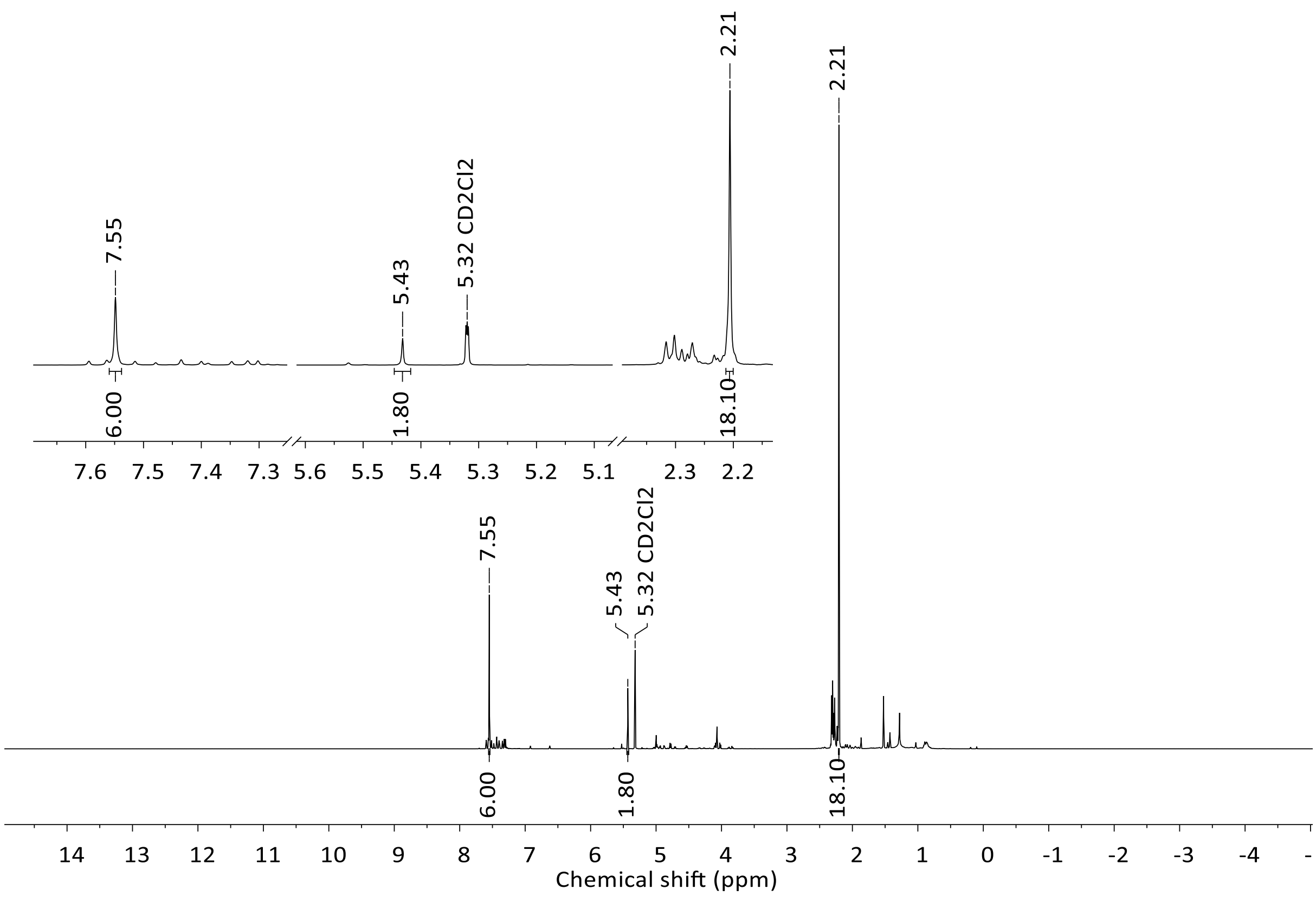

Figure S20. ^{1}H NMR spectrum (500.2 MHz, CD_2Cl_2) of **VII**.

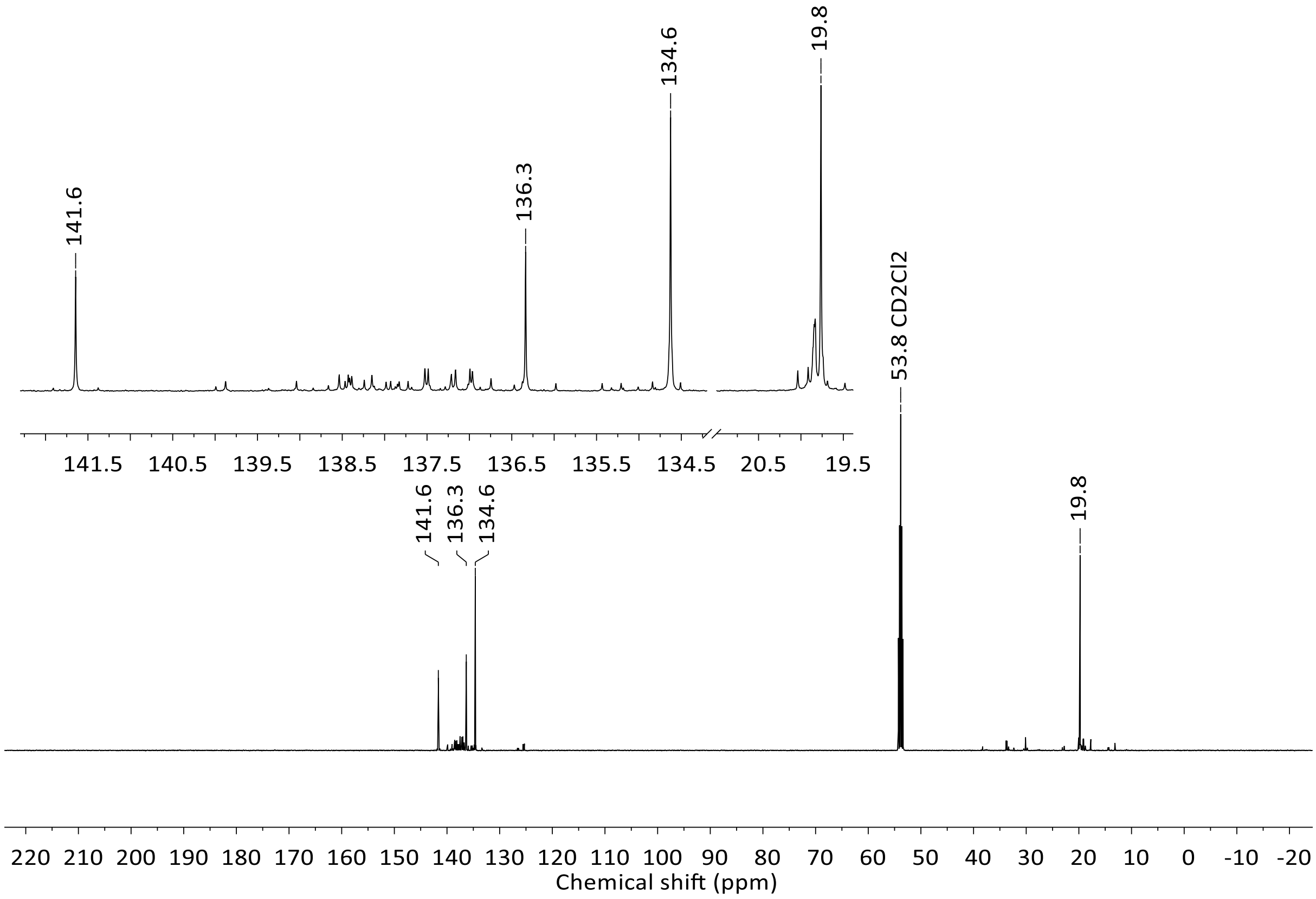

Figure S21. $^{13}C\{^{1}H\}$ NMR spectrum (125.8 MHz, CD_2Cl_2) of **VII**.

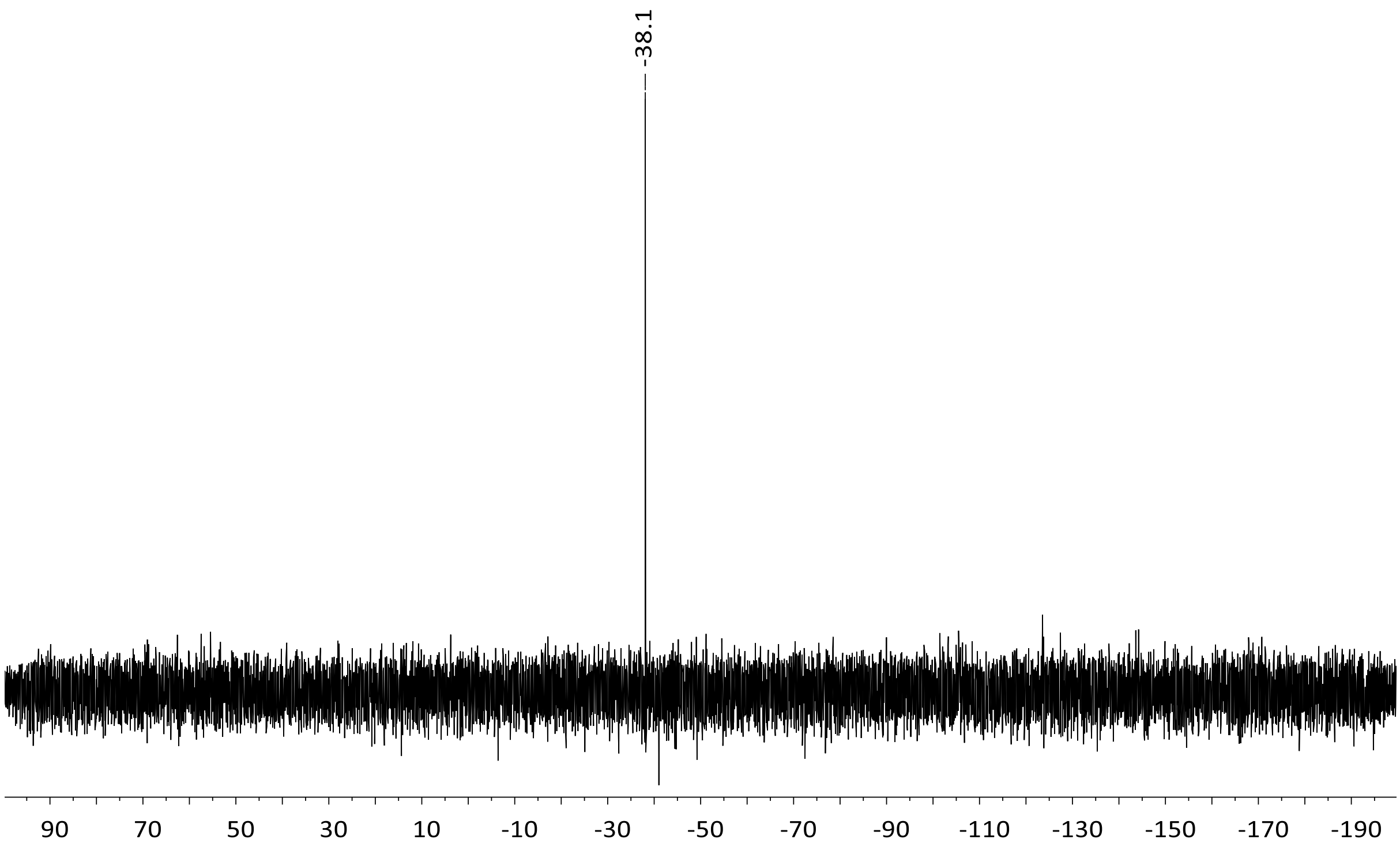

Figure S22. $^{29}Si\{^{1}H\}$ NMR spectrum (99.4 MHz, CD_2Cl_2) of **VII**.

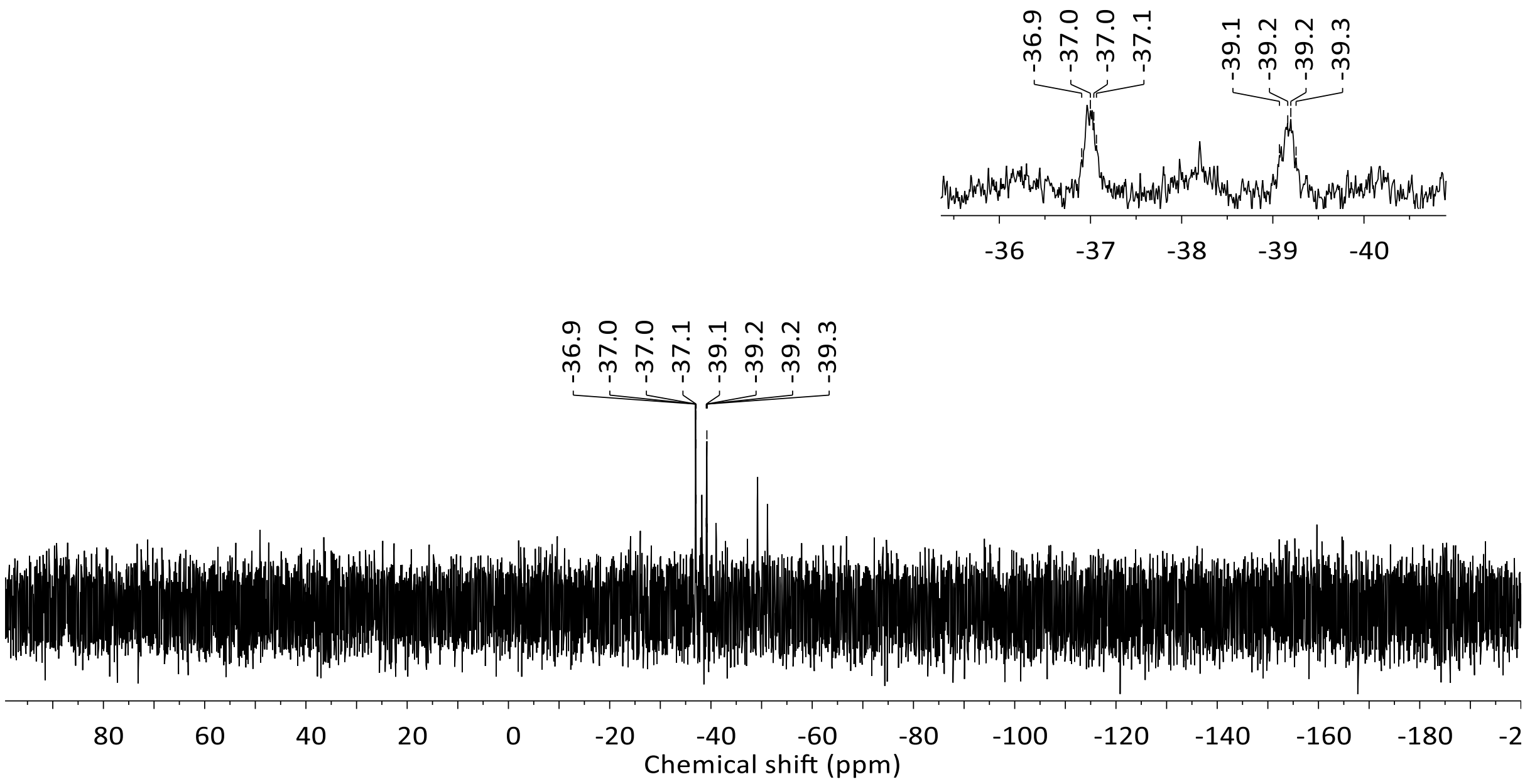

Figure S23. ^{29}Si NMR spectrum (99.4 MHz, CD_2Cl_2) of **VII**.

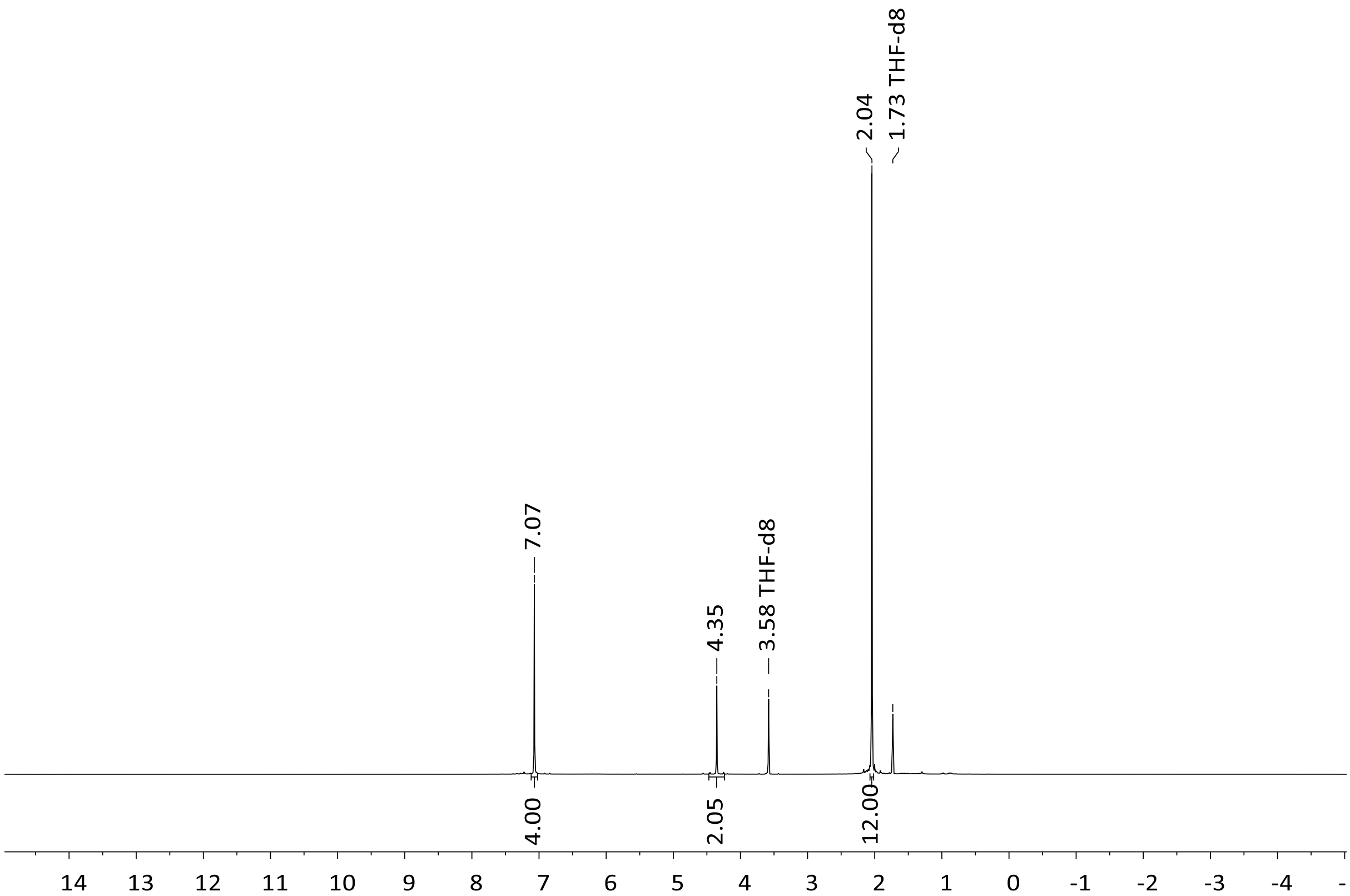

Figure S24. ^{1}H NMR spectrum (500.2 MHz, THF-d_8) of K_2[**1**].

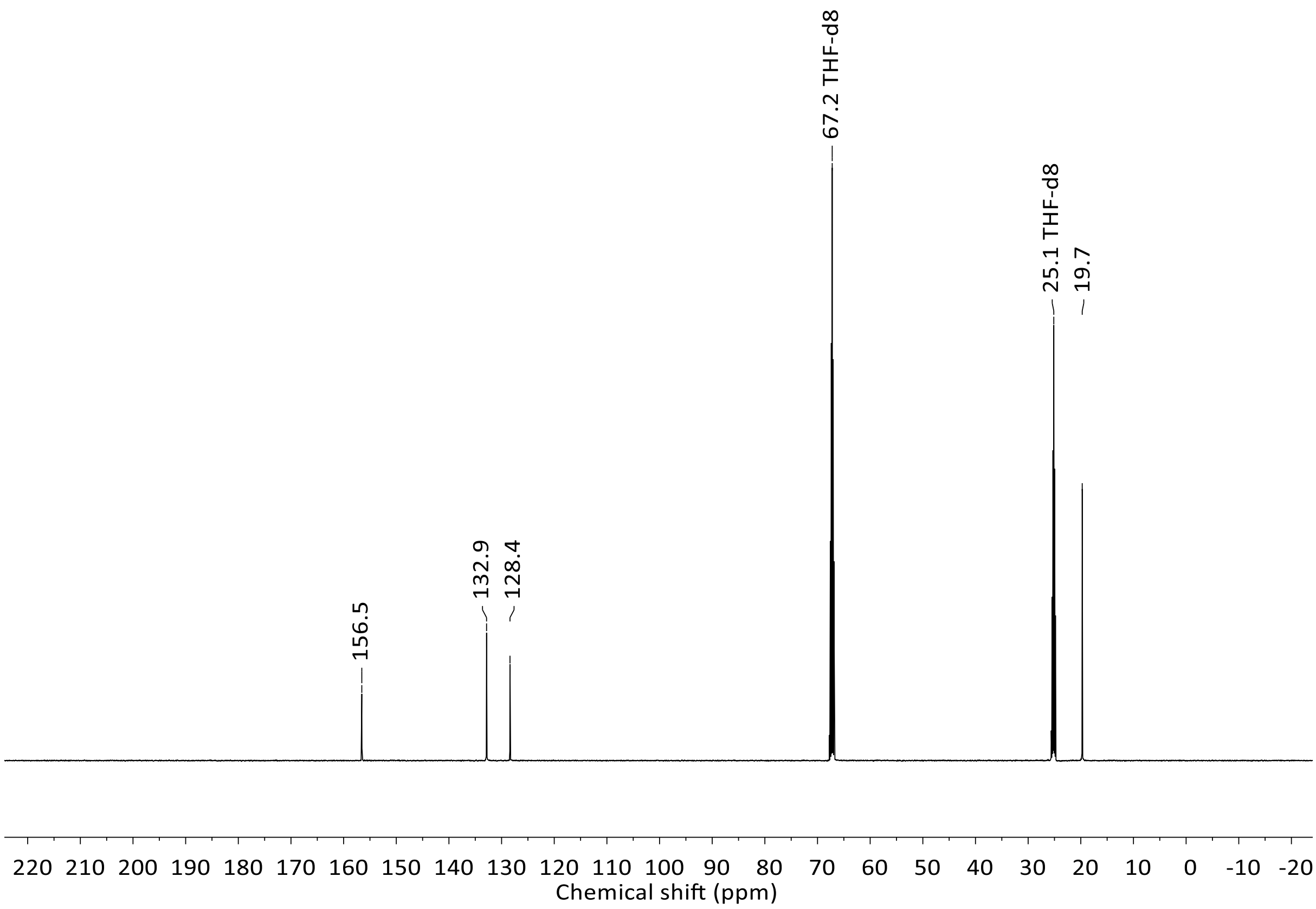

Figure S25. $^{13}C\{^{1}H\}$ NMR spectrum (125.8 MHz, THF-d_8) of K_2[**1**].

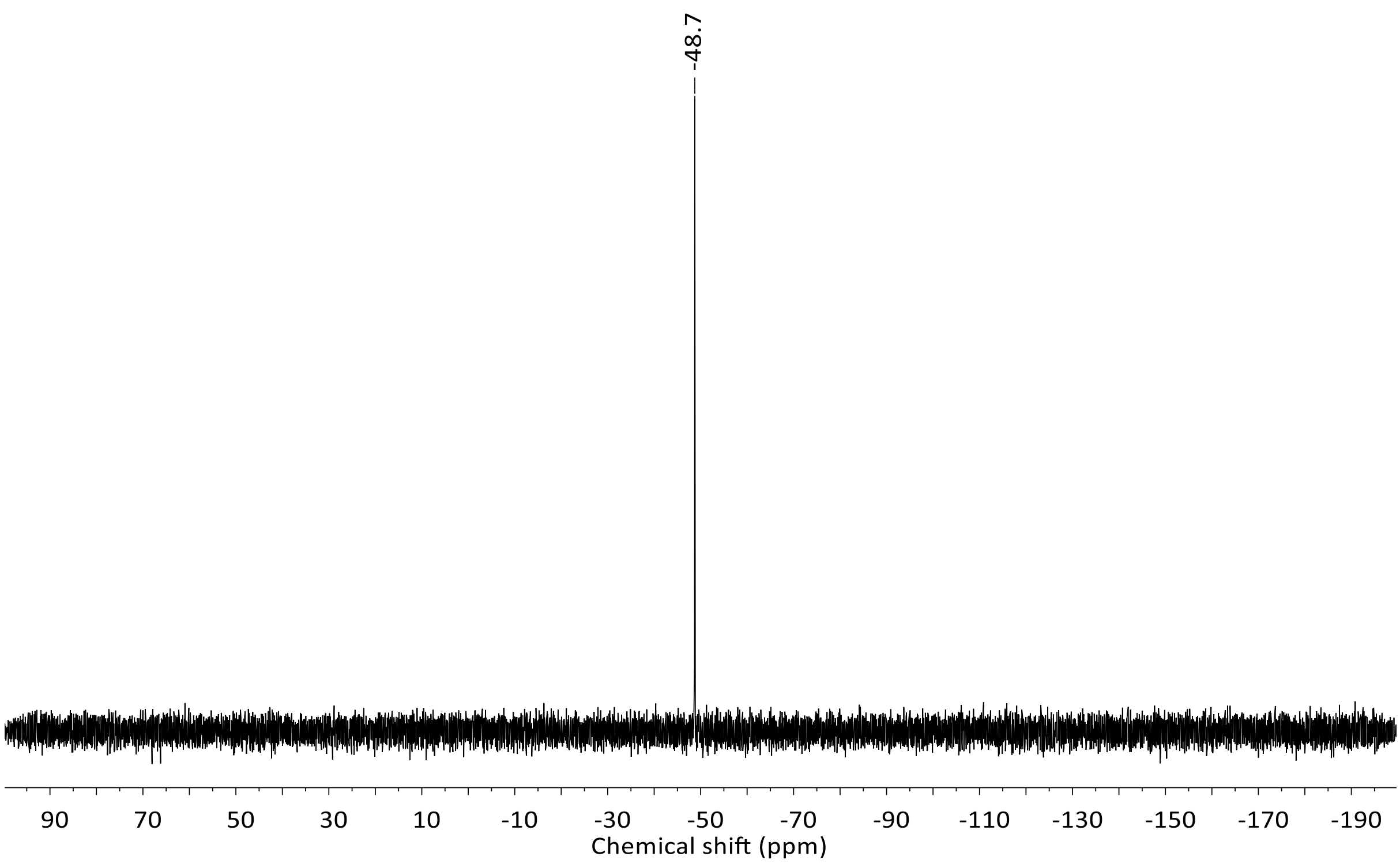

Figure S26. $^{29}Si\{^{1}H\}$ NMR spectrum (99.4 MHz, THF-d_8) of K_2[**1**].

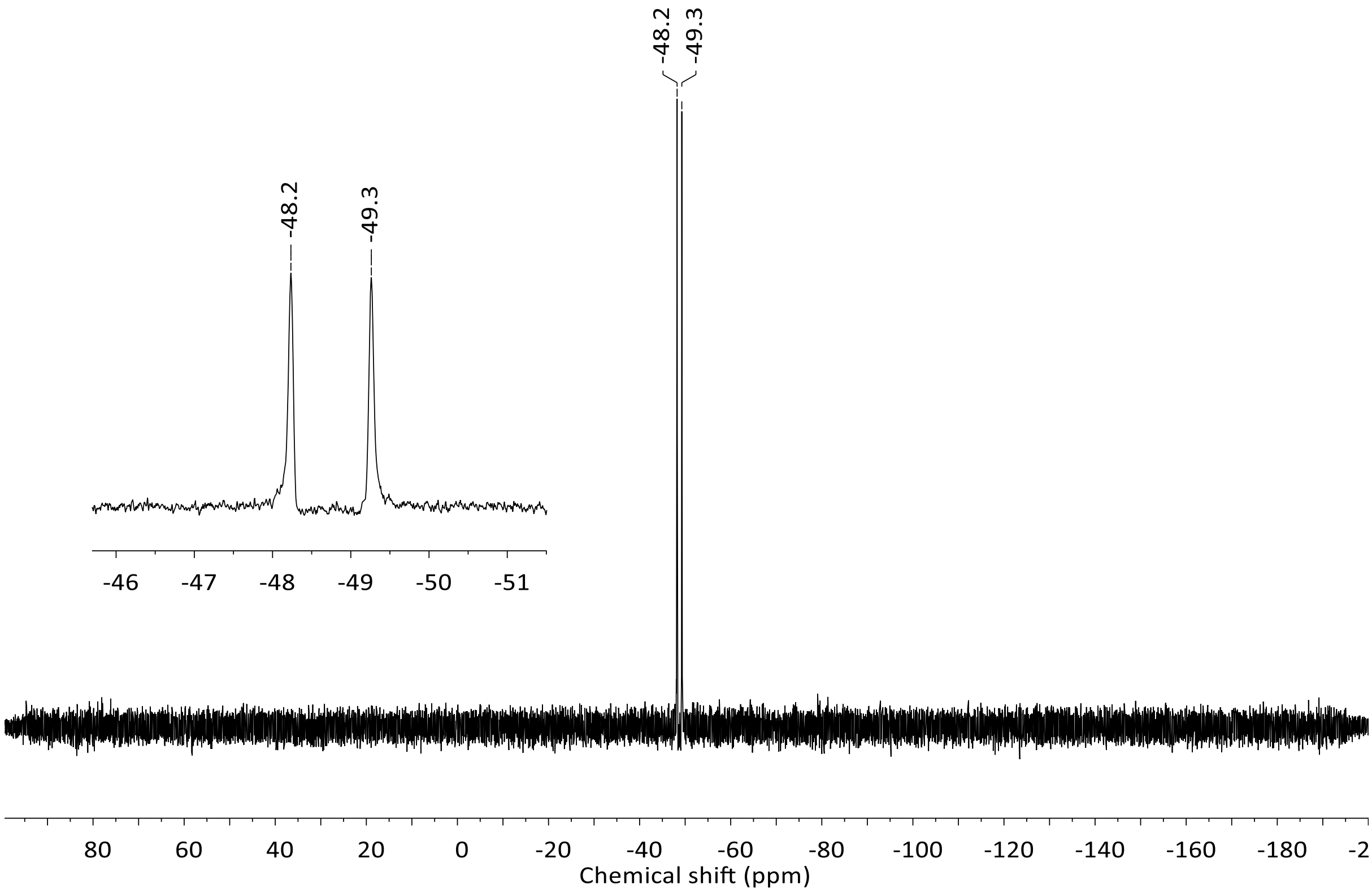

Figure S27. ^{29}Si NMR spectrum (99.4 MHz, THF-d_8) of K_2[**1**].

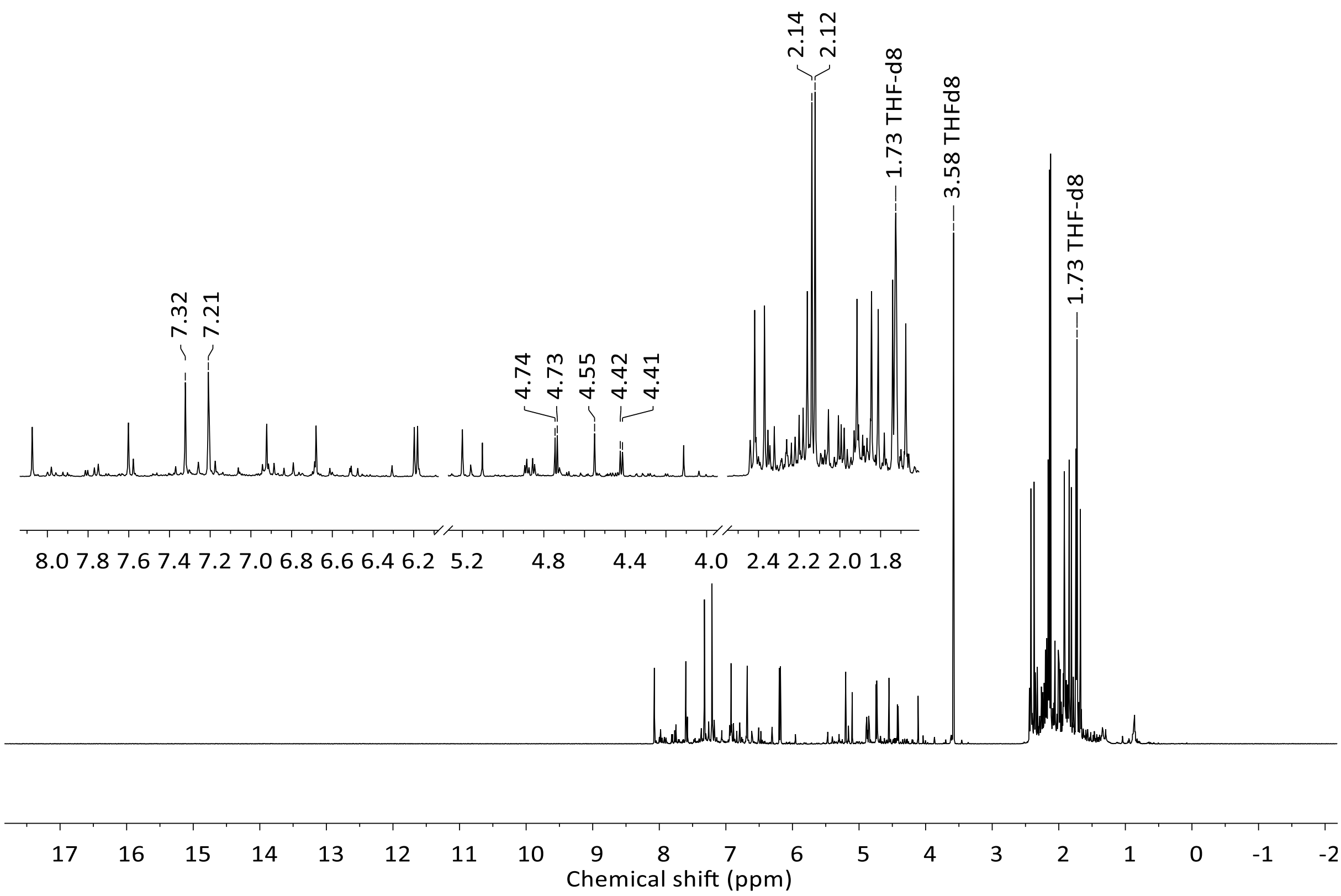

Figure S28. ^{1}H NMR spectrum (500.2 MHz, THF-d_8) of the reaction mixture obtained after treatment of $\mathbf{1^{2H}}$ with Li metal in THF-d_8 (1 d, room temperature), showing formation of multiple species. Signals tentatively attributable to Li[$\mathbf{1^{H}}$] are picked; however, unambiguous assignment was not possible.

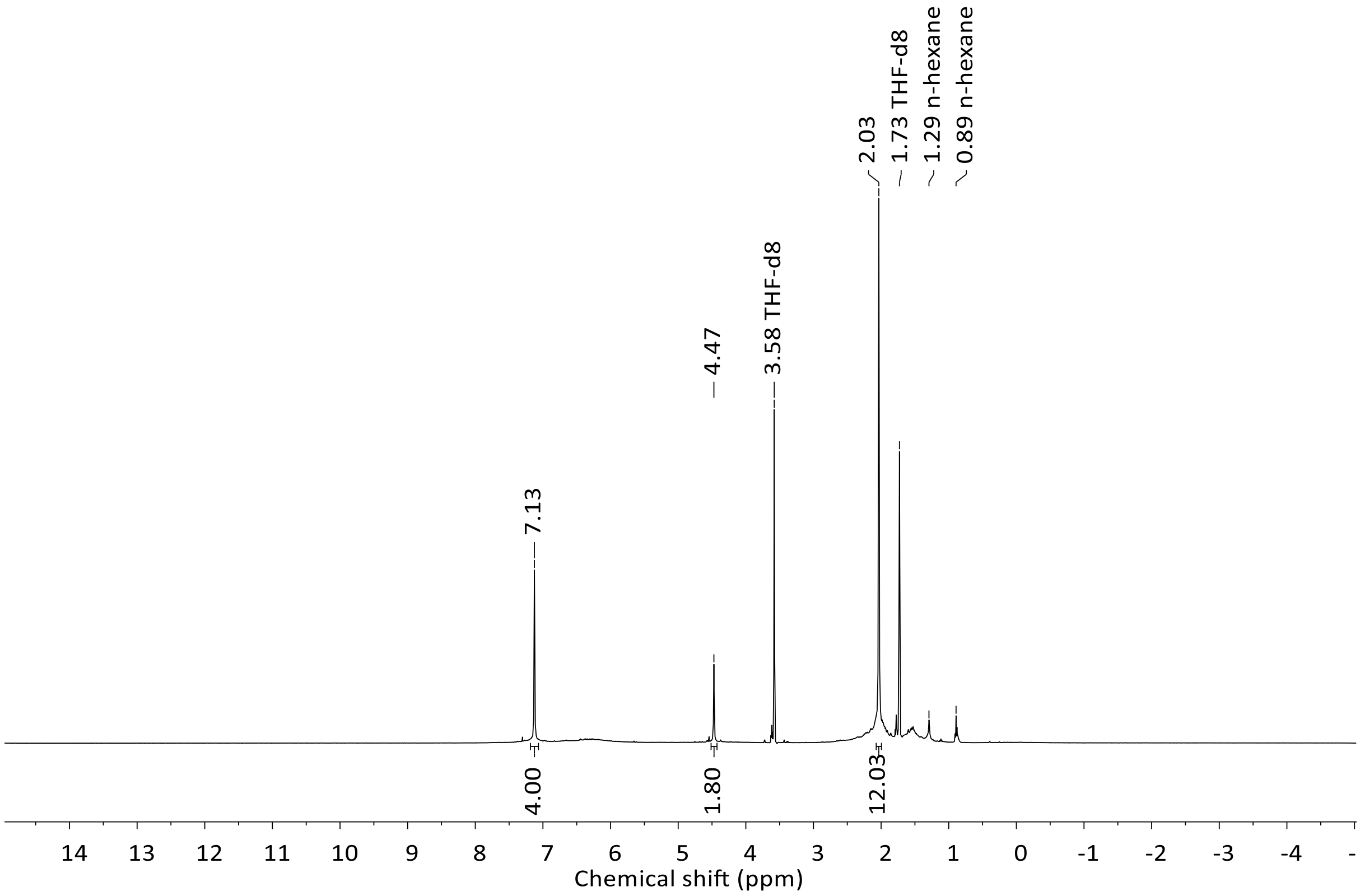

Figure S29. ^{1}H NMR spectrum (500.2 MHz, THF-d_8) of Rb_2[**1**].

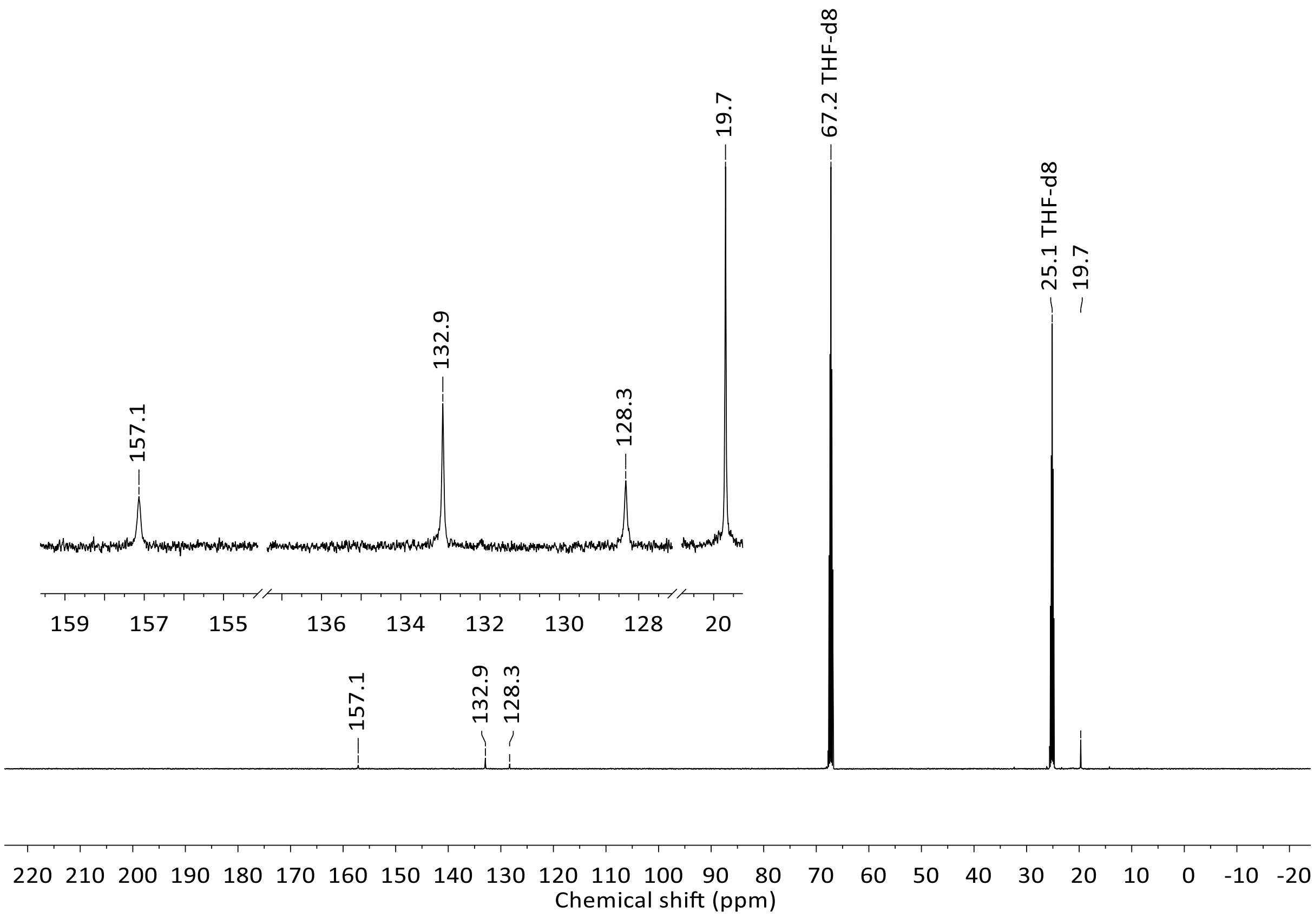

Figure S30. $^{13}C\{^{1}H\}$ NMR spectrum (125.8 MHz, THF-d_8) of Rb_2[**1**].

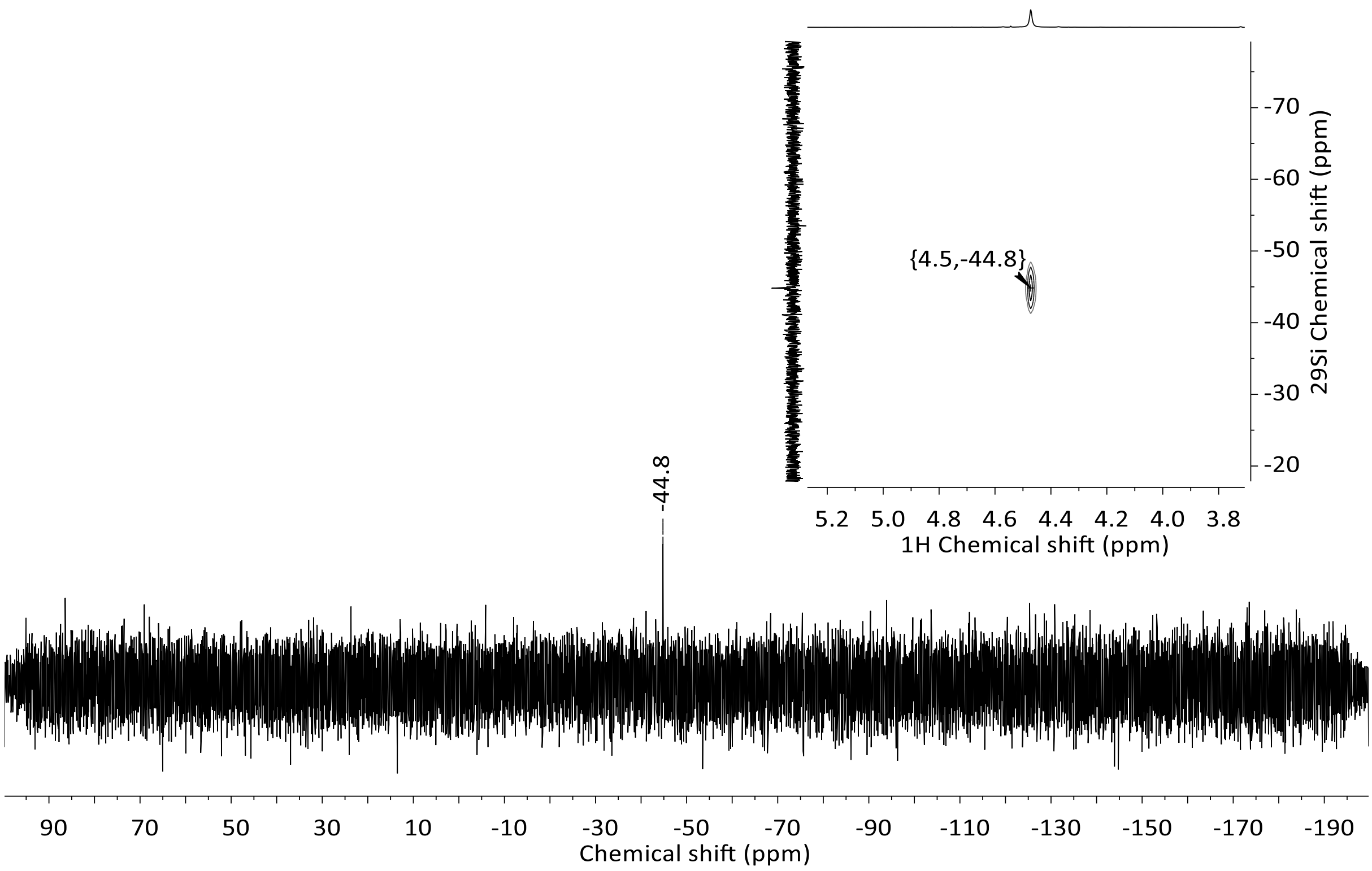

Figure S31. $^{29}Si\{^{1}H\}$ NMR spectrum (99.4 MHz, THF-d_8) of Rb_2[**1**], including an excerpt of the ^{1}H–^{29}Si HSQC NMR spectrum (^{1}H: 500.2 MHz, ^{29}Si: 99.4 MHz, THF-d_8).

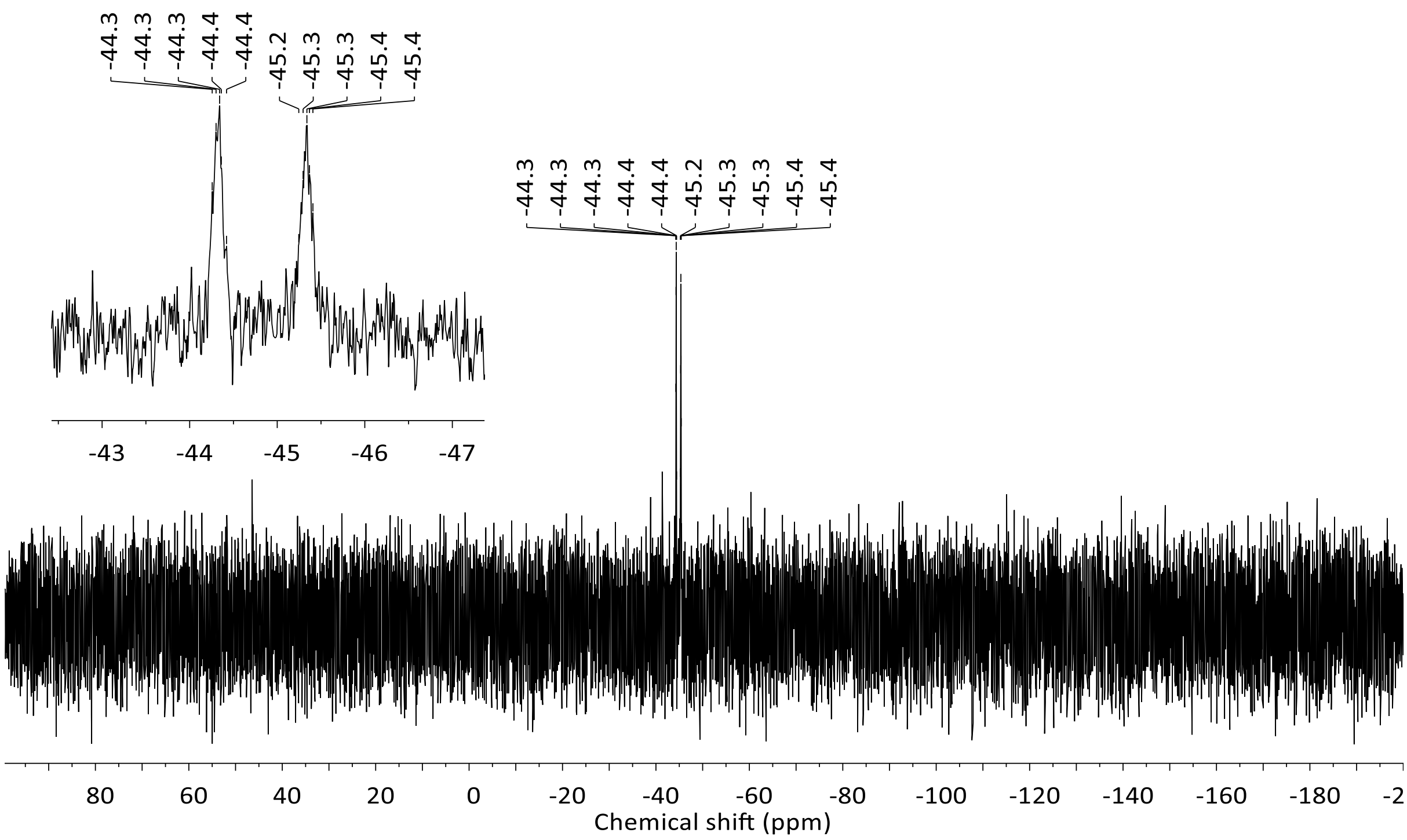

Figure S32. ^{29}Si NMR spectrum (99.4 MHz, THF-d_8) of Rb_2[**1**].

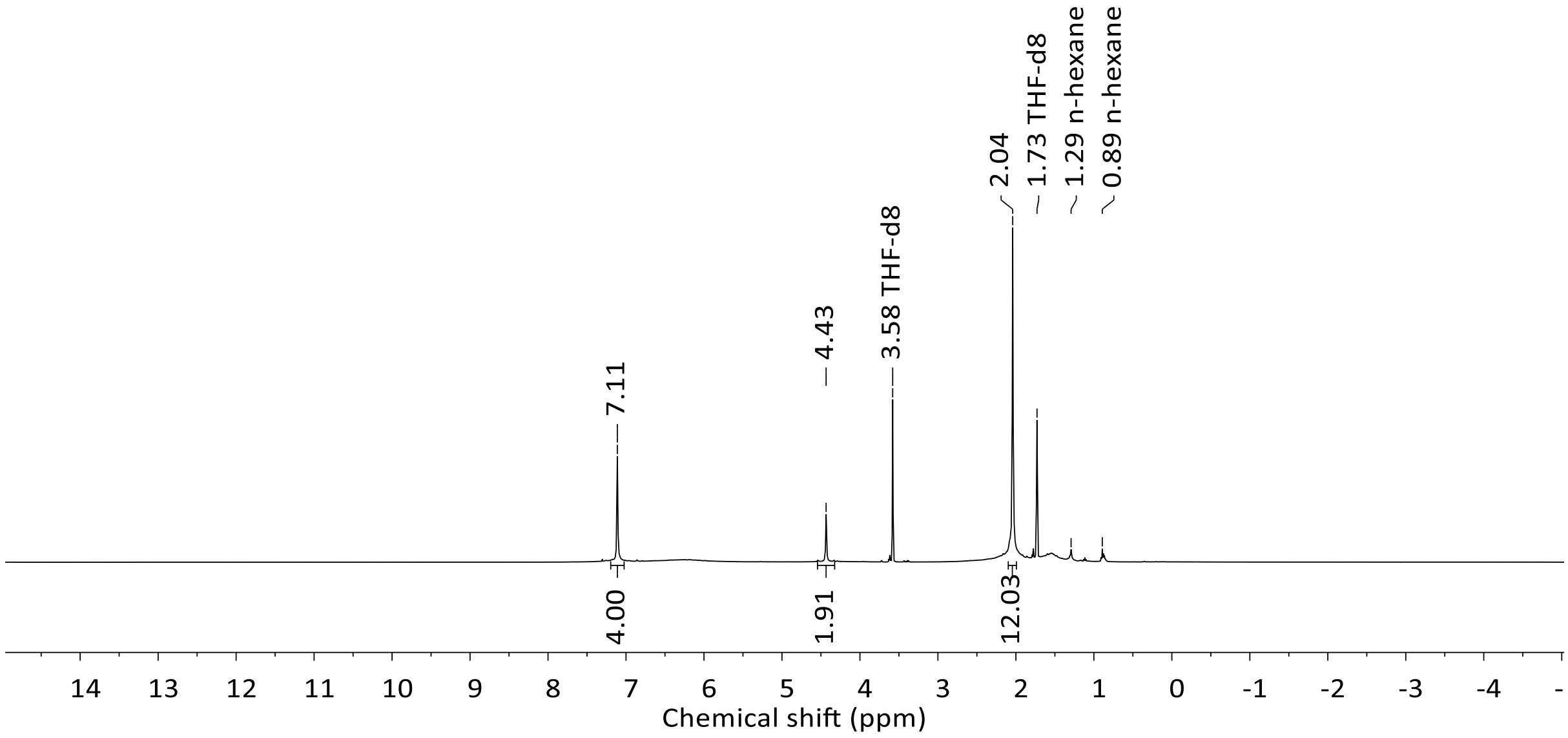

Figure S33. ^{1}H NMR spectrum (500.2 MHz, THF-d_8) of Cs_2[**1**].

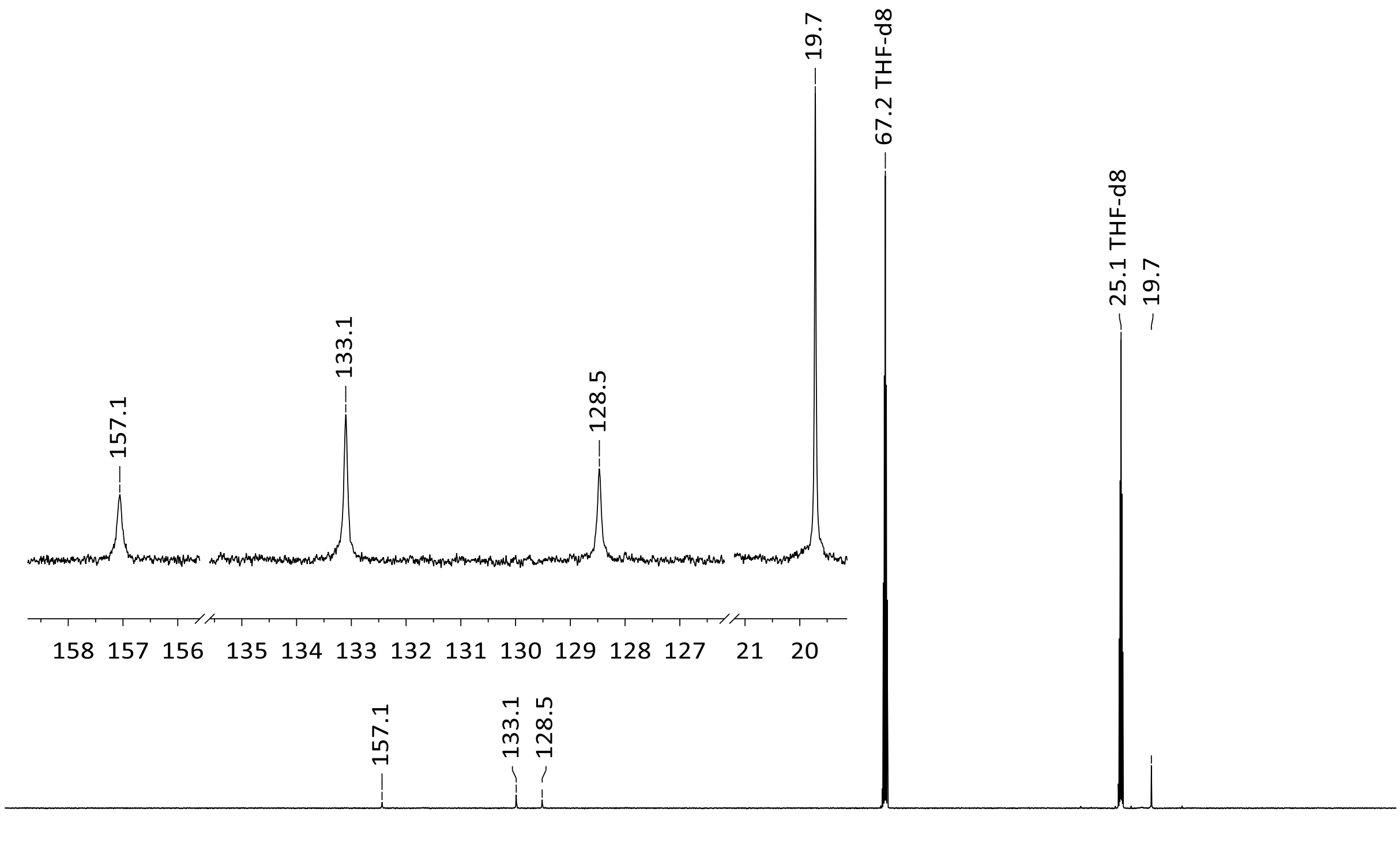

Figure S34. $^{13}C\{^{1}H\}$ NMR spectrum (125.8 MHz, THF-d_8) of Cs_2[**1**].

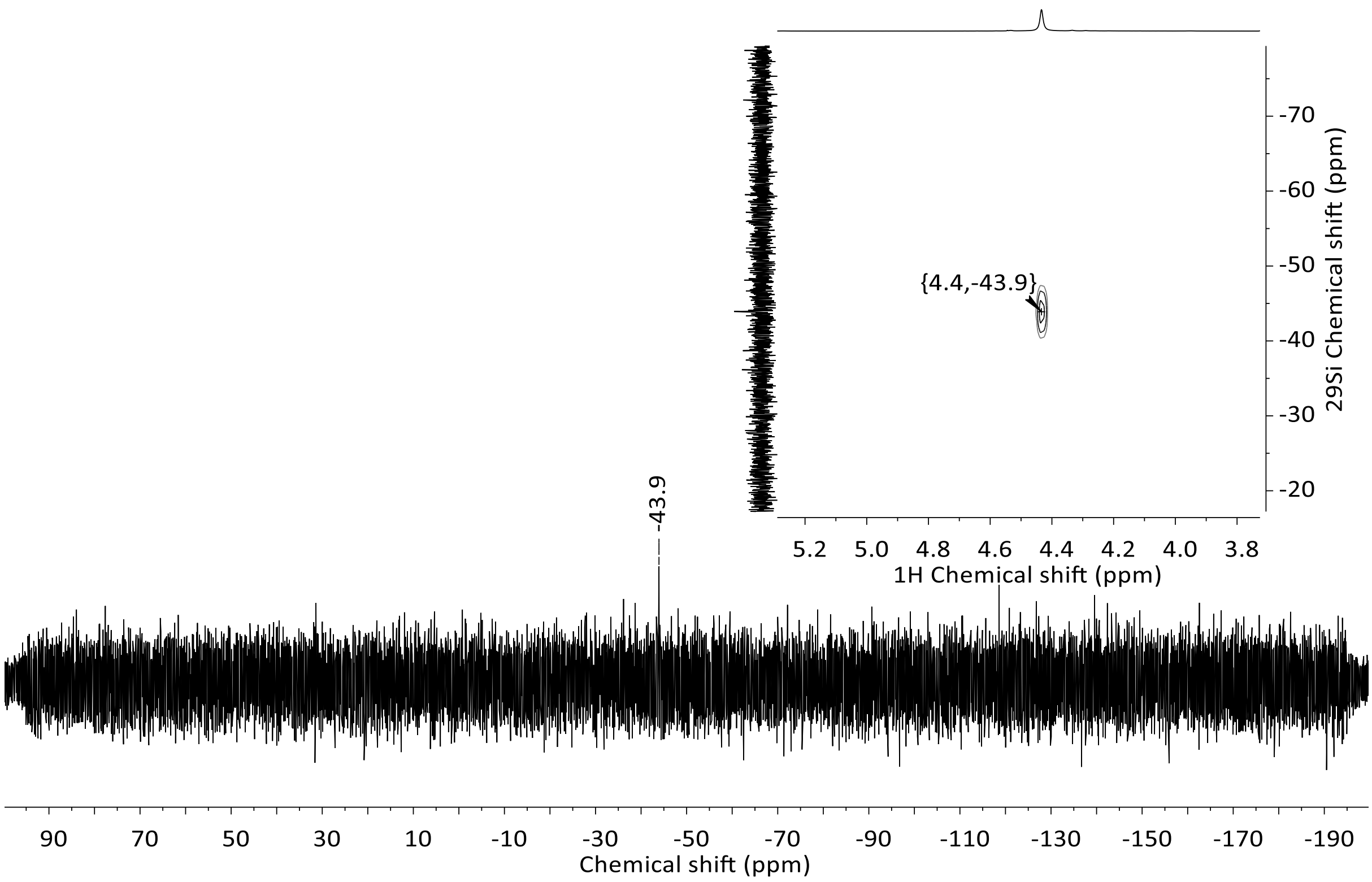

Figure S35. $^{29}Si\{^1H\}$ NMR spectrum (99.4 MHz, THF-d_8) of Cs_2[**1**], including an excerpt of the ^1H–^{29}Si HSQC NMR spectrum (^1H: 500.2 MHz, ^{29}Si: 99.4 MHz, THF-d_8).

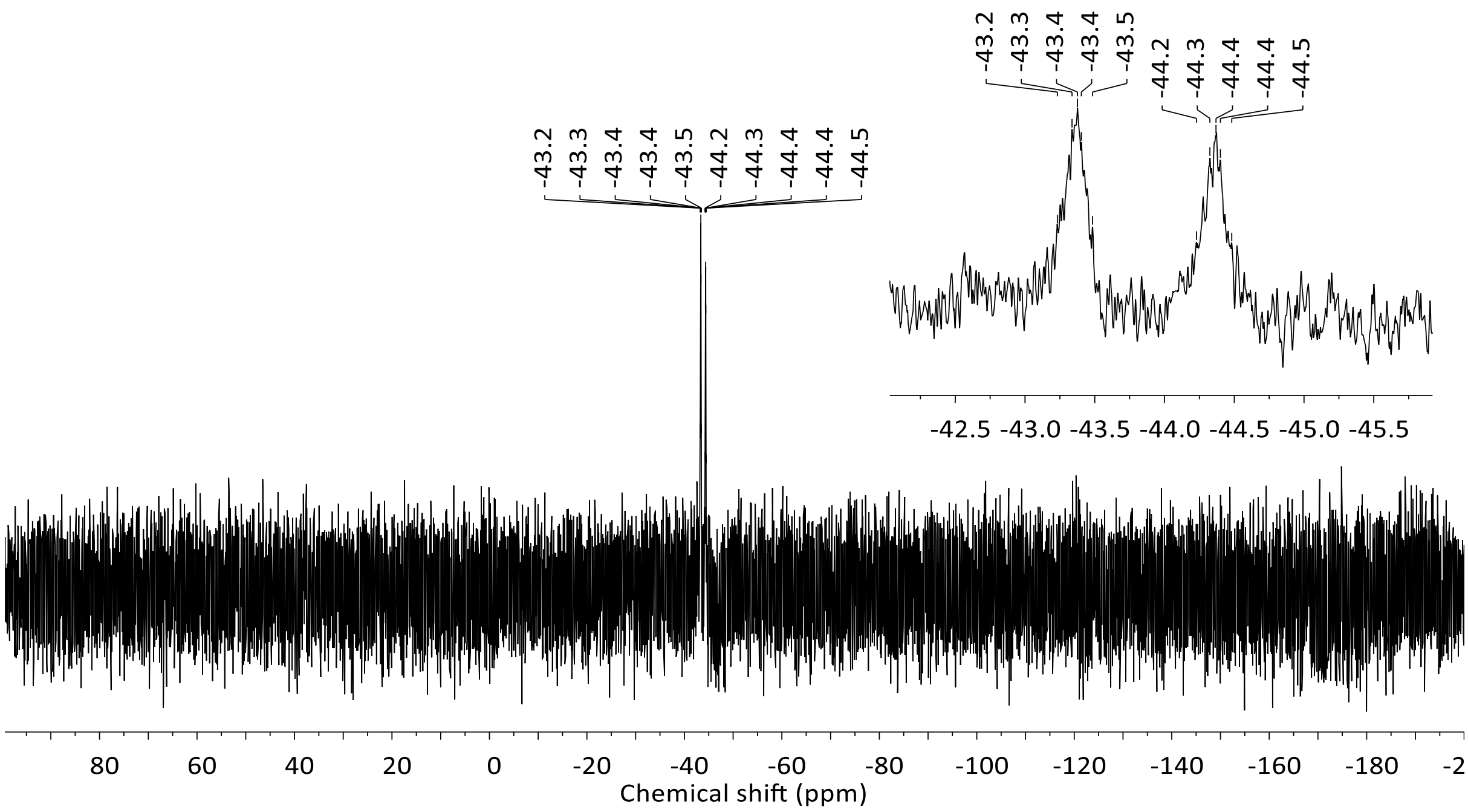

Figure S36. ^{29}Si NMR spectrum (99.4 MHz, THF-d_8) of Cs_2[**1**].

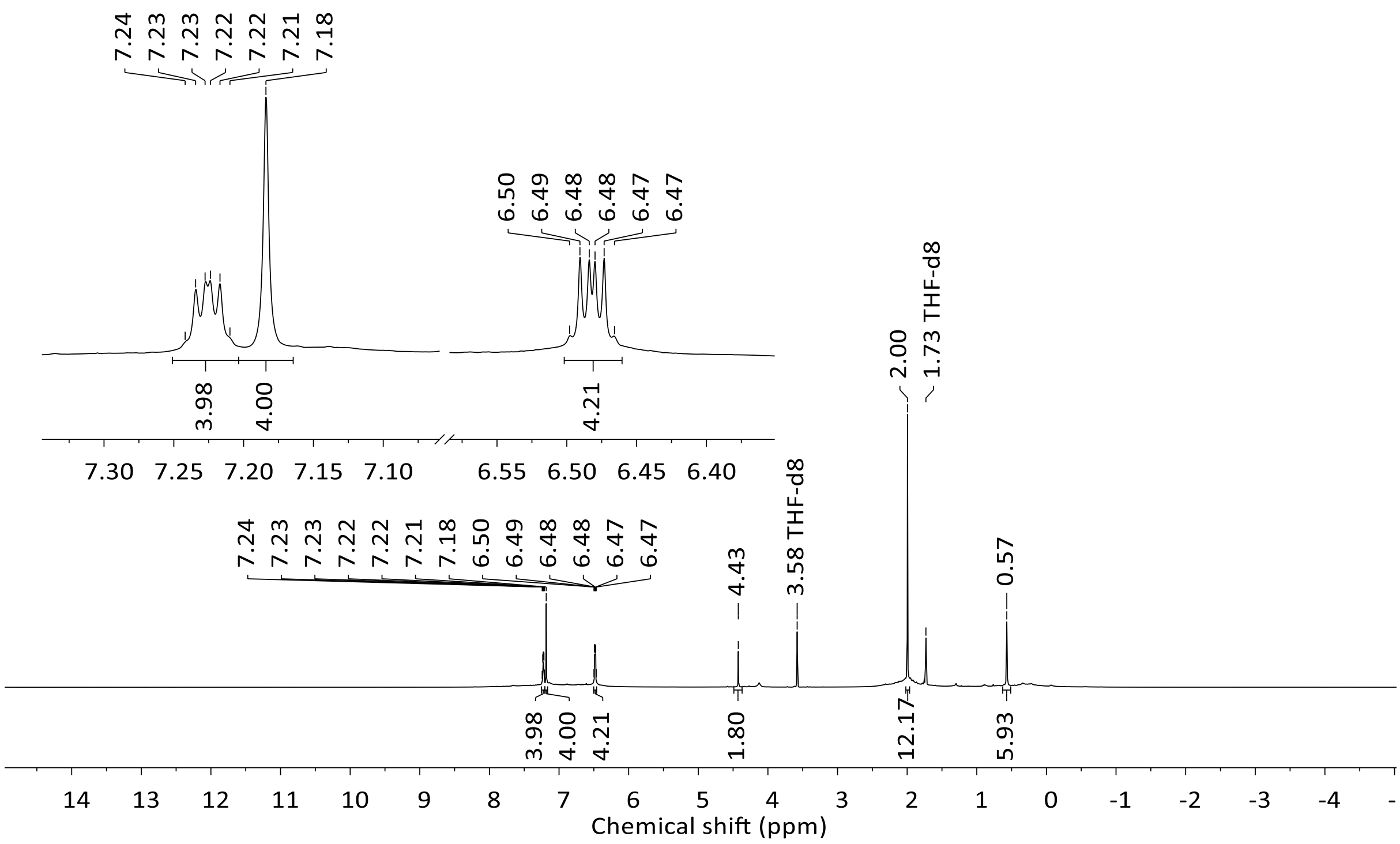

Figure S37. ^{1}H NMR spectrum (500.2 MHz, THF-d_8) of K_2[**10**].

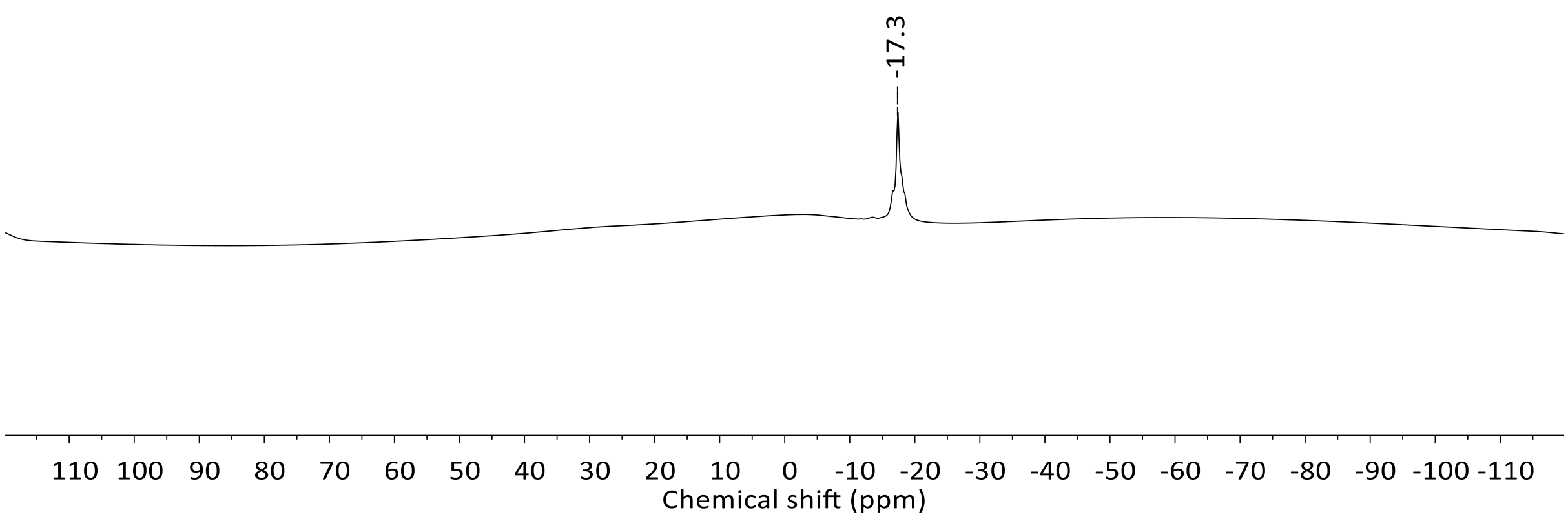

Figure S38. ^{11}B NMR spectrum (160.5 MHz, THF-d_8) of K_2[**10**].

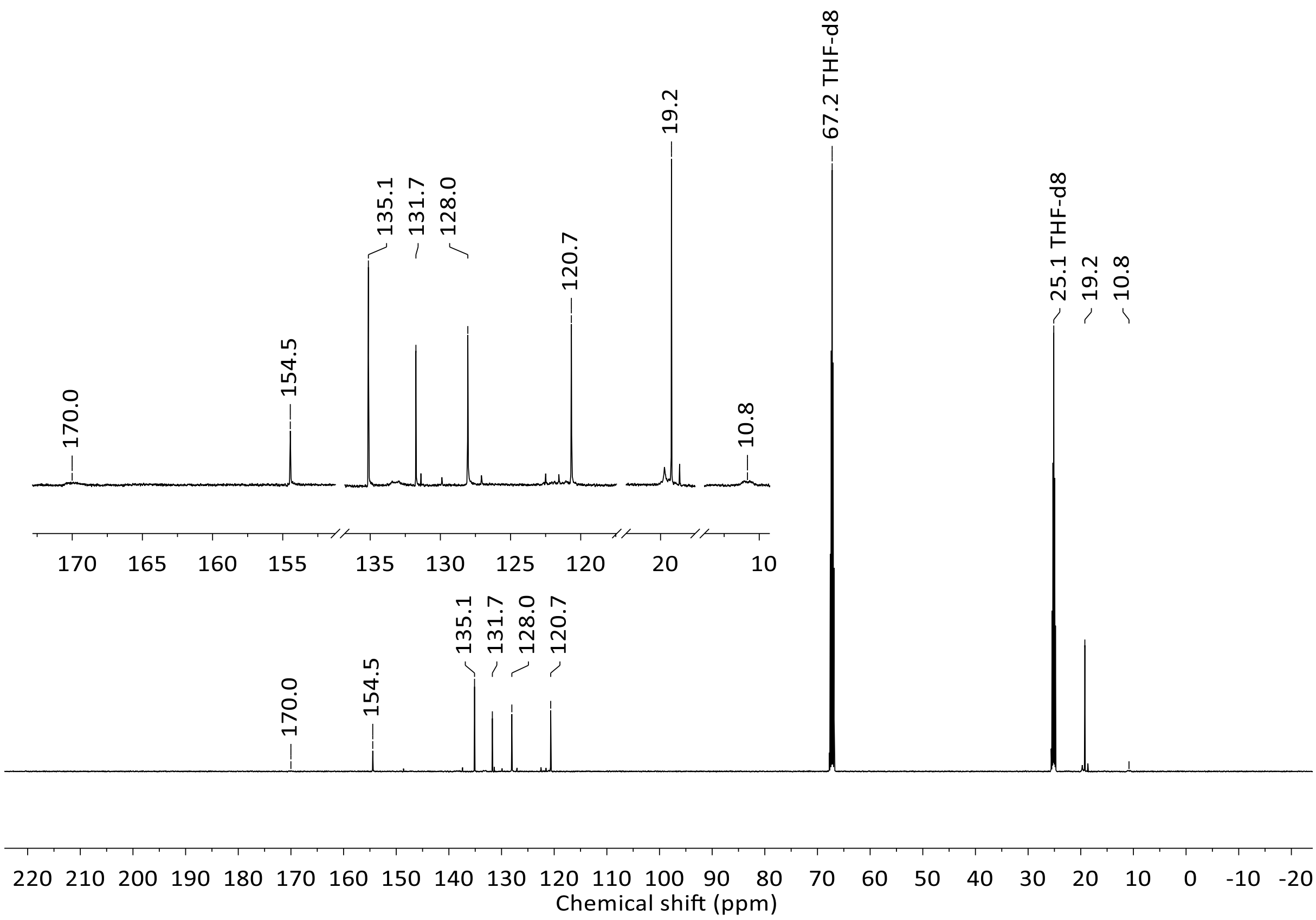

Figure S39. $^{13}C\{^1H\}$ NMR spectrum (125.8 MHz, THF-d_8) of K_2[**10**].

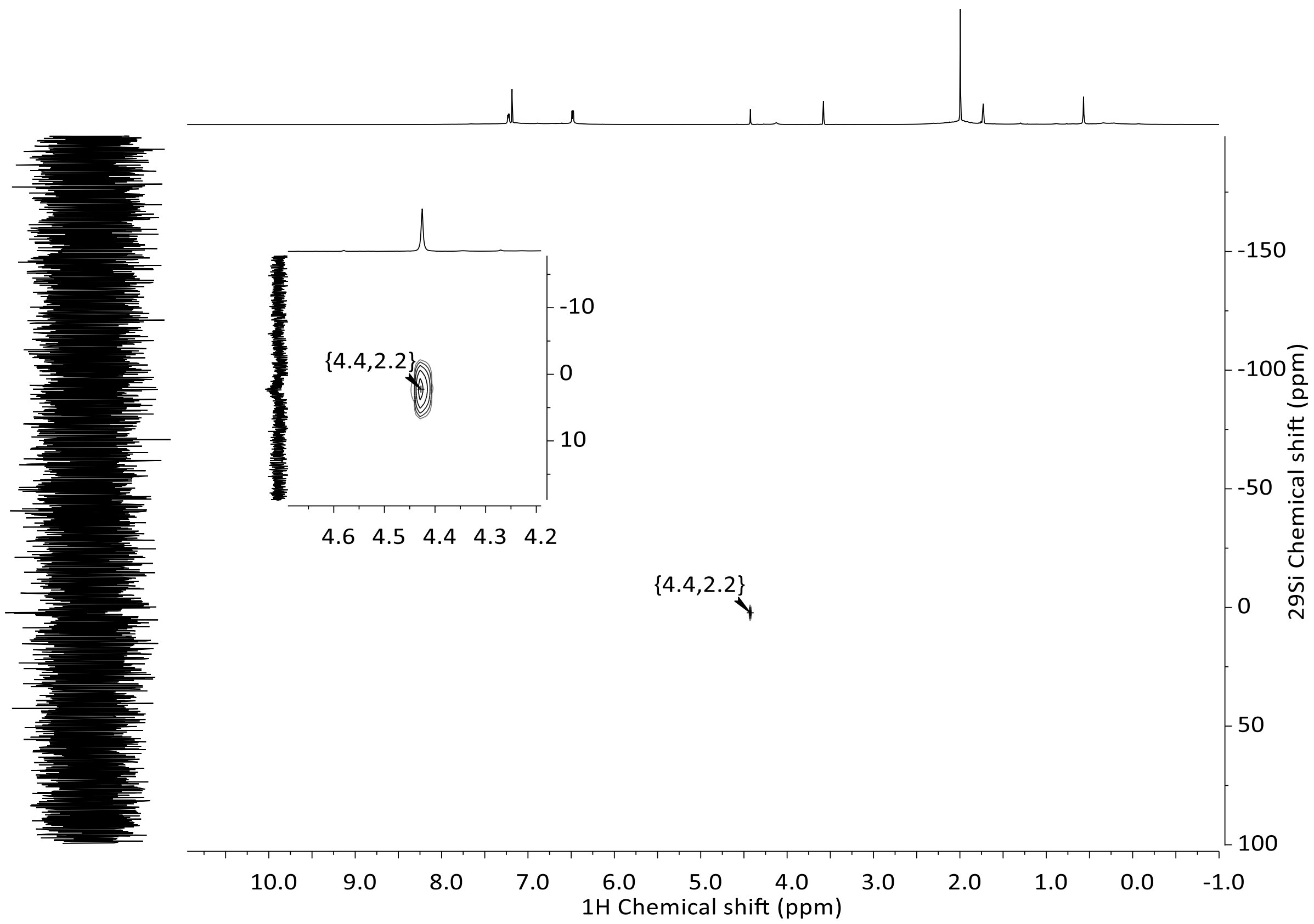

Figure S40. ^1H–^{29}Si HSQC NMR spectrum (^1H: 500.2 MHz, ^{29}Si: 99.4 MHz, THF-d_8) of K_2[**10**].

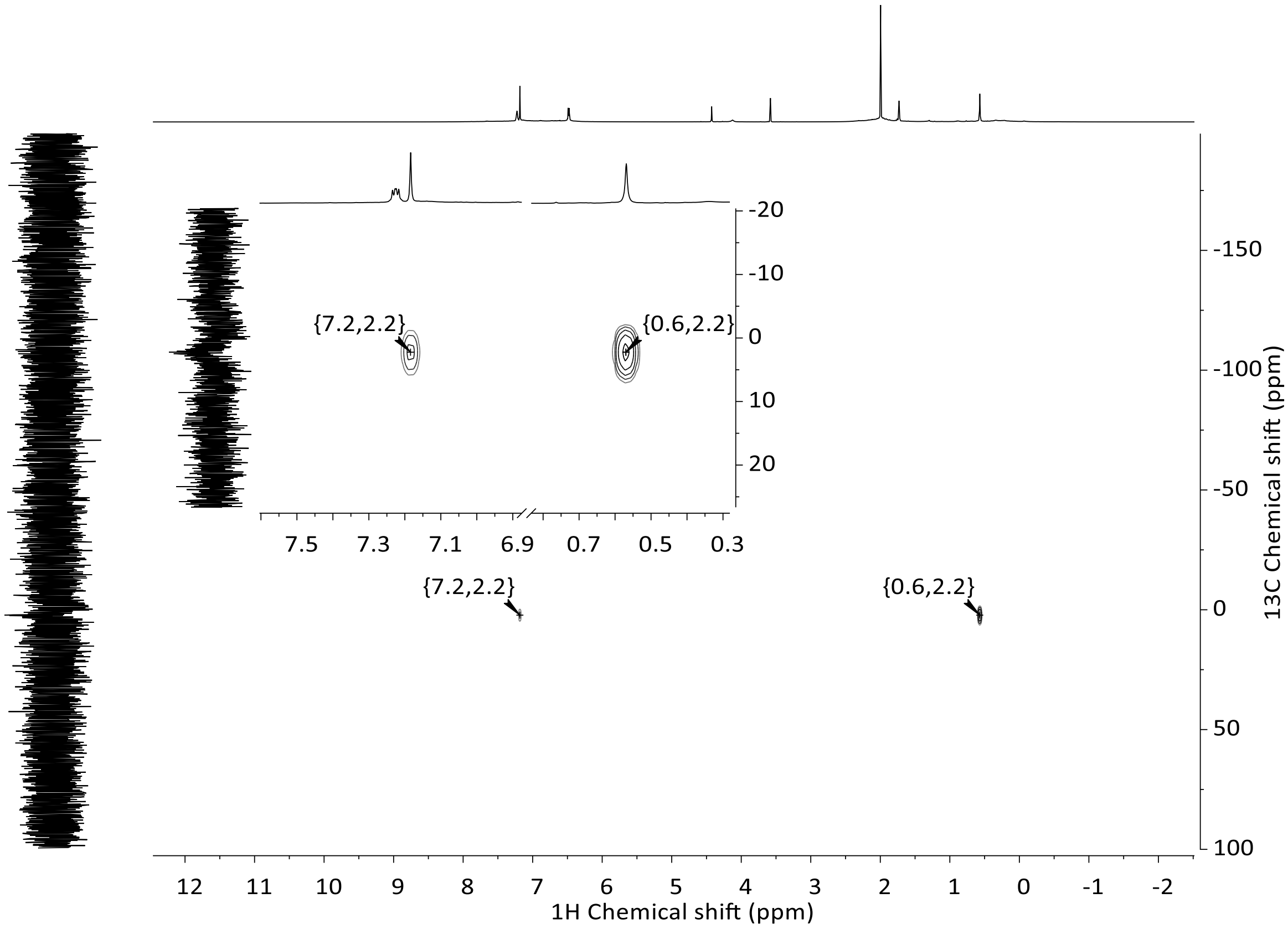

Figure S41. ^{1}H–^{29}Si HMBC NMR spectrum (^{1}H: 500.2 MHz, ^{29}Si: 99.4 MHz, THF-d_8) of K_2[**10**].

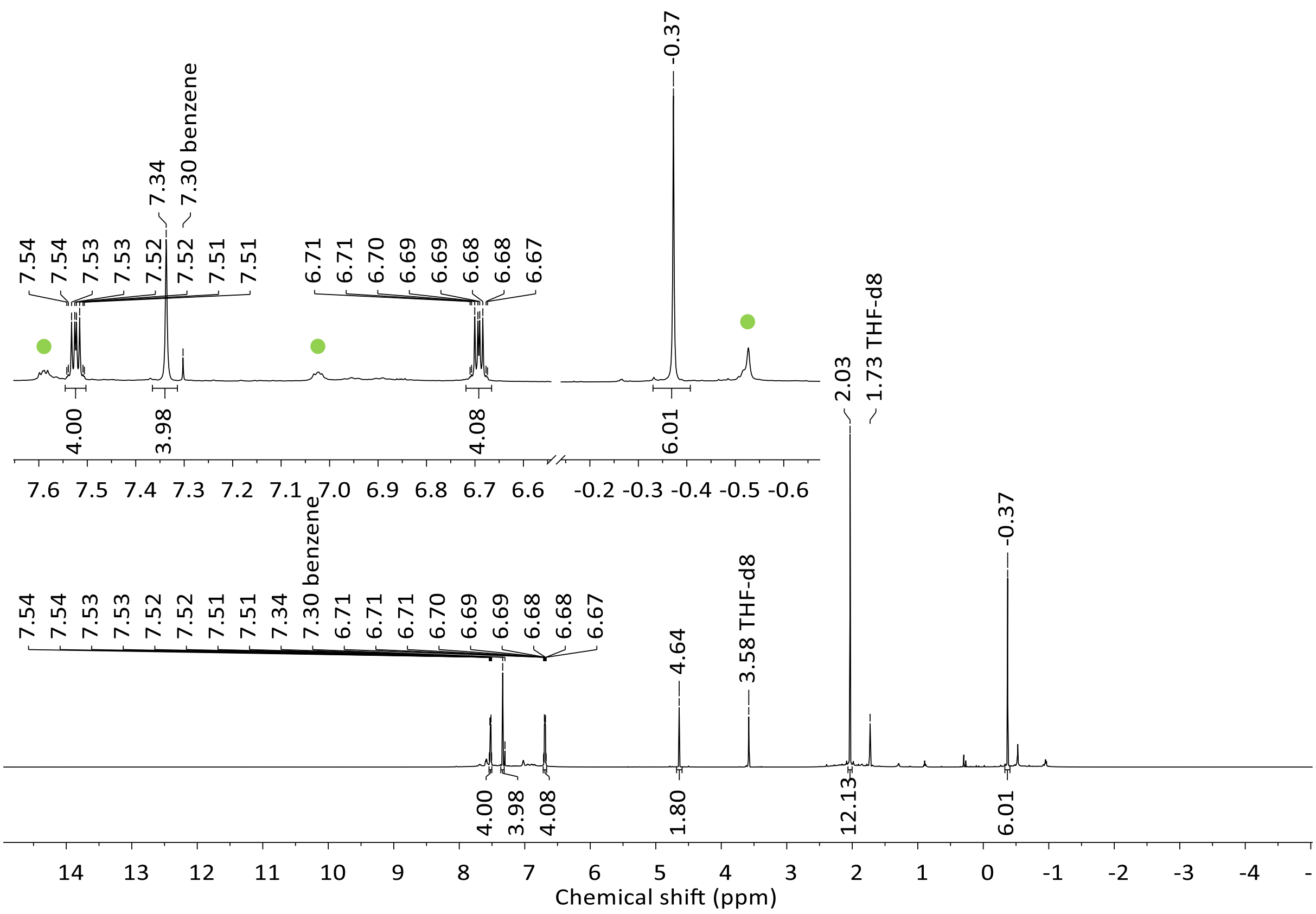

Figure S42. ^{1}H NMR spectrum (500.2 MHz, THF-d_8) of K_2[**11**]. Marked contaminant: **9** (green dots).

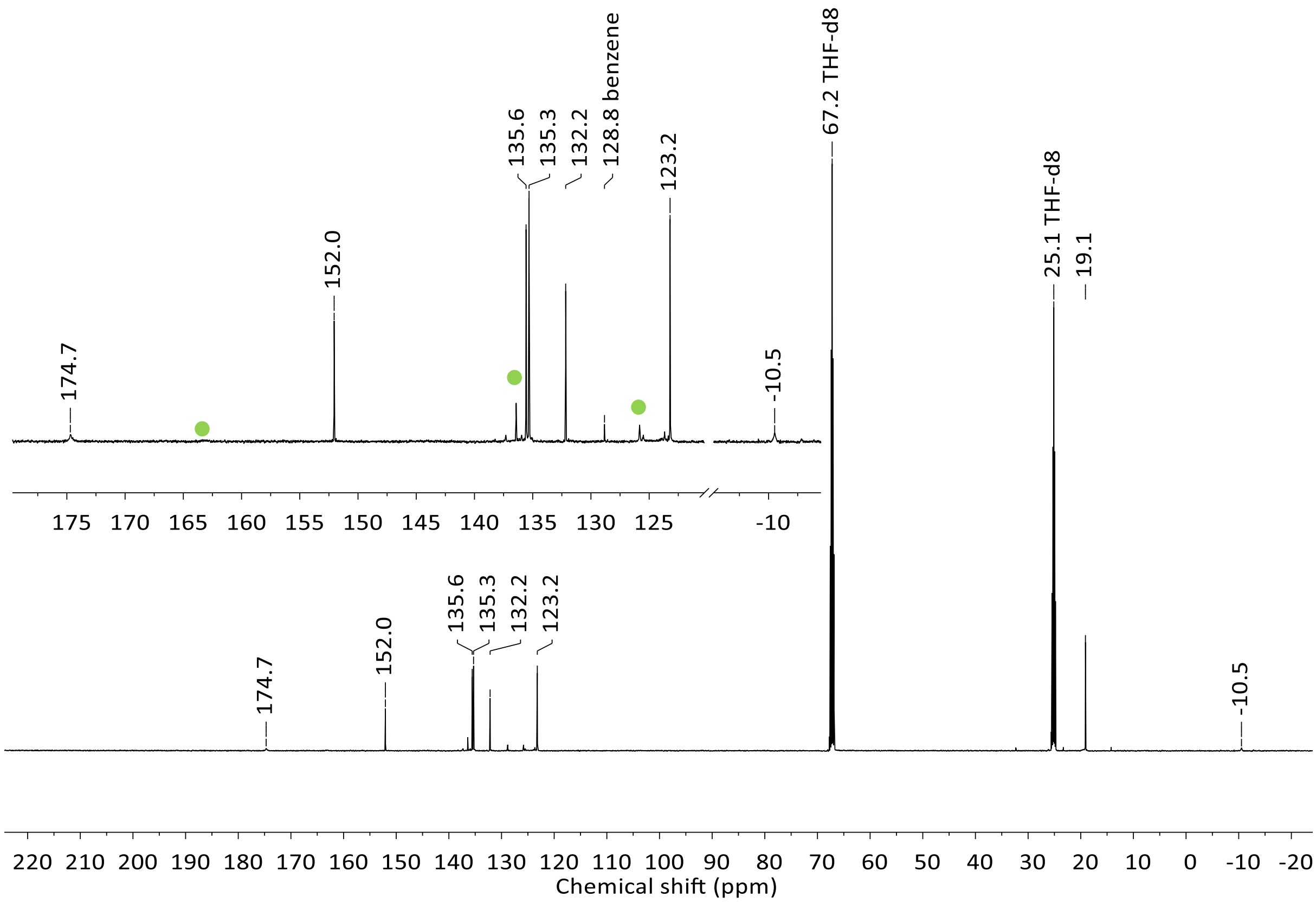

Figure S43. $^{13}C\{^{1}H\}$ NMR spectrum (125.8 MHz, THF-d_8) of K_2[**11**]. Marked contaminant: **9** (green dots).

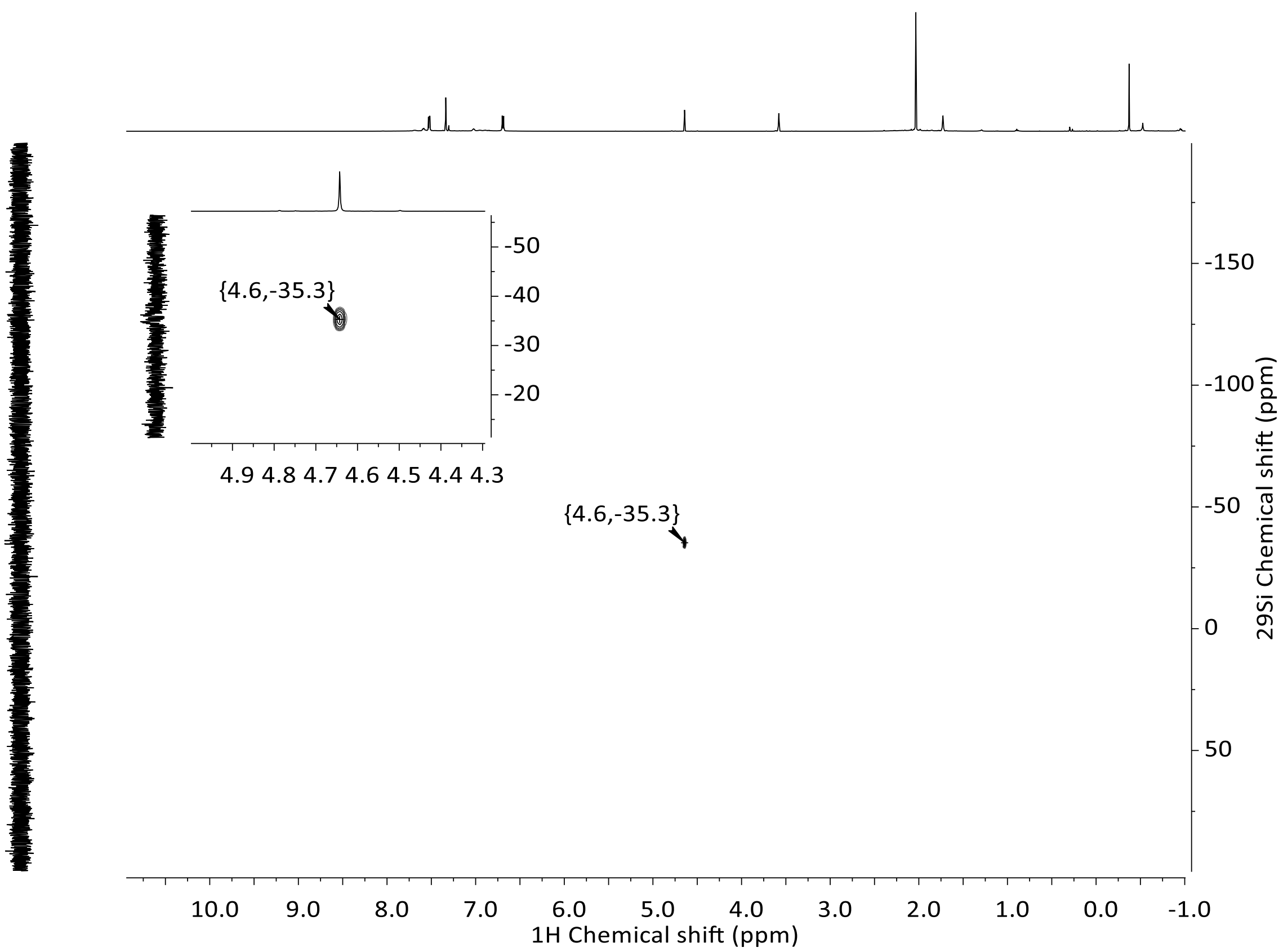

Figure S44. ^{1}H–^{29}Si HSQC NMR spectrum (^{1}H: 500.2 MHz, ^{29}Si: 99.4 MHz, THF-d_8) of K_2[**11**].

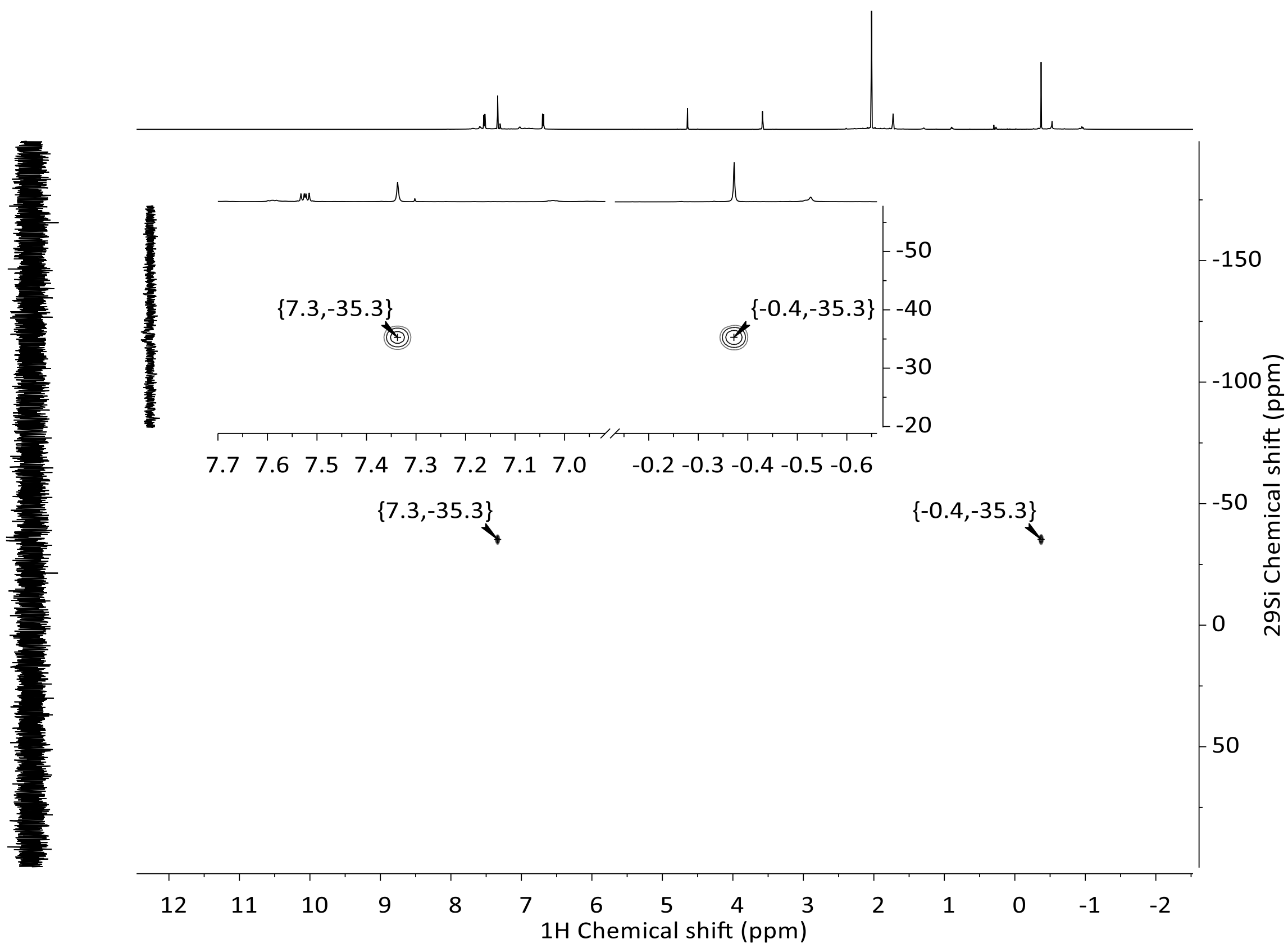

Figure S45. ^{1}H–^{29}Si HMBC NMR spectrum (^{1}H: 500.2 MHz, ^{29}Si: 99.4 MHz, THF-d_8) of K_2[**11**].

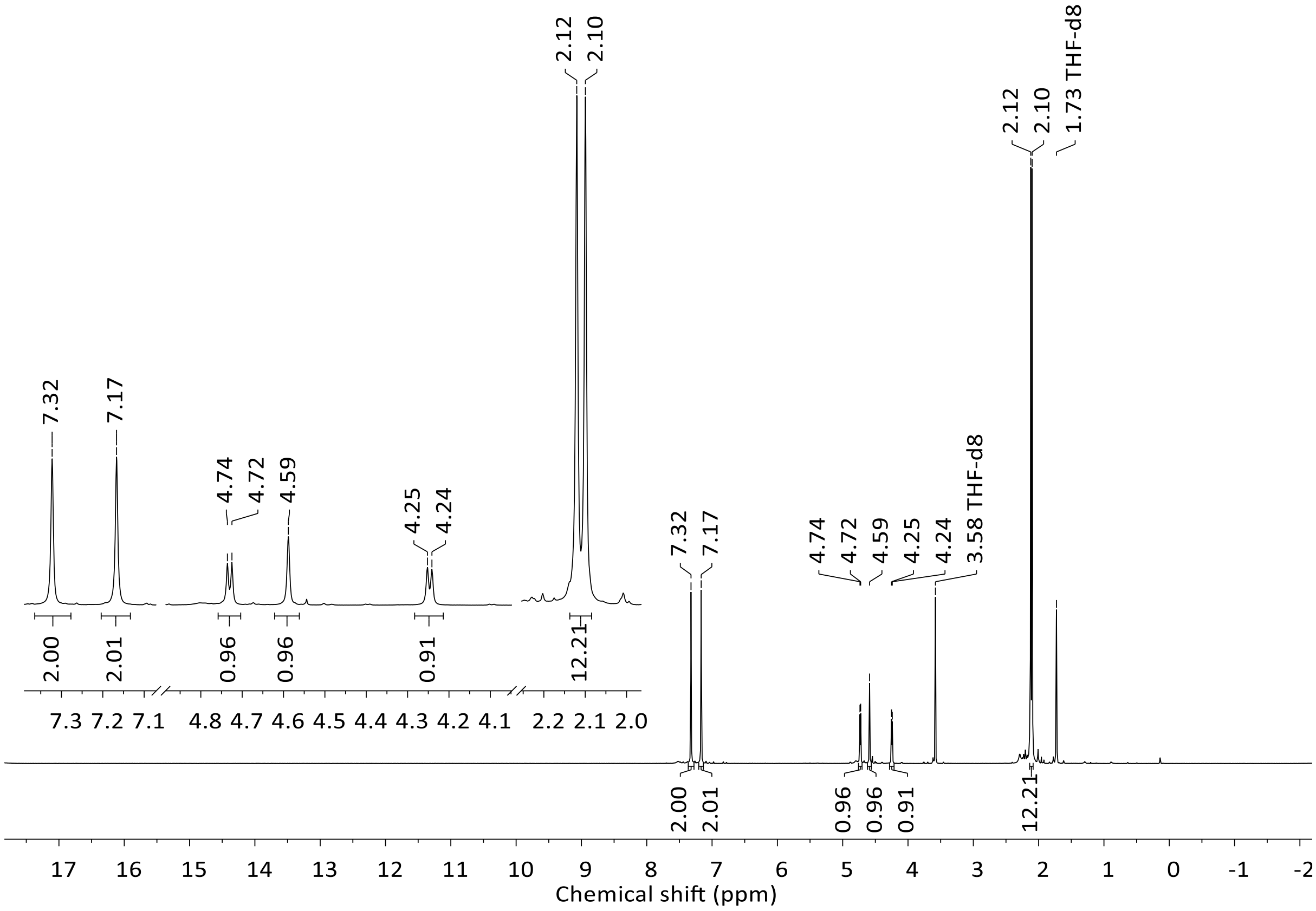

Figure S46. ^{1}H NMR spectrum (500.2 MHz, THF-d_8) of K[**1^H**].

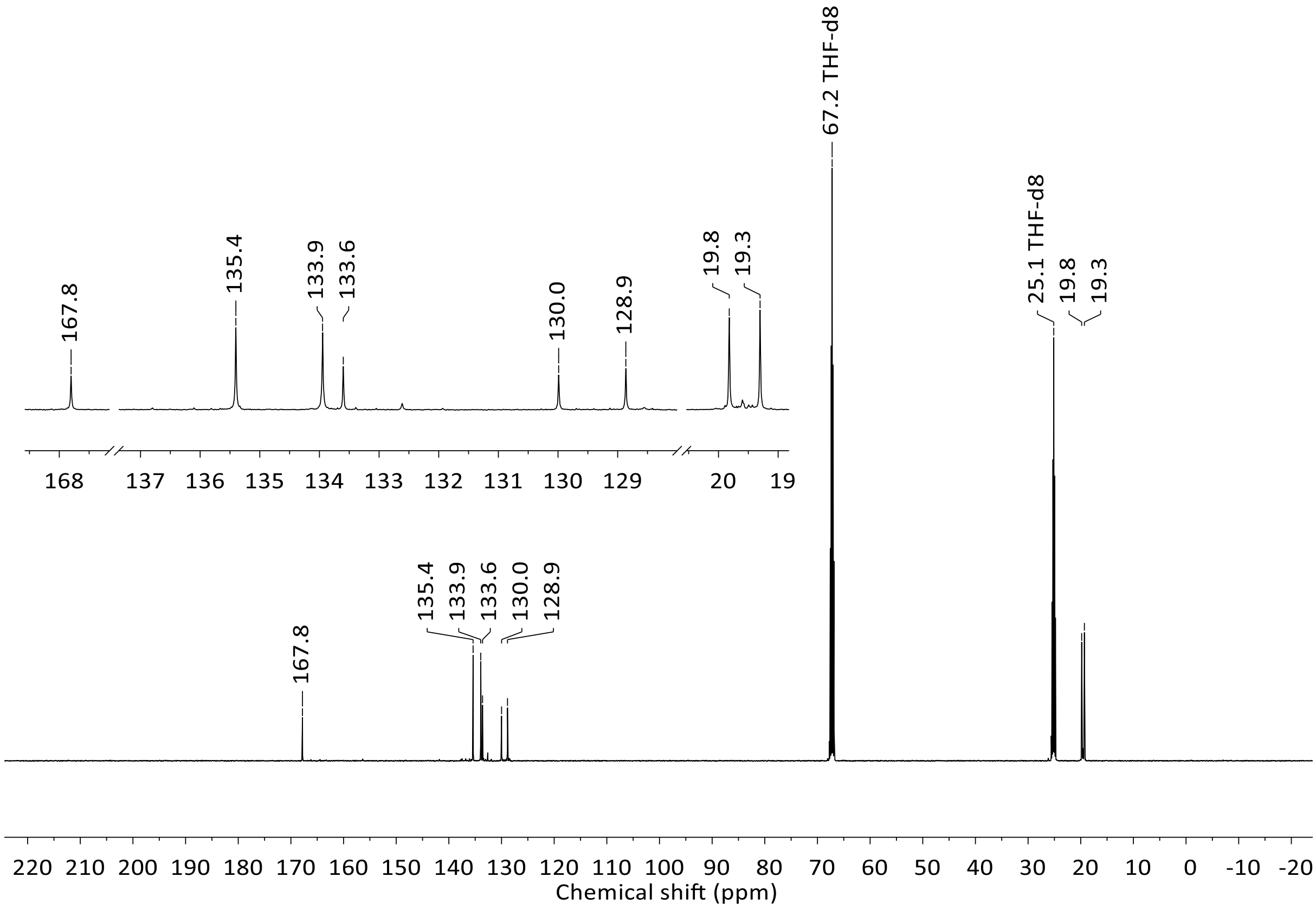

Figure S47. $^{13}C\{^{1}H\}$ NMR spectrum (125.8 MHz, THF-d_8) of K[**1$^{\mathbf{H}}$**].

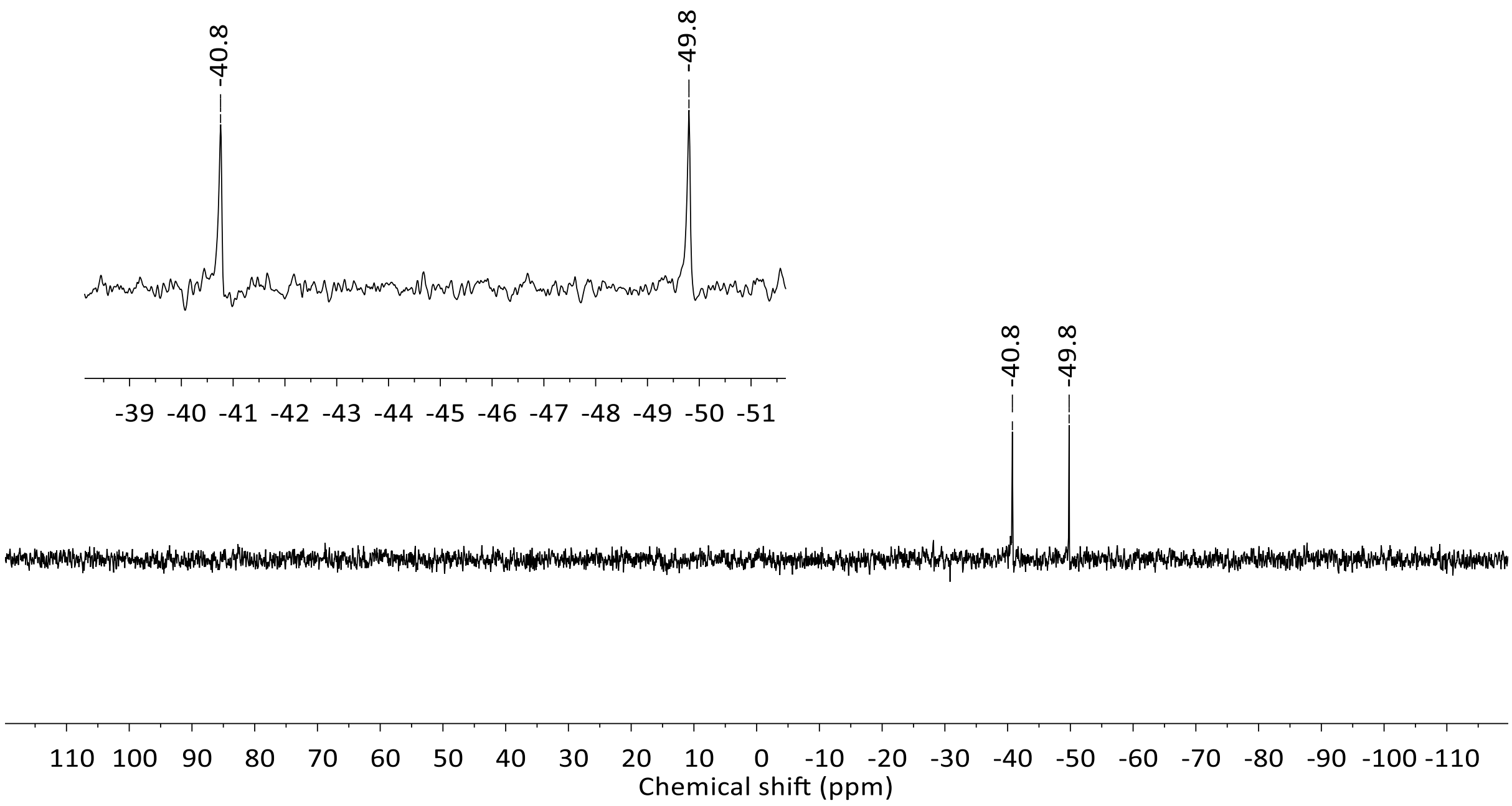

Figure S48. $^{29}Si\{^{1}H\}$ NMR spectrum (99.4 MHz, THF-d_8) of K[**1$^{\mathbf{H}}$**].

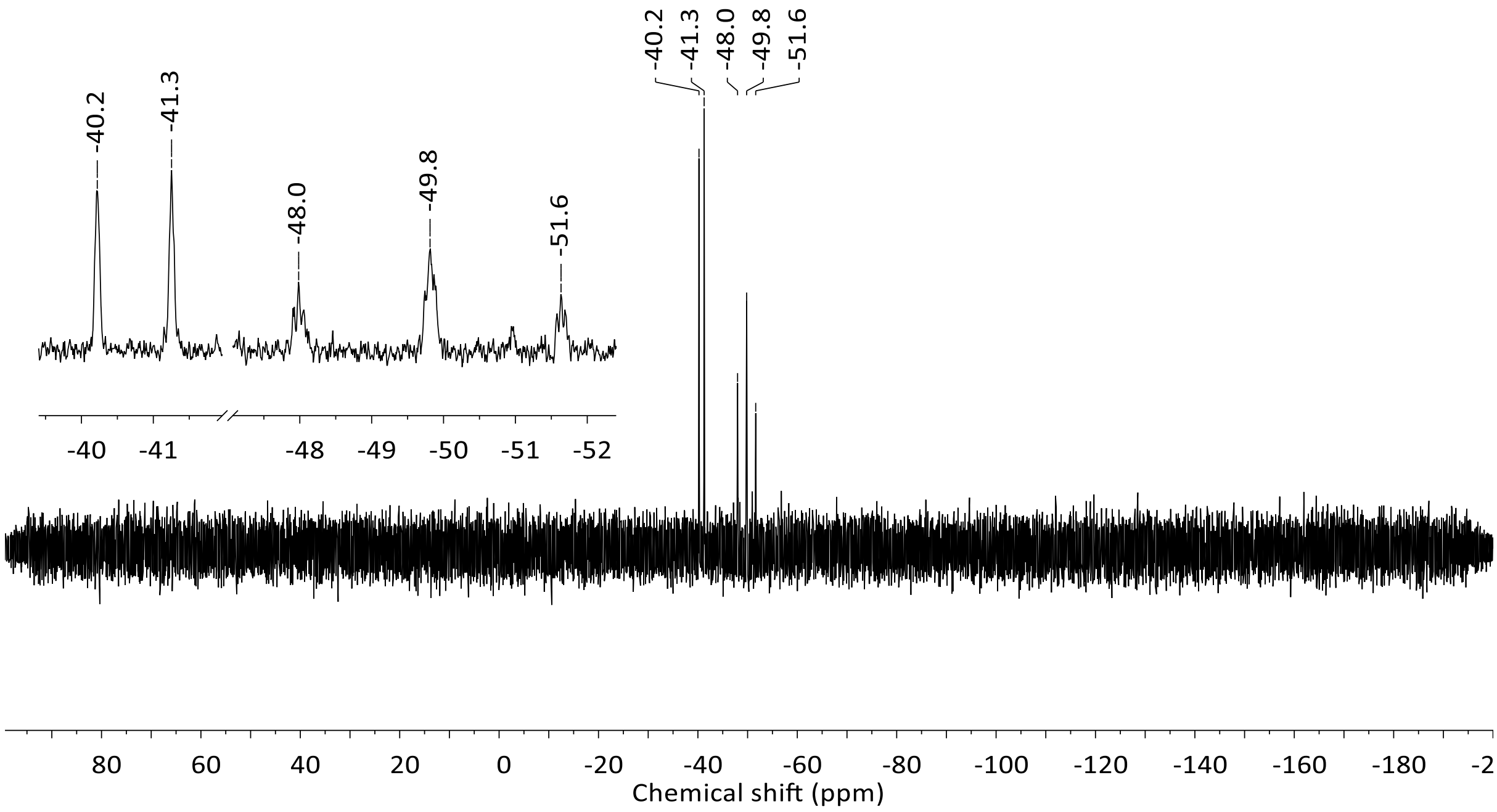

Figure S49. ^{29}Si NMR spectrum (99.4 MHz, THF-d_8) of K[**1^{H}**].

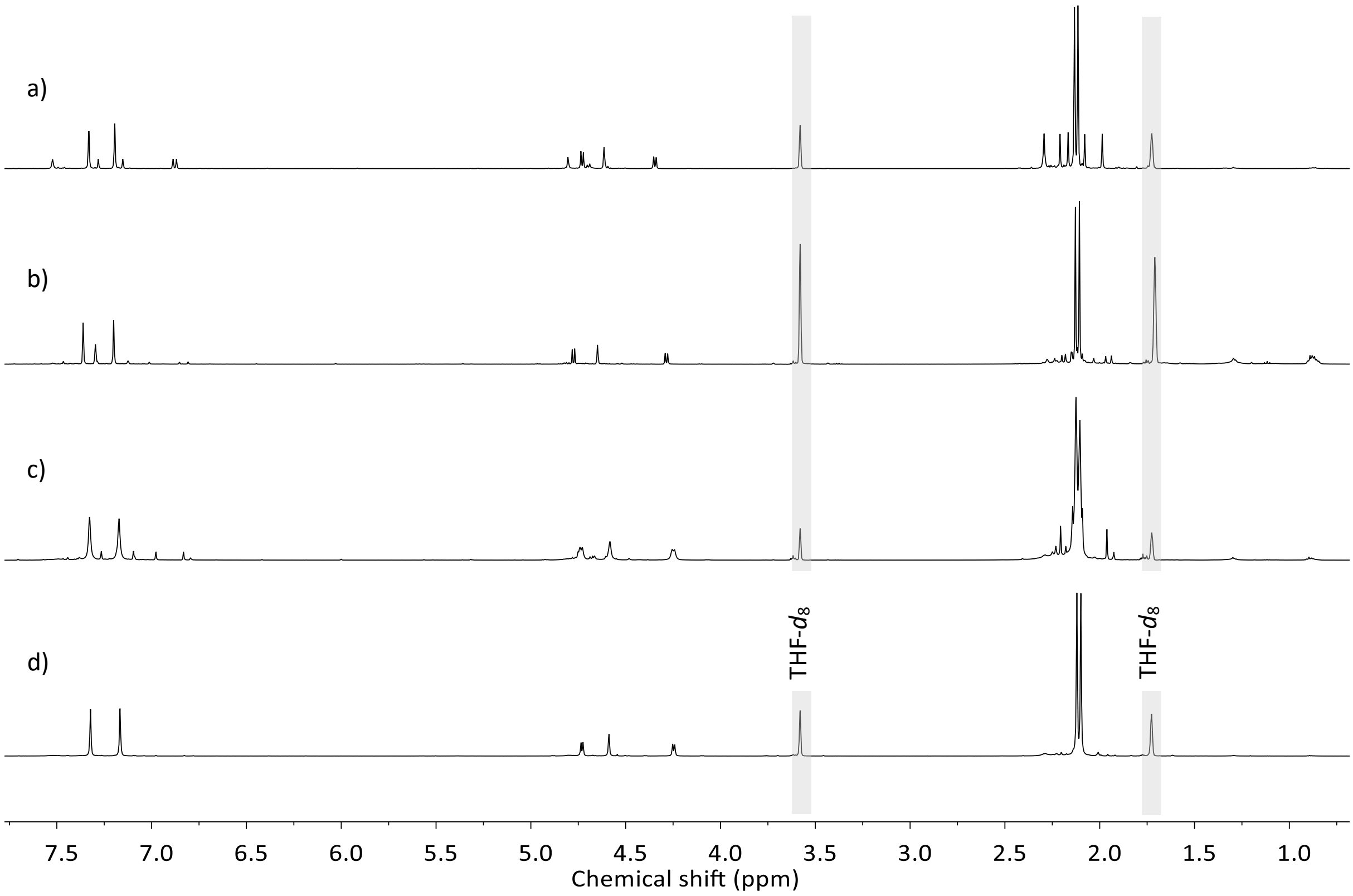

Figure S50. Stacked ^{1}H NMR spectra (500.2 MHz, THF-d_8) of M[**1^{H}**] prepared by different methods (a–d; M = Na or K), illustrating variations in sample purity. a) Treatment of **1^{2H}** with excess Na in THF-d_8 (1 d). b) THF-d_8 extract of the filter cake obtained after treatment of **1^{2H}** with excess K in C_6D_6 (1 d). c) Addition of **1^{2H}** to a freshly prepared THF-d_8 solution of K_2[**1**]. d) Treatment of **1^{2H}** with KC_8 (2 equiv.) in THF-d_8.

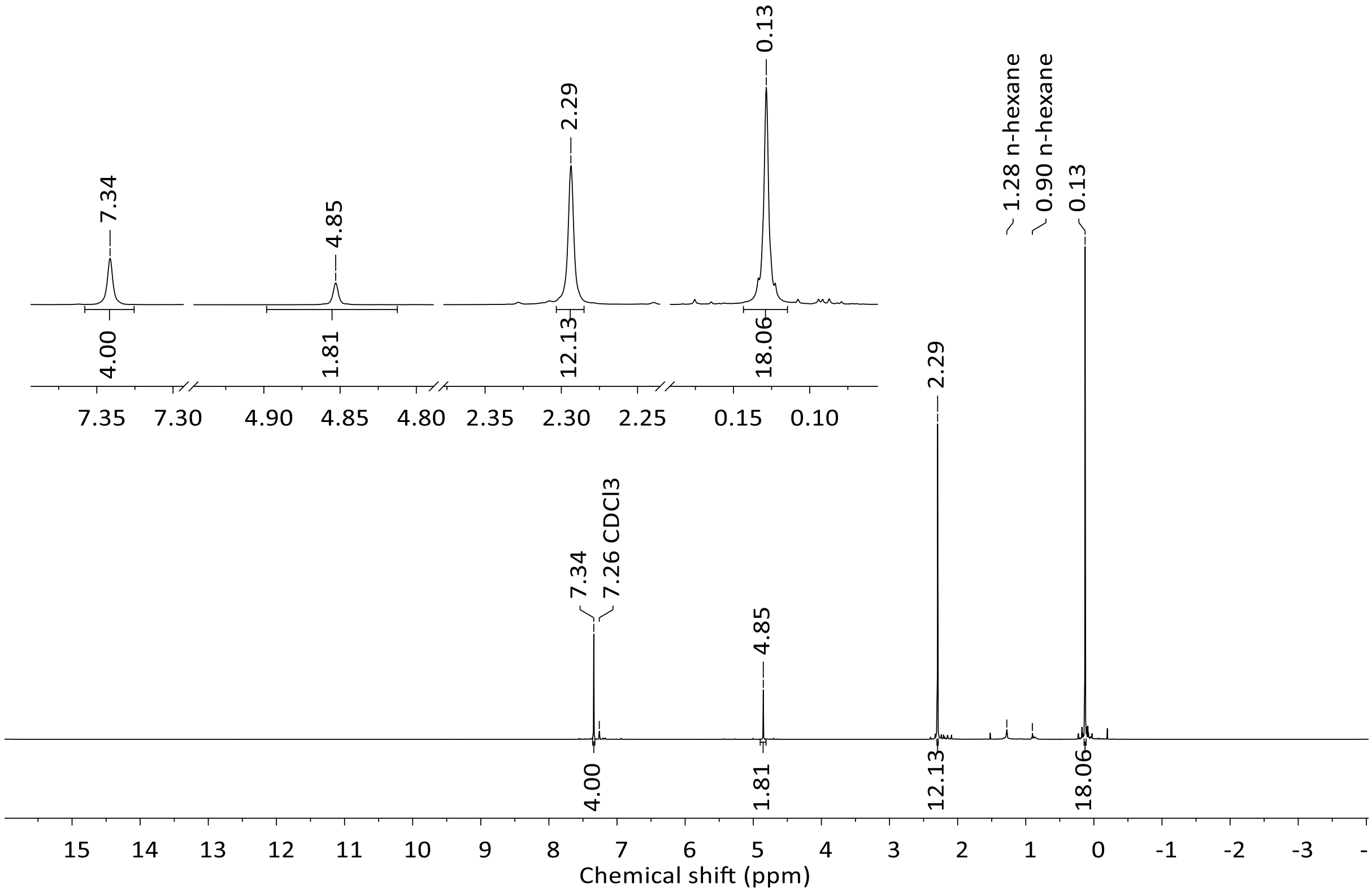

Figure S51. ^{1}H NMR spectrum (500.2 MHz, $CDCl_3$) of *cis*-**2**.

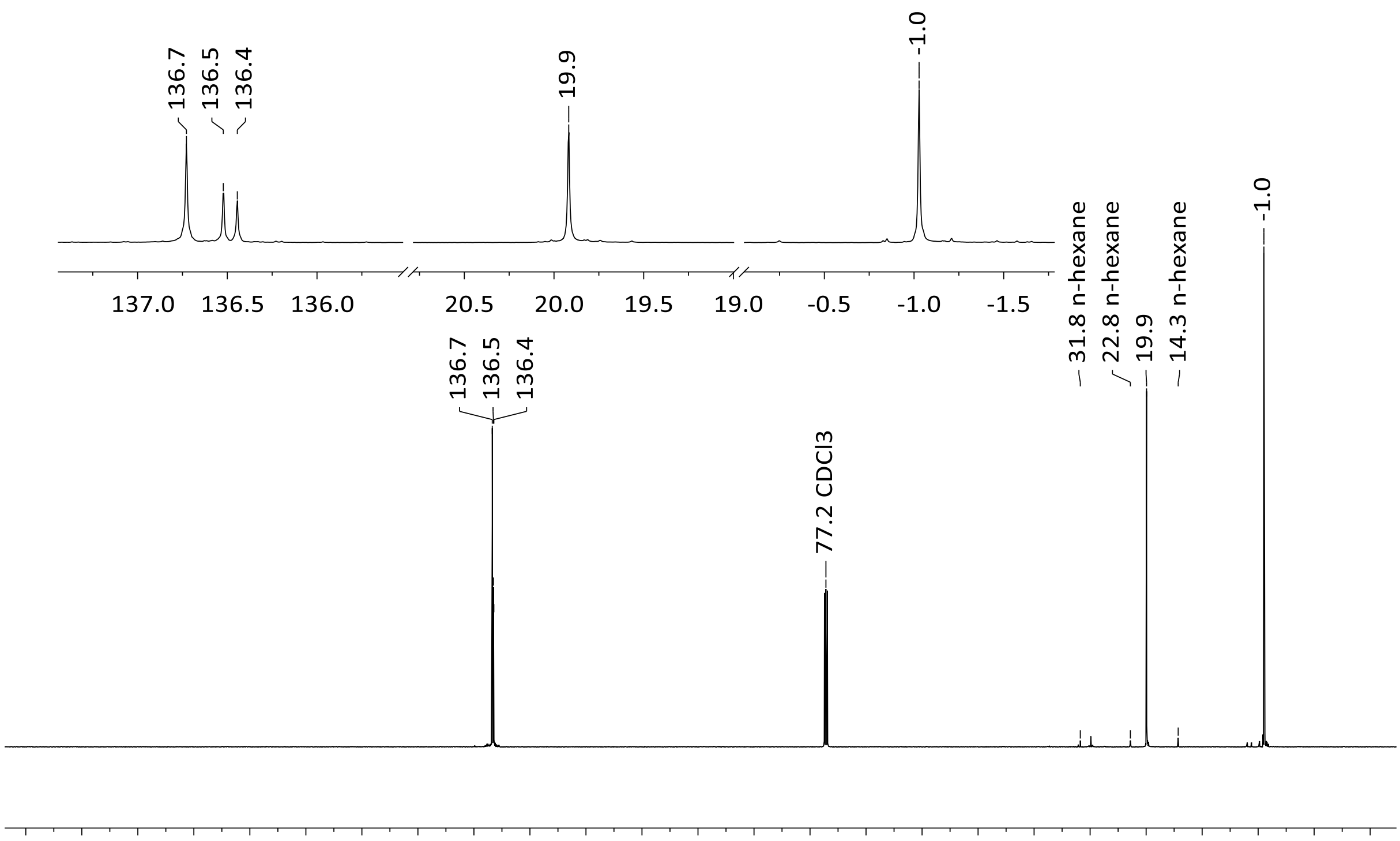

Figure S52. $^{13}C\{^{1}H\}$ NMR spectrum (125.8 MHz, $CDCl_3$) of *cis*-**2**.

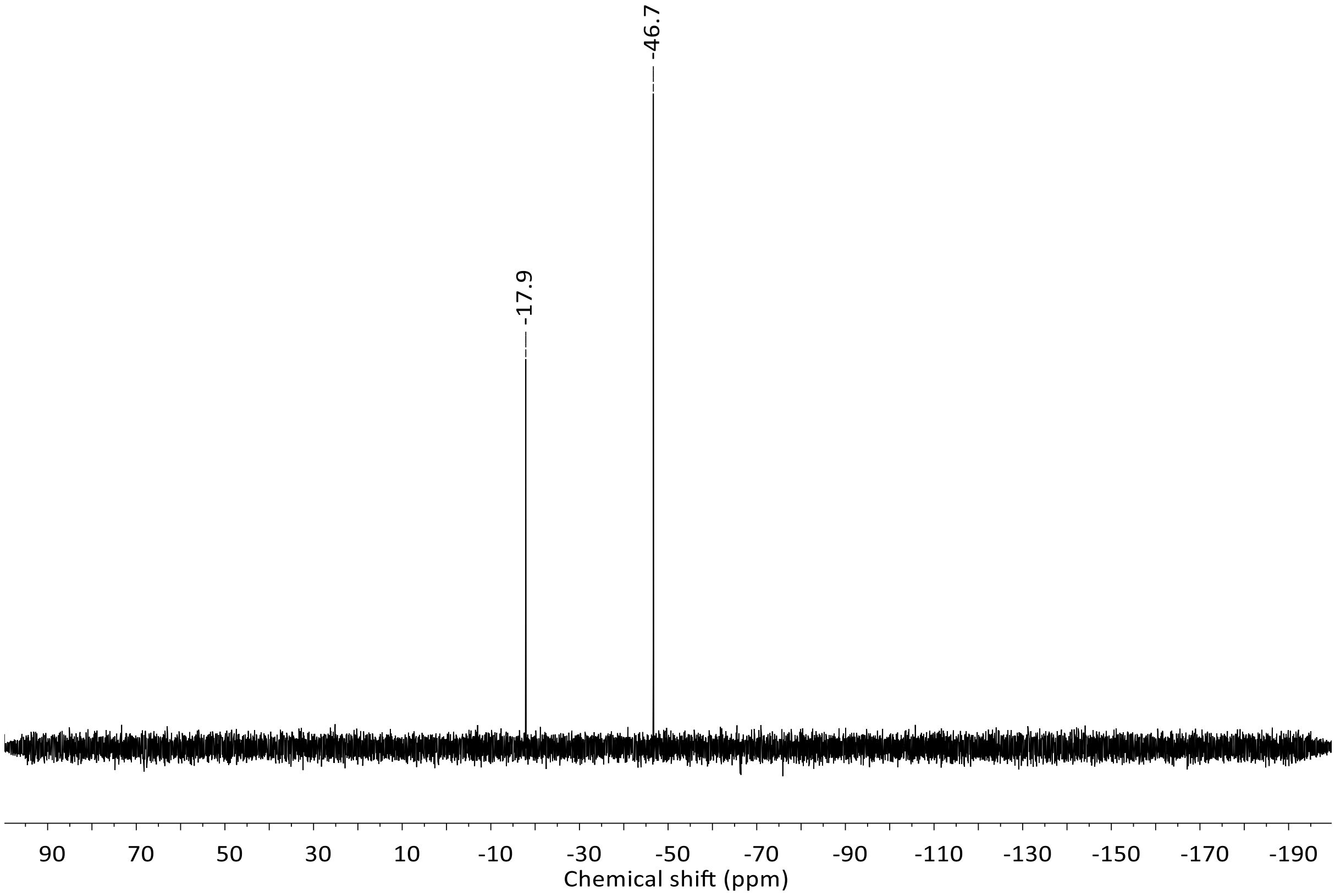

Figure S53. $^{29}Si\{^{1}H\}$ NMR spectrum (99.4 MHz, $CDCl_3$) of *cis*-**2**.

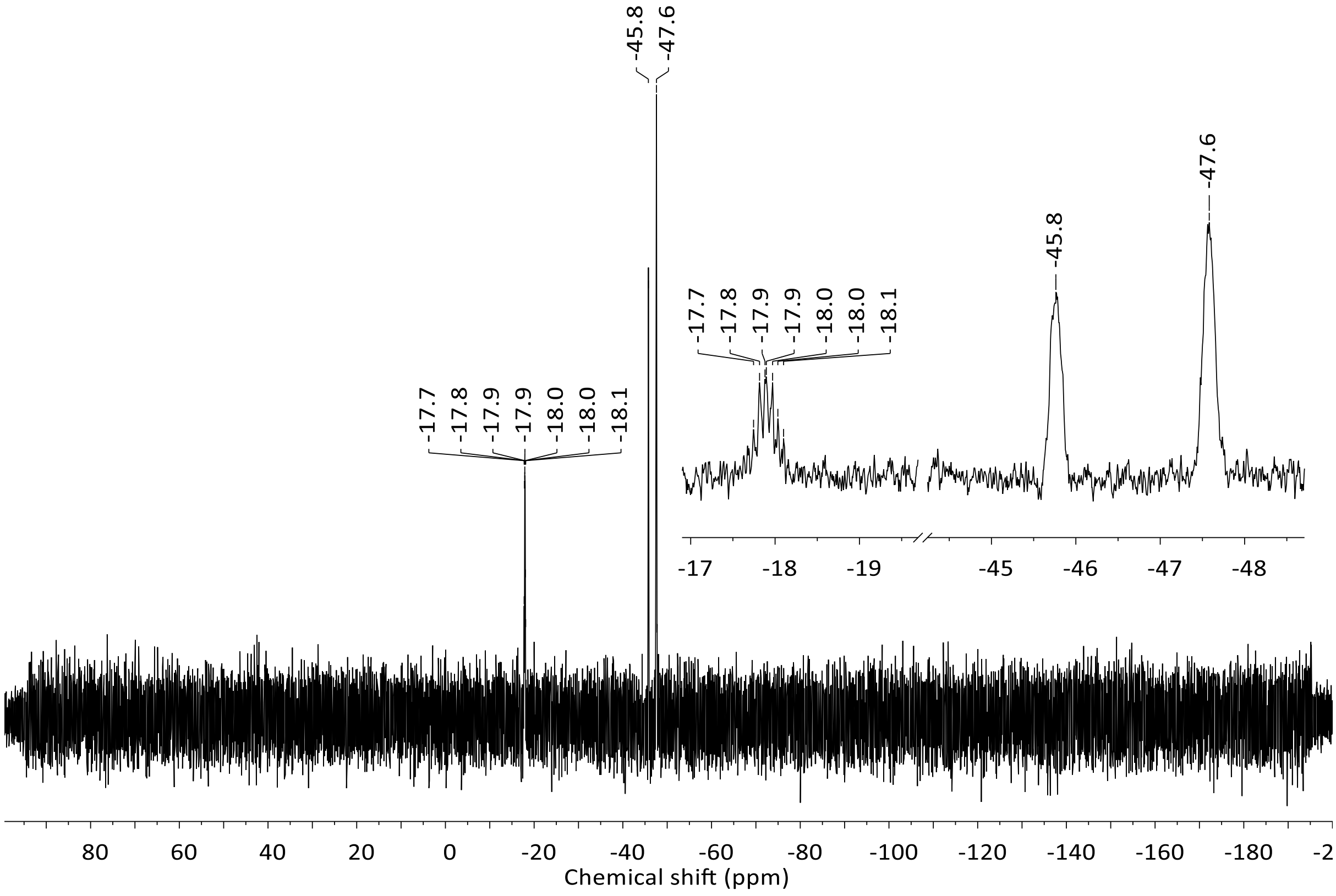

Figure S54. ^{29}Si NMR spectrum (99.4 MHz, $CDCl_3$) of *cis*-**2**.

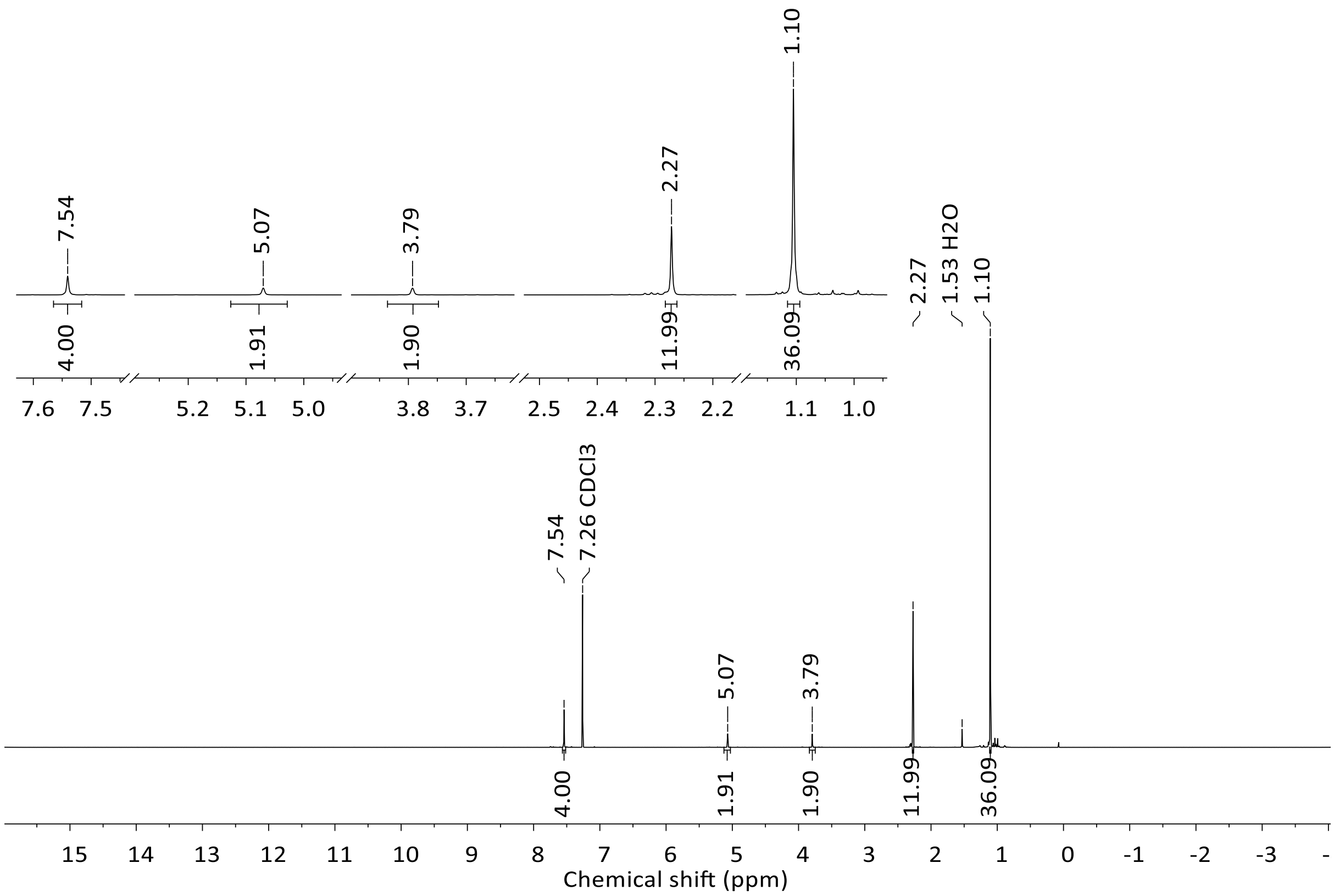

Figure S55. ^{1}H NMR spectrum (500.2 MHz, $CDCl_3$) of *cis*-**3**.

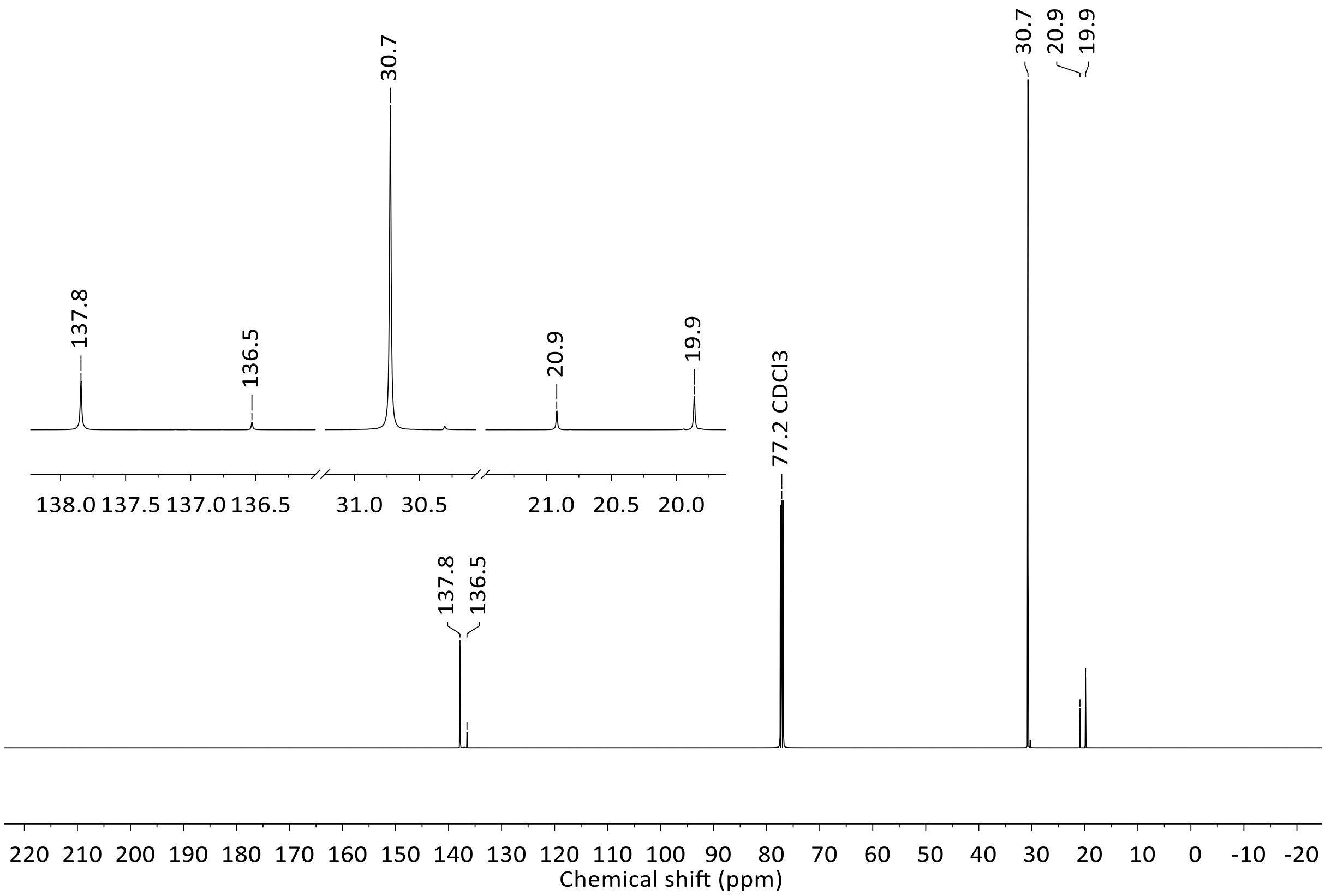

Figure S56. $^{13}C\{^{1}H\}$ NMR spectrum (125.8 MHz, $CDCl_3$) of *cis*-**3**.

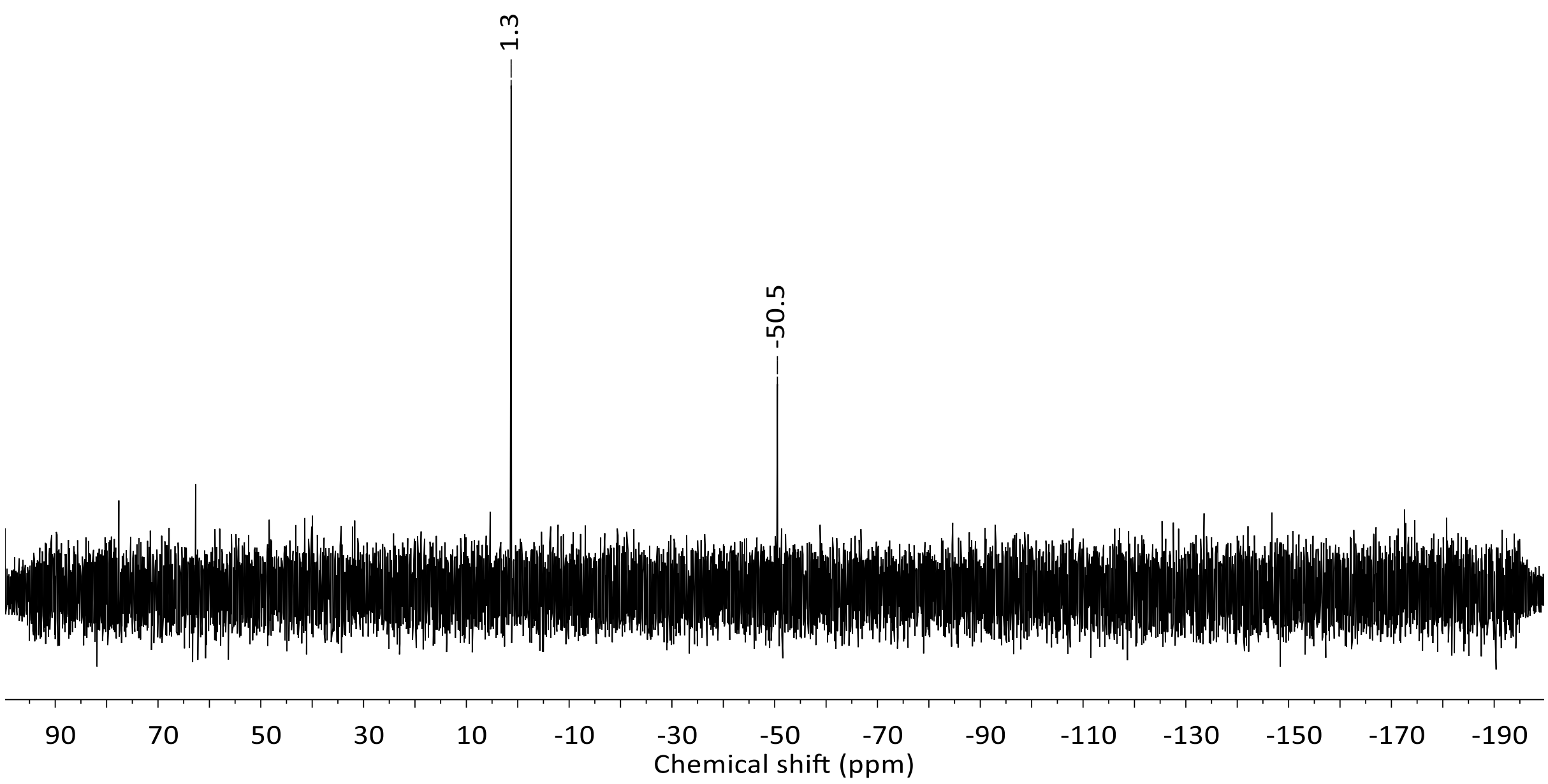

Figure S57. $^{29}Si\{^{1}H\}$ NMR spectrum (99.4 MHz, $CDCl_3$) of *cis*-**3**.

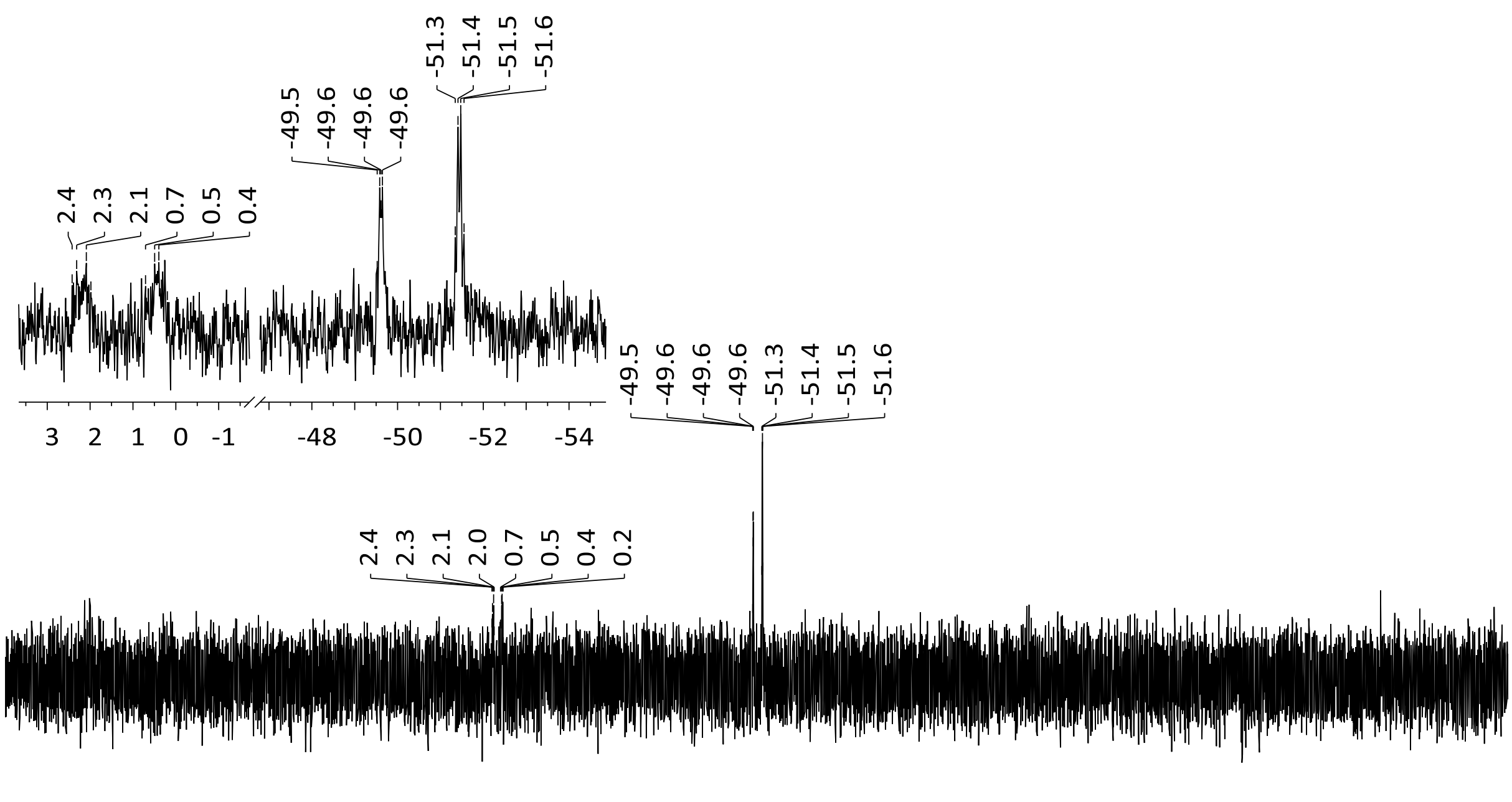

Figure S58. ^{29}Si NMR spectrum (99.4 MHz, $CDCl_3$) of *cis*-**3**.

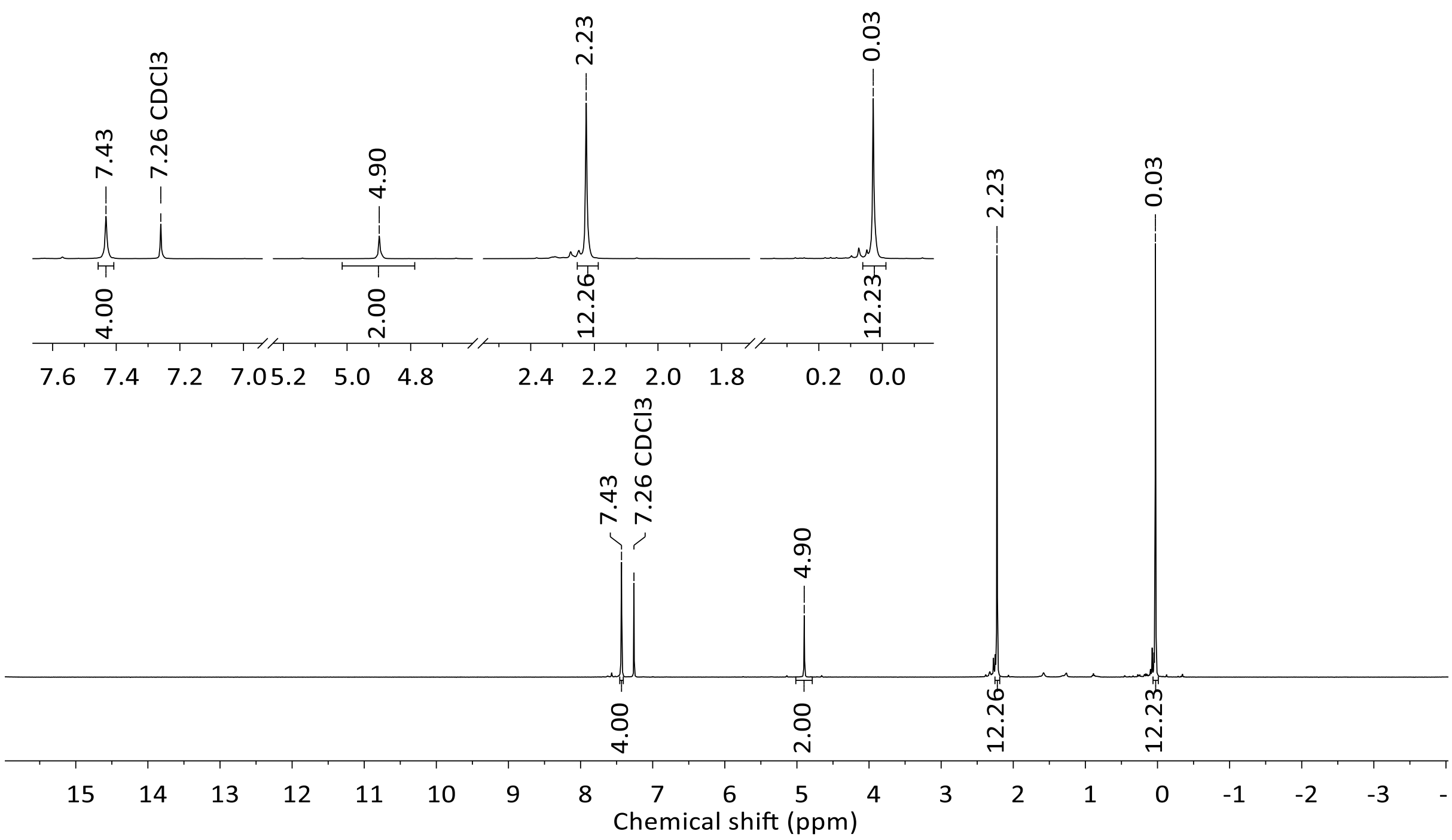

Figure S59. ^{1}H NMR spectrum (500.2 MHz, $CDCl_3$) of **4**.

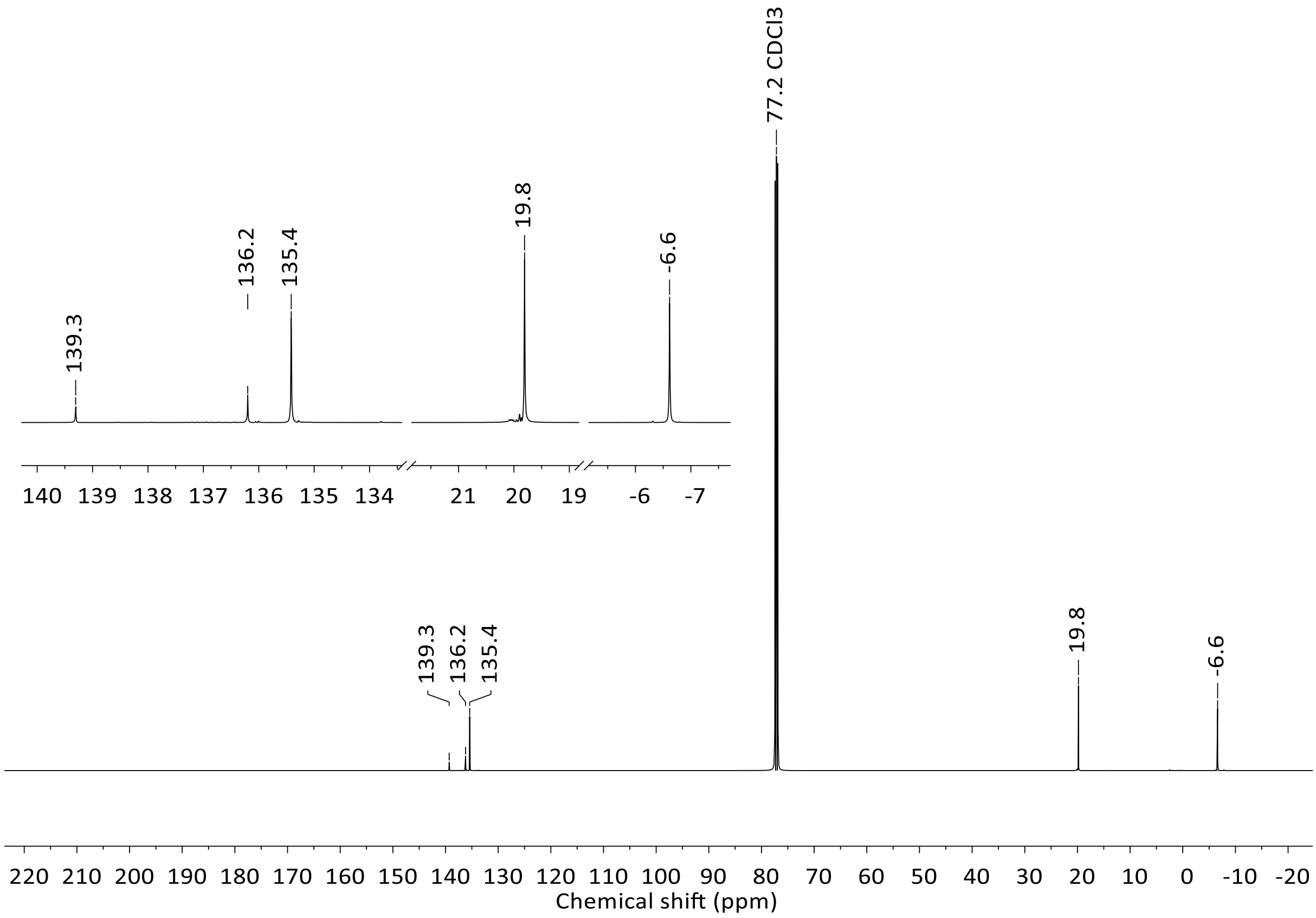

Figure S60. $^{13}C\{^{1}H\}$ NMR spectrum (125.8 MHz, $CDCl_3$) of **4**.

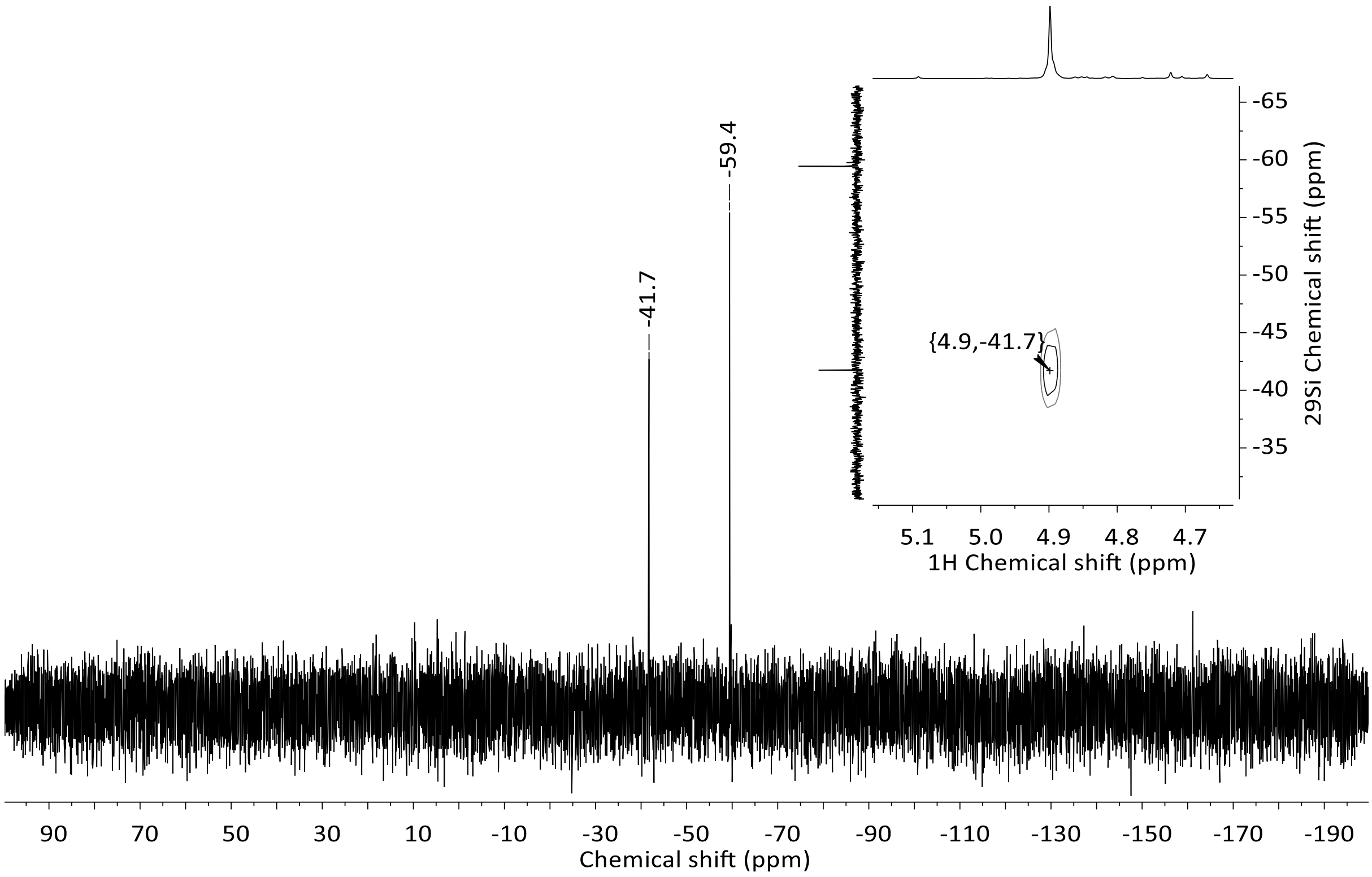

Figure S61. $^{29}Si\{^1H\}$ NMR spectrum (99.4 MHz, $CDCl_3$) of **4**, including an excerpt of the ^1H–^{29}Si HSQC NMR spectrum (^1H: 500.2 MHz, ^{29}Si: 99.4 MHz, $CDCl_3$).

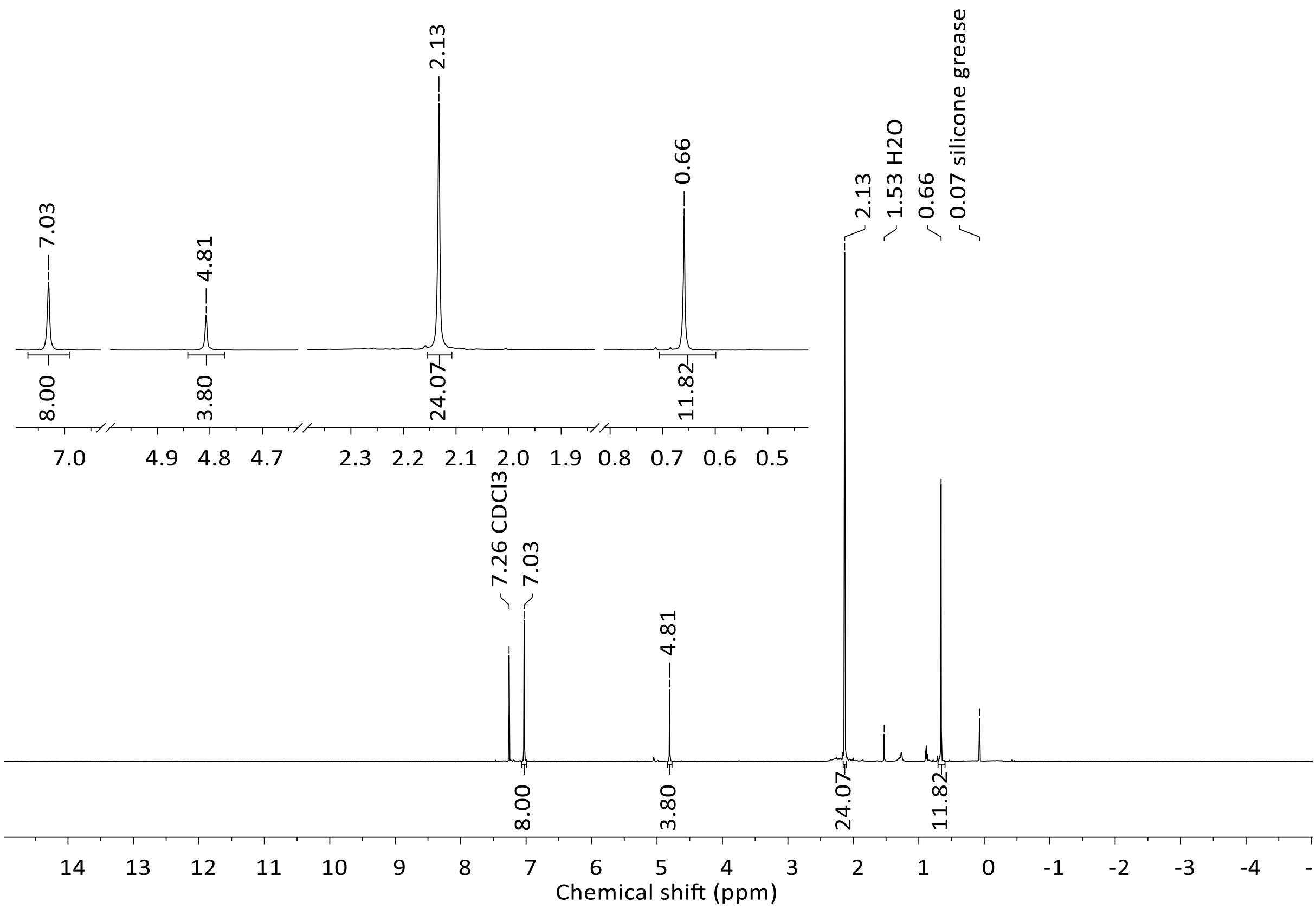

Figure S62. ^1H NMR spectrum (500.2 MHz, $CDCl_3$) of **5**.

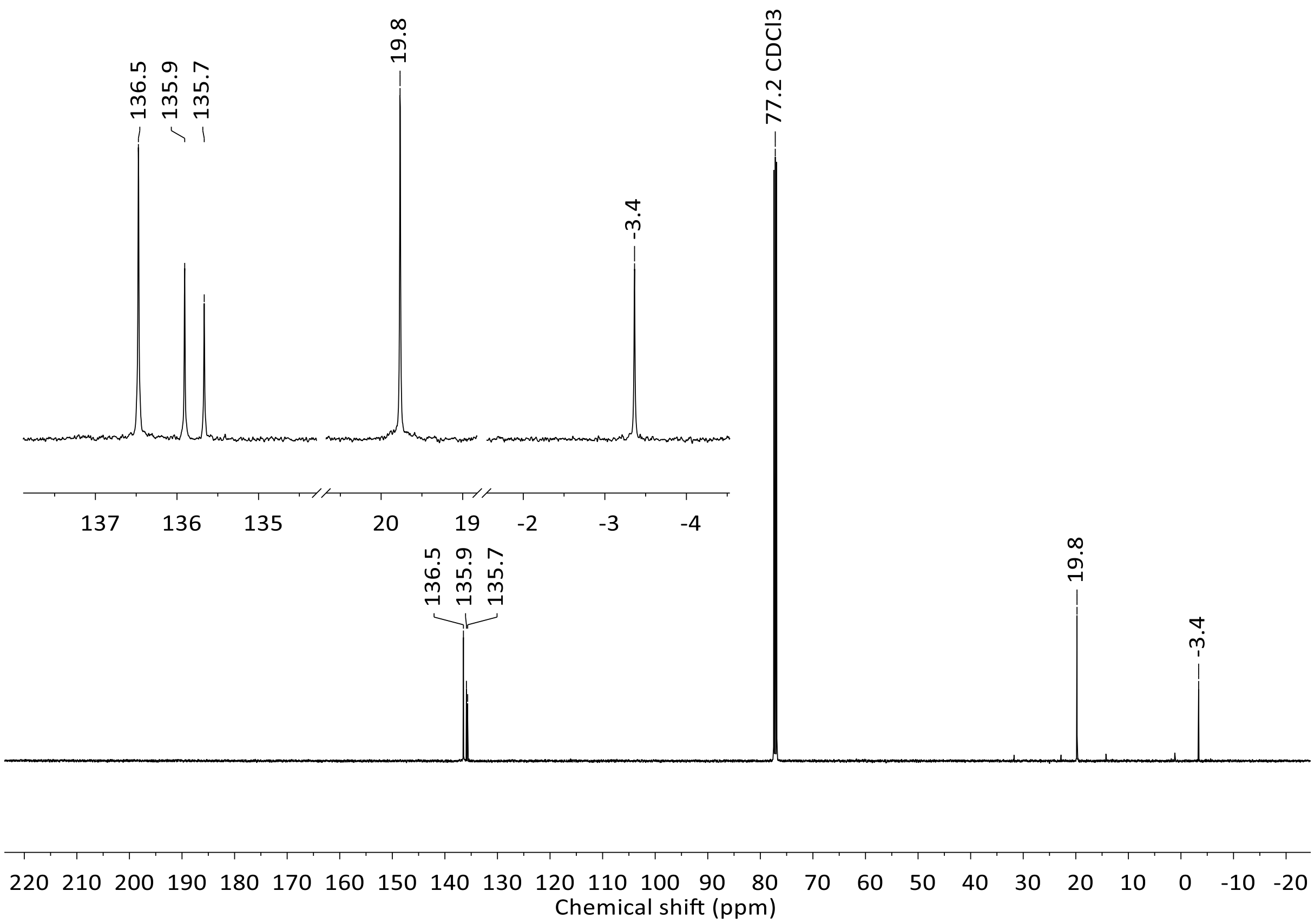

Figure S63. $^{13}C\{^{1}H\}$ NMR spectrum (125.8 MHz, $CDCl_3$) of **5**.

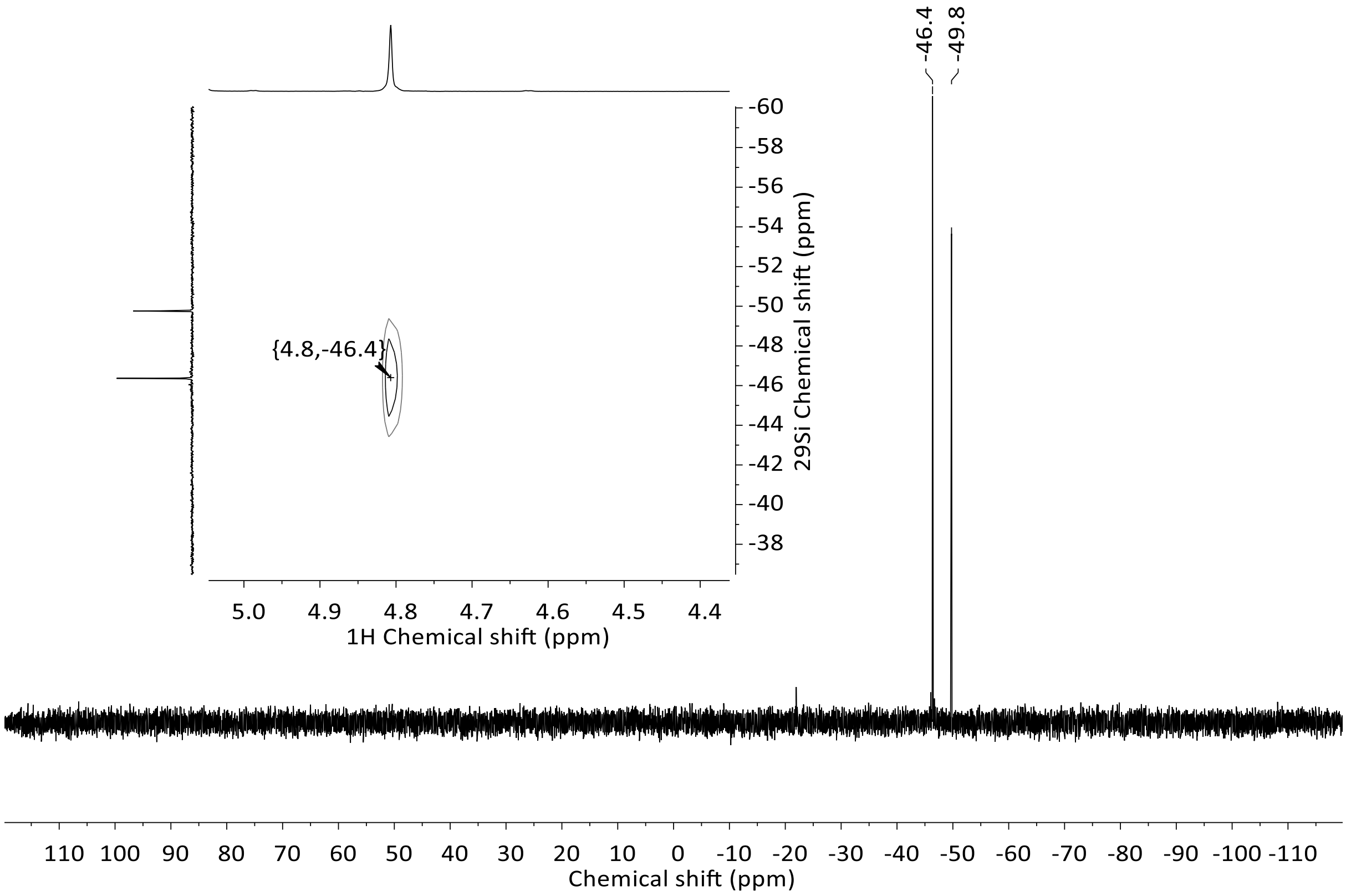

Figure S64. $^{29}Si\{^{1}H\}$ NMR spectrum (99.4 MHz, $CDCl_3$) of **5**, including an excerpt of the ^{1}H–^{29}Si HSQC NMR spectrum (^{1}H: 500.2 MHz, ^{29}Si: 99.4 MHz, $CDCl_3$).

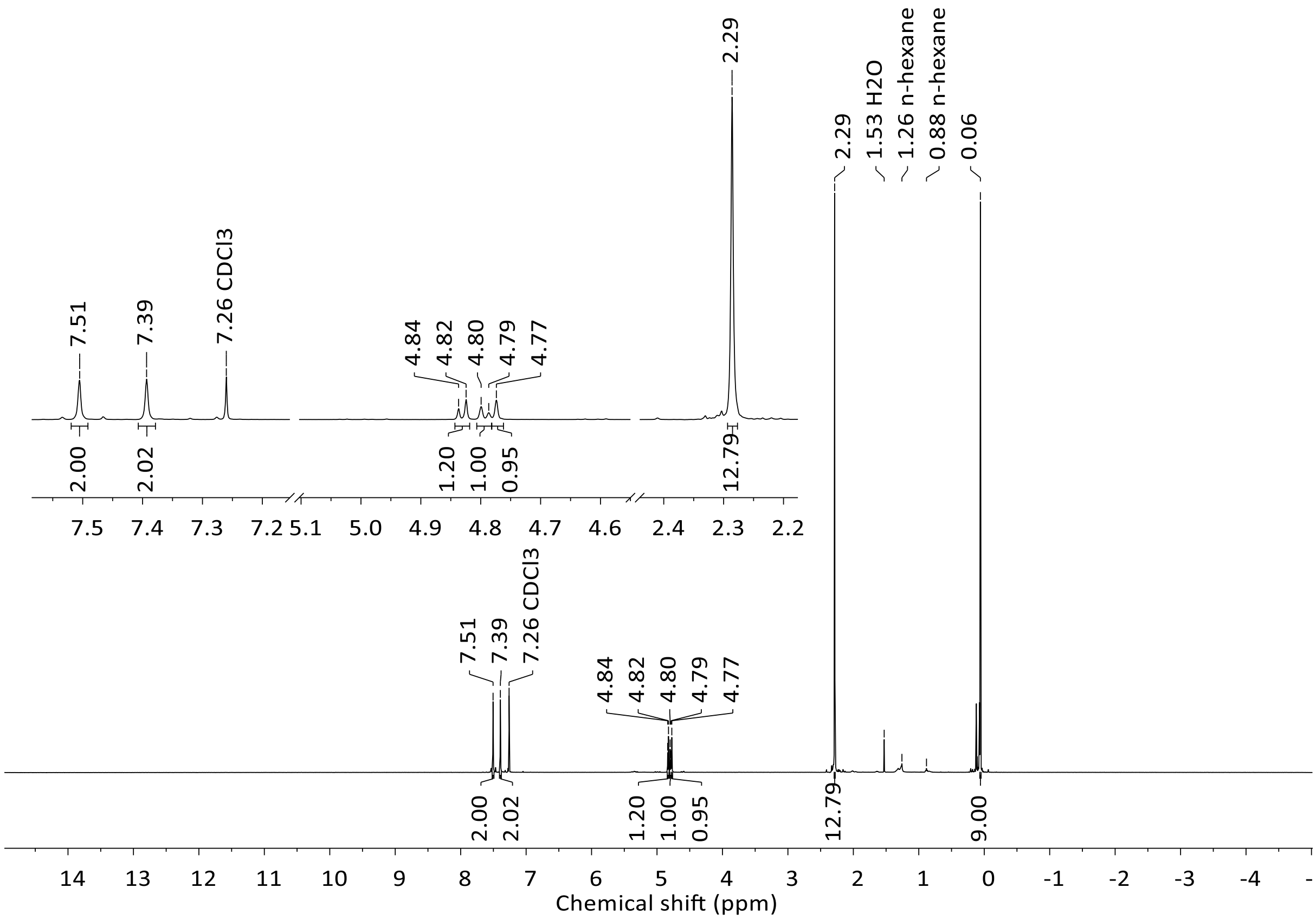

Figure S65. ^{1}H NMR spectrum (500.2 MHz, $CDCl_3$) of **6**.

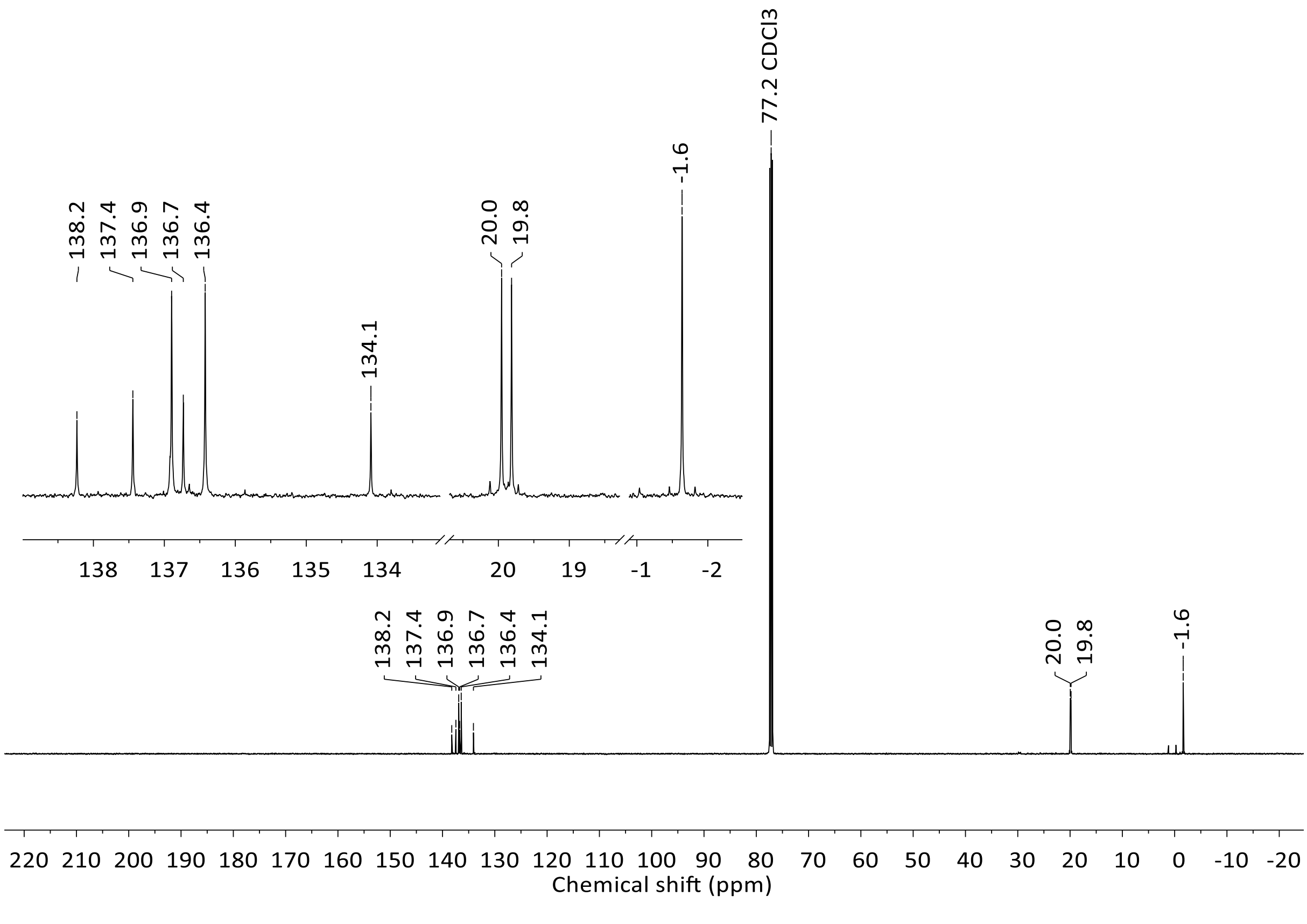

Figure S66. $^{13}C\{^{1}H\}$ NMR spectrum (125.8 MHz, $CDCl_3$) of **6**.

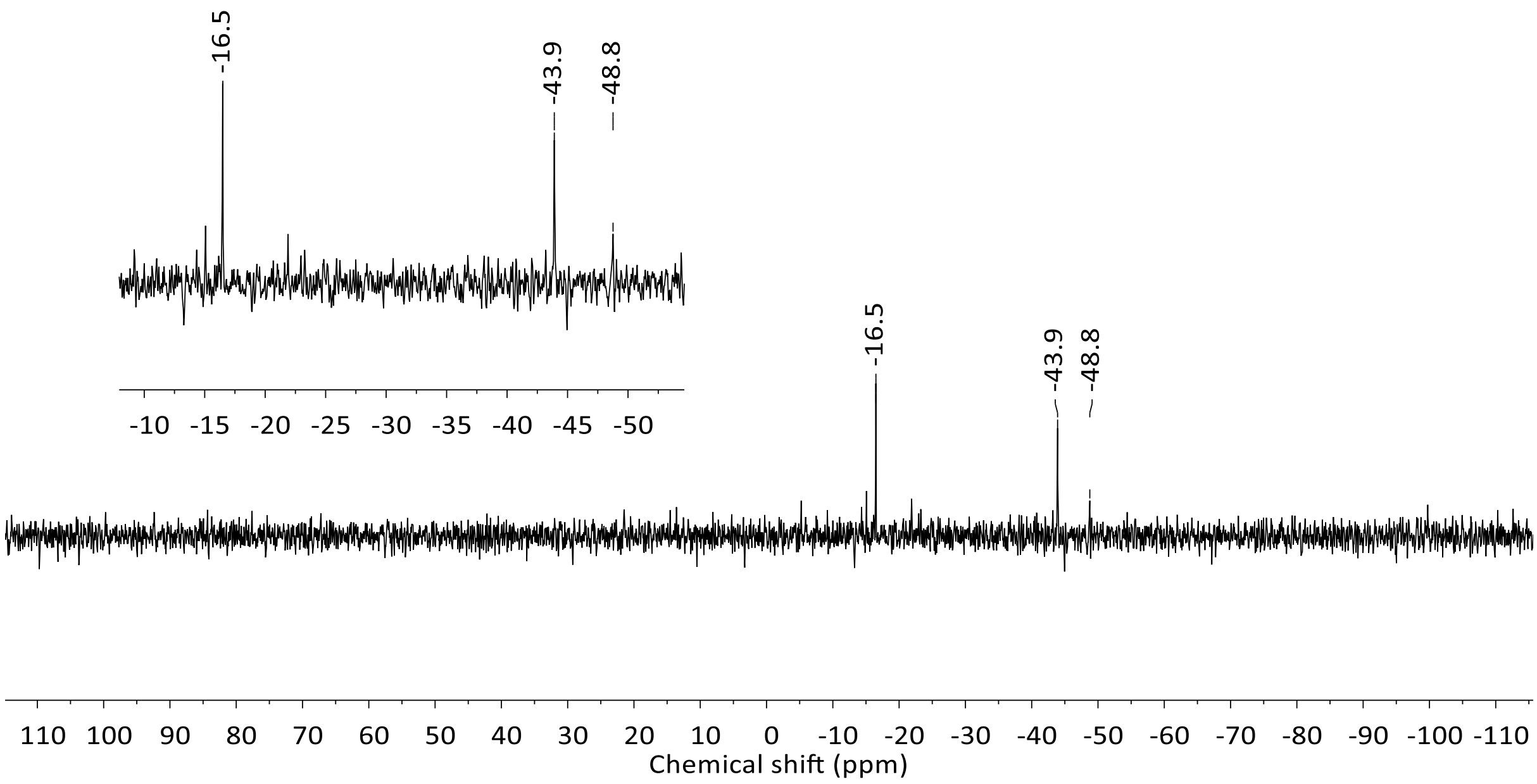

Figure S67. $^{29}Si\{^{1}H\}$ NMR spectrum (99.4 MHz, $CDCl_3$) of **6**.

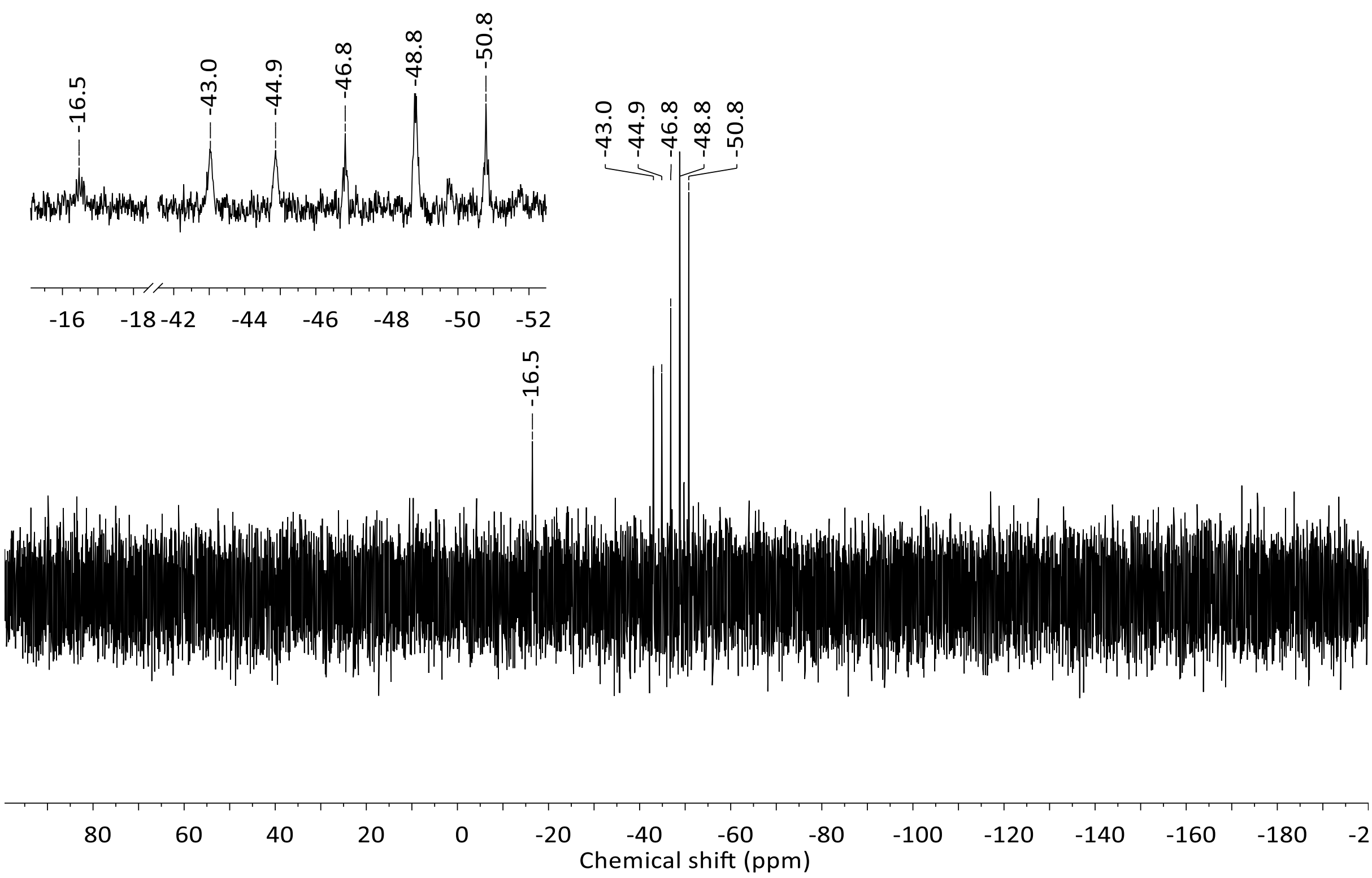

Figure S68. ^{29}Si NMR spectrum (99.4 MHz, $CDCl_3$) of **6**.

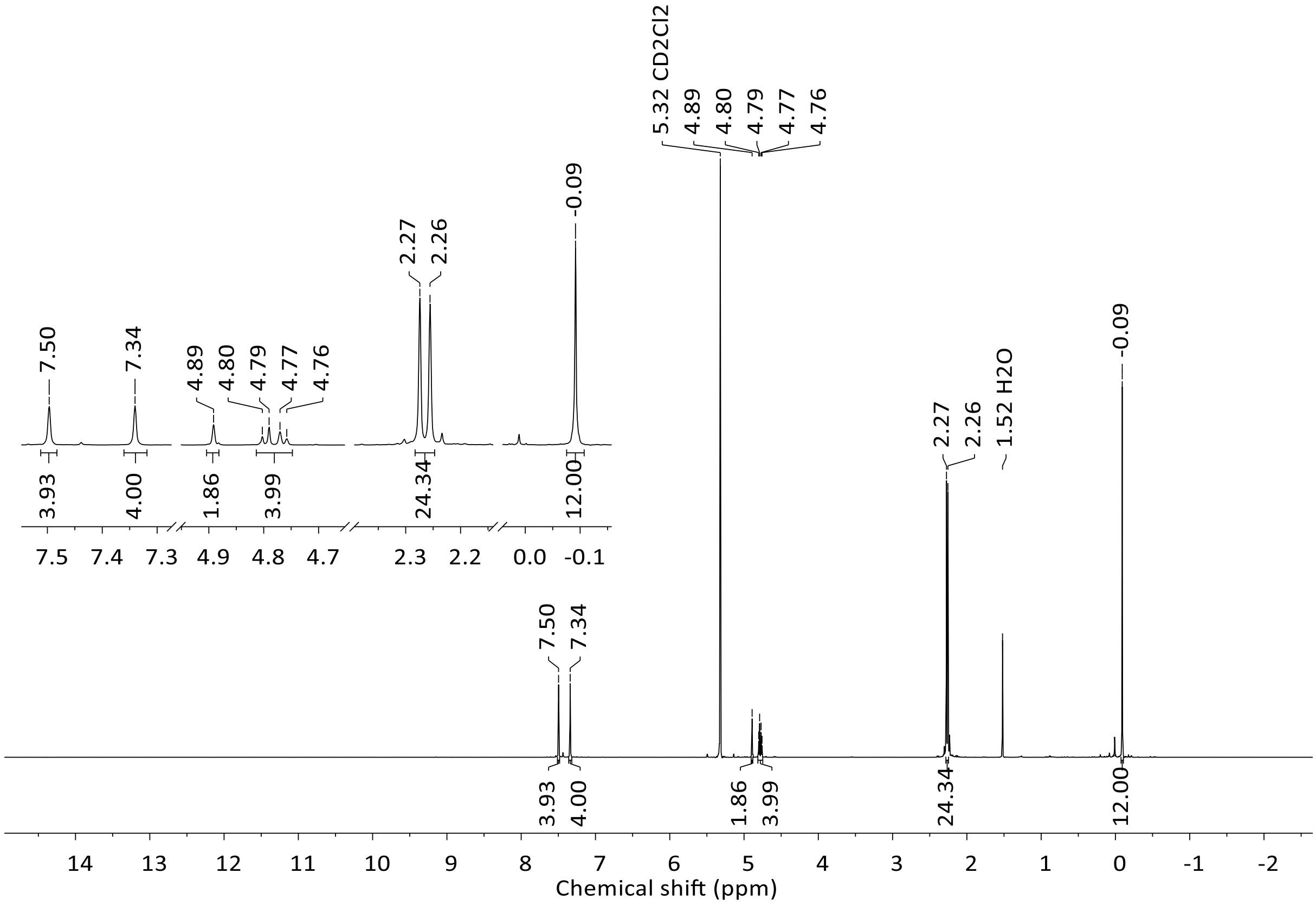

Figure S69. ^{1}H NMR spectrum (500.2 MHz, CD_2Cl_2) of **7**.

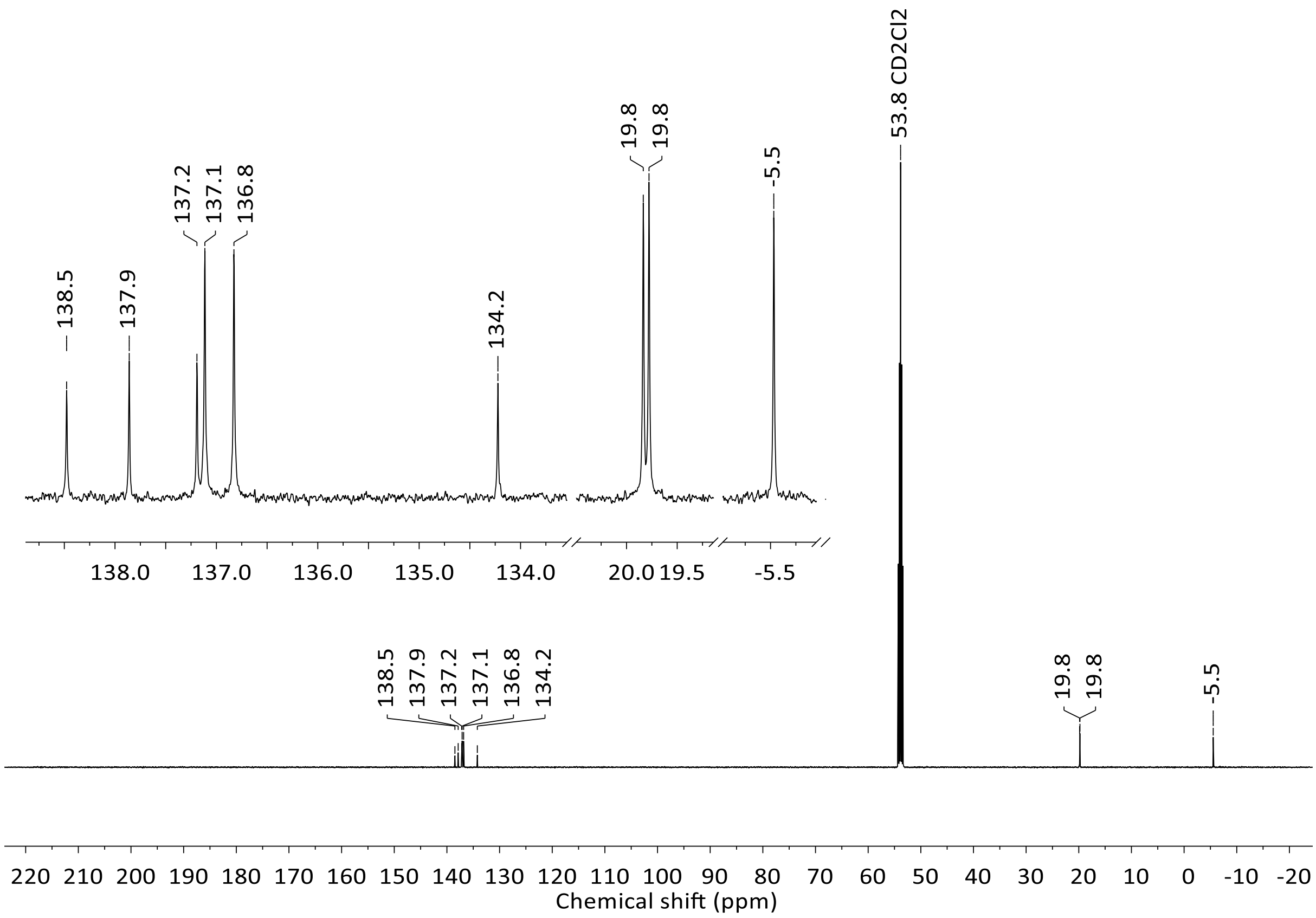

Figure S70. $^{13}C\{^1H\}$ NMR spectrum (125.8 MHz, CD_2Cl_2) of **7**.

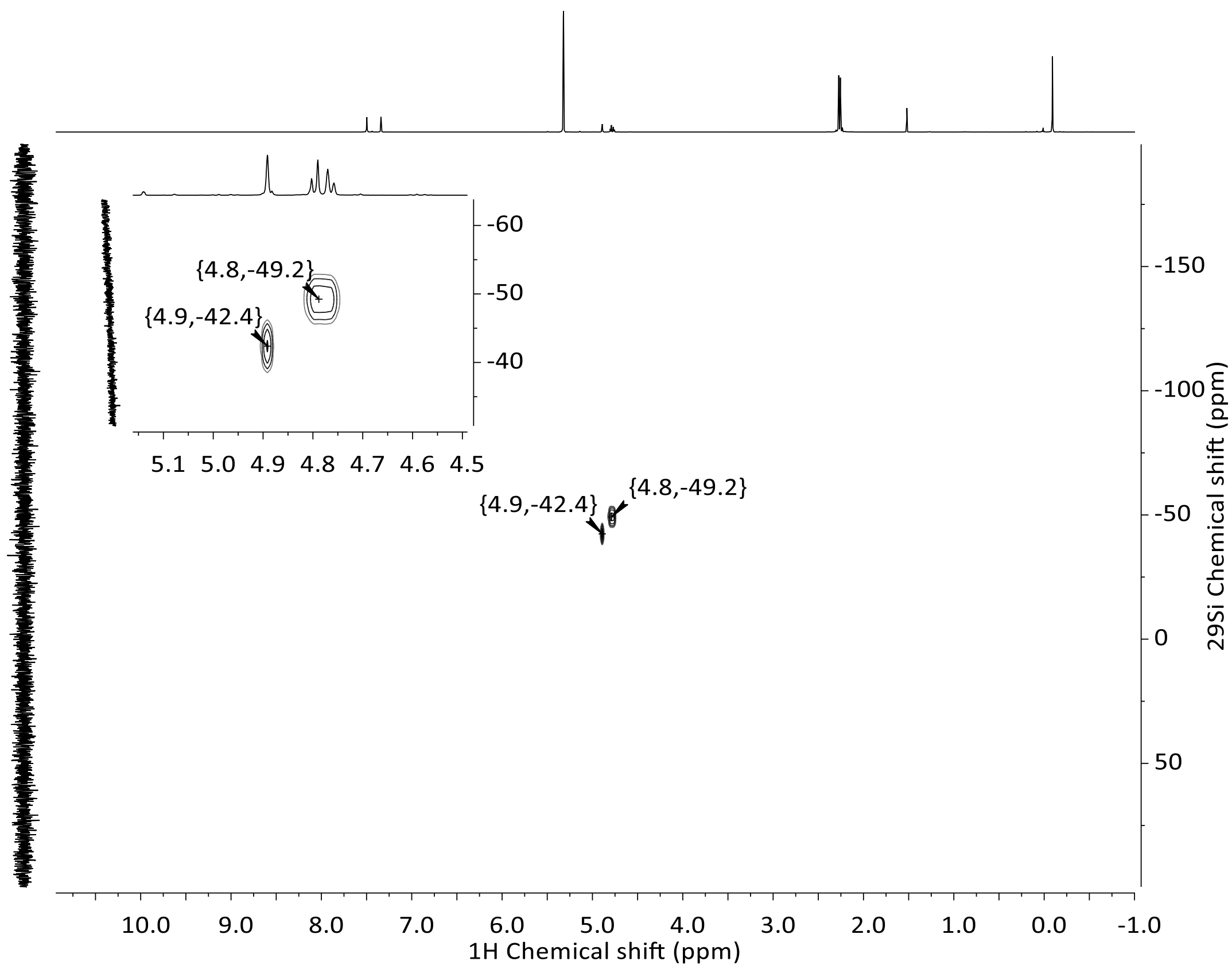

Figure S71. ^{1}H–^{29}Si HSQC NMR spectrum (^{1}H: 500.2 MHz, ^{29}Si: 99.4 MHz, CD_2Cl_2) of **7**.

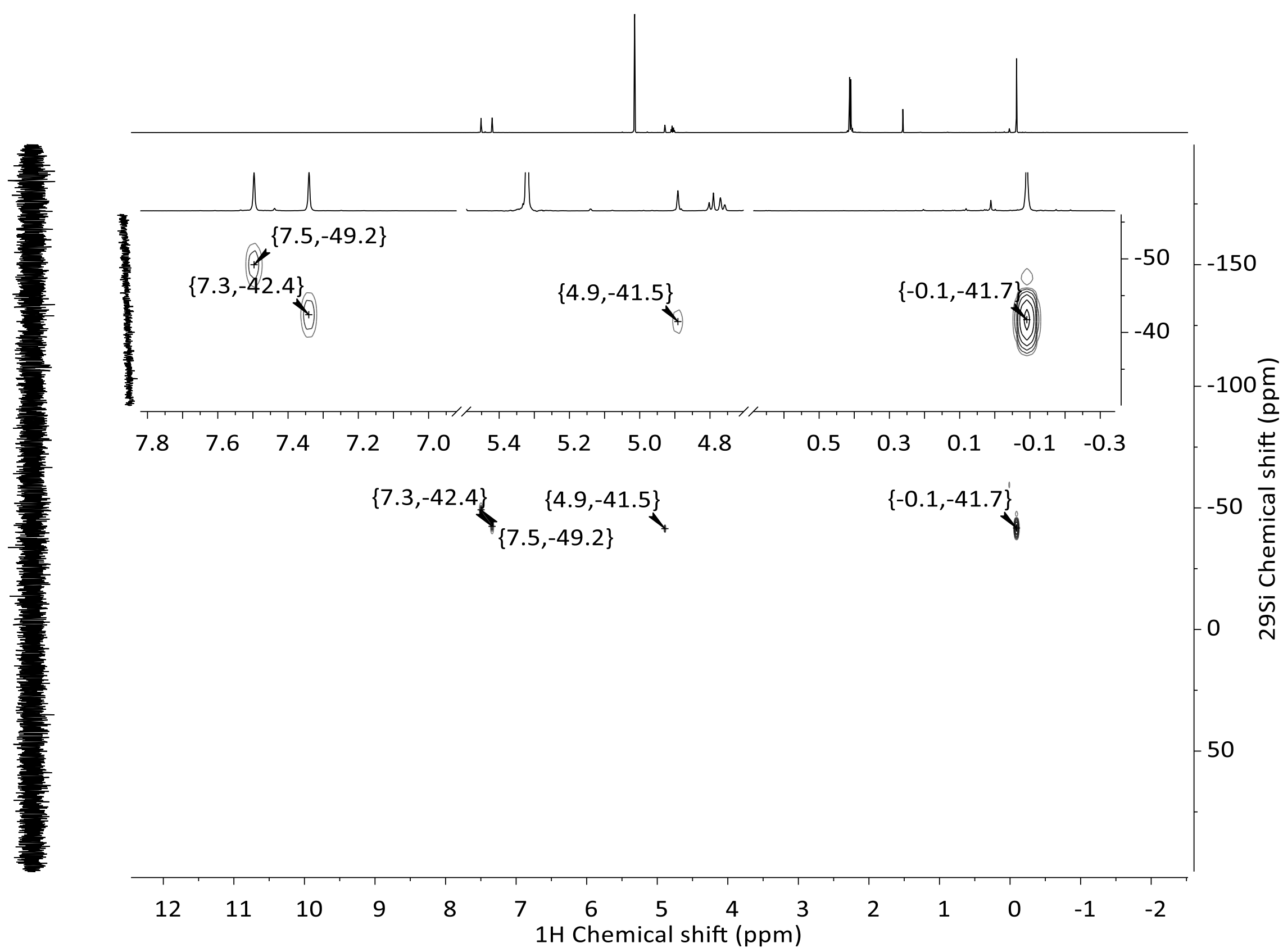

Figure S72. ^{1}H–^{29}Si HMBC NMR spectrum (^{1}H: 500.2 MHz, ^{29}Si: 99.4 MHz, CD_2Cl_2) of **7**.

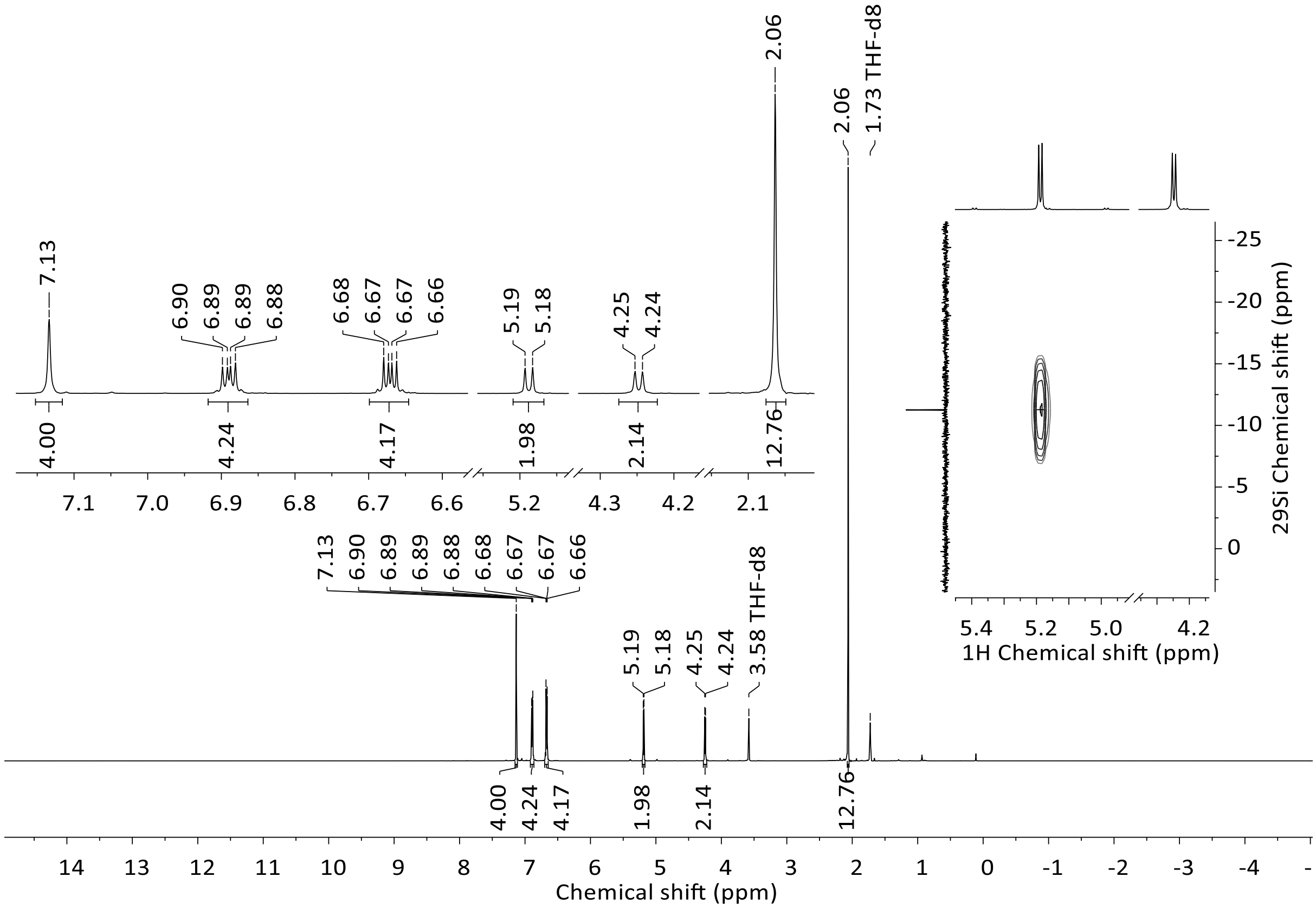

Figure S73. ^{1}H NMR spectrum (500.2 MHz, THF-d_8) of **13**, including an excerpt of the $^{1}H–^{29}Si$ HSQC NMR spectrum (^{1}H: 500.2 MHz, ^{29}Si: 99.4 MHz, THF-d_8).

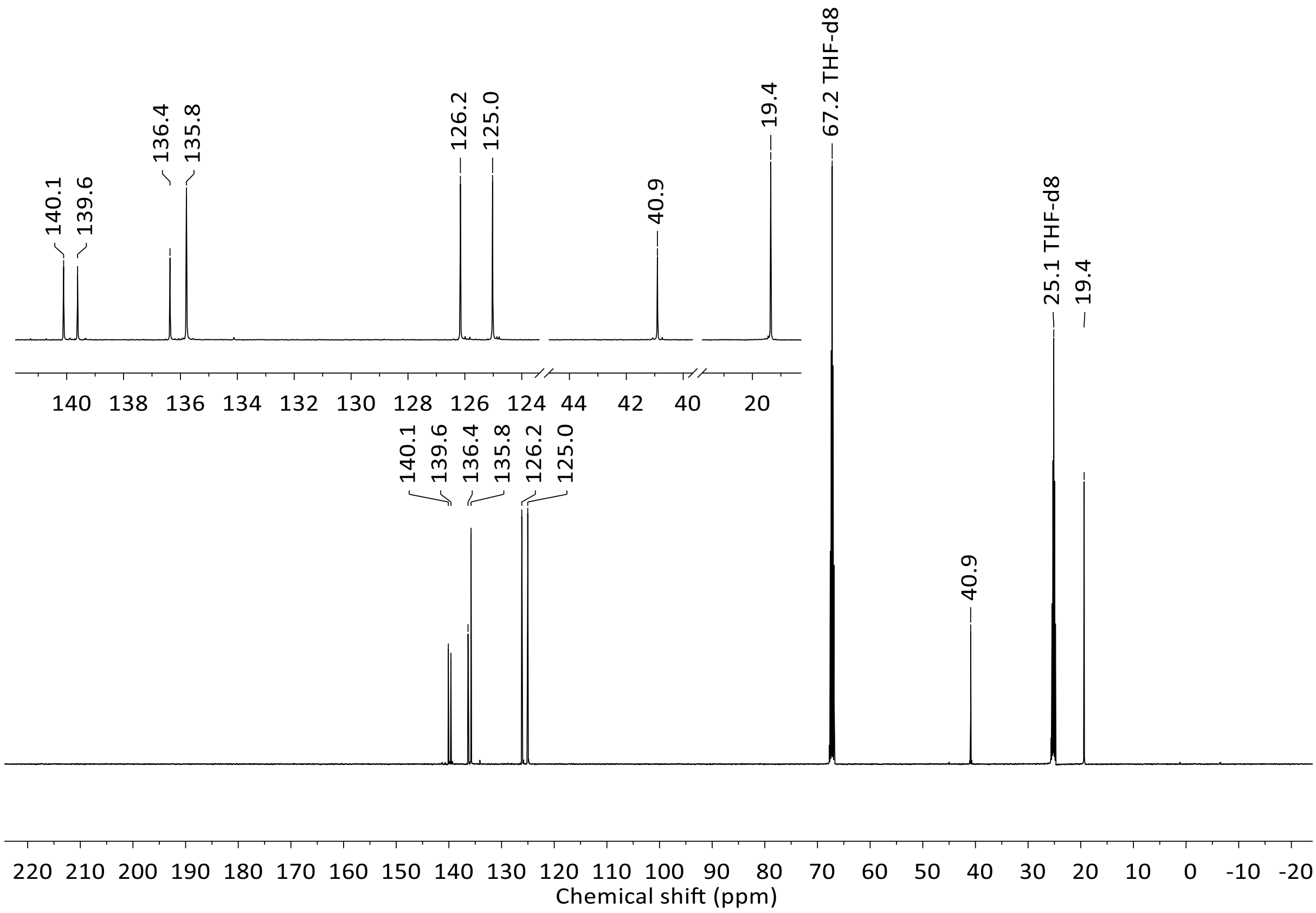

Figure S74. $^{13}C\{^{1}H\}$ NMR spectrum (125.8 MHz, THF-d_8) of **13**.

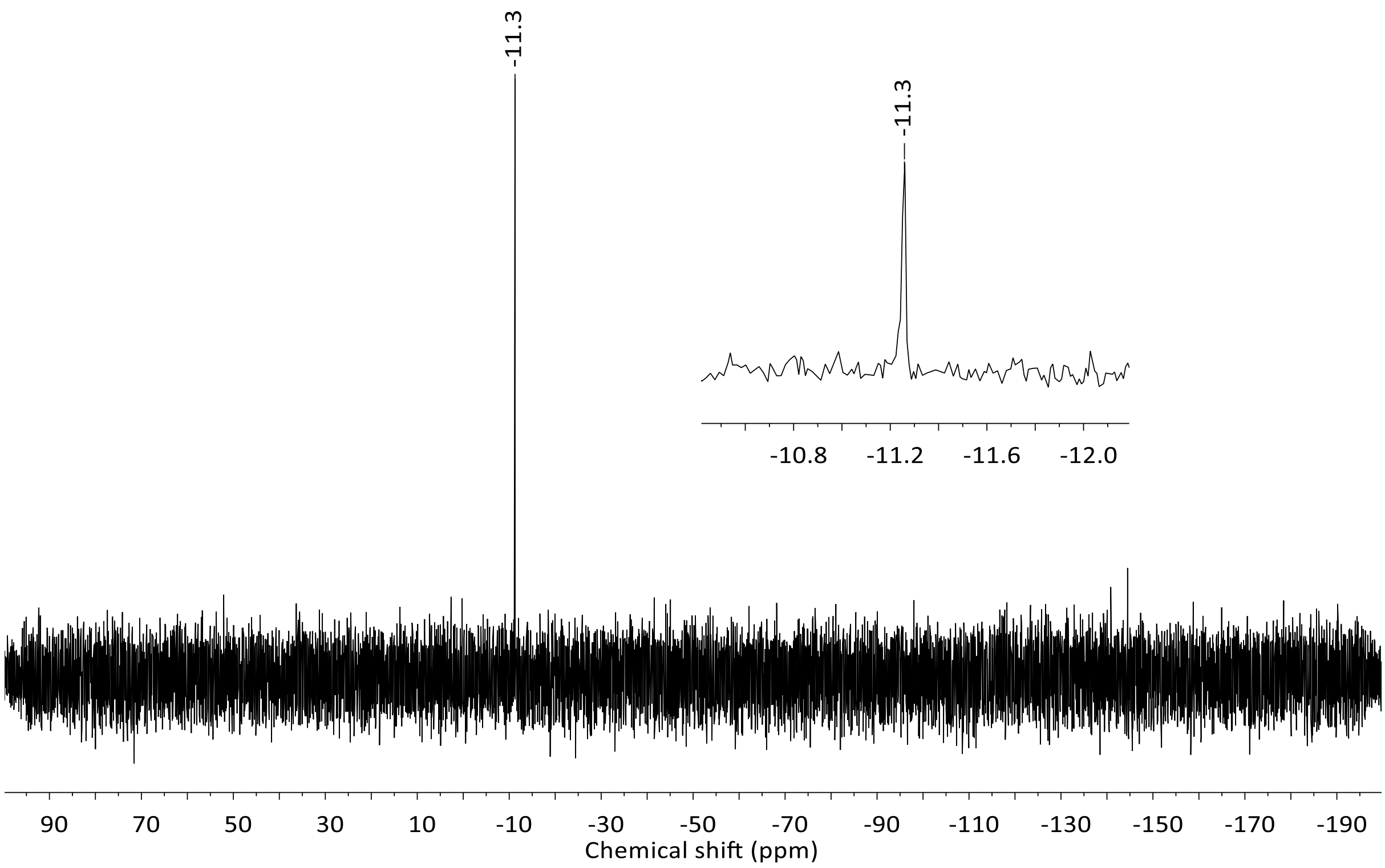

Figure S75. $^{29}Si\{^{1}H\}$ NMR spectrum (99.4 MHz, THF-d_8) of **13**.

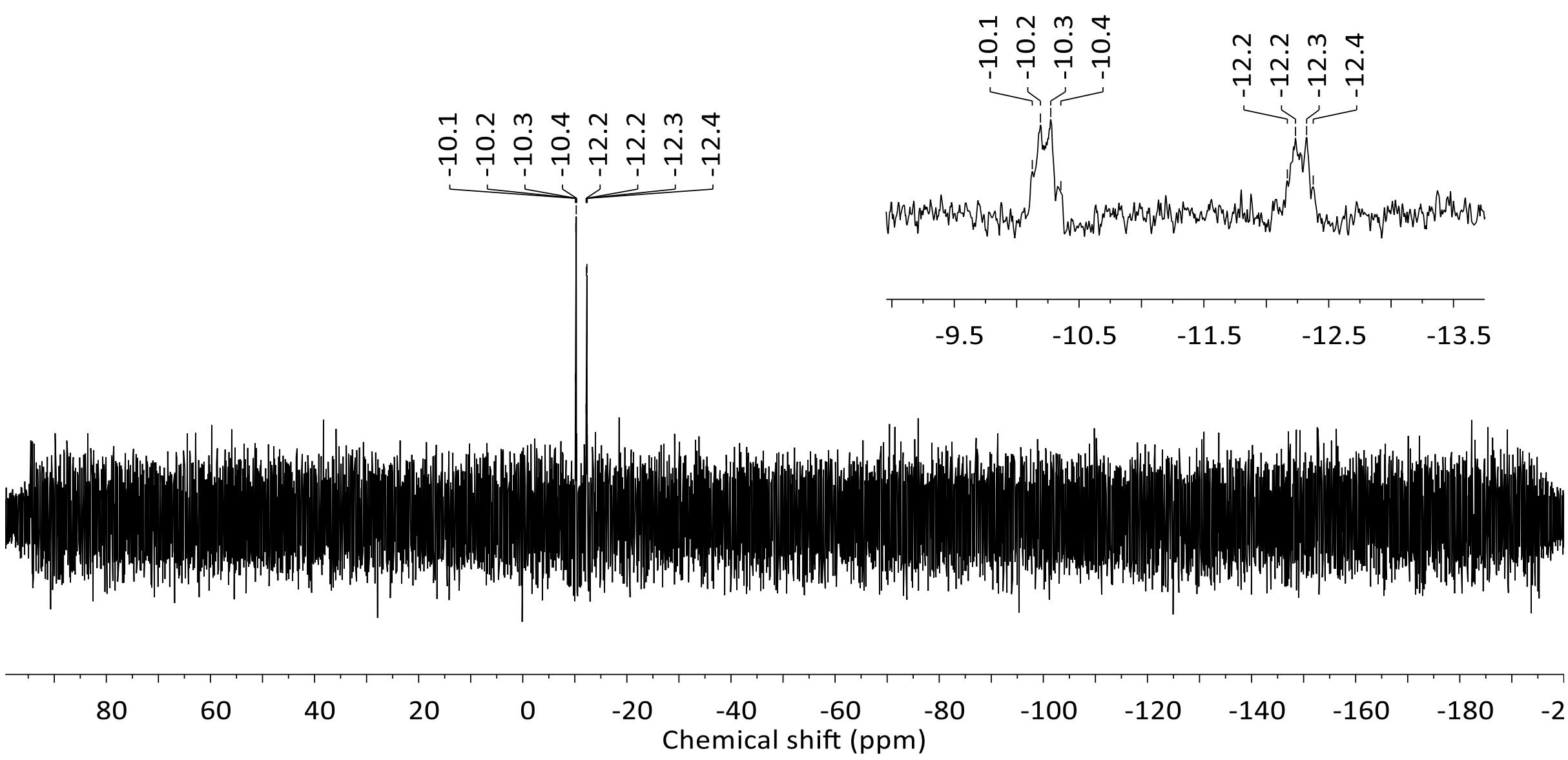

Figure S76. ^{29}Si NMR spectrum (99.4 MHz, THF-d_8) of **13**.

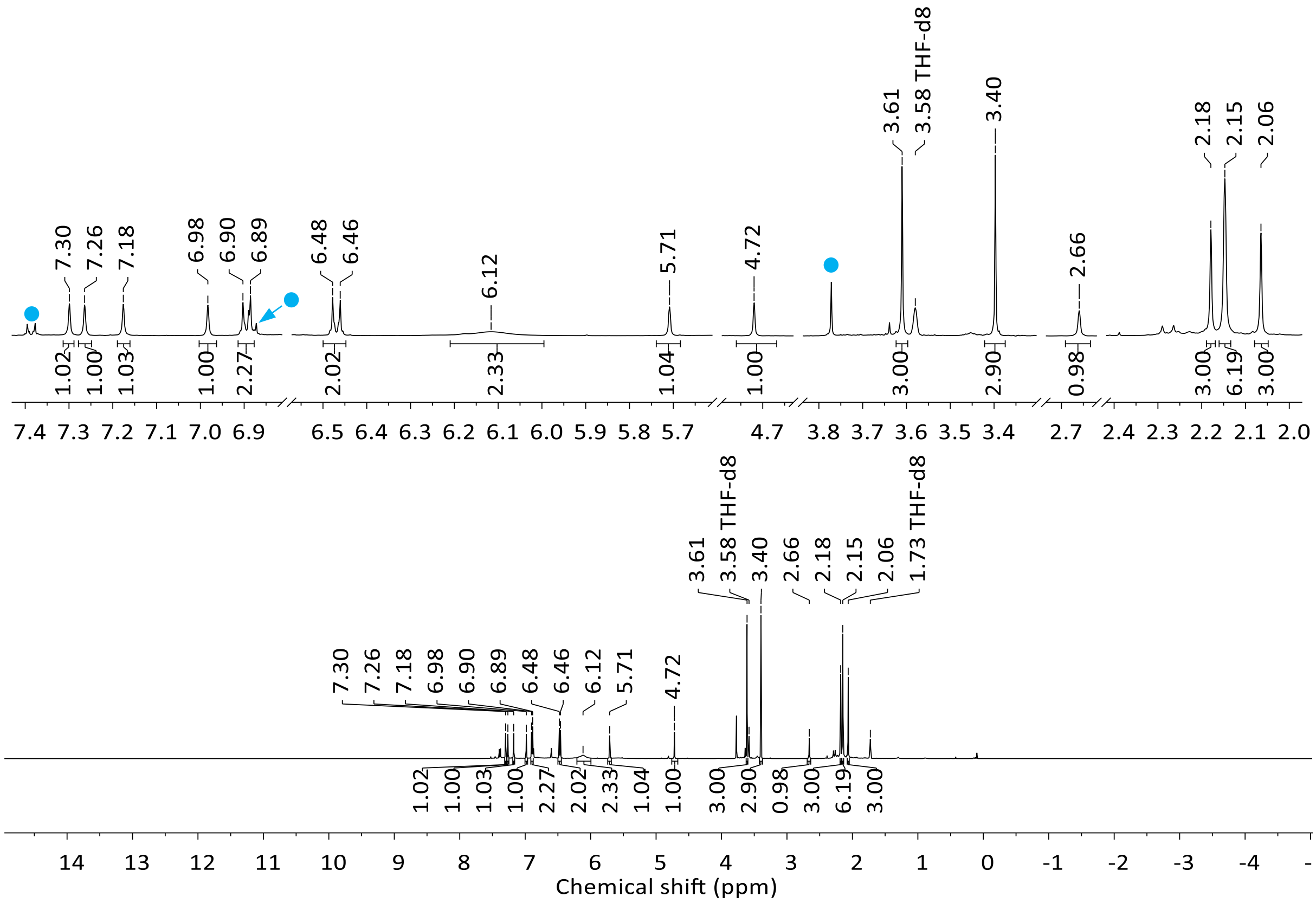

Figure S77. ^{1}H NMR spectrum (500.2 MHz, THF-d_8) of K[**15^{Ans}**]. Marked contaminant: AnsC≡CAns (blue dots).

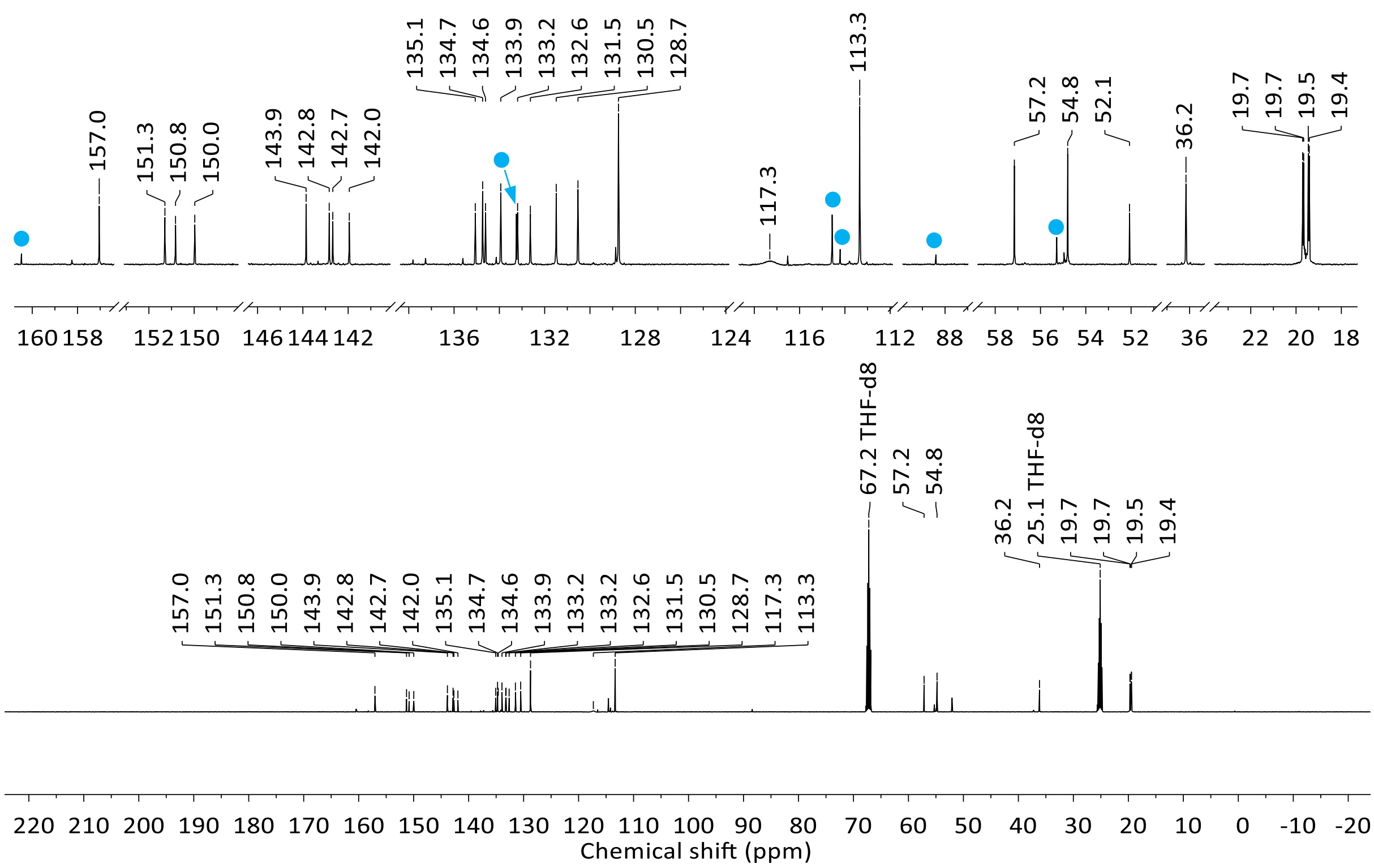

Figure S78. ^{13}C{^{1}H} NMR spectrum (125.8 MHz, THF-d_8) of K[**15^{Ans}**]. Marked contaminant: AnsC≡CAns (blue dots).

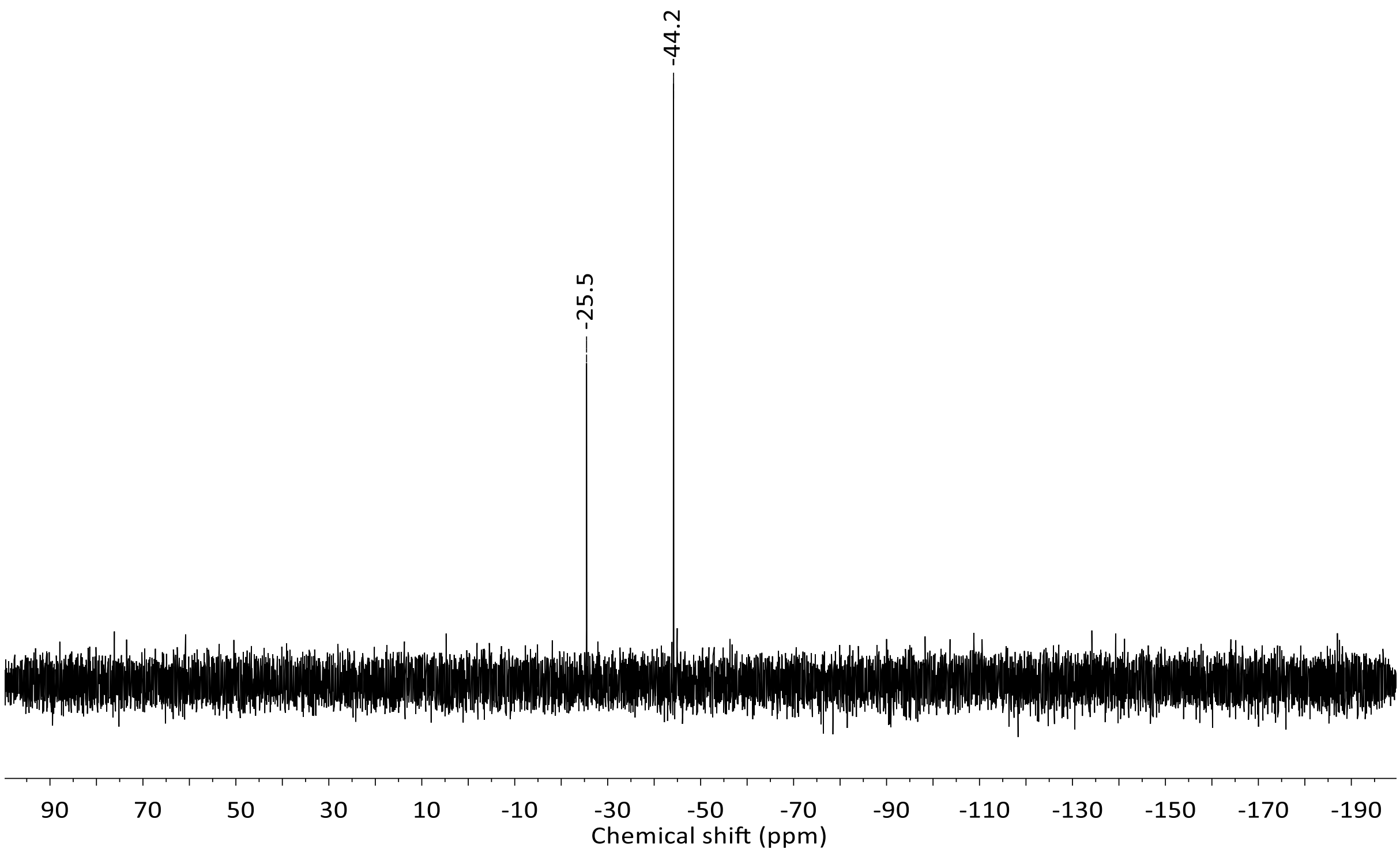

Figure S79. $^{29}Si\{^{1}H\}$ NMR spectrum (99.4 MHz, THF-d_8) of K[**15**$^{\mathbf{Ans}}$].

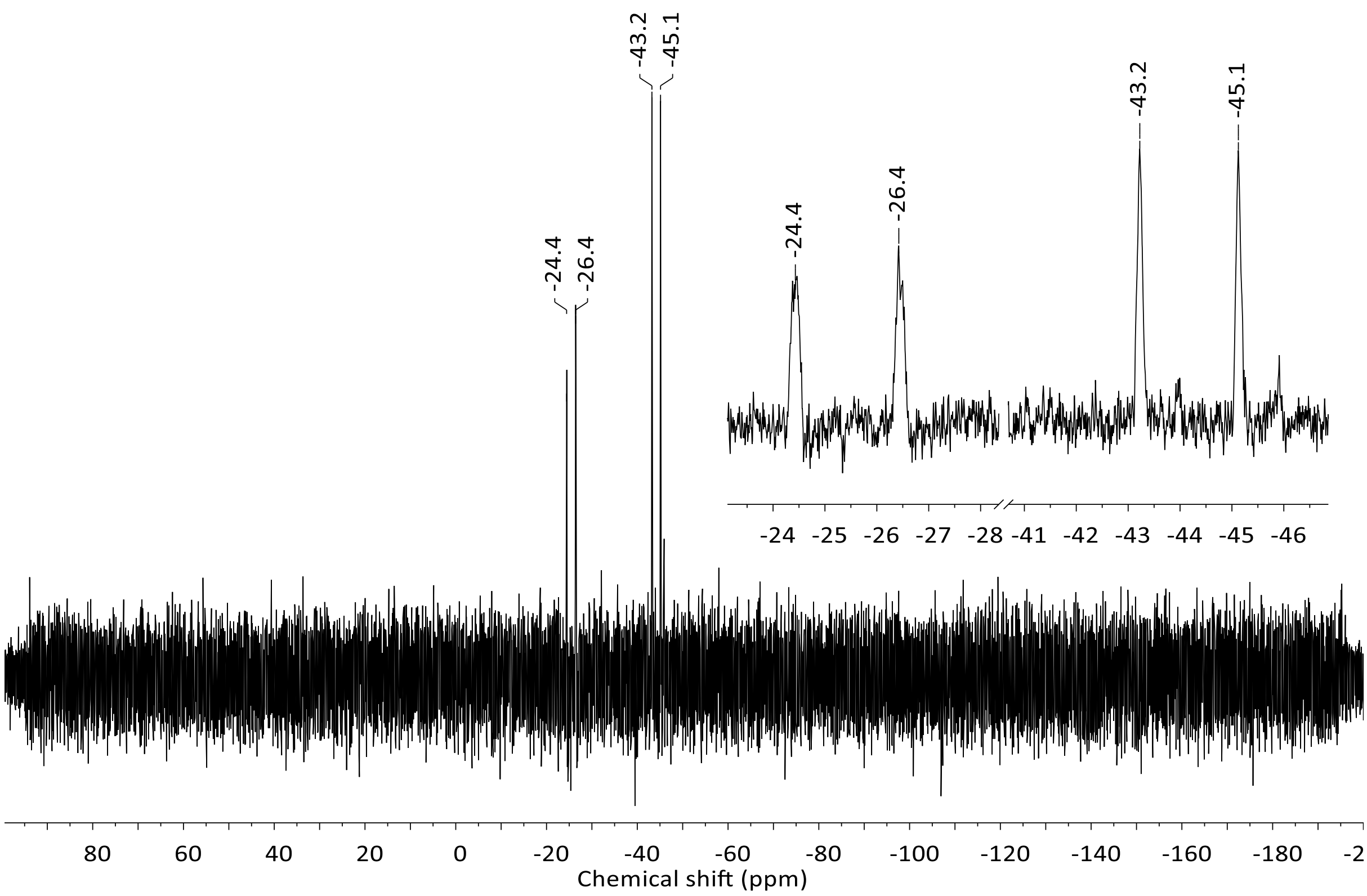

Figure S80. ^{29}Si NMR spectrum (99.4 MHz, THF-d_8) of K[**15**$^{\mathbf{Ans}}$].

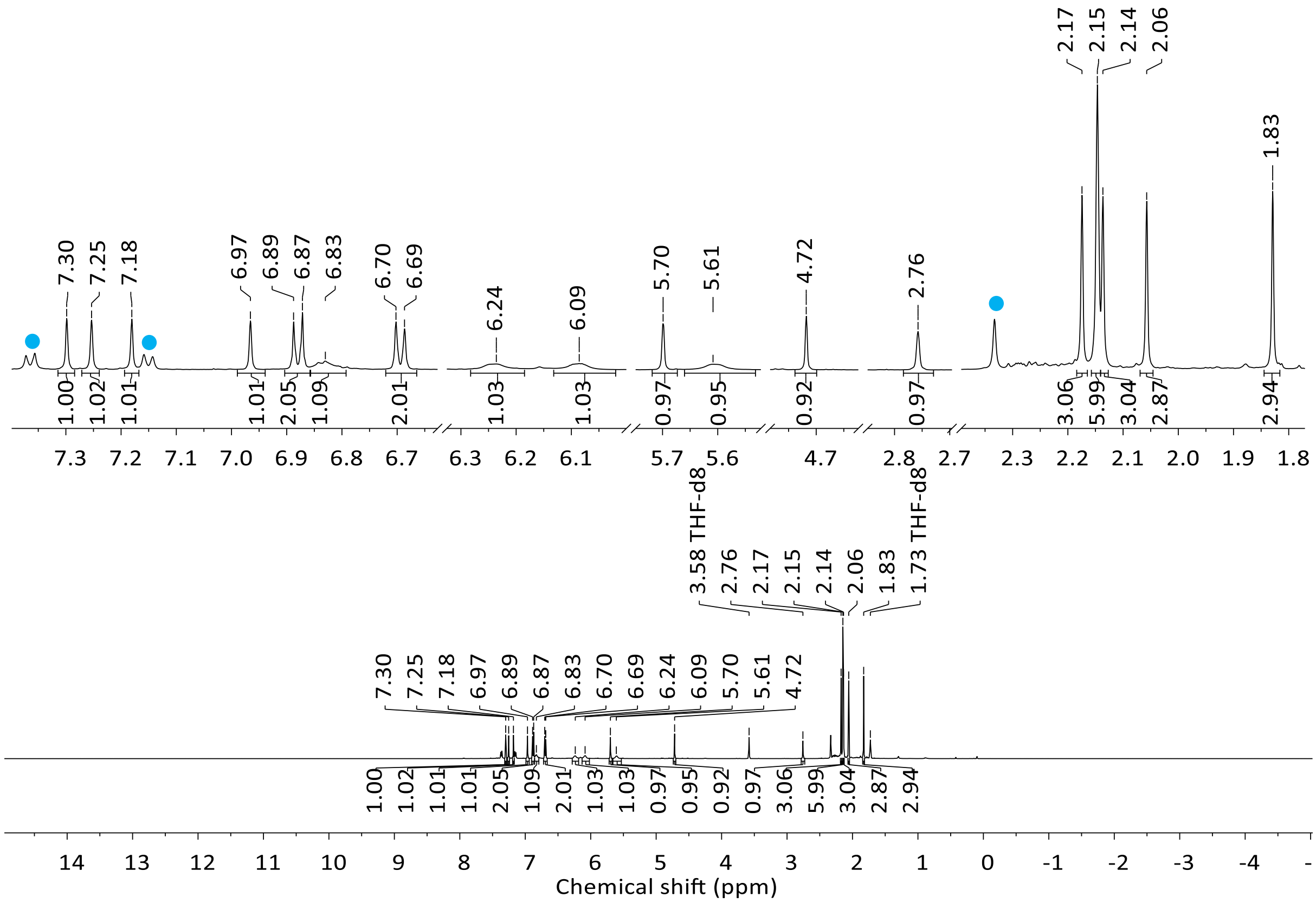

Figure S81. ^{1}H NMR spectrum (500.2 MHz, THF-d_8) of K[**15**]. Marked contaminant: *p*TolC≡C*p*Tol (blue dots).

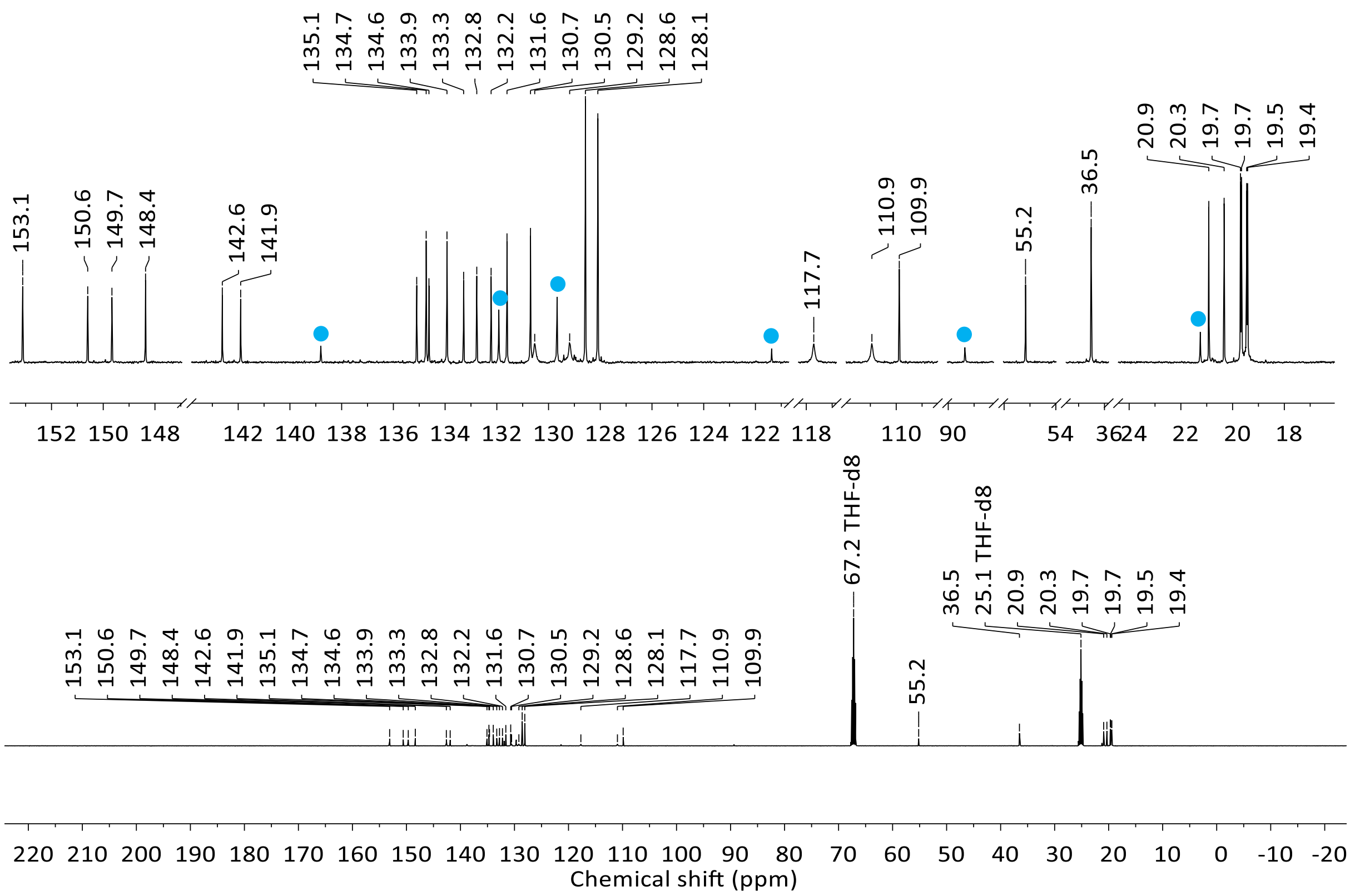

Figure S82. ^{13}C{^{1}H} NMR spectrum (125.8 MHz, THF-d_8) of K[**15**]. Marked contaminant: *p*TolC≡C*p*Tol (blue dots).

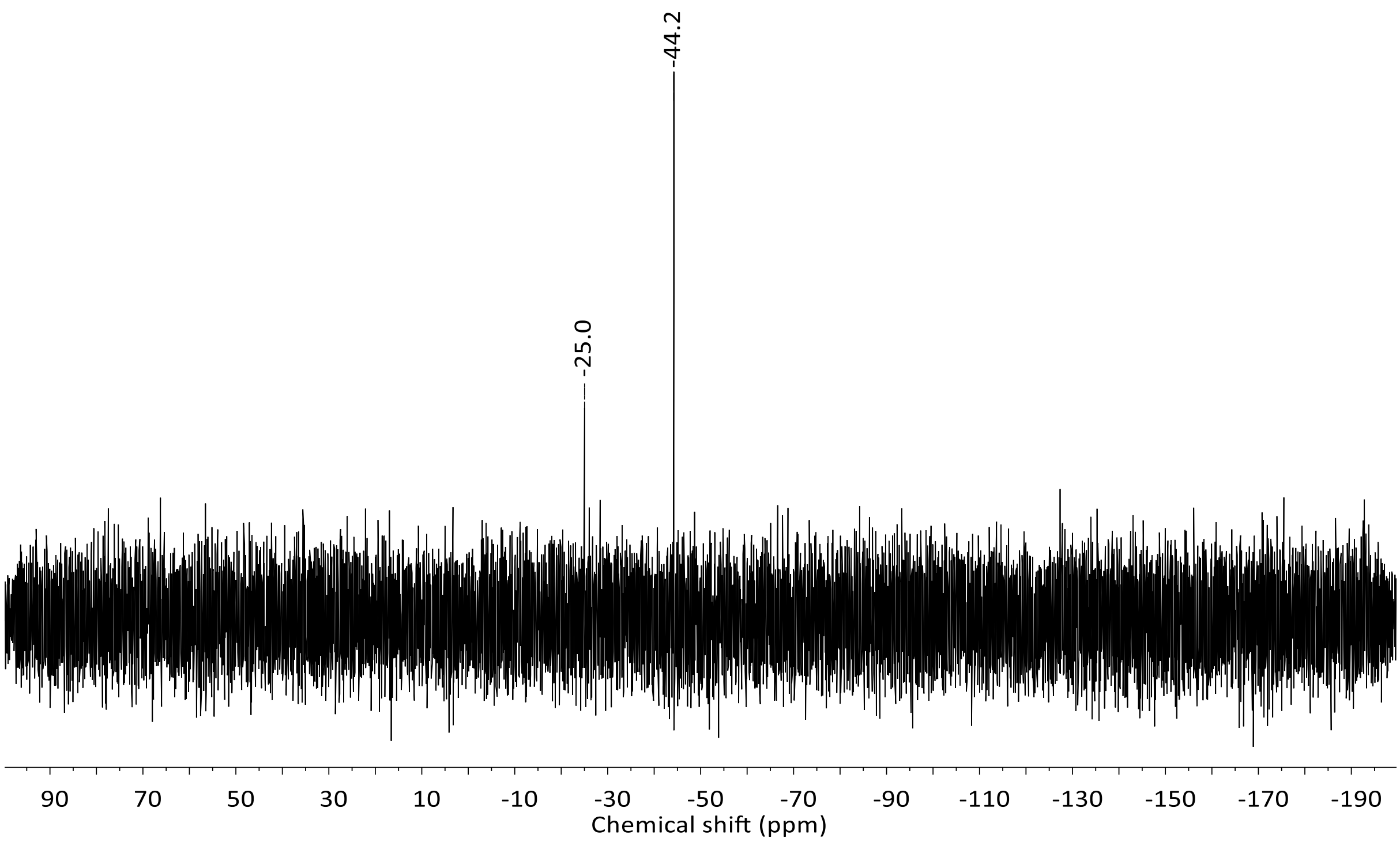

Figure S83. $^{29}Si\{^{1}H\}$ NMR spectrum (99.4 MHz, THF-d_8) of K[**15**].

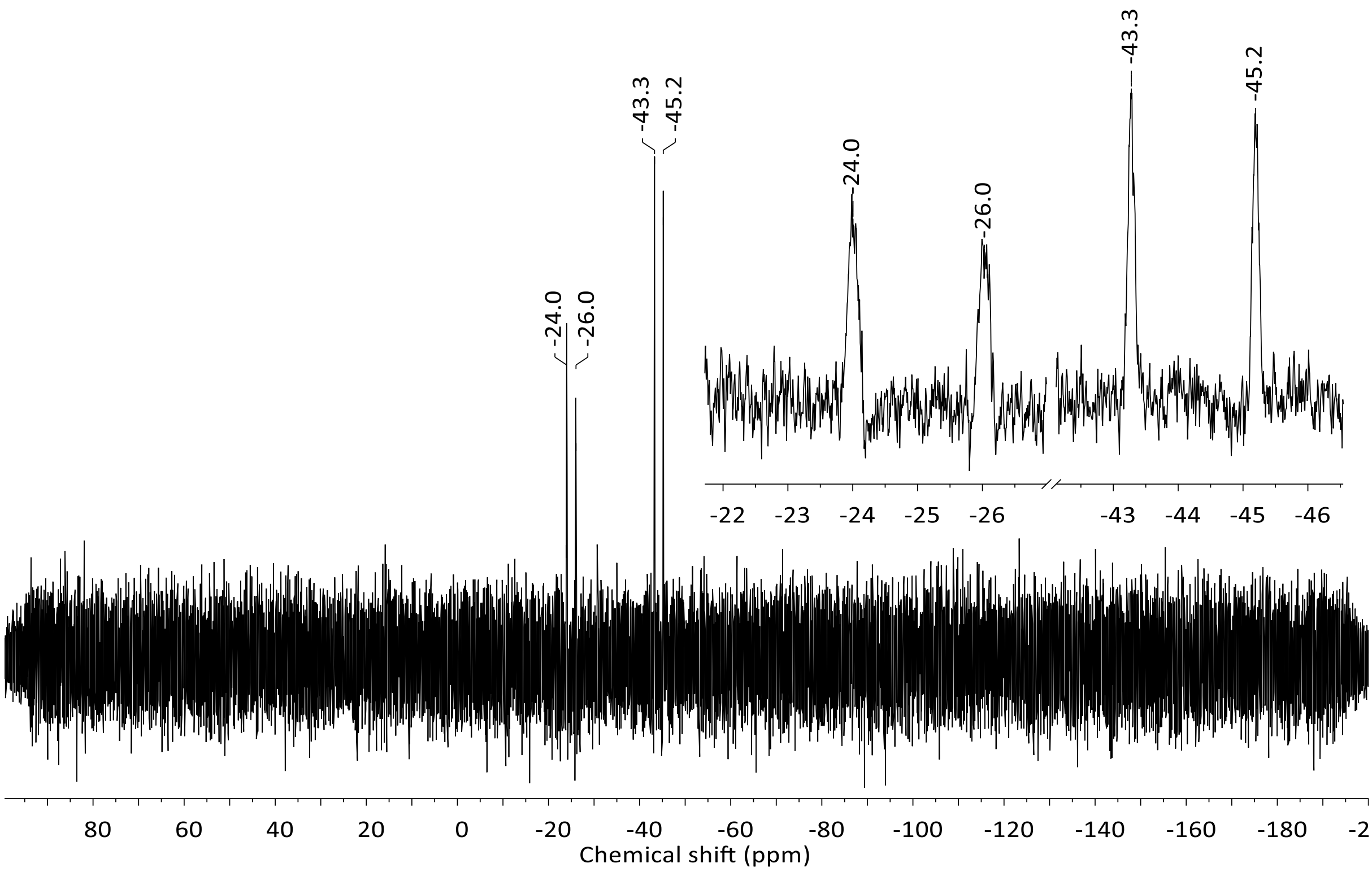

Figure S84. ^{29}Si NMR spectrum (99.4 MHz, THF-d_8) of K[**15**].

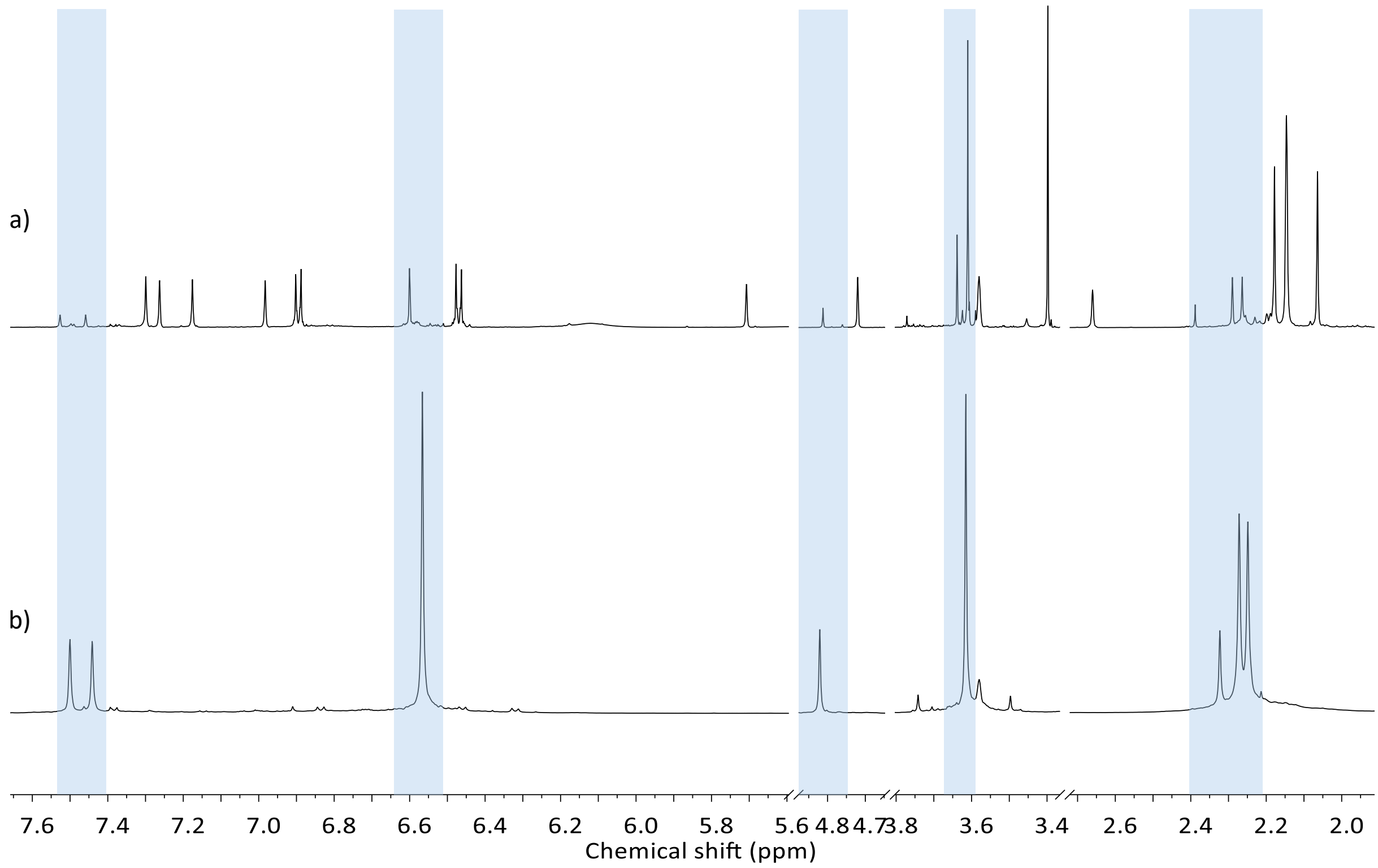

Figure S85. ^{1}H NMR spectra (500.2 MHz, THF-d_8) monitoring the reaction of K[**1^{H}**] with 1,2-bis(4-methoxyphenyl)ethyne in a sealed NMR tube: (a) reaction mixture after addition of K[**1^{H}**], showing K[**15^{Ans}**] as the major species together with resonances of **16a^{Ans}**; (b) same mixture after addition of $HNTf_2$, resulting in immediate decolorization and complete conversion of K[**15^{Ans}**] into **16a^{Ans}**.

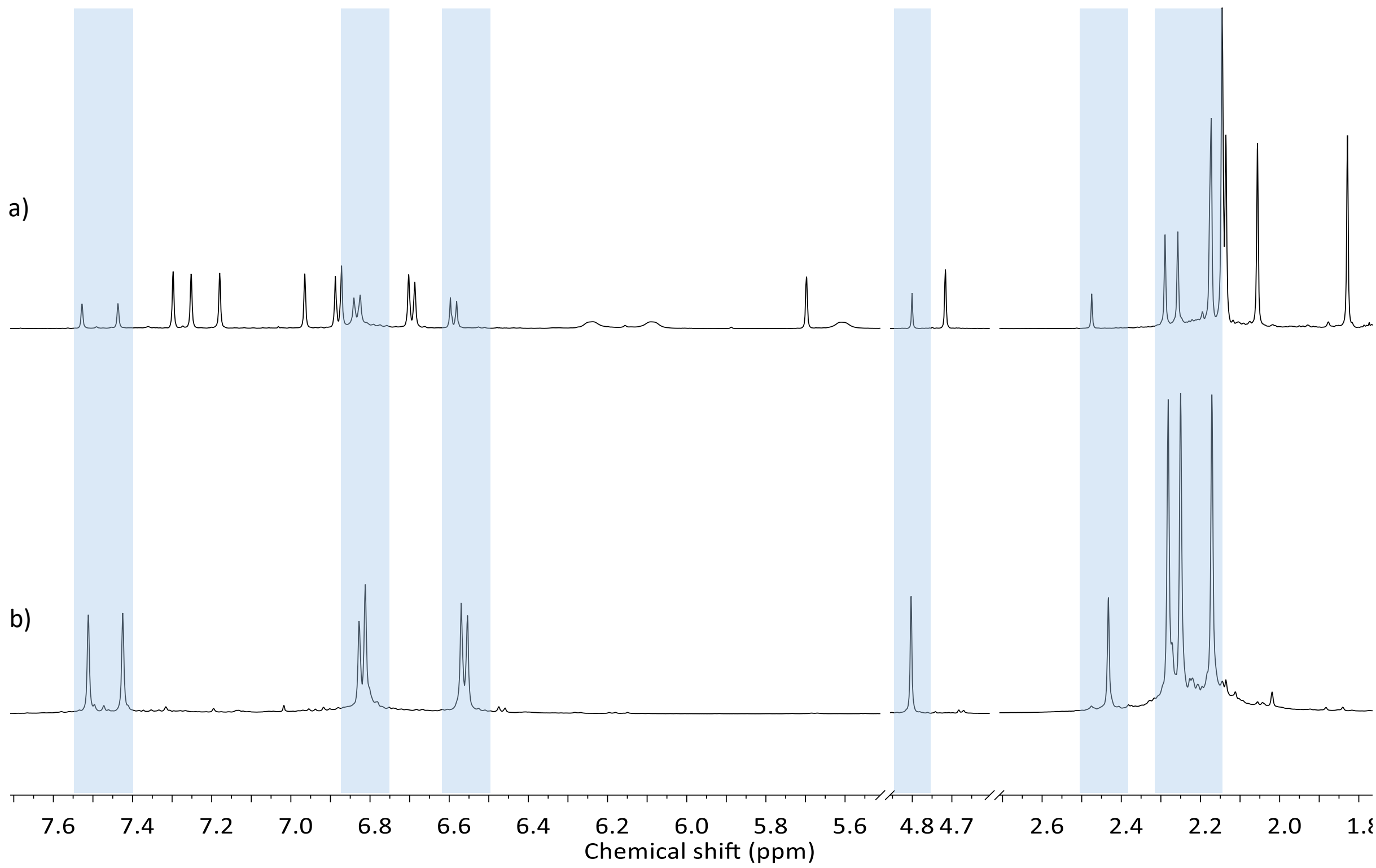

Figure S86. ^{1}H NMR spectra (500.2 MHz, THF-d_8) monitoring the reaction of K[**1^{H}**] with 1,2-di-*p*-tolylethyne in a sealed NMR tube: (a) reaction mixture after addition of K[**1^{H}**], showing K[**15**] as the major species together with resonances of **16a**; (b) same mixture after addition of $HNTf_2$, resulting in immediate decolorization and complete conversion of K[**15**] into **16a**.

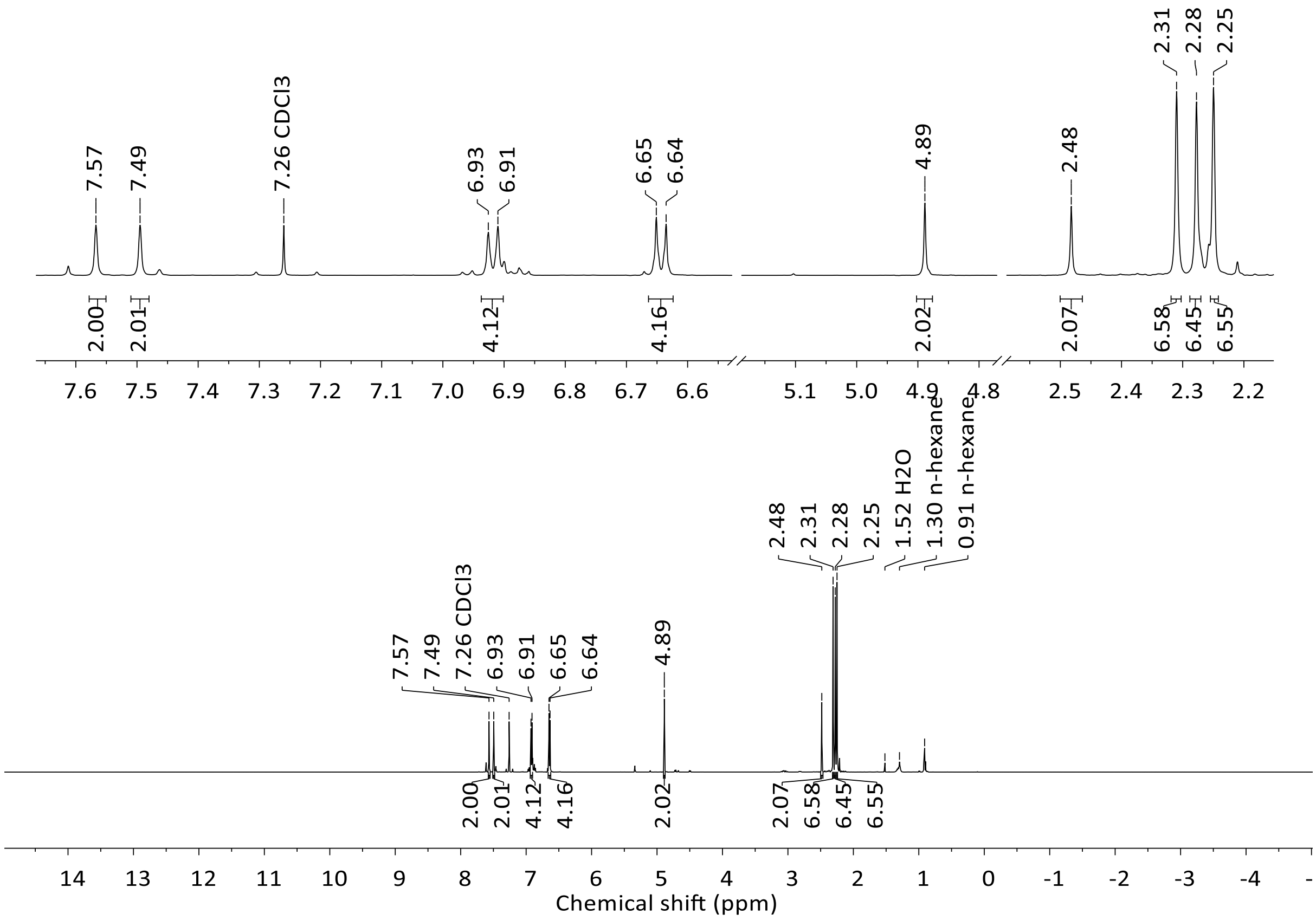

Figure S87. ^{1}H NMR spectrum (500.2 MHz, $CDCl_3$) of **16a**.

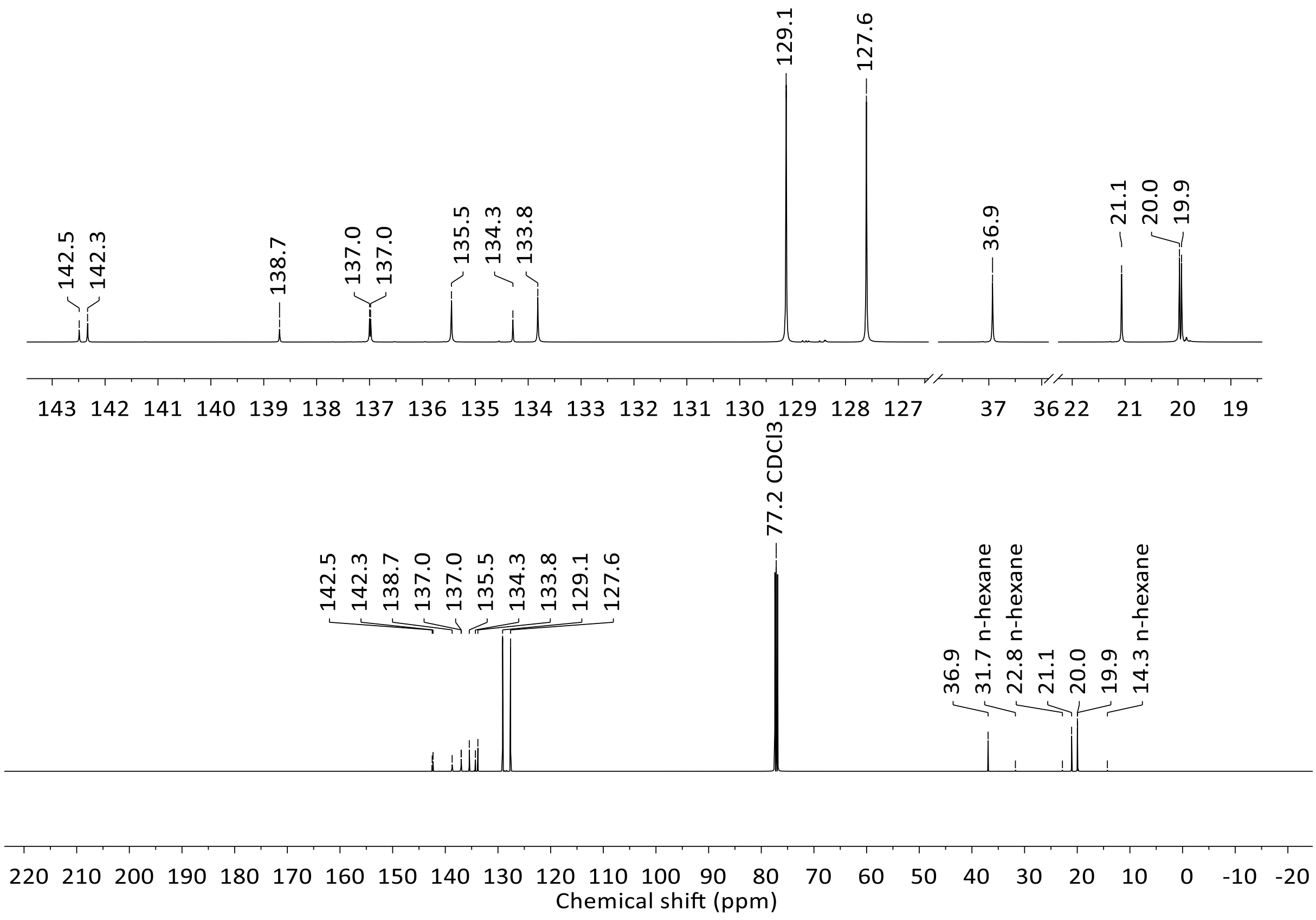

Figure S88. $^{13}C\{^{1}H\}$ NMR spectrum (125.8 MHz, $CDCl_3$) of **16a**.

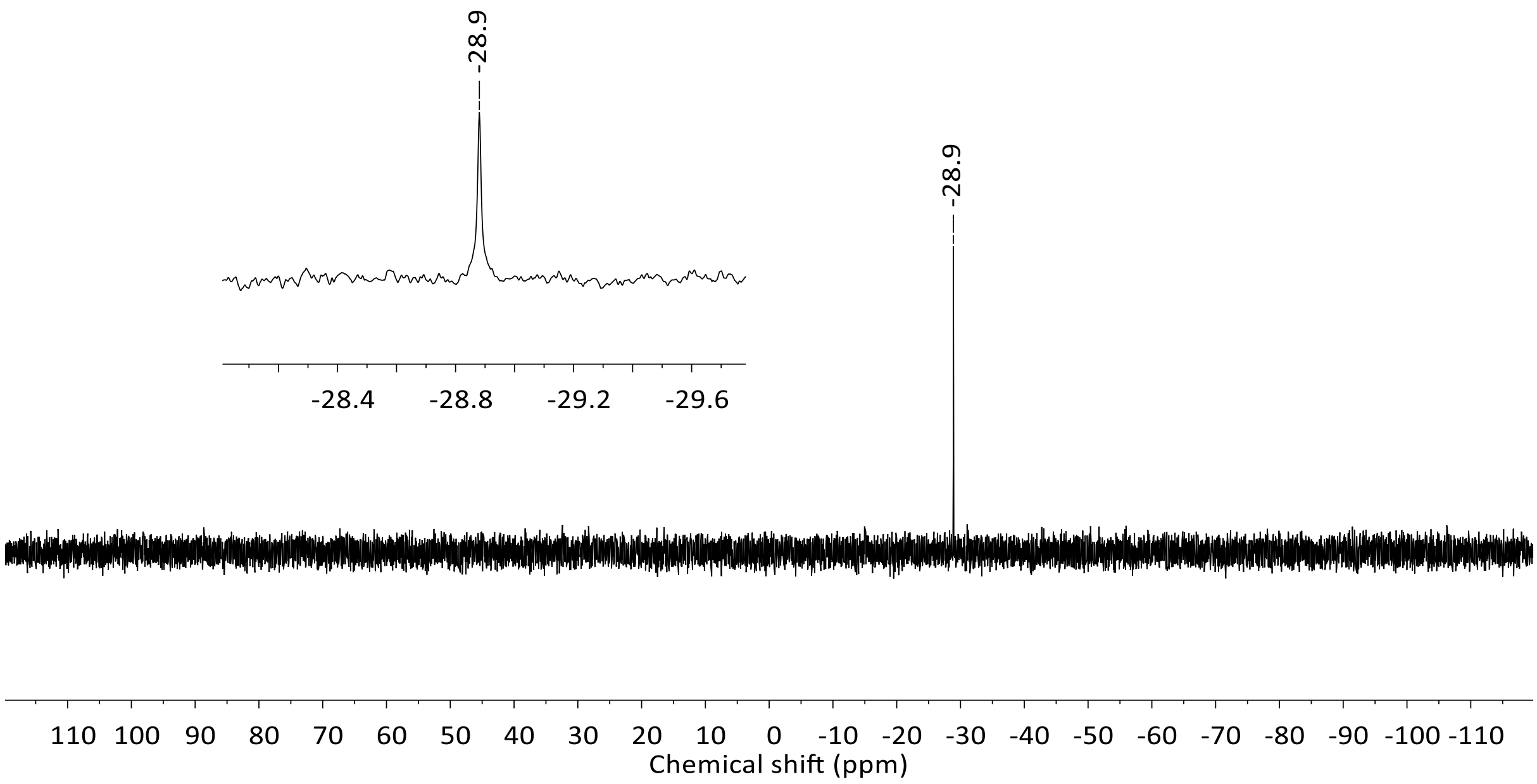

Figure S89. $^{29}Si\{^{1}H\}$ NMR spectrum (99.4 MHz, $CDCl_3$) of **16a**.

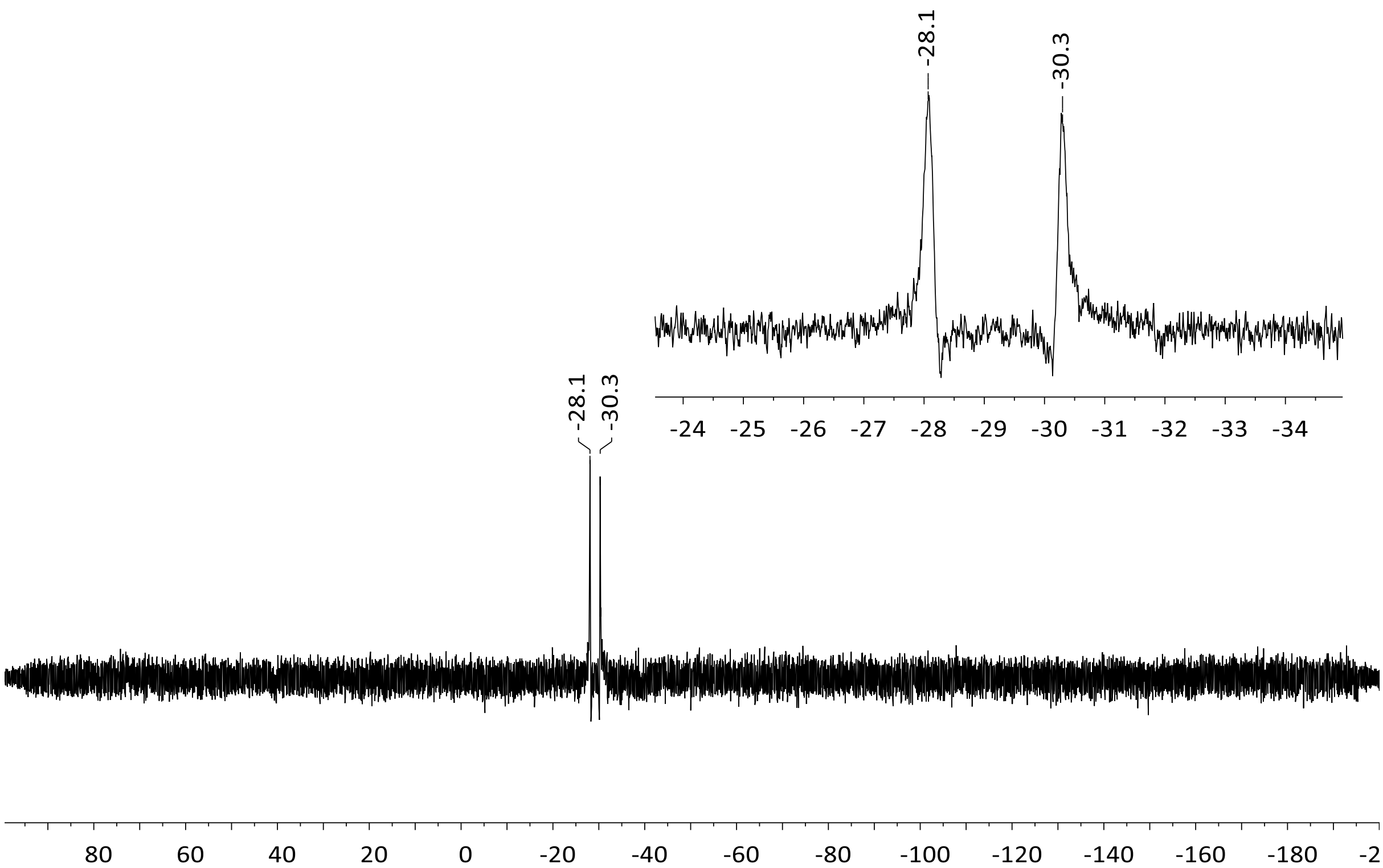

Figure S90. ^{29}Si NMR spectrum (99.4 MHz, $CDCl_3$) of **16a**.

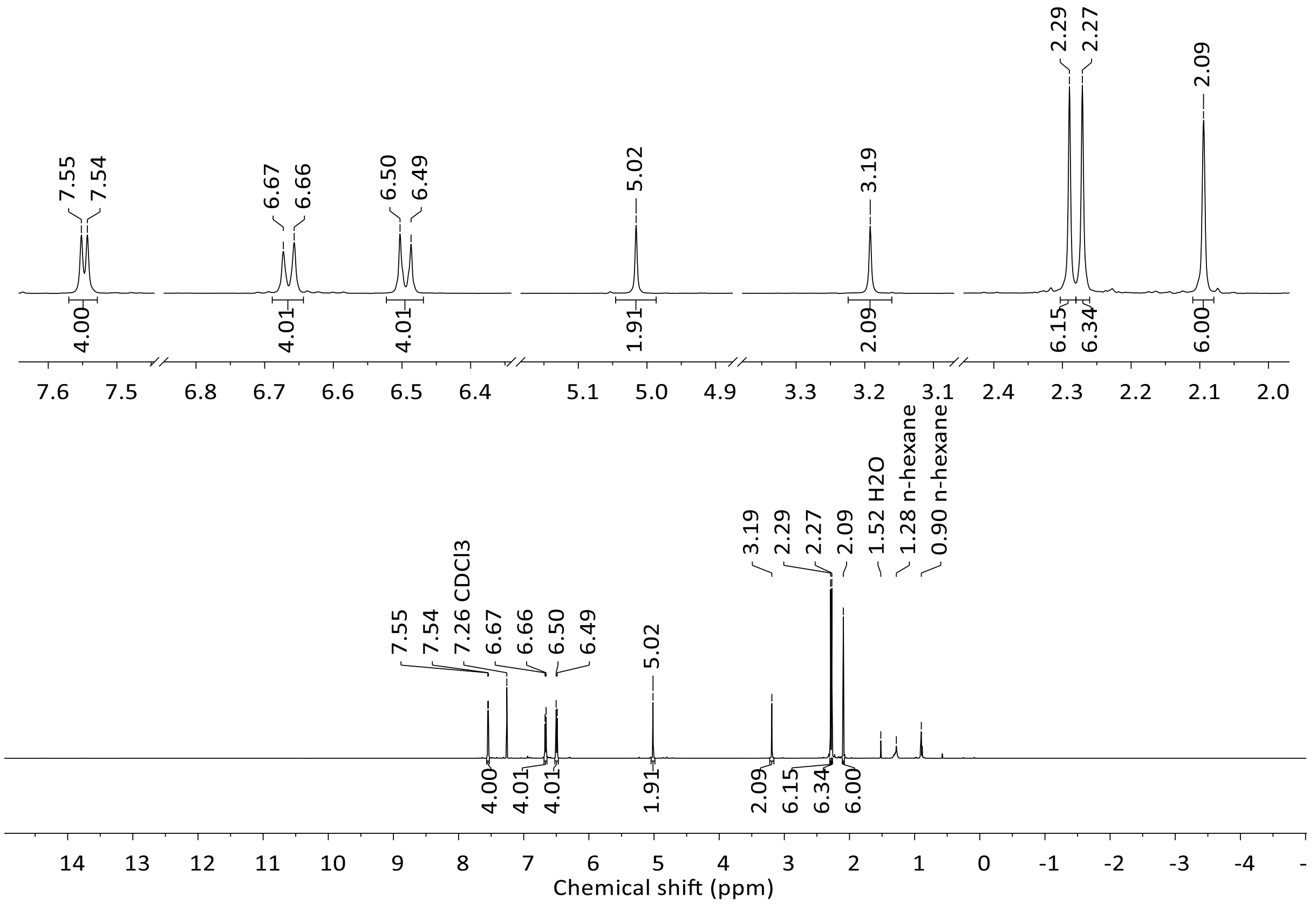

Figure S91. ^{1}H NMR spectrum (500.2 MHz, $CDCl_3$) of **16b**.

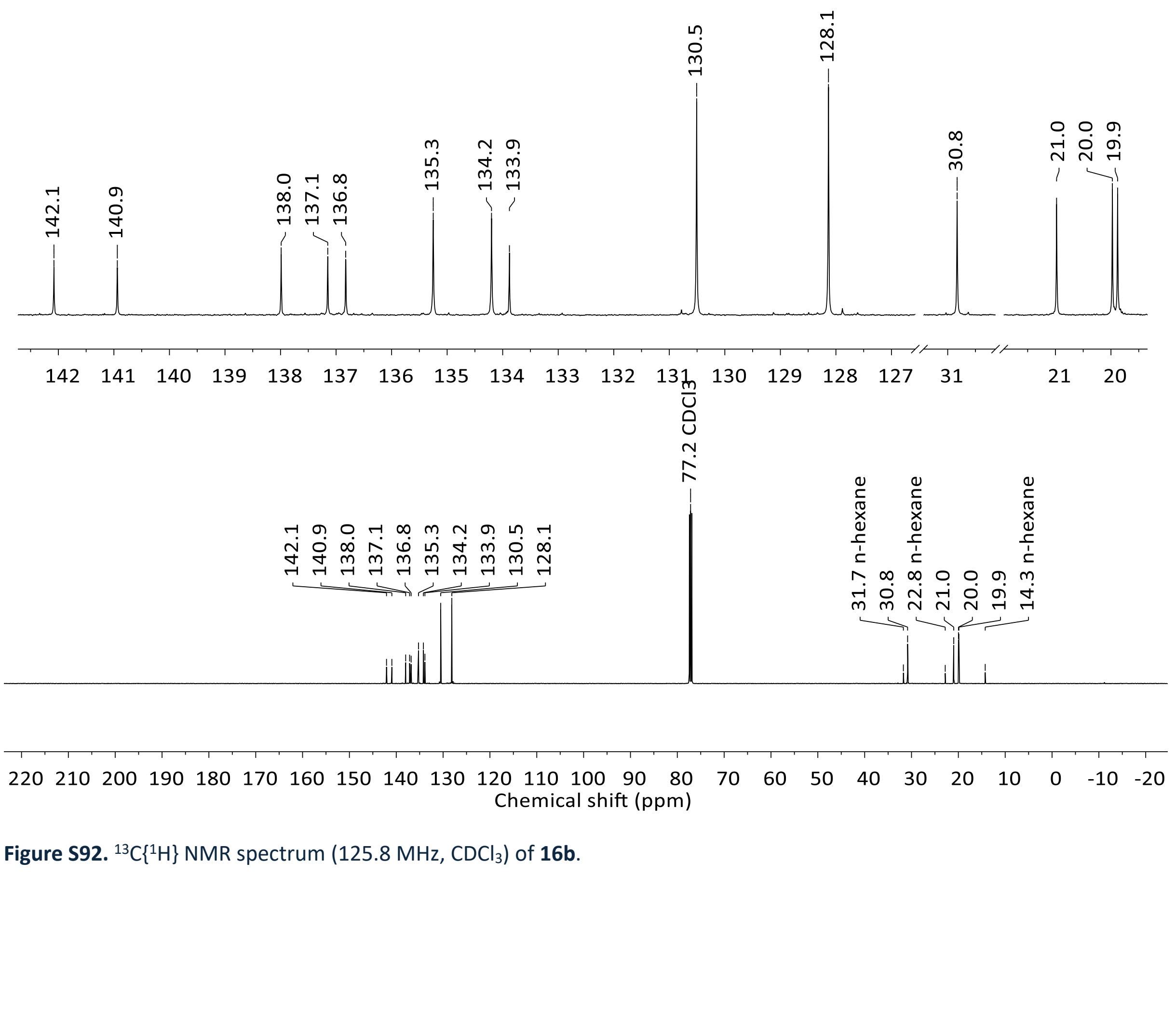

Figure S92. $^{13}C\{^{1}H\}$ NMR spectrum (125.8 MHz, $CDCl_3$) of **16b**.

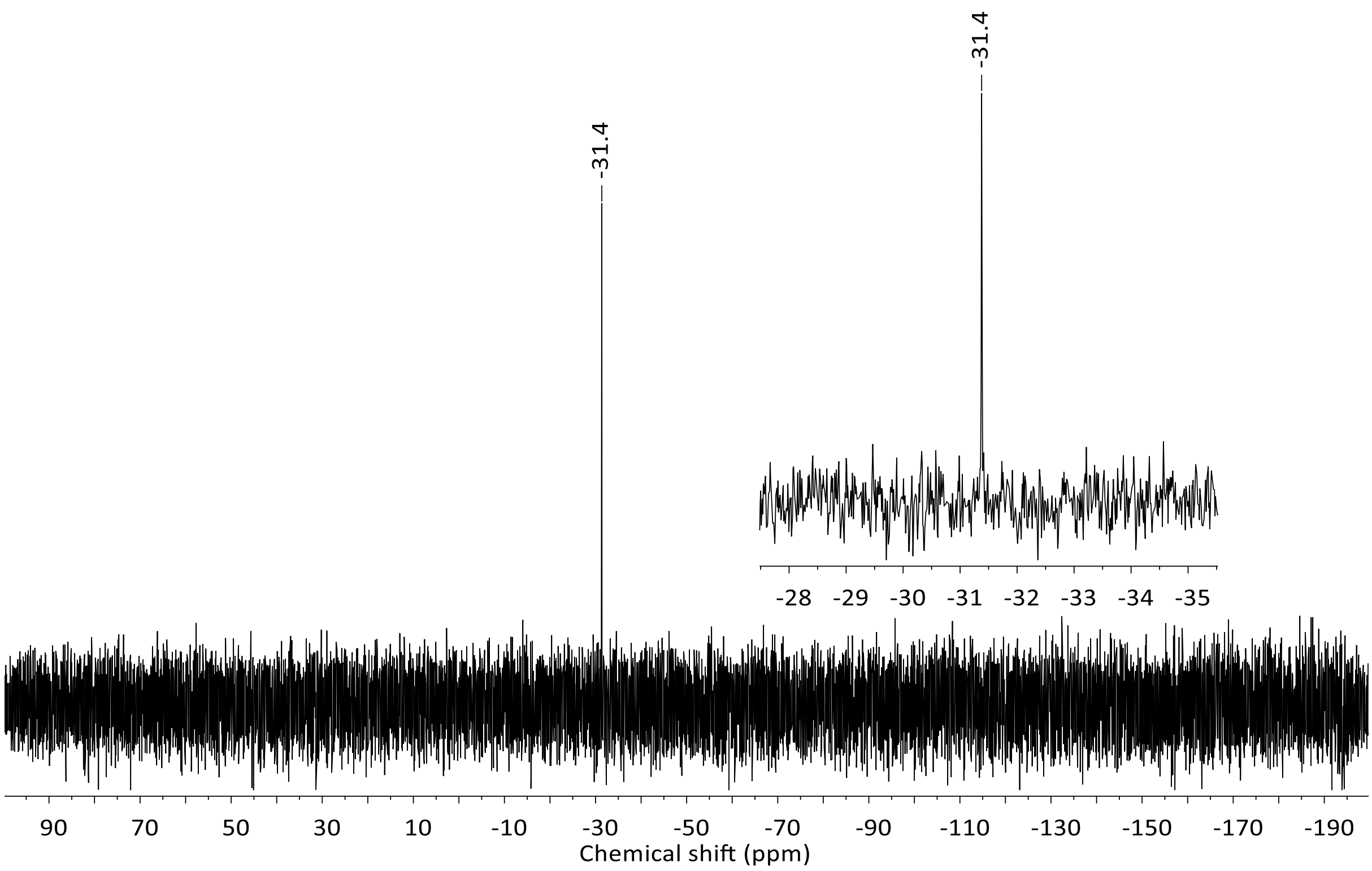

Figure S93. $^{29}Si\{^{1}H\}$ NMR spectrum (99.4 MHz, $CDCl_3$) of **16b**.

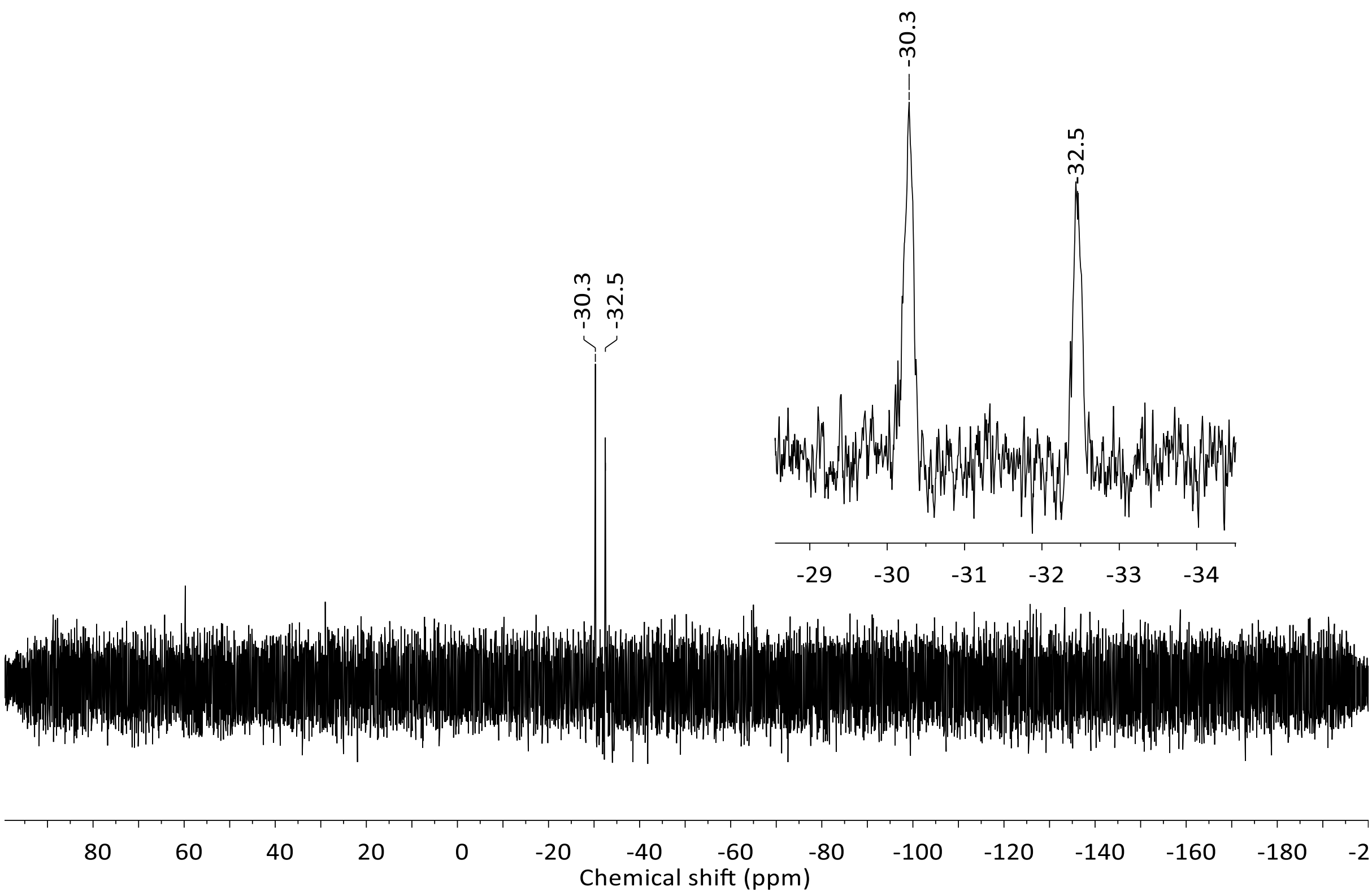

Figure S94. ^{29}Si NMR spectrum (99.4 MHz, $CDCl_3$) of **16b**.

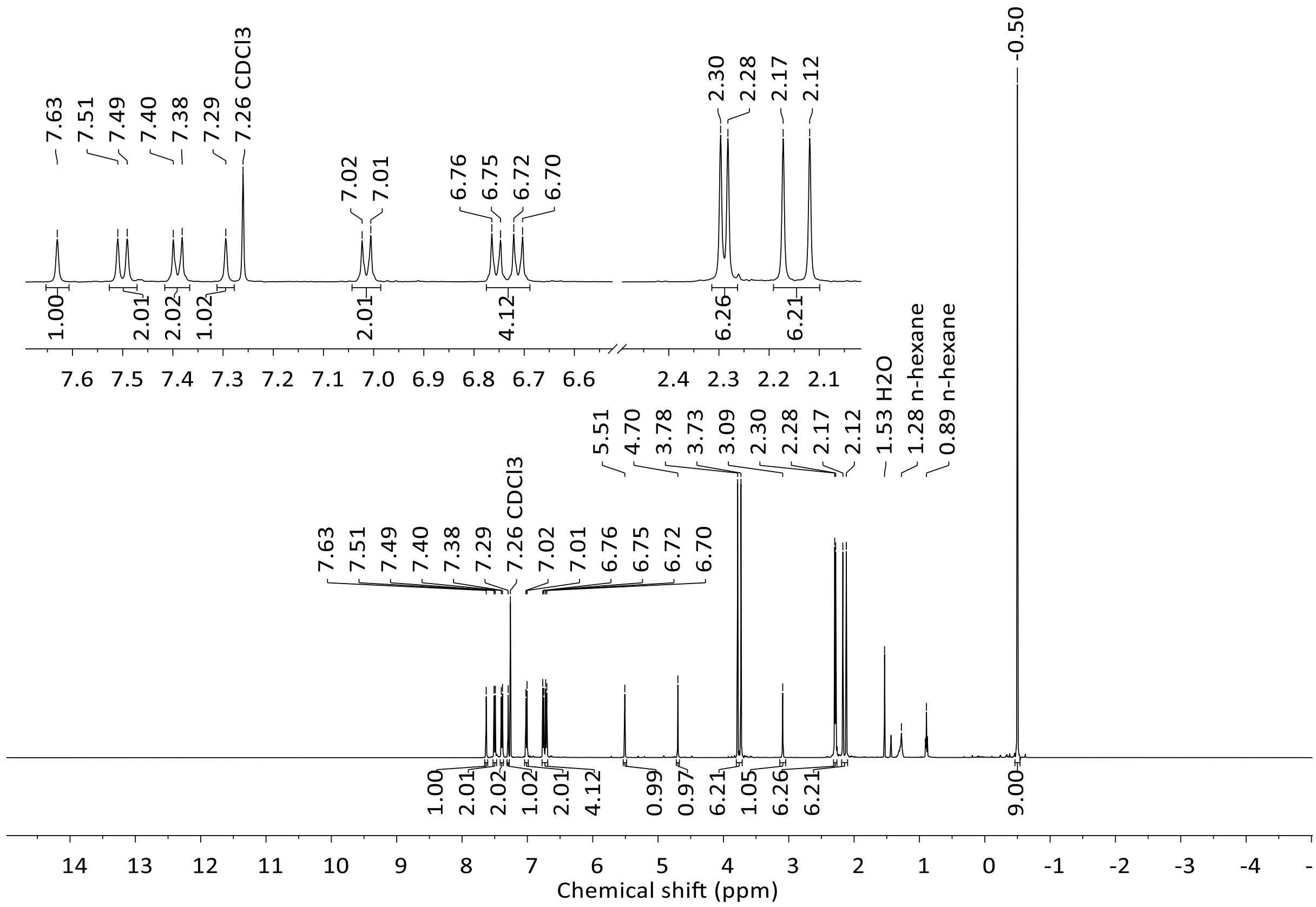

Figure S95. ^{1}H NMR spectrum (500.2 MHz, $CDCl_3$) of **16a^{Ans}**.

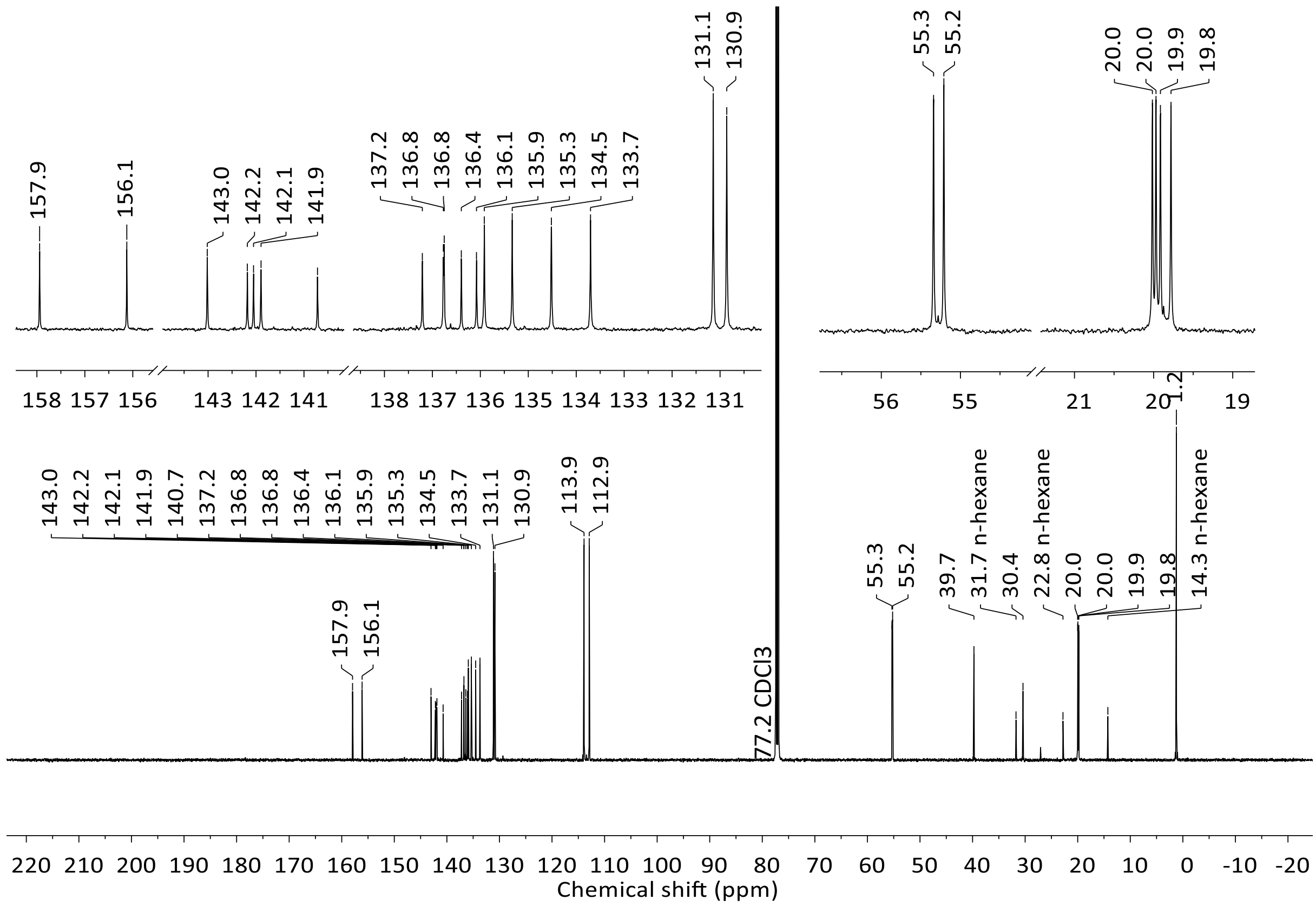

Figure S96. $^{13}C\{^{1}H\}$ NMR spectrum (125.8 MHz, $CDCl_3$) of **16a^{Ans}**.

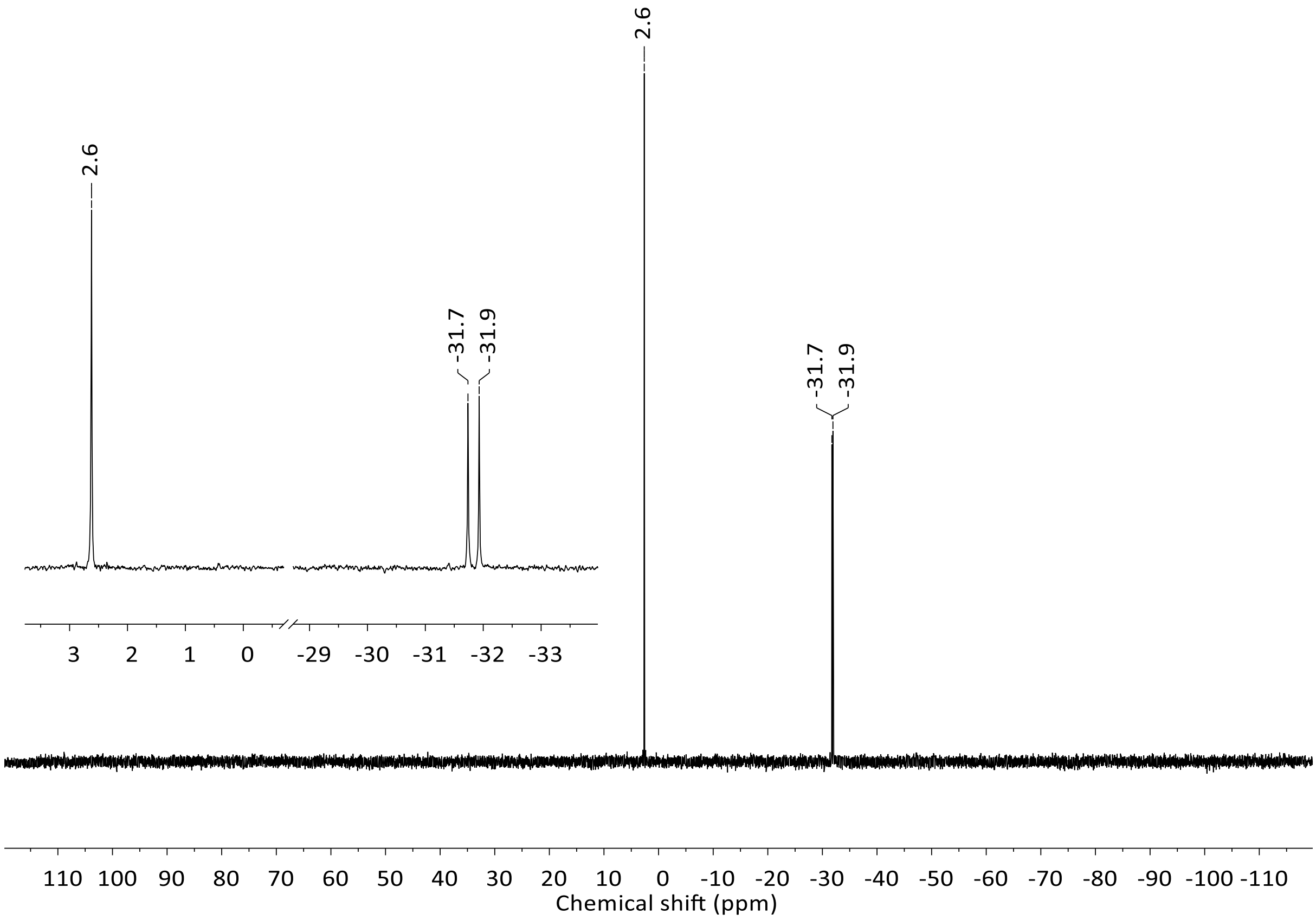

Figure S97. $^{29}Si\{^{1}H\}$ NMR spectrum (99.4 MHz, $CDCl_3$) of **16a^{Ans}**.

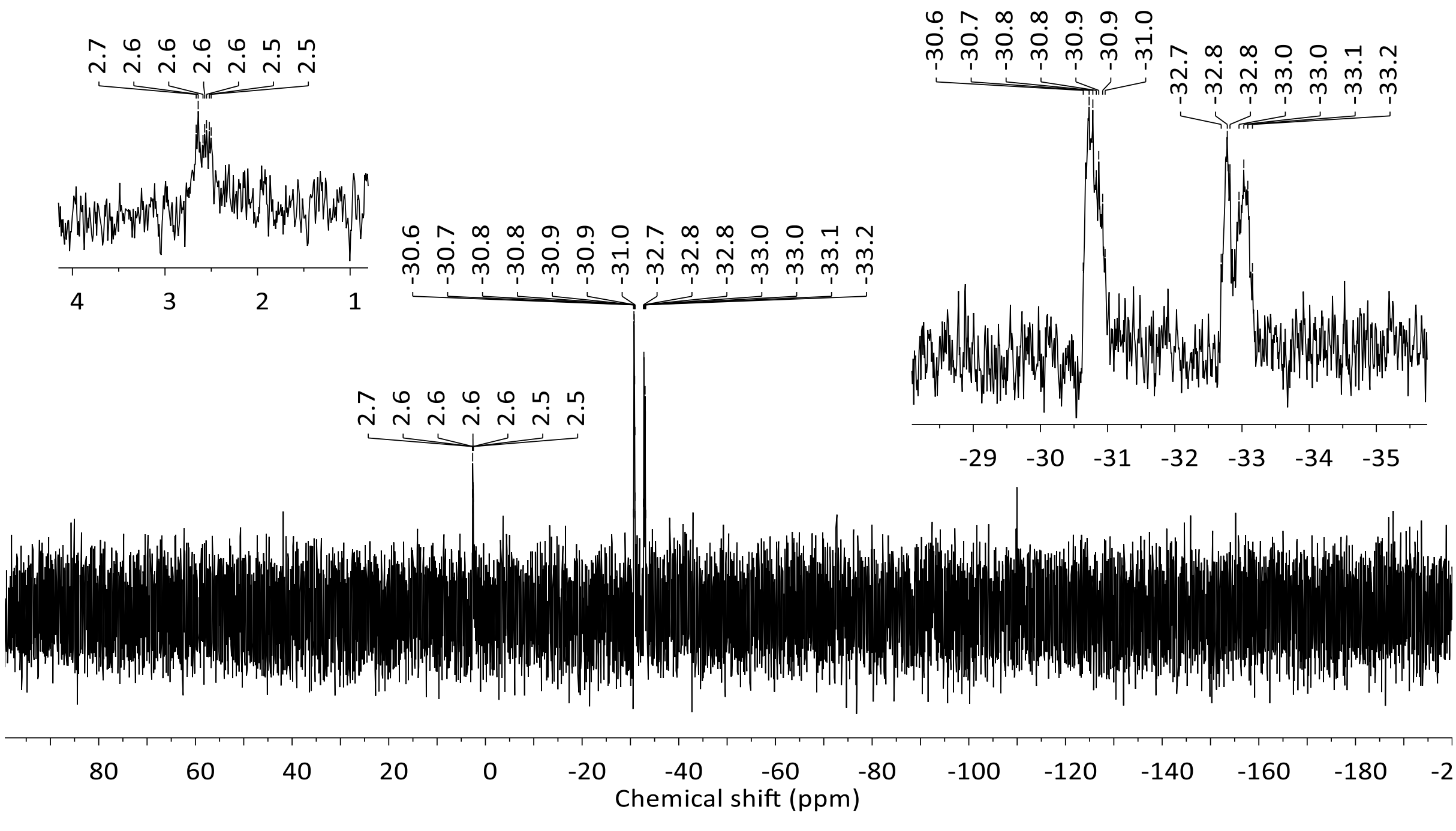

Figure S98. ^{29}Si NMR spectrum (99.4 MHz, $CDCl_3$) of **16a^{Ans}**.

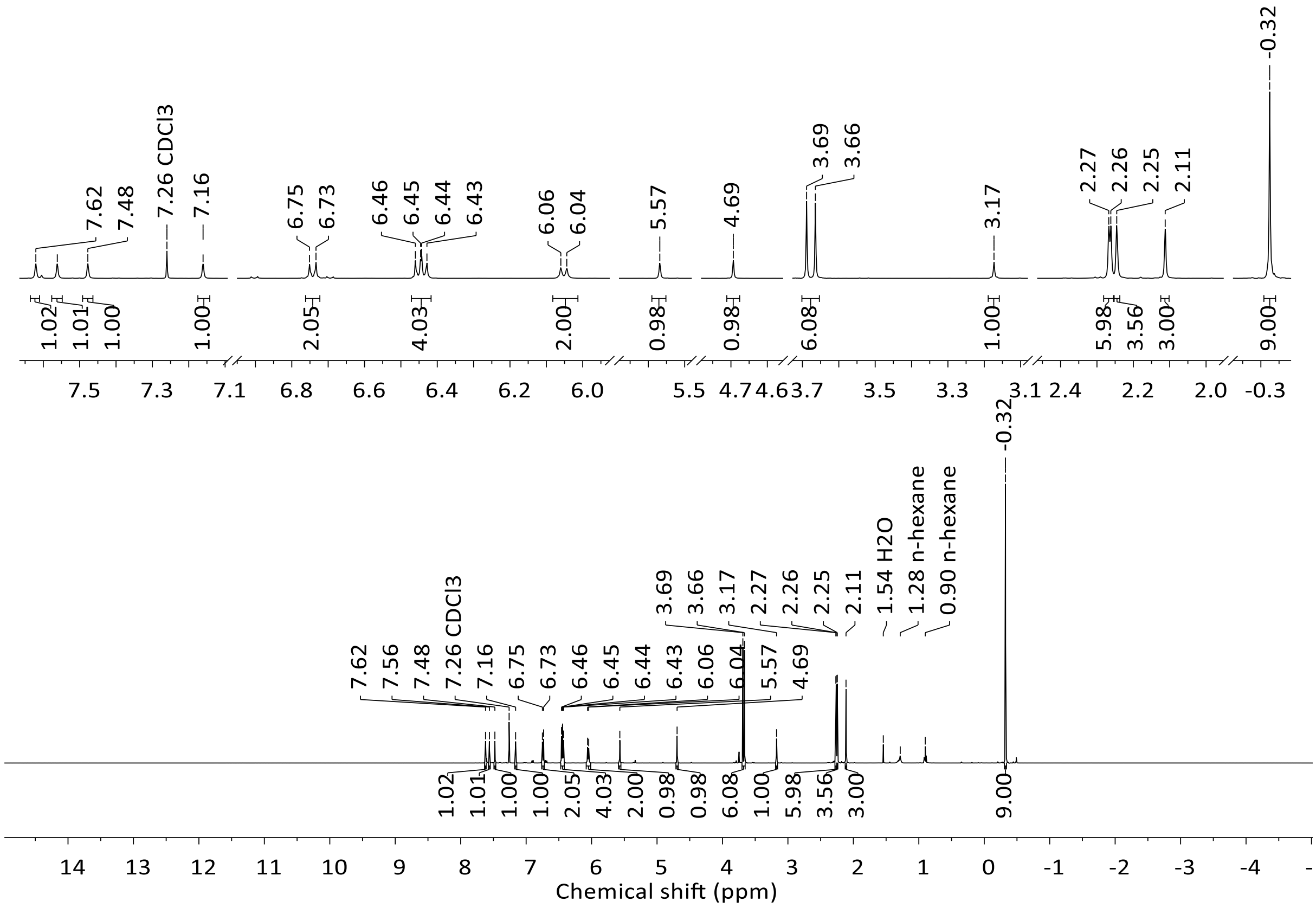

Figure S99. ^{1}H NMR spectrum (500.2 MHz, $CDCl_3$) of **16b^{Ans}**.

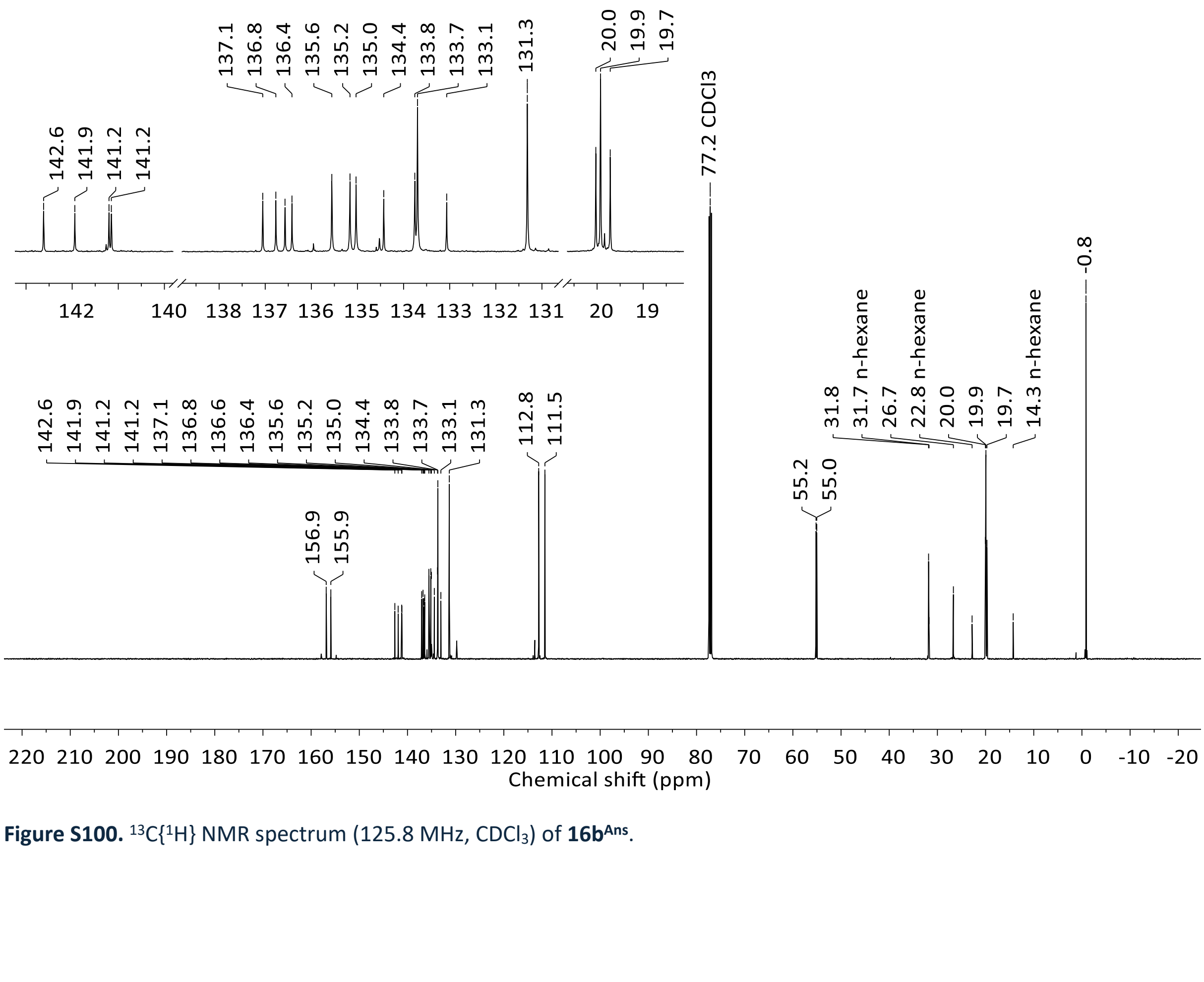

Figure S100. $^{13}C\{^{1}H\}$ NMR spectrum (125.8 MHz, $CDCl_3$) of **16b^{Ans}**.

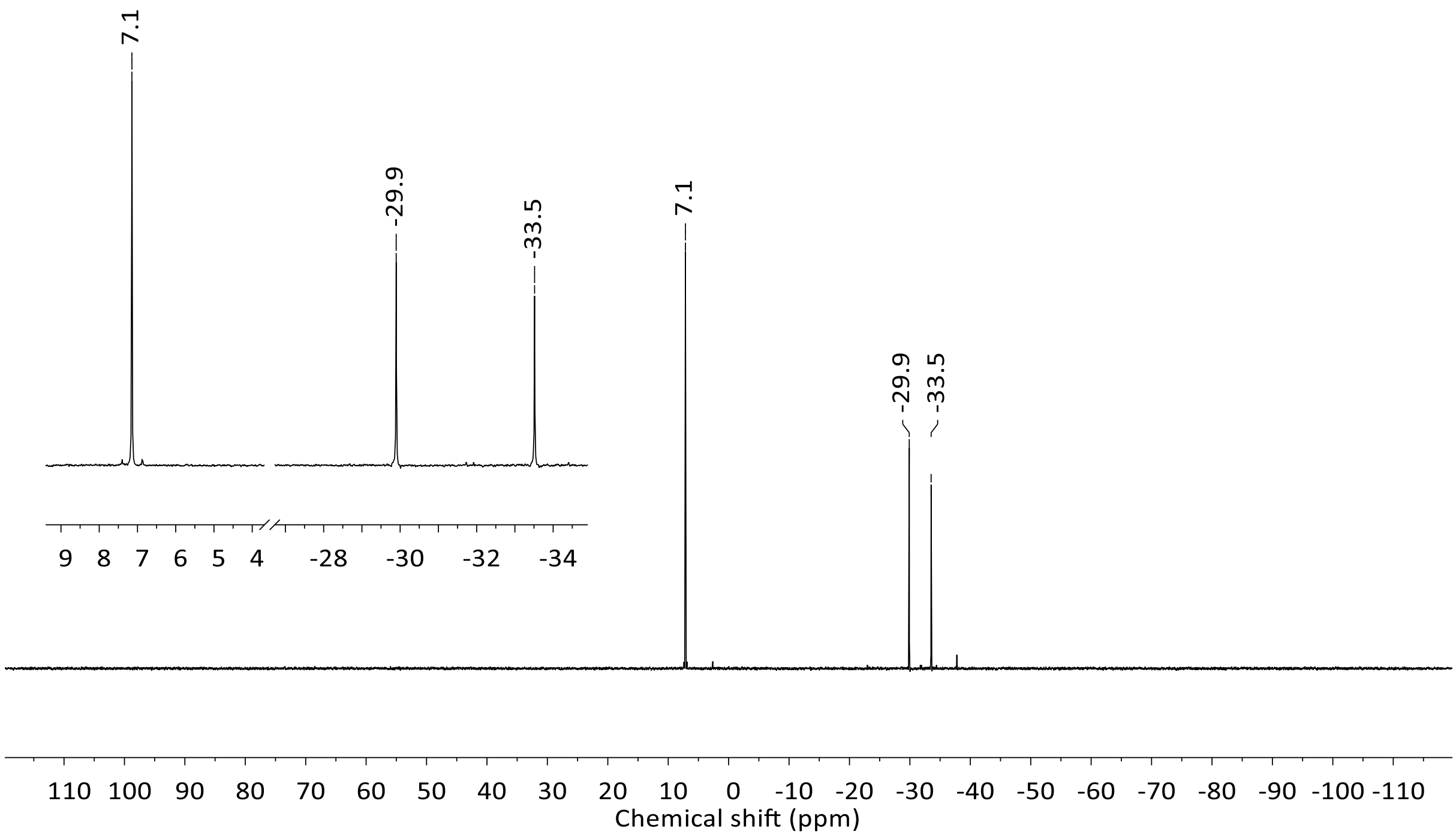

Figure S101. $^{29}Si\{^{1}H\}$ NMR spectrum (99.4 MHz, $CDCl_3$) of **16b^{Ans}**.

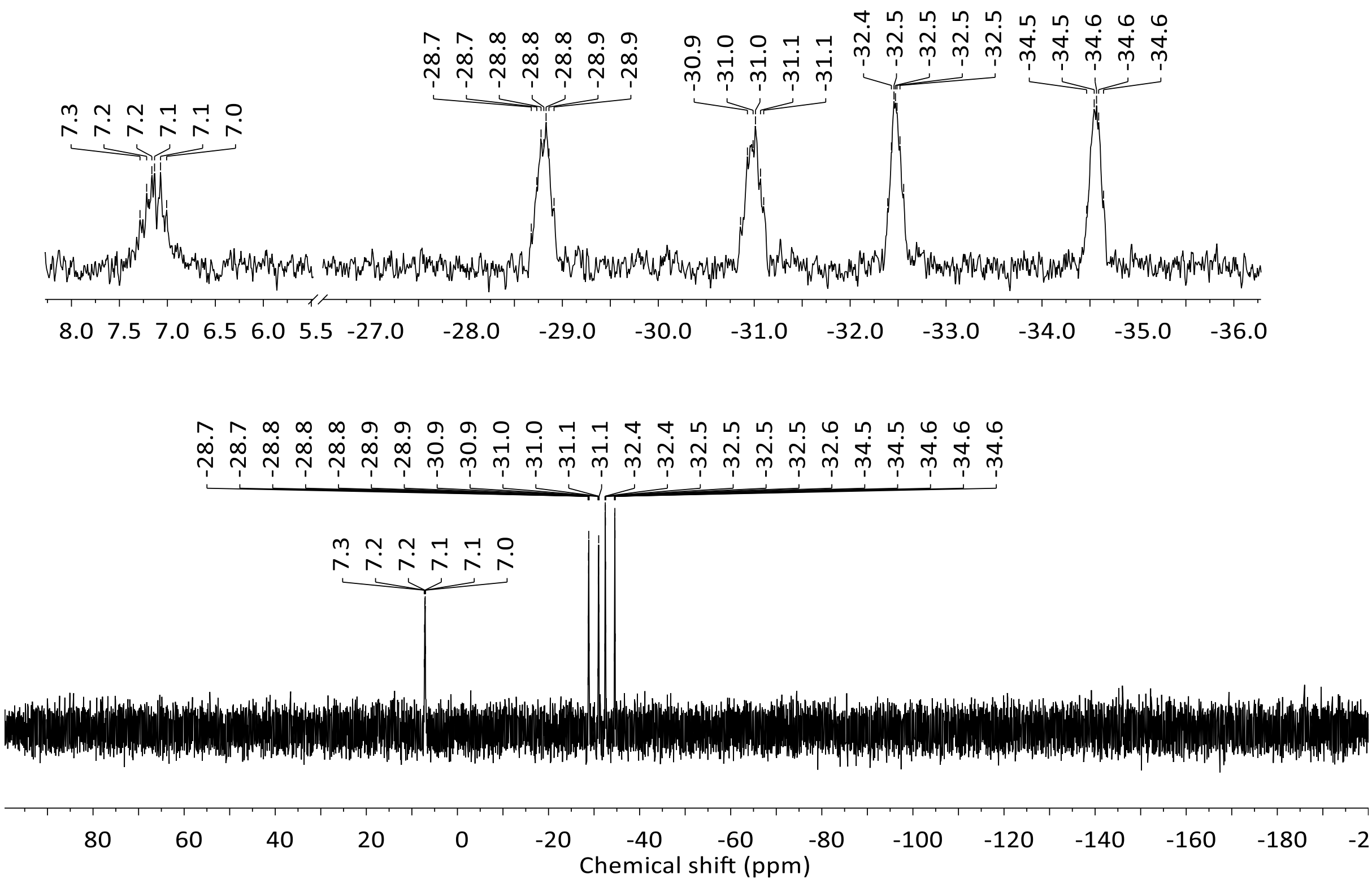

Figure S102. ^{29}Si NMR spectrum (99.4 MHz, $CDCl_3$) of **16b^{Ans}**.

3. Single-crystal X-ray structure analyses

Single-crystal diffraction data were collected on a *STOE IPDS II* two-circle diffractometer equipped with *Genix 3D HS* microfocus Mo *K*α X-ray source (λ = 0.71073 Å) at low temperature(s). The finalization of the data, including multi-scan empirical absorption corrections, was carried out using *CrysAlis*^{Pro} (version 1.171.42.43a, Rigaku Oxford Diffraction, 2022).

The structures were solved using the *SHELXT* program and refined against $|F|^2$ using full-matrix least-squares techniques with *SHELXL*-2018/3.[S14–S16] Further details on the structure refinement are provided in the corresponding sections for each crystal structure. All H atoms not bonded to Si were located geometrically and refined riding on the pivot atom; Si*H* atoms were located in the difference electron-density map and refined freely.

CIF files containing the crystallographic information are deposited in the Cambridge Crystallographic Data Centre under the deposition codes CCDC 2539949, 2539950, 2539952–2539962, and 2540095, and can be obtained free of charge via www.ccdc.cam.ac.uk/data_request/cif. Crystallographic data and parameters of the diffraction experiments are given in Table S3–Table S9.

Table S3. Selected crystallographic data and experimental details for $\mathbf{1^{2H}}$ and **VI**.

	$\mathbf{1^{2H}}$	**VI**
**Deposition code**	2539949	2539961
**Chemical formula**	$C_{16}H_{20}Si_2$	$C_{24}H_{30}Si_3$
M_r	268.50	402.75
**Crystal system, space group**	Triclinic, $P\bar{1}$	Monoclinic, *Cc*
**Temperature (K)**	100	100
a, b, c **(Å)**	5.4092(4), 7.5468(7), 9.7832(8)	14.1816(8), 18.1212(11), 9.1501(6)
**α, β, γ (°)**	67.638(8), 84.223(6), 86.444(6)	90, 100.864(6), 90
V **(Å³)**	367.34(6)	2309.3(2)
Z	1	4
F**(000)**	144	864
D_x **(Mg m⁻³)**	1.214	1.158
**Radiation type**	Mo $K\alpha$	Mo $K\alpha$
**μ (mm⁻¹)**	0.22	0.21
**Crystal shape**	Plate	Plate
**Color**	Colorless	Colorless
**Crystal size (mm)**	0.40 × 0.20 × 0.03	0.17 × 0.07 × 0.04
T_{min}, T_{max}	0.672, 1.000	0.840, 1.000
**No. of measured, independent and observed [$I > 2\sigma(I)$] reflections**	6037, 1620, 1548	9474, 4252, 3488
R_{int}	0.042	0.063
**θ values (°)**	θ_{max} = 27.1, θ_{min} = 2.3	θ_{max} = 26.0, θ_{min} = 2.3
**Range of** ***h, k, l***	$h = -6 \rightarrow 6$, $k = -9 \rightarrow 9$, $l = -12 \rightarrow 12$	$h = -17 \rightarrow 17$, $k = -22 \rightarrow 22$, $l = -10 \rightarrow 11$
$R[F^2 > 2\sigma(F^2)]$, $wR(F^2)$, S	0.037, 0.101, 1.15	0.046, 0.094, 0.99
**No. of reflections**	1620	4252
**No. of parameters**	92	274
**No. of restraints**	0	2
**H-atom treatment**	H atoms treated by a mixture of independent and constrained refinement	H atoms treated by a mixture of independent and constrained refinement
$\Delta\rho_{max}$, $\Delta\rho_{min}$ **(e Å⁻³)**	0.37, –0.21	0.29, –0.20
**Absolute structure parameter**	–	–0.05(13)

Computer programs: *X-AREA* (Stoe & Cie, 2001), *CrysAlis^{Pro}* 1.171.42.43a (Rigaku OD, 2022), *SHELXS* (G. M. Sheldrick, 1997), *SHELXT* (G. M. Sheldrick, 2015), *SHELXL-2018*/3 (Sheldrick, 2018), IUCr *publCIF* online service.

Table S4. Selected crystallographic data and experimental details for **VII** and K_2[**1**].

	**VII**	**K_2[1]**
**Deposition code**	2539962	2539950
**Chemical formula**	$C_{24}H_{26}Si_2$	$C_{116}H_{176}K_8O_{13}Si_8 \cdot C_{112}H_{168}K_8O_{12}Si_8$
**M_r**	370.63	570.01
**Crystal system, space group**	Monoclinic, *C*2/*c*	Triclinic, *P*1
**Temperature (K)**	173	173
a, b, c **(Å)**	16.9680(12), 9.7502(6), 14.1126(13)	23.9433(8), 24.0823(9), 27.0416(10)
**α, β, γ (°)**	90, 107.578(9), 90	71.137(3), 84.030(3), 61.603(4)
V **(Å^3)**	2225.8(3)	12956.5(9)
Z	4	16
F**(000)**	792	4880
**D_x (Mg m^{-3})**	1.106	1.169
**Radiation type**	Mo *K*α	Mo *K*α
**μ (mm^{-1})**	0.16	0.39
**Crystal shape**	Block	Elongated prism
**Color**	Colorless	Orange
**Crystal size (mm)**	0.14 × 0.11 × 0.07	0.34 × 0.17 × 0.11
**T_{min}, T_{max}**	0.659, 1.000	0.528, 1.000
**No. of measured, independent and observed [$I > 2\sigma(I)$] reflections**	14573, 2291, 2024	105388, 47168, 27723
**R_{int}**	0.085	0.072
**θ values (°)**	θ_{max} = 26.4, θ_{min} = 2.4	θ_{max} = 25.4, θ_{min} = 2.1
**Range of *h, k, l***	*h* = –19→21, *k* = –12→12, *l* = –17→17	*h* = –28→27, *k* = –29→28, *l* = –32→32
**$R[F^2 > 2\sigma(F^2)]$, $wR(F^2)$, S**	0.041, 0.108, 1.06	0.068, 0.204, 1.01
**No. of reflections**	2291	47168
**No. of parameters**	125	2629
**No. of restraints**	0	175
**H-atom treatment**	H atoms treated by a mixture of independent and constrained refinement	H atoms treated by a mixture of independent and constrained refinement
**$\Delta\rho_{max}$, $\Delta\rho_{min}$ (e Å^{-3})**	0.45, –0.21	0.78, –0.54

Computer programs: *X-AREA* (Stoe & Cie, 2001), *CrysAlis^{Pro}* 1.171.42.43a (Rigaku OD, 2022), *SHELXS* (G. M. Sheldrick, 1997), *SHELXT* (G. M. Sheldrick, 2015), *SHELXL-2018*/3 (Sheldrick, 2018).

Table S5. Selected crystallographic data and experimental details for K_2[**10**] and [K(18-c-6)(THF)$_2$][K(18-c-6)]$_3$[**10**]$_2$.

	K_2[**10**]	**[K(18-c-6)(THF)$_2$][K(18-c-6)]$_3$[10]$_2$**
**Deposition code**	2540095	2539952
**Chemical formula**	$C_{30}H_{32}B_2K_2Si_2$	$C_{61.23}H_{94.58}B_2K_2O_{13.73}Si_2$
M_r	548.55	1206.35
**Crystal system, space group**	Cubic, *Im*3*m*	Triclinic, *P*1
**Temperature (K)**	173	220
***a*, *b*, *c* (Å)**	17.2620(8), 17.2620(8), 17.2620(8)	15.2741(11), 17.7365(15), 28.893(2)
**α, β, γ (°)**	90, 90, 90	98.515(7), 91.616(6), 91.820(6)
***V* (Å^3)**	5143.7(7)	7732.9(11)
Z	6	4
***F*(000)**	1728	2591
**D_x (Mg m^{-3})**	1.063	1.036
**Radiation type**	Mo *K*α	Mo *K*α
**μ (mm^{-1})**	0.36	0.20
**Crystal shape**	Prism	Plate
**Color**	Orange	Orange
**Crystal size (mm)**	0.20 × 0.16 × 0.10	0.14 × 0.11 × 0.04
**T_{min}, T_{max}**	0.540, 1.000	0.541, 1.000
**No. of measured, independent and observed [$I > 2\sigma(I)$] reflections**	6860, 298, 240	38781, 18703, 10819
**R_{int}**	0.064	0.061
**θ values (°)**	θ_{max} = 20.7, θ_{min} = 3.3	θ_{max} = 22.0, θ_{min} = 2.5
**Range of *h*, *k*, *l***	*h* = −14→17, *k* = −15→17, *l* = −17→17	*h* = −15→16, *k* = −18→18, *l* = −30→30
**$R[F^2 > 2\sigma(F^2)]$, $wR(F^2)$, *S***	0.096, 0.332, 1.56	0.088, 0.290, 1.04
**No. of reflections**	298	18703
**No. of parameters**	42	2026
**No. of restraints**	1	870
**H-atom treatment**	H atoms treated by a mixture of independent and constrained refinement	H atom parameters constrained
**$\Delta\rho_{max}$, $\Delta\rho_{min}$ (e Å^{-3})**	0.25, −0.30	0.79, −0.35

Computer programs: *X-AREA* (Stoe & Cie, 2001), *CrysAlis^{Pro}* 1.171.42.43a (Rigaku OD, 2022), *SHELXS* (G. M. Sheldrick, 1997), *SHELXT* (G. M. Sheldrick, 2015), *SHELXL-2018*/3 (Sheldrick, 2018).

Table S6. Selected crystallographic data and experimental details for *cis*-**2** and *cis*-**3**.

	cis-**2**	*cis*-**3**
**Deposition code**	2539953	2539954
**Chemical formula**	$C_{22}H_{36}Si_4$	$C_{32}H_{56}Si_4$
M_r	412.87	553.12
**Crystal system, space group**	Monoclinic, *C*2/*c*	Monoclinic, $P2_1/c$
**Temperature (K)**	100	173
a, b, c **(Å)**	36.5257(17), 14.1632(6), 27.1547(11)	13.5635(3), 17.8643(3), 15.2759(3)
**α, β, γ (°)**	90, 114.373(4), 90	90, 107.420(2), 90
V **(Å^3)**	12795.6(10)	3531.64(13)
Z	20	4
$F(000)$	4480	1216
D_x **(Mg m^{-3})**	1.072	1.040
**Radiation type**	Mo *K*α	Mo *K*α
**μ (mm^{-1})**	0.24	0.19
**Crystal shape**	Block	Plate
**Color**	Colorless	Colorless
**Crystal size (mm)**	0.90 × 0.22 × 0.20	0.21 × 0.20 × 0.11
T_{min}, T_{max}	0.904, 1.000	0.908, 1.000
**No. of measured, independent and observed [$I > 2\sigma(I)$] reflections**	34538, 13558, 9765	39515, 7782, 6782
R_{int}	0.067	0.069
**θ values (°)**	$\theta_{max} = 26.7$, $\theta_{min} = 2.5$	$\theta_{max} = 27.1$, $\theta_{min} = 2.7$
**Range of *h*, *k*, *l***	$h = -39 \rightarrow 46$, $k = -15 \rightarrow 17$, $l = -34 \rightarrow 34$	$h = -17 \rightarrow 17$, $k = -22 \rightarrow 22$, $l = -18 \rightarrow 19$
$R[F^2 > 2\sigma(F^2)]$, $wR(F^2)$, S	0.046, 0.114, 1.01	0.041, 0.114, 1.06
**No. of reflections**	13558	7782
**No. of parameters**	631	361
**No. of restraints**	0	0
**H-atom treatment**	H atoms treated by a mixture of independent and constrained refinement	H atoms treated by a mixture of independent and constrained refinement
$\Delta\rho_{max}$, $\Delta\rho_{min}$ **(e Å^{-3})**	0.42, –0.26	0.41, –0.22

Computer programs: *X-AREA* (Stoe & Cie, 2001), *CrysAlis^{Pro}* 1.171.42.43a (Rigaku OD, 2022), *SHELXS* (G. M. Sheldrick, 1997), *SHELXT* (G. M. Sheldrick, 2015), *SHELXL-2018*/3 (Sheldrick, 2018).

Table S7. Selected crystallographic data and experimental details for **4** and **5**.

	**4**	**5**
**Deposition code**	2539955	2539956
**Chemical formula**	$C_{20}H_{30}Si_4$	$C_{36}H_{48}Si_6$
M_r	382.80	649.28
**Crystal system, space group**	Triclinic, *P*1̄	Monoclinic, $P2_1/c$
**Temperature (K)**	173	173
a, b, c **(Å)**	7.6394(4), 7.8172(3), 19.3948(8)	10.0861(8), 17.8299(14), 10.8684(13)
**α, β, γ (°)**	90.982(3), 94.505(4), 108.332(4)	90, 107.929(10), 90
V **(Å^3)**	1094.98(9)	1859.6(3)
Z	2	2
F**(000)**	412	696
D_x **(Mg m^{-3})**	1.161	1.160
**Radiation type**	Mo *K*α	Mo *K*α
**μ (mm^{-1})**	0.27	0.25
**Crystal shape**	Plate	Prism
**Color**	Colorless	Colorless
**Crystal size (mm)**	0.24 × 0.13 × 0.04	0.10 × 0.08 × 0.06
T_{min}, T_{max}	0.583, 1.000	0.914, 1.000
**No. of measured, independent and observed [$I > 2\sigma(I)$] reflections**	10077, 4936, 3953	11857, 3661, 1455
R_{int}	0.040	0.088
**θ values (°)**	θ_{max} = 27.5, θ_{min} = 2.8	θ_{max} = 26.0, θ_{min} = 2.7
**Range of *h, k, l***	*h* = –9→9, *k* = –10→9, *l* = –25→25	*h* = –12→12, *k* = –22→22, *l* = –13→12
$R[F^2 > 2\sigma(F^2)]$, $wR(F^2)$, S	0.052, 0.147, 1.02	0.036, 0.059, 0.57
**No. of reflections**	4936	3661
**No. of parameters**	233	204
**No. of restraints**	0	0
**H-atom treatment**	H atoms treated by a mixture of independent and constrained refinement	H atoms treated by a mixture of independent and constrained refinement
$\Delta\rho_{max}$, $\Delta\rho_{min}$ **(e Å^{-3})**	1.28, –0.48	0.27, –0.21

Computer programs: *X-AREA* (Stoe & Cie, 2001), *CrysAlis^{Pro}* 1.171.42.43a (Rigaku OD, 2022), *SHELXS* (G. M. Sheldrick, 1997), *SHELXT* (G. M. Sheldrick, 2015), *SHELXL-2018*/3 (Sheldrick, 2018).

Table S8. Selected crystallographic data and experimental details for **6** and **7**.

	**6**	**7**
**Deposition code**	2539957	2539958
**Chemical formula**	$C_{19}H_{28}Si_3$	$C_{36.60}H_{51.20}Si_6$
M_r	340.68	659.71
**Crystal system, space group**	Orthorhombic, $P2_12_12_1$	Triclinic, $P1$
**Temperature (K)**	173	173
a, b, c **(Å)**	7.3850(2), 14.8354(3), 19.1227(4)	8.9789(13), 9.8351(15), 12.085(2)
**α, β, γ (°)**	90, 90, 90	74.076(15), 69.820(15), 73.842(13)
V **(Å^3)**	2095.07(8)	943.1(3)
Z	4	1
F**(000)**	736	355
D_x **(Mg m^{-3})**	1.080	1.162
**Radiation type**	Mo *K*α	Mo *K*α
**μ (mm^{-1})**	0.22	0.25
**Crystal shape**	Irregular shape	Elongated plate
**Color**	Colorless	Colorless
**Crystal size (mm)**	0.47 × 0.35 × 0.32	0.25 × 0.08 × 0.04
T_{min}, T_{max}	0.867, 1.000	0.577, 1.000
**No. of measured, independent and observed [$I > 2\sigma(I)$] reflections**	26457, 4629, 4534	4681, 2286, 1553
R_{int}	0.057	0.056
**θ values (°)**	θ_{max} = 27.2, θ_{min} = 2.5	θ_{max} = 22.0, θ_{min} = 2.5
**Range of *h, k, l***	$h = -9 \rightarrow 9$, $k = -19 \rightarrow 17$, $l = -24 \rightarrow 24$	$h = -9 \rightarrow 9$, $k = -10 \rightarrow 10$, $l = -12 \rightarrow 12$
$R[F^2 > 2\sigma(F^2)]$, $wR(F^2)$, S	0.032, 0.088, 1.04	0.052, 0.120, 1.01
**No. of reflections**	4629	2286
**No. of parameters**	218	212
**No. of restraints**	0	0
**H-atom treatment**	H atoms treated by a mixture of independent and constrained refinement	H atoms treated by a mixture of independent and constrained refinement
$\Delta\rho_{max}$, $\Delta\rho_{min}$ **(e Å^{-3})**	0.32, –0.16	0.27, –0.20
**Absolute structure parameter**	0.05(5)	–

Computer programs: *X-AREA* (Stoe & Cie, 2001), *CrysAlis^{Pro}* 1.171.42.43a (Rigaku OD, 2022), *SHELXS* (G. M. Sheldrick, 1997), *SHELXT* (G. M. Sheldrick, 2015), *SHELXL-2018*/3 (Sheldrick, 2018).

Table S9. Selected crystallographic data and experimental details for **13** and K[**15**].

	**13**	K[**15**]
**Deposition code**	2539959	2539960
**Chemical formula**	$C_{30}H_{28}Si_2$	$C_{34}H_{37}KO_{0.5}Si_2{\cdot}(C_4H_8O)_{0.2}{\cdot}(C_4H_{10}O)_{0.175}$
***M*_r**	444.70	576.31
**Crystal system, space group**	Monoclinic, $P2_1/c$	Monoclinic, $P2/n$
**Temperature (K)**	173	220
***a*, *b*, *c* (Å)**	24.2749(6), 8.4159(2), 11.8704(3)	10.6271(8), 11.5782(8), 28.703(2)
**α, β, γ (°)**	90, 90.585(3), 90	90, 90.715(7), 90
***V* (Å^3)**	2424.92(11)	3531.5(5)
Z	4	4
***F*(000)**	944	1229
**D_x (Mg m^{-3})**	1.218	1.084
**Radiation type**	Mo *K*α	Mo *K*α
**μ (mm^{-1})**	0.16	0.24
**Crystal shape**	Plate	Elongated plate
**Color**	Colorless	Red
**Crystal size (mm)**	0.29 × 0.16 × 0.08	0.30 × 0.05 × 0.03
**T_{min}, T_{max}**	0.861, 1.000	0.541, 1.000
**No. of measured, independent and observed [$I > 2\sigma(I)$] reflections**	19726, 5332, 4434	15640, 6630, 3111
**R_{int}**	0.058	0.112
**θ values (°)**	θ_{max} = 27.1, θ_{min} = 2.6	θ_{max} = 25.7, θ_{min} = 2.3
**Range of *h*, *k*, *l***	$h = -31 \rightarrow 31$, $k = -10 \rightarrow 10$, $l = -15 \rightarrow 15$	$h = -11 \rightarrow 12$, $k = -14 \rightarrow 12$, $l = -34 \rightarrow 35$
**$R[F^2 > 2\sigma(F^2)]$, $wR(F^2)$, S**	0.043, 0.115, 1.05	0.079, 0.239, 0.98
**No. of reflections**	5332	6630
**No. of parameters**	301	445
**No. of restraints**	0	78
**H-atom treatment**	H atoms treated by a mixture of independent and constrained refinement	H atoms treated by a mixture of independent and constrained refinement
**$\Delta\rho_{max}$, $\Delta\rho_{min}$ (e Å^{-3})**	0.44, –0.20	0.43, –0.29

Computer programs: *X-AREA* (Stoe & Cie, 2001), *CrysAlis*^{Pro} 1.171.42.43a (Rigaku OD, 2022), *SHELXS* (G. M. Sheldrick, 1997), *SHELXT* (G. M. Sheldrick, 2015), *SHELXL-2018*/3 (Sheldrick, 2018).

3.1. Crystal structure of 1^{2H}

Molecular compound $\mathbf{1^{2H}}$ crystallizes in a solvent-free form in the triclinic crystal system, with one crystallographically unique molecule occupying an inversion center of the space group $P\bar{1}$ (No. 2, Figure S103). The H atoms belonging to the SiH_2 groups were located on the difference electron-density map and refined without constraints. The DSA fragment is planar as imposed by symmetry. The C–Si–C bond angle amounts to 112.58(7)°. Further geometric parameters can be found in the comparative Table S10.

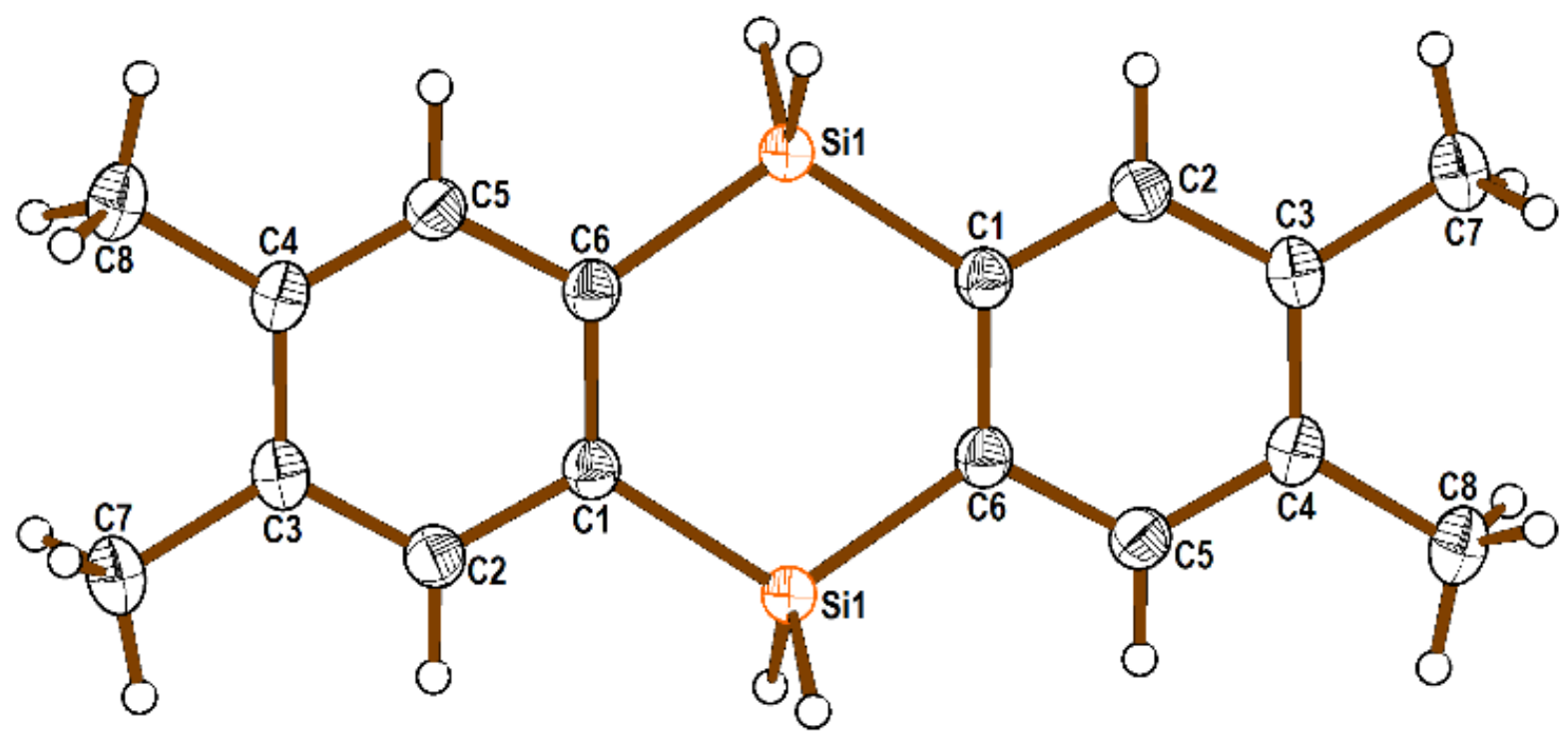

Figure S103. Molecular structure of $\mathbf{1^{2H}}$ with atomic displacement ellipsoids drawn at the 50% probability level.

3.2. Crystal structure of **VI**

Molecular compound **VI** crystallizes in a solvent-free form in the monoclinic crystal system, polar space group *Cc* (No. 9), with one crystallographically unique molecule in the general position (Figure S104). The H atoms belonging to the SiH, SiH_2, and SiH_3 groups were located on the difference electron-density map and refined without constraints.

The DSA fragment is planar within experimental uncertainty. The C–Si–C bond angles comprise 112.6(2)° for Si1 and 111.4(2)° for Si2. The aryl substituent at Si2 is rotated relative to the Si1···Si2 vector by 11.6(4)°.

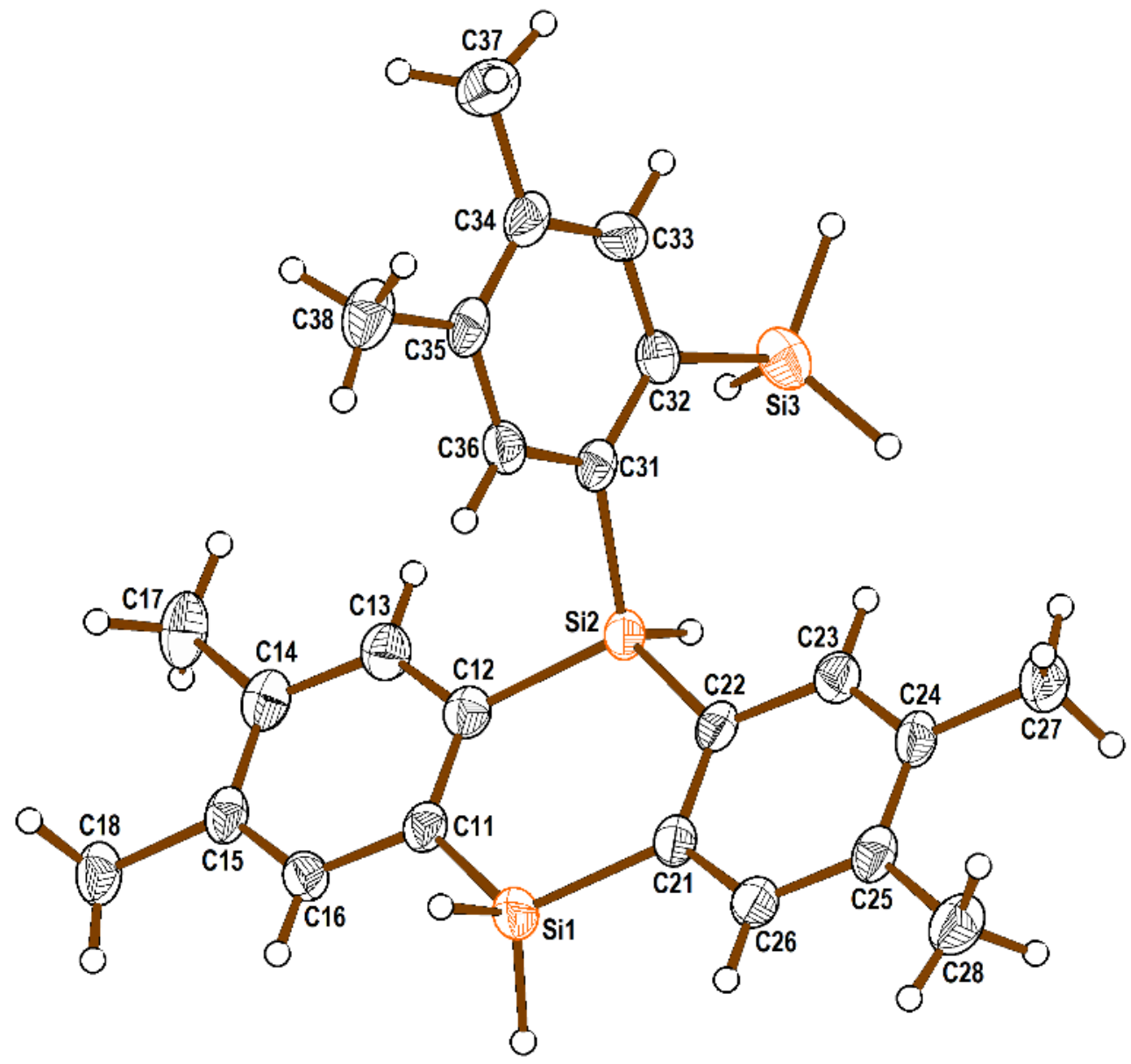

Figure S104. Molecular structure of **VI** with atomic displacement ellipsoids drawn at the 50% probability level.

3.3. Crystal structure of **VII**

Molecular compound **VII** crystallizes in a solvent-free form in the monoclinic crystal system with one crystallographically unique molecule located on a 2-fold axis in the space group *C*2/*c* (No. 15, Figure S105). The H atoms belonging to the SiH groups were located on the difference electron-density map and refined without constraints. The C–Si–C bond angles vary in a narrow range of 103.37(4)–104.58(6)°. With its tricyclic structure, the molecule **VII** features one of the shortest Si···Si intramolecular distances of 2.9816(8) Å in the investigated series (Table S10).

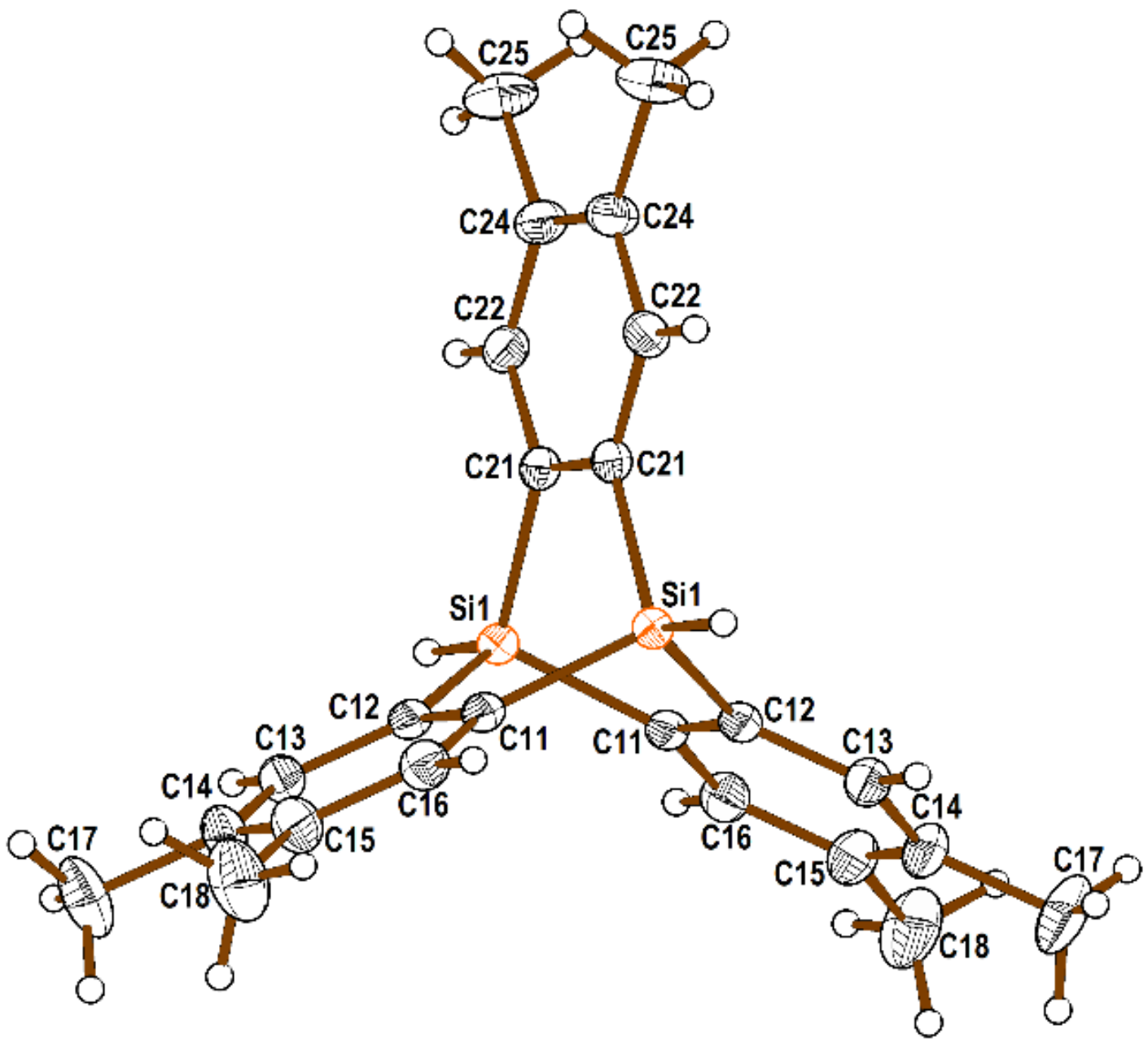

Figure S105. Molecular structure of **VII** with atomic displacement ellipsoids drawn at the 50% probability level.

3.4. Crystal structure of K_2[**1**]

Compound K_2[**1**] crystallizes in the triclinic crystal system with eight crystallographically unique [**1**]$^{2-}$ anions, 16 K^+ cations, and 25 conformationally disordered THF molecules in the general positions of the space group $P\bar{1}$ (No. 2). The H atoms belonging to the SiH groups were located on the difference electron-density map and refined without constraints. The C–Si–C bond angles range from 95.7(2) to 98.21(17)° (av. 97.1[7]°). Eight significantly folded [**1**]$^{2-}$ anions assemble into four pairwise capsules, each encapsulating one K^+ cation that weakly interacts with the aryl fragments of the DSA (K1–K4; Figure S106). The shortest K···C contacts range from 2.964(4) to 3.127(4) Å.

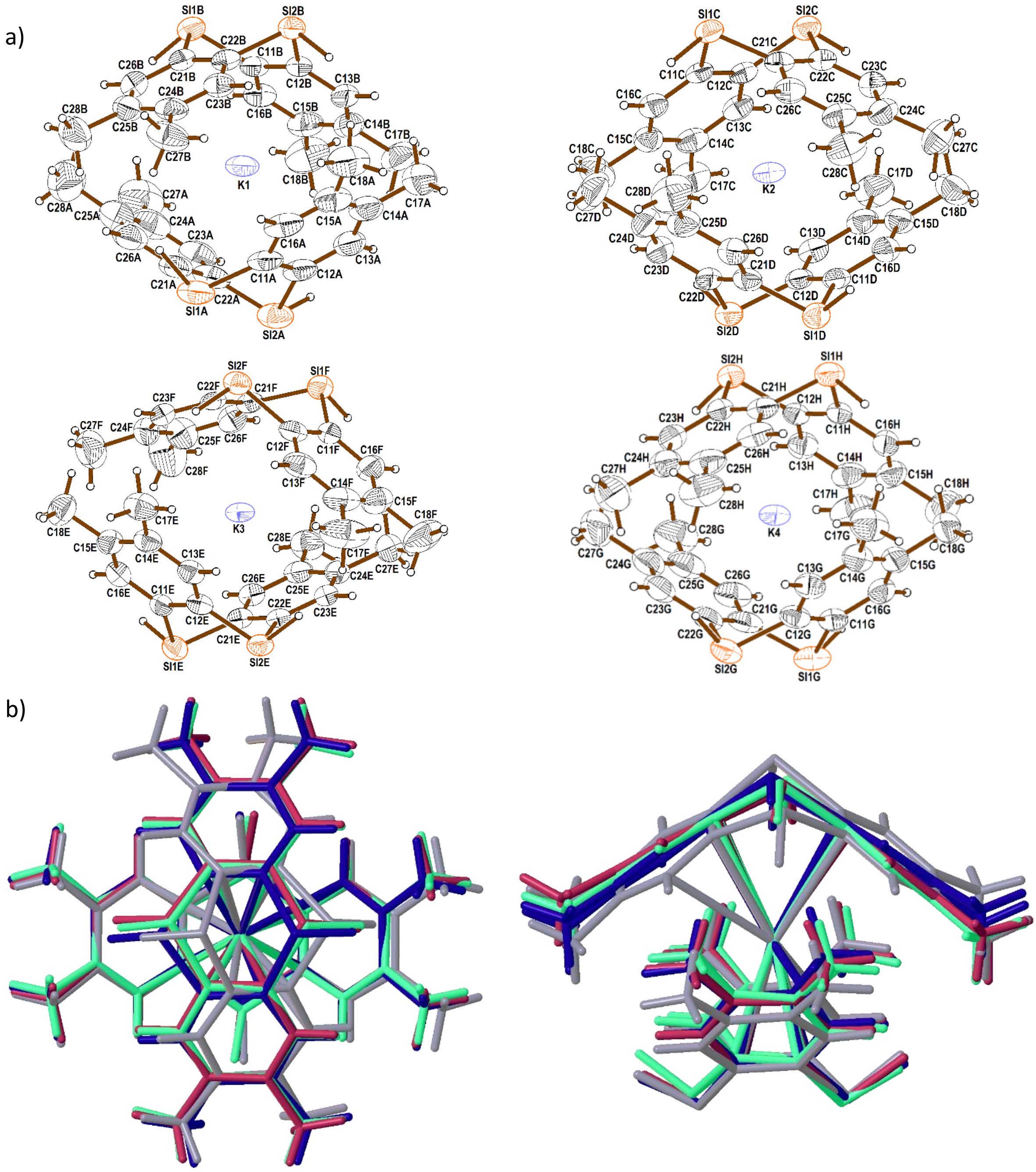

Figure S106. a) Molecular structures of the four crystallographically unique capsules formed by pairs of [**1**]$^{2-}$ anions, each encapsulating one potassium cation with atomic displacement ellipsoids drawn at the 50% probability level. b) Overlay of the four crystallographically unique capsules (K1: red, K2: blue, K3: grey, K4: green). The root-mean-square deviations (RMSD) between the capsules amount to 0.203 Å (K1/K2), 0.033 Å (K1/K3), and 0.210 Å (K1/K4). The vectors defined by the Si···Si directions of the two concave DSA fragments are close to orthogonal, with an average angle of 92[2]°.

The remaining twelve potassium cations are weakly coordinated through K···C interactions to the capsules and additionally interact with THF solvent molecules. These twelve potassium cations are arranged in four triangular units located between adjacent capsules, which link the capsules into extended motifs. In the triangular units K5–K7 and K14–K16, each potassium cation is coordinated by two THF molecules. In the triangle formed by K8–K10, each potassium cation is likewise coordinated by two THF molecules; however, one THF molecule is shared between K8 and K10. Similarly, in the triangle formed by K11–K13, each potassium cation is coordinated by two THF molecules, with an additional THF molecule shared between K11 and K12. As a result, linear chains are formed in the crystal structure along the b axis (Figure S107).

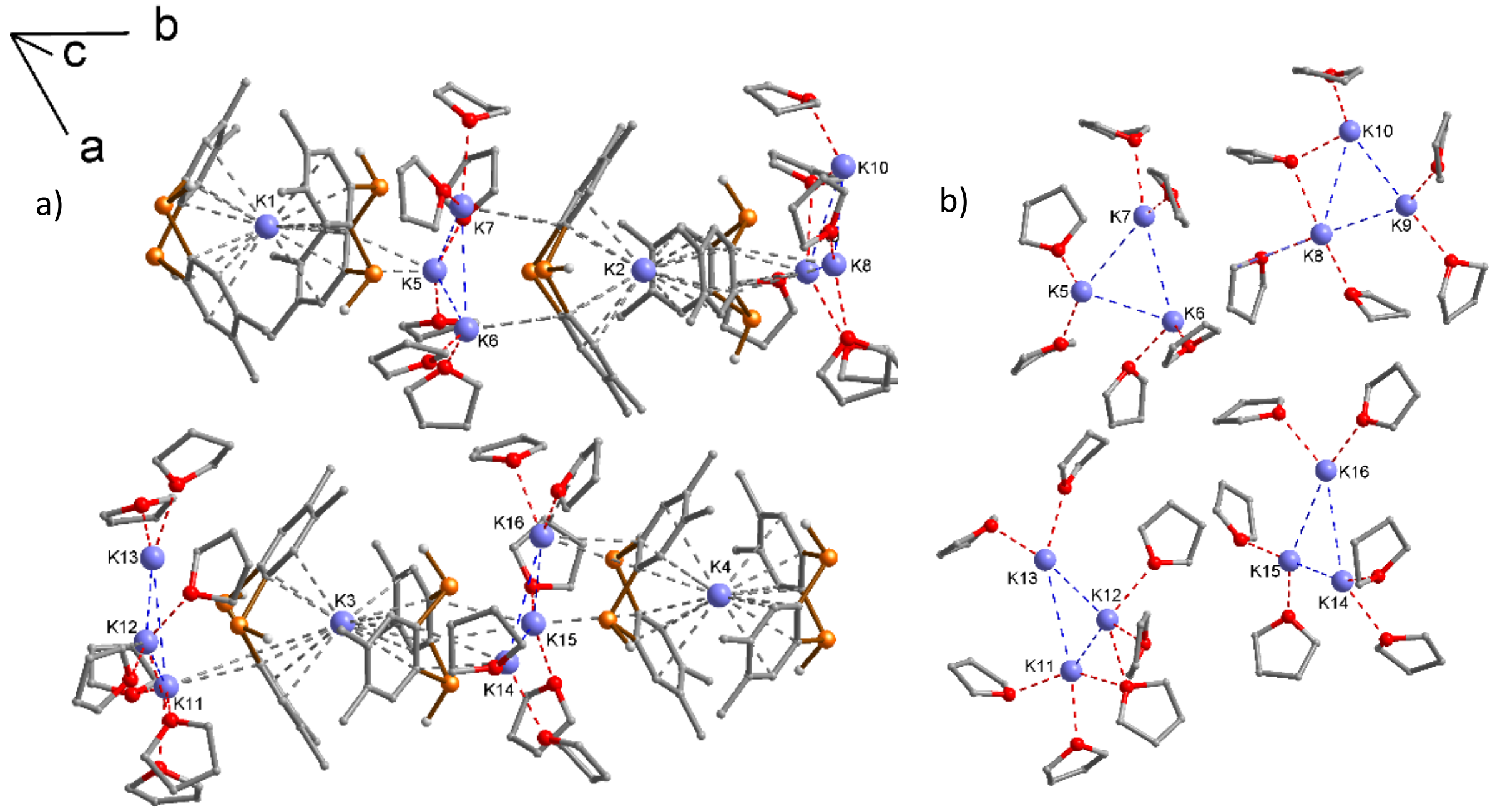

Figure S107. (a) Crystal packing of K_2[**1**] showing the linear chains formed along the b axis by the capsules and the interstitial potassium cations. The potassium cations are arranged in four triangular units located between adjacent capsules, thereby linking the capsules into extended motifs. (b) Isolated representation of the twelve potassium cations and their coordinating THF solvent molecules, illustrating the triangular arrangements and shared THF molecules within selected potassium triangles; the capsule framework is omitted for clarity. Only Si-bound hydrogen atoms are shown. Si: orange; O: red; H: light grey; C: grey.

3.5. Crystal structure of K_2[**10**]

Compound K_2[**10**] crystallizes in the cubic crystal system, space group *Im*$\bar{3}$*m* (No. 229). The structure is severely disordered over a crystallographic position (detailed description below) with the highest possible crystallographic point symmetry Oh (m¯3m). Due to this severe disorder, the atomic displacement parameters are relatively large, which may also explain the weak scattering at high 2θ angles. For the final structure refinement, all reflections beyond the diffraction limit d_{hkl} of 1 Å ($sin\theta/\lambda$ = 0.5 $Å^{-1}$) were discarded. Owing to the disorder, the Si–H bond length in the SiH group was constrained to the literature-known value of 1.37 Å, while all other H atoms were refined in riding mode.

The crystallographically unique part contains only seven non-hydrogen atoms, namely one K, one mixed Si/B position, and five carbon atoms (Figure S108a). The atoms K1, C2, C3, and C4 are fully occupied. The carbon atoms C2, C3, and C4 represent half of an aryl ring, which is bisected by a mirror plane. These atoms belong simultaneously to both DBA and DSA fragments, which are superimposed due to the high crystallographic symmetry. At the same time, the Si1 position is shared between silicon and boron in a 1:1 ratio. In one orientation, these atoms form the B···B pair of the DBA unit, whereas in the alternative orientation, they correspond to the Si···Si unit of the DSA fragment.

Based on the presence of B–CH_3 and Si–H groups, the C1 atom of the terminal methyl group of the DBA fragment is half-occupied and is shared with the H1 atom of the SiH group. In addition to atoms C2–C4 of the aryl ring, the DSA fragment includes the half-occupied atom C5 and three hydrogen atoms. The methyl group defined by these four atoms overlaps with the terminal H4A atom (50%) of the DBA fragment (Figure S108b).

The K^+ cation is weakly coordinated to four aryl rings (K···C = 3.275(4)–3.373(5) Å), thereby linking the organic anions into a three-dimensional network.

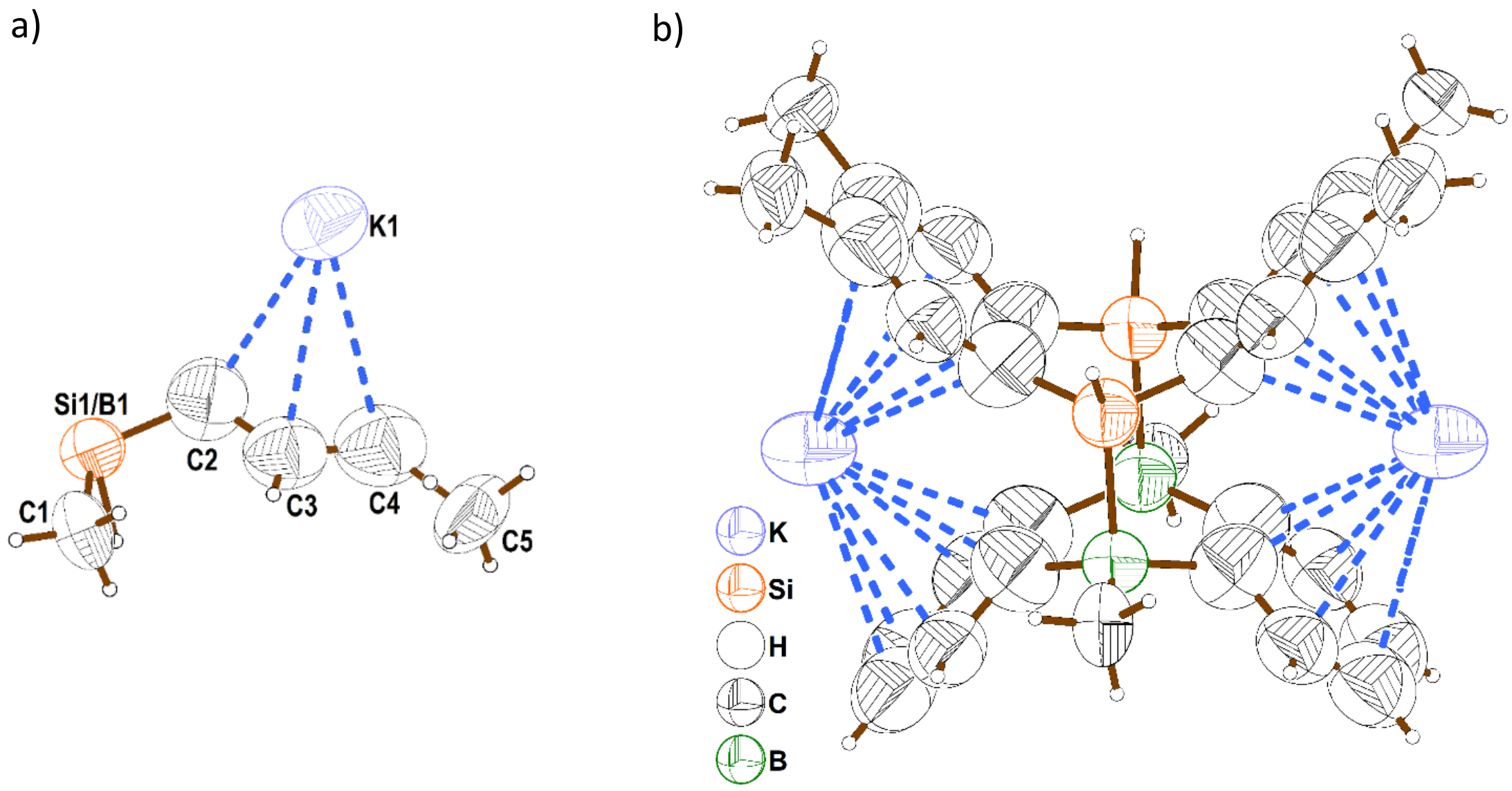

Figure S108. Structure of K_2[**10**] with atomic displacement ellipsoids drawn at the 50% probability level: (a) crystallographically unique part; (b) the anion coordinated to the K^+ cations.

3.6. Crystal structure of [K(18-c-6)(THF)$_2$][K(18-c-6)]$_3$[**10**]$_2$

The formation of the (18-c-6) adduct of K_2[**10**] was intended to avoid crystallization of the cubic phase, where the dianion is disordered by symmetry. This crystallization attempt afforded small orange plates of the ionic compound [K(18-c-6)(THF)$_2$][K(18-c-6)]$_3$[**10**]$_2$ in the triclinic crystal system, space group $P\bar{1}$ (No. 2), as a THF/pentane solvate. Unfortunately, the diffraction experiment could only be carried out at a relatively high temperature of 220 K due to a malfunction of the low-temperature device. Together with the small size of the single crystal, the dataset obtained does not meet the highest standards of modern SCXRD because of the relatively low diffraction limit of 0.95 Å and the presence of numerous disordered structural fragments arising from thermal motion. However, the data quality is sufficient to establish the structure of the dianion and to model most of the disordered regions, including the solvent molecules. The geometric parameters of the anionic structural fragments are sufficiently reliable. However, the solvent content may be significantly underestimated, as at each site the sum of the occupancy factors of the overlapping solvent molecules remains well below 100%. The localized solvent molecules were refined isotropically using multiple constraints on interatomic distances. Furthermore, the residual electron density in these regions is rather flat, and additional solvent molecule positions cannot be refined.

The asymmetric unit contains four crystallographically unique K cations and two crystallographically independent [**10**]$^{2-}$ dianions in general positions (Figure S109). The C–Si–C bond angles in the ordered DSA fragment (including atoms Si11 and Si12) are 103.3(3) and 103.6(3)°. The disorder pattern for the DSA fragment containing Si11 and Si12 atoms does not allow accurate determination of the bond angles, as two positions of the aryl rings (ratio 0.53:0.47) are very close to each other.

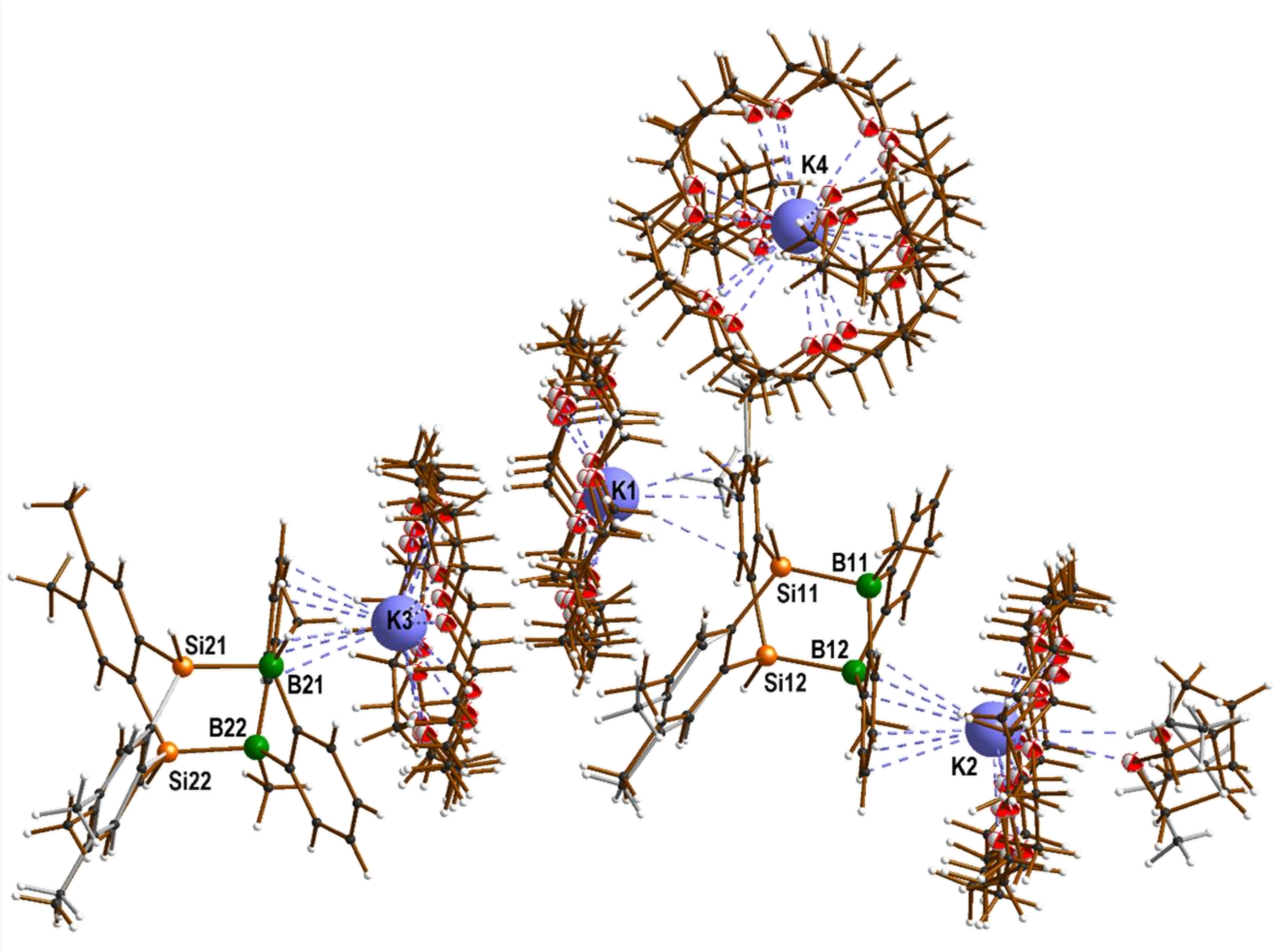

Figure S109. Asymmetric unit of [K(18-c-6)(THF)$_2$][K(18-c-6)]$_3$[**10**]$_2$ represented in a ball-and-stick style. Solvated THF and pentane molecules are not shown. The disordered fragments of the anions are shown in light grey. O: red; H: light grey; C: black.

Both dianions form contact ion pairs (Figure S110); one dianion (containing atoms Si11 and Si12) is weakly coordinated by [K(18-c-6)(THF)]$^+$ to the aryl fragment of the DBA unit (K2···C = 3.180(7)–3.353(6) Å) and by [K(18-c-6)]$^+$ to the DSA unit, with K1···C distances in the range 3.283(7)–3.471(6) Å. The other dianion, containing atoms Si21 and Si22, interacts with only one [K(18-c-6)]$^+$ cation. The K3···C distances are 3.130(6)–3.392(5) Å. For both dianions, the positions of the hydrogen atoms were refined in riding positions on the pivot Si atoms (Si–H = 1.37 Å), as free refinement gave unreliable results. The isotropic atomic displacement parameter (ADP) was set to 120% of the equivalent ADP of the pivot Si atom.

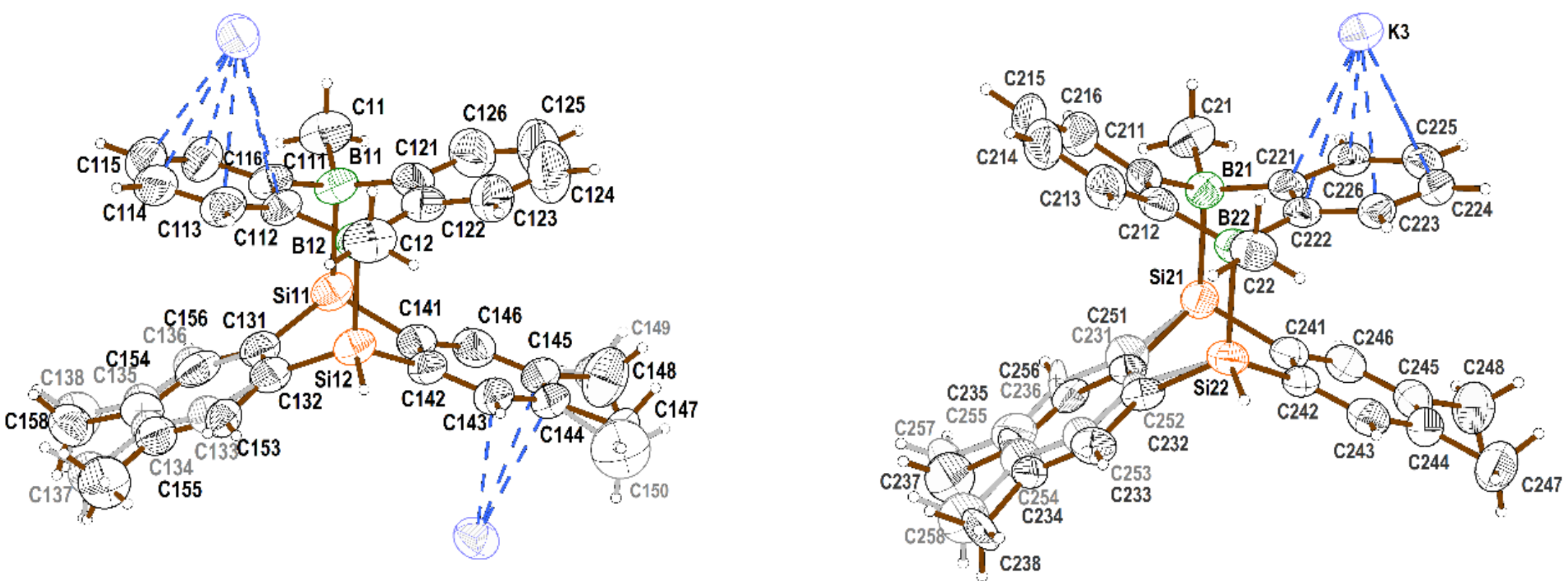

Figure S110. Structures of the contact ion pairs, [K(18-c-6)(THF)][K(18-c-6)][**10**] (left) and [K(18-c-6)][K(18-c-6)][**10**] (right) with atomic displacement ellipsoids drawn at the 50% probability level. The disordered fragments of the anions are shown in light grey. Coordinated 18-c-6 and THF molecules are not shown for clarity.

The fourth cation (K4), [K(18-c-6)(THF)$_2$]$^+$, is located in the outer coordination sphere (Figure S111). All four K-coordinated 18-crown-6 molecules are disordered over three positions each and refined using constraints and restraints on bond lengths (DFIX, SADI) and 1,3 distances (DANG). The THF and pentane solvent molecules are disordered over multiple positions and refined with restrained geometry.

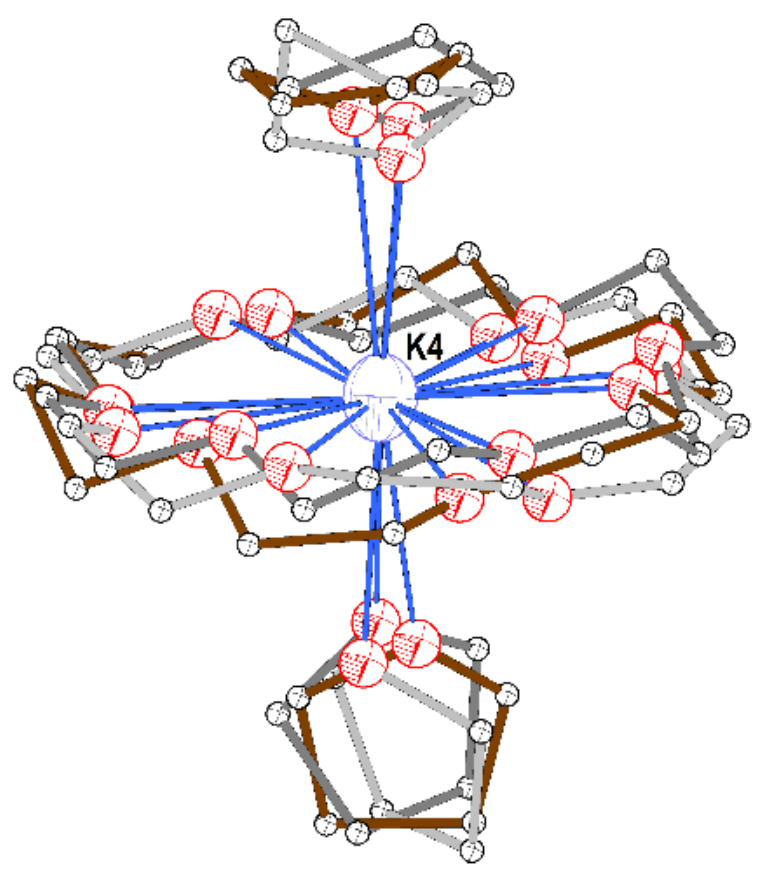

Figure S111. Structure of [K(18-c-6)(THF)$_2$]$^+$ in a ball-and-stick style. The disordered positions of 18-c-6 and THF molecules are shown in different colors.

3.7. Crystal structure of *cis*-**2**

Molecular compound *cis*-**2** crystallizes in a solvent-free form in the monoclinic crystal system, space group *C*2/*c* (No. 15), with three crystallographically unique molecules. Two of them (denoted as molecules A and B) lie in general positions, while the third (molecule C) lies on the 2-fold axis, resulting in 20 molecules (8A + 8B + 4C) per unit cell (Figure S112). The H atoms of the SiH groups were located on the difference electron-density map and refined without constraints. The overlay of the three independent molecules shows that all three molecules are geometrically similar. Moreover, the $SiMe_3$ substituents of molecules A and B nearly coincide. The conformation of molecule C on the 2-fold axis differs in the orientation of one of the $SiMe_3$ groups, which is rotated by up to 45° relative to the corresponding substituent in molecule B. The C–Si–C bond angles in DSA fragments range from 110.82(9) to 111.31(9)°.

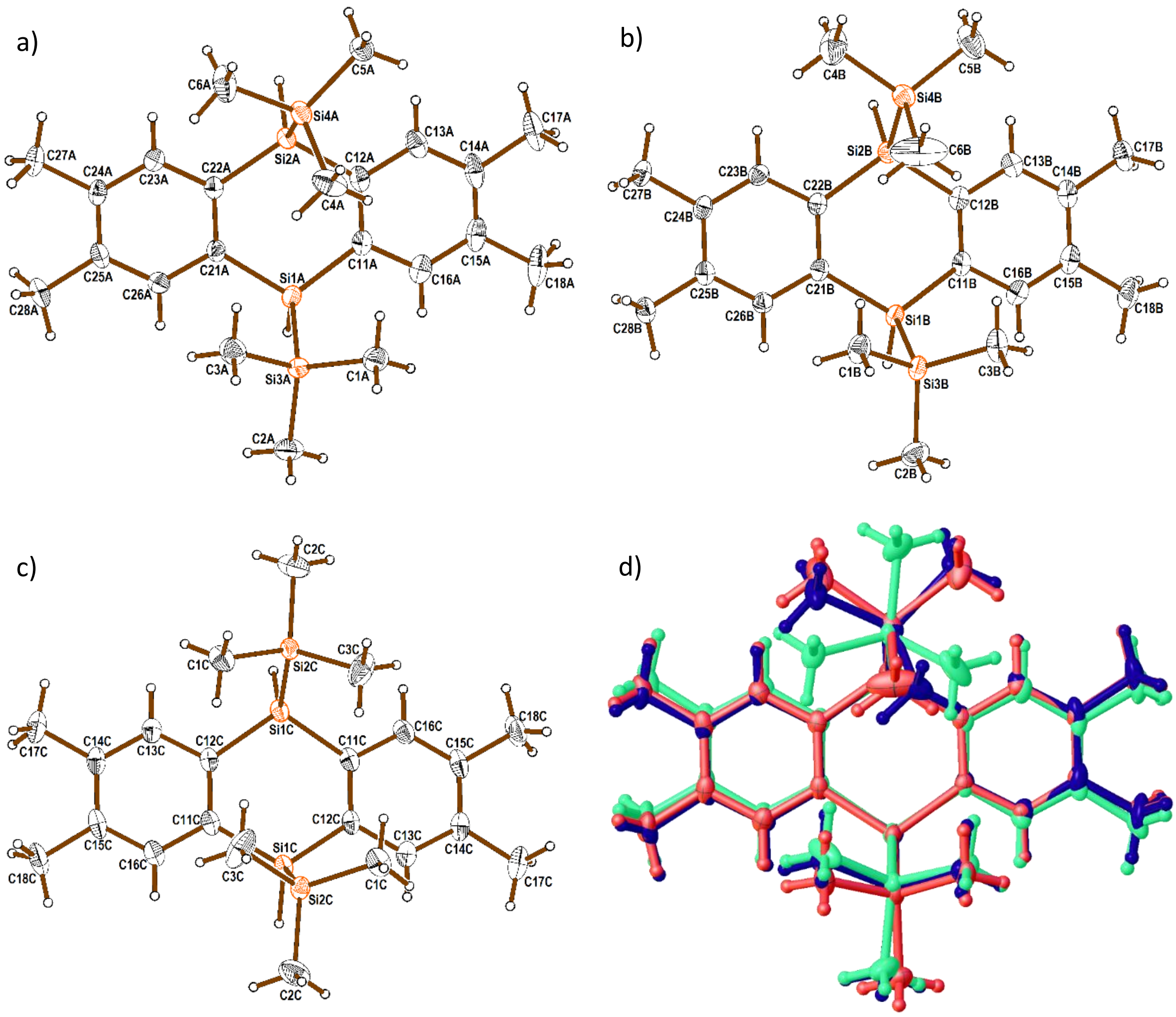

Figure S112. Molecular structure of *cis*-**2**. (a–c) Three crystallographically unique molecules with atomic displacement ellipsoids drawn at the 50% probability level. Atoms belonging to the independent molecules A, B, and C are labeled with the corresponding suffixes. Atoms of molecule C are generated by the symmetry operation (1−x, y, ½−z); (d) Overlay of the crystallographically unique molecules (A: blue, B: red, C: green).

3.8. Crystal structure of *cis*-**3**

Molecular compound *cis*-**3** crystallizes in the monoclinic crystal system with one crystallographically unique molecule in the general position of the space group $P2_1/c$ (No. 14). One of the Si(H)*t*Bu_2 fragments is conformationally disordered with the SiH group split into two positions with a 97:3 occupancy ratio (Figure S113, dashed lines). The H atoms of the SiH groups (including the major position for the disordered one) were located on the difference electron-density map and refined without constraints. For the minor position (at Si3B), the H atom was refined as riding on the pivot silicon atom. The C–Si–C bond angles in DSA fragments are 111.65(6) and 111.94(6)° for Si1 and Si2, respectively. The torsion angle Si3–Si1···Si2–Si4 of 9.67(4)° shows a slightly twisted arrangement of the substituents relative to the transannular Si···Si vector. The substituents at the DSA Si atoms are rotated by 10.53(4)° and 35.90(6)°, respectively, as indicated by the respective C31–Si3–Si1···Si2 and Si1···Si2–Si4–C41 torsion angles, to avoid unfavorable close contacts.

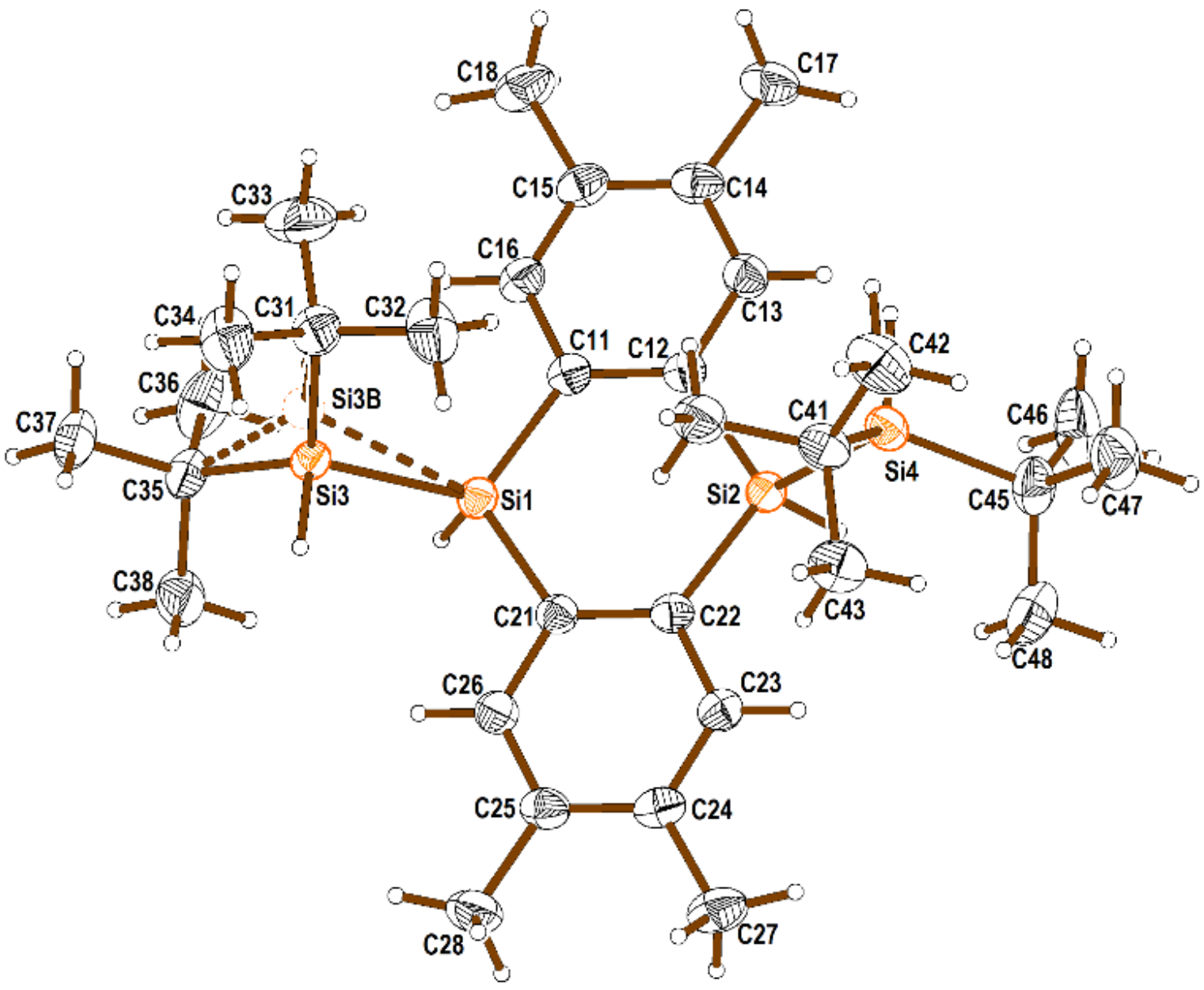

Figure S113. Molecular structure of *cis*-**3** with atomic displacement ellipsoids drawn at the 50% probability level. Dashed lines show the minor (3%) position of the SiH fragment.

3.9. Crystal structure of **4**

Molecular compound **4** crystallizes in a solvent-free form in the triclinic crystal system, space group $P\bar{1}$ (No. 2), with one crystallographically unique molecule in the general position (Figure S114). The H atoms of the SiH groups were located on the difference electron-density map and refined without constraints. The Si–Si bond lengths in the tricyclic fragment are 2.3645(9) and 2.3956(10) Å for the bonds to the bridgehead atoms Si1 and Si4, and 2.3636(10) Å for the bridging unit (Si2–Si3). The C–Si–C bond angles in the DSA fragment are 107.74(11) and 107.12(11)° for Si1 and Si4, respectively. The C–Si–C bond angles within the bridging fragment are almost equal (110.61(13) and 110.61(15)° for C1–Si2–C2 and C3–Si3–C4, respectively) and are closer to the tetrahedral angle.

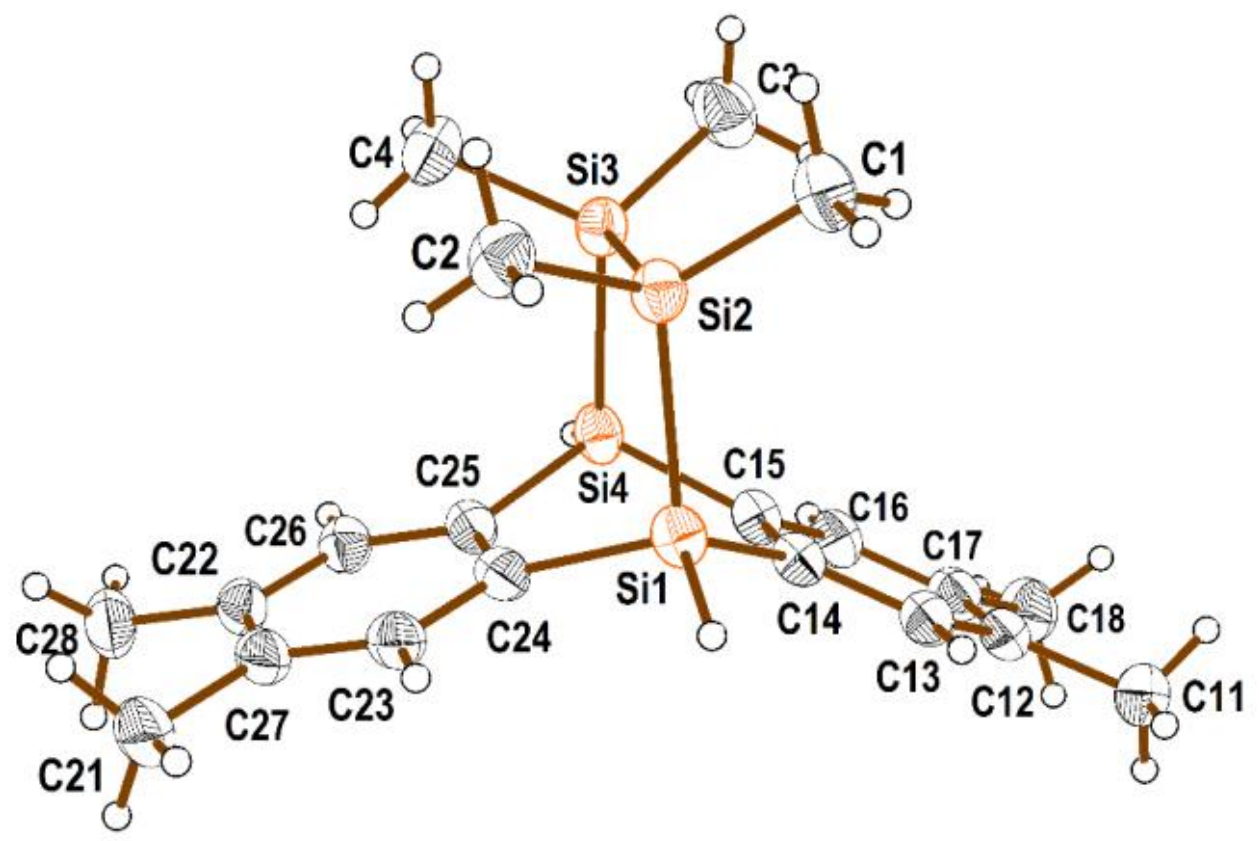

Figure S114. Molecular structure of **4** with atomic displacement ellipsoids drawn at the 50% probability level.

3.10. Crystal structure of **5**

Molecular compound **5** crystallizes in a solvent-free form in the monoclinic crystal system, space group $P2_1/c$ (No. 14), with one crystallographically unique molecule in the general position (Figure S115). The H atoms of the SiH groups were located on the difference electron-density map and refined without constraints. The C–Si–C bond angles in the DSA fragment are 111.08(13) and 111.58(13)° for Si3 and Si2, respectively, whereas the Si–Si bond lengths are 2.3411(12) Å (Si1–Si2) and 2.3523(10) Å (Si1–Si3). For the bridging $SiMe_2$ fragment, the C–Si1–C bond angle is more acute and measures 107.70(13)°. The Si2–Si1–Si3 bond angle is 107.70(4)°.

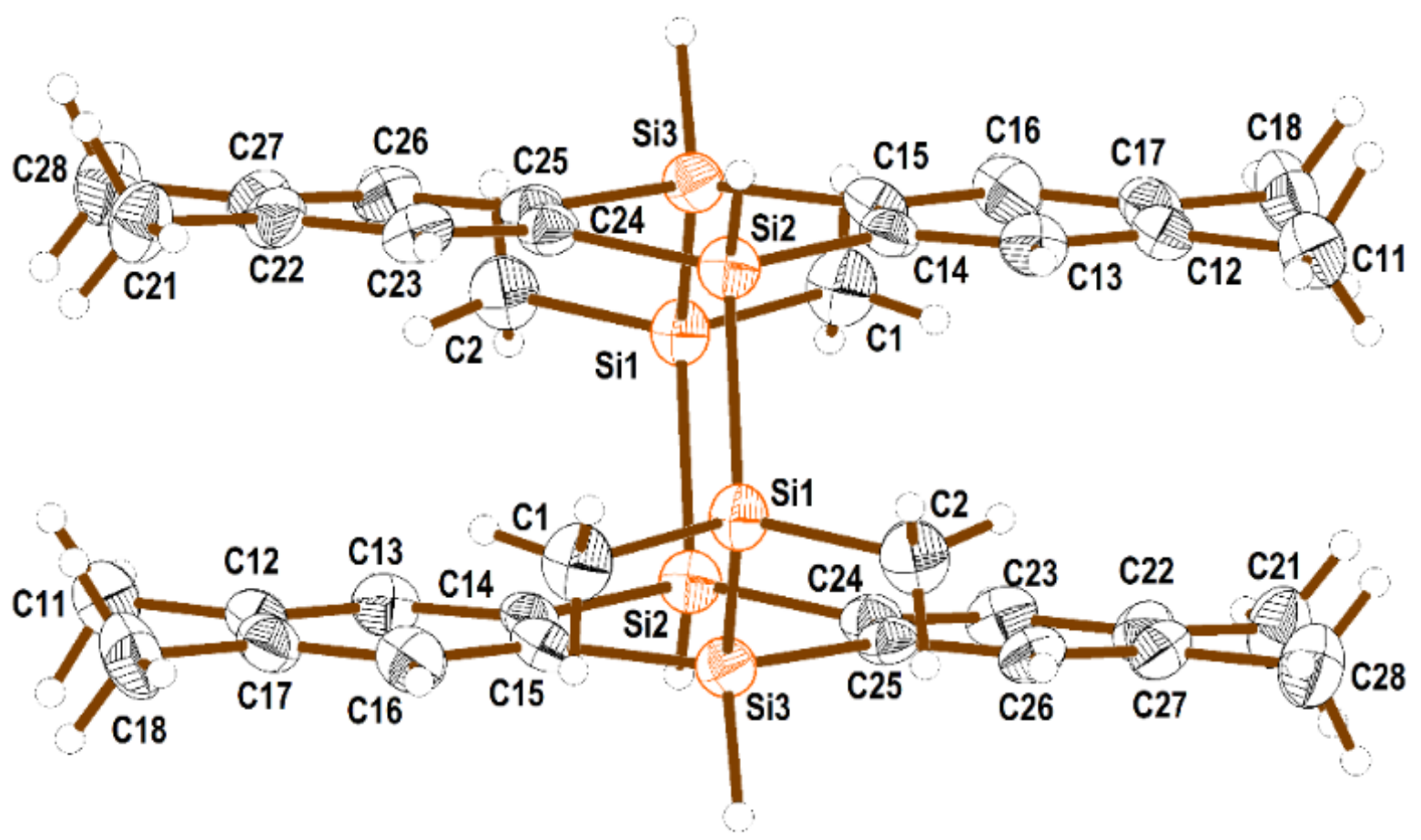

Figure S115. Molecular structure of **5** with atomic displacement ellipsoids drawn at the 50% probability level.

3.11. Crystal structure of **6**

Molecular compound **6** crystallizes in the orthorhombic crystal system with one crystallographically unique molecule in the general position of the chiral space group $P2_12_12_1$ (No. 19, Figure S116). The H atoms of the SiH and SiH_2 groups were located on the difference electron-density map and refined without constraints. The C–Si–C bond angles in the folded DSA fragment comprise 110.93(9) and 109.45(9)° for Si1 and Si2, respectively. The Si2–Si3 bond to the $SiMe_3$ group is 2.3432(7) Å. The torsion angle Si1···Si2–Si3–C3 of 2.20(17)° indicates the absence of significant steric hindrance for the $SiMe_3$ group.

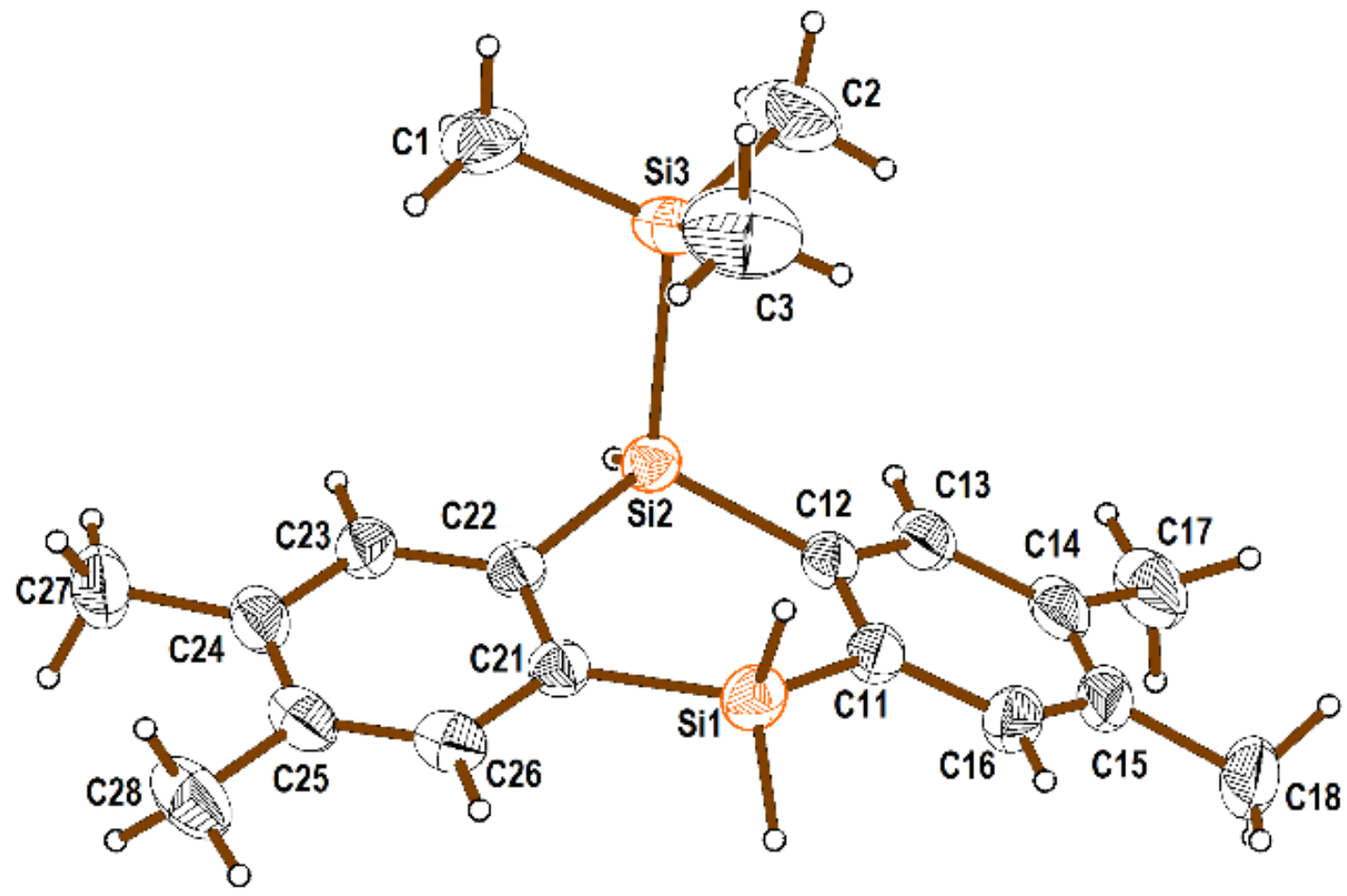

Figure S116. Molecular structure of **6** with atomic displacement ellipsoids drawn at the 50% probability level.

3.12. Crystal structure of **7**

Molecular compound **7** crystallizes in the triclinic crystal system, space group $P\bar{1}$ (No. 2) with one crystallographically unique molecule located on an inversion center (Figure S117). The H atoms of the SiH and SiH_2 groups were located on the difference electron-density map and refined without constraints. Analysis of the difference electron density near the Si2 atom revealed the presence of a methyl group, with the occupancy factor refined to 30%. The alternative position at Si2 is occupied by the terminal atom H2 (by 70%). Therefore, the crystal does not correspond to a single compound but to a solid solution of two or three distinct chemical species, each bearing either an H or a Me group at the two symmetry-related Si2 positions (related by crystallographic inversion). Due to crystallographic symmetry C_i, the following disorder models are equally consistent with the SCXRD data, and neither can be preferred on crystallographic grounds alone:

1) 70% *(H,H)* + 30% *(Me,Me)*

2) 40% *(H,H)* + 30% *(H,Me)* + 30% *(Me,H)*

Here, *(H,H)* denotes a molecule bearing SiH groups at both Si2 sites related by inversion symmetry, whereas *(H,Me)* and *(Me,H)* refer to mixed substitution patterns.

This substitutional disorder is likely due to partial methylation occurring during the synthesis of $\mathbf{1}^{\mathbf{2H}}$ from **DSB** using MeLi, where residual methylating agent may lead to minor formation of methyl-substituted species. Such species were only observed in very small amounts during the synthesis of $\mathbf{1}^{\mathbf{2H}}$ and are largely removed during the work-up (e.g., washing with *n*-pentane), although trace amounts may persist. Subsequent reactions carried out with this material may retain these minor impurities. It should be noted, however, that NMR spectroscopic data do not indicate the presence of such methylated species in the bulk material, suggesting that the observed disorder arises from a minor component selectively incorporated into the analyzed single crystal.

The Si–C bond lengths within the DSA fragment range from 1.852(4) to 1.878(4) Å, whereas the C–Si–C bond angles are 111.5(2)° for Si2 and 112.3(2)° for Si3. The Si–Si bond lengths are 2.3548(18) Å for the bridgehead Si2 atom (Si1–Si2) and 2.348(2) Å for the central bond Si1–Si1. The torsion angle Si3···Si2–Si1–Si1 between the Si3···Si2 axis of the DSA fragment and the central Si1–Si1 bond amounts to 2.48(9)°, which is similar to the value observed for the corresponding torsion angle in **6**. The C–Si–C bond angles at the bridging Si1 (111.0(2)°) are closer to the tetrahedral angle than the corresponding angles within the DBA fragment.

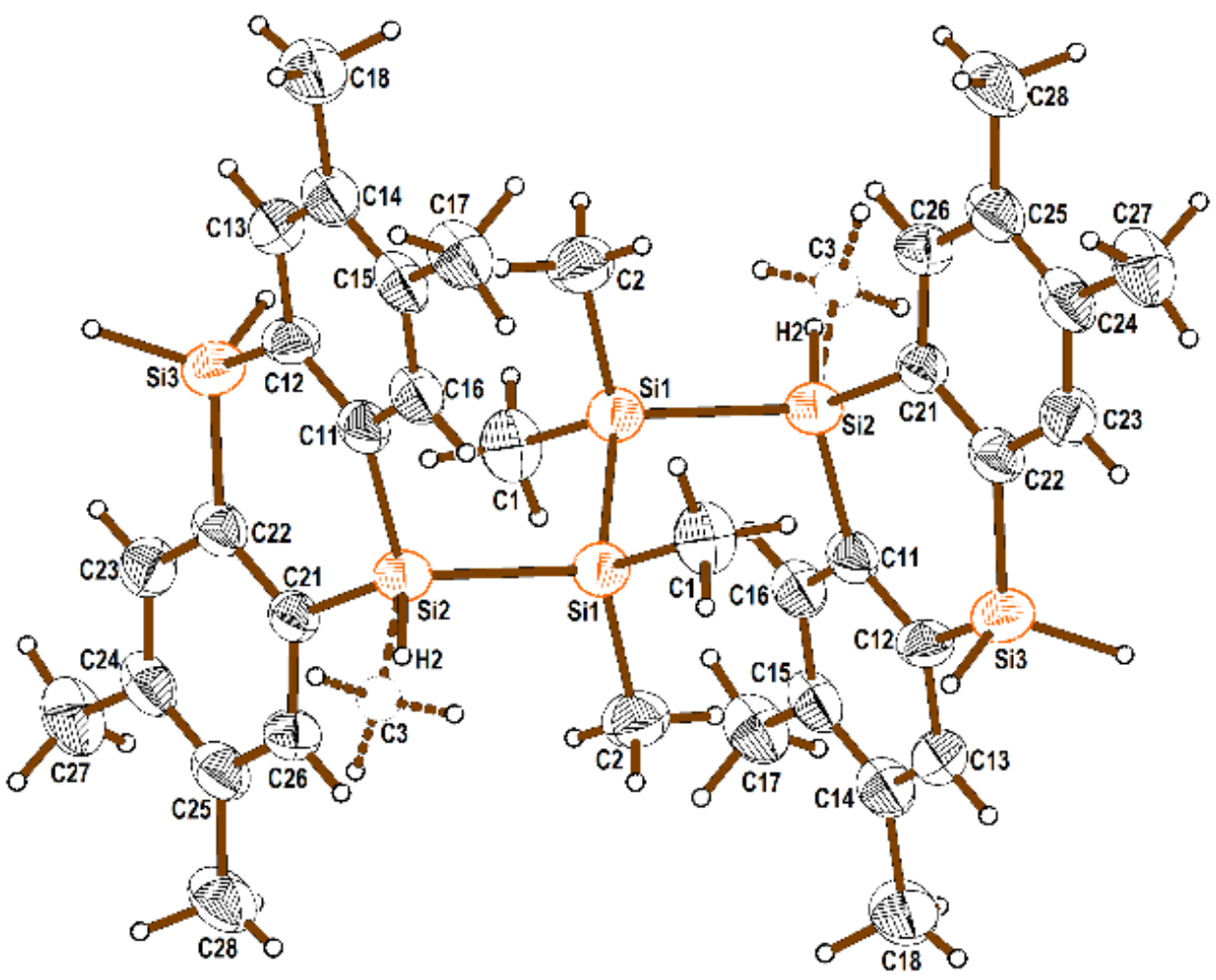

Figure S117. Molecular structure of **7** with atomic displacement ellipsoids drawn at the 50% probability level. The position of the partly occupied CH_3 group at atom Si2 is depicted with dashed lines.

3.13. Crystal structure of **13**

Molecular compound **13** crystallizes in the monoclinic crystal system, space group $P2_1/c$ (No. 14), with one crystallographically unique molecule in the general position (Figure S118). The H atoms of the SiH groups were located on the difference electron-density map and refined without constraints. Both DSA and DHA fragments are significantly folded. The Si–C bond lengths within the DSA fragment range from 1.8676(17) to 1.8749(17) Å, whereas the bridging Si1–C1 bond between DSA and DHA is longer and is 1.9416(16) Å. The bond angles at the Si center are close to the tetrahedral angle: they are 107.41(7)° and 107.63(7)° within the DSA fragment and range from 107.14(7) to 109.21(7)° for angles involving the C1 and C2 bridgehead atoms.

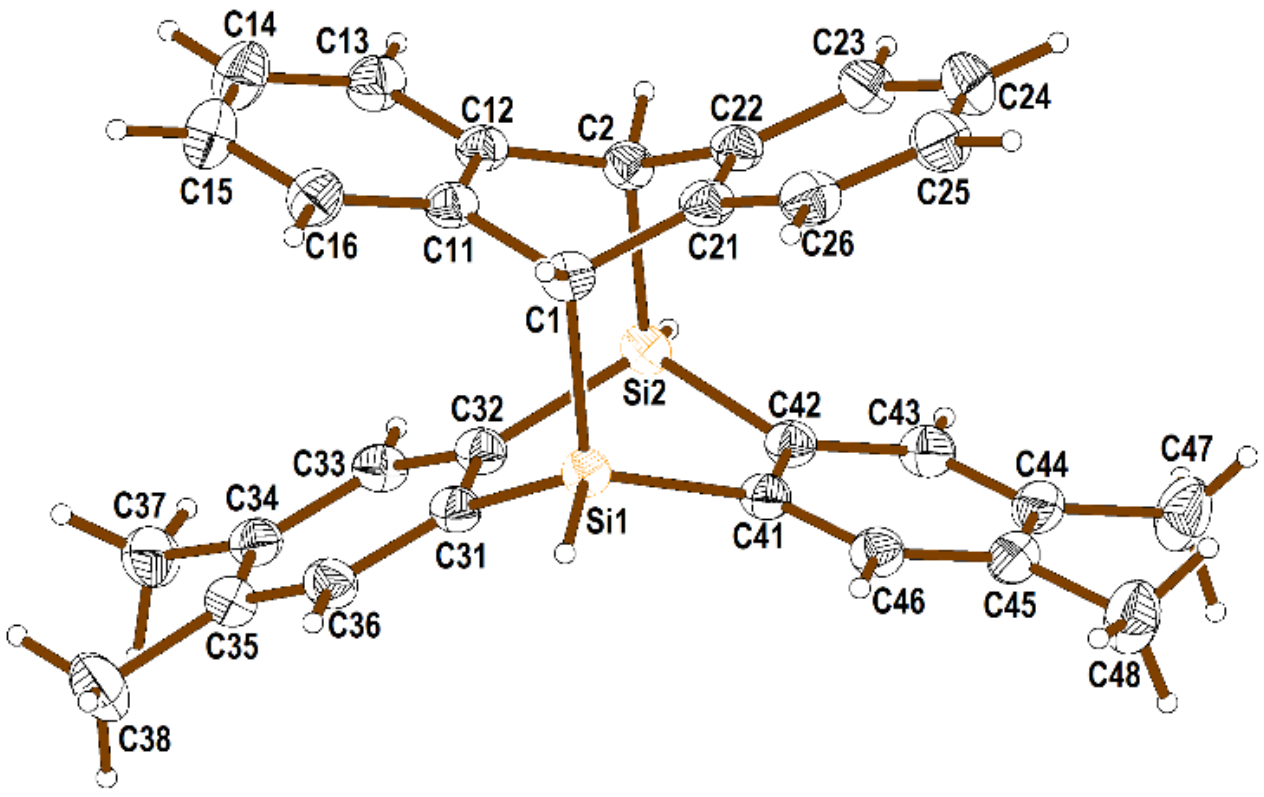

Figure S118. Molecular structure of **13** with atomic displacement ellipsoids drawn at the 50% probability level.

3.14. Crystal structure of K[**15**]

Compound K[**15**] crystallizes in the monoclinic crystal system, space group $P2/n$ (No. 13), as a THF/Et_2O solvate, with two crystallographically unique K^+ positions and a carbanion [**15**]$^-$ (Figure S119a) in the asymmetric unit. The carbanion occupies the general position. The position of the hydrogen atom at C2 is refined without geometrical constraints with the isotropic atomic displacement parameter (ADP) set to 120% of the equivalent ADP of atom C2. As C2 is a chiral center, and the compound crystallizes in the centrosymmetric space group, the anion exists as a racemic mixture of enantiomers.

The DSA fragment of [**15**]$^-$ is folded. The *p*Tol–C–C–*p*Tol bridge results in the shortest intramolecular Si1···Si2 contact of 2.971(2) Å in the investigated series, similar to compound **VII** with a comparable tricyclo[2.2.2] configuration. Due to the slightly longer C1–C2 single bond of 1.556(7) Å (compared to conjugated C11–C12 and C21–C22 bonds of 1.431(7) Å and 1.432(7) Å, respectively), the C–Si–C bond angles are slightly distorted. The C–Si–C bond angles within the DSA fragment are smaller (100.9(2) and 103.4(3)°) than the C–Si–C angles involving the bridgehead C1 and C2 atoms. These angles vary from 103.9(2) to 110.0(2)°, with the most distorted angles involving a negatively charged C2 atom. However, the largest influence on the angular distortion of the pyramidal Si atoms likely arises from the coordination of the potassium cations. The K1 cation lies on a 2-fold axis; the second cation (K2) is disordered about this position, together with the coordinated THF molecule. The cation K1 is weakly coordinated to the anionic carbon center C1, as well as to one of the aryl rings with K···C distances from 3.037(5) to 3.144(5) Å, thereby forming the smallest C–Si–C bond angle within the anionic fragment between C11, Si1 and C1 atoms. The disordered cation K2 is, in turn, coordinated to the aryl rings (K···C distances of 2.938(6)–3.163(6) Å) and one of the THF molecules with K–O of 2.583(6) Å and does not significantly influence the coordination spheres of the Si atoms.

In addition, THF and Et_2O solvent molecules in the interstitial space are severely disordered and overlap in the same position. The site occupancy factors of the three localized THF molecules are 0.125, 0.075, and 0.075; the Et_2O molecule has an occupancy of 0.100 (Figure S119b). The solvent molecules were refined isotropically and with multiple constraints on interatomic distances.

a)

b)

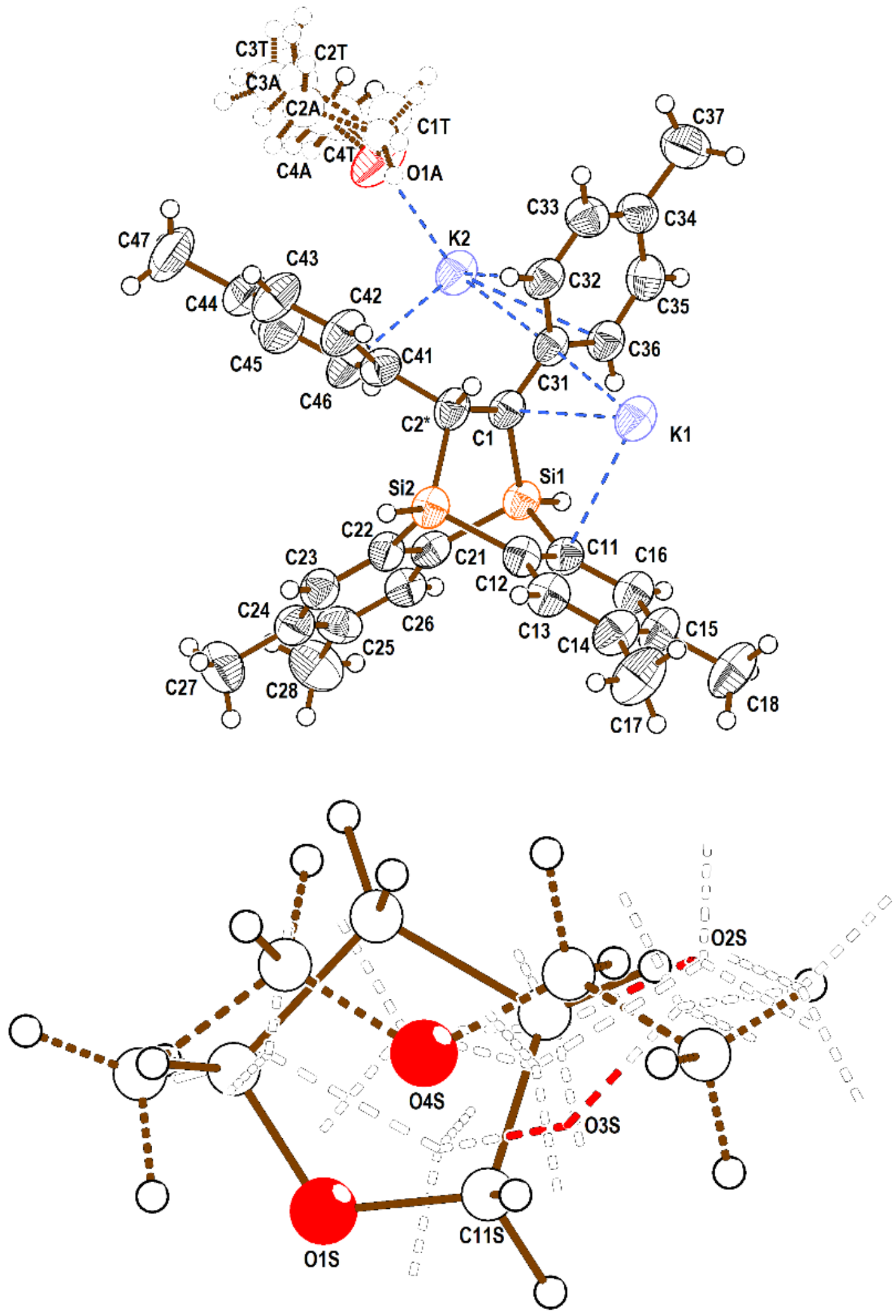

Figure S119. (a) Structure of the anion and its nearest K^+ cations in K[**15**] with atomic displacement ellipsoids drawn at the 50% probability level. The disordered part of the coordinated THF molecule is depicted with dashed lines; (b) Schematic representation of the disorder of the solvent molecules, showing the major occupied positions of the THF (12.5%) and Et_2O (10%) molecules represented in a ball-and-stick style.

3.15. Selected geometrical characteristics of the DSA fragment

Table S10. Selected geometrical characteristics of the DSA fragment. Average values are given with standard deviations in square brackets; where more than one value is listed, the values correspond to the minimum and maximum observed values. Entries without average values correspond to one or two individual values.

Compound[a]	ϕ [°][b]	χ [°][c]	Si–C [Å][d]	Si···Si [Å][e]	Δ(Si···Ar) [Å][f]	$\mathrm{Si_{DSA}}$[g]
**1**^{2H}	180	112.6(1)	1.864(2)<br>1.867(2)	3.481(1)	0.007(2)<br>0.015(2)	$\mathrm{C_2}$**Si**$\mathrm{H_2}$
[**1**]$^{2-}$	107.0(1)<br>115.5(1)<br>av. 111[3]	95.7(2)<br>98.2(2)<br>av. 97.1[8]	1.903(6)<br>1.930(5)<br>av. 1.916[7]	3.203(2)<br>3.262(2)<br>av. 3.234[19]	0.012(6)<br>0.185(5)	$\mathrm{C_2}$**Si**H
cis-**2**	166.4(1)<br>170.6(1)<br>av. 168[2]	110.8(1)<br>111.7(1)<br>av. 111.4[4]	1.875(2)<br>1.891(2)<br>av. 1.881[5]	3.474(1)<br>3.499(1)<br>av. 3.48[14]	0.089(1)<br>0.090(3)	$\mathrm{C_2}$**Si**(H)Si
cis-**3**	175.5(1)	111.7(1)<br>111.9(1)	1.876(1)<br>1.883(1)<br>av. 1.880[3]	3.513(1)	0.090(2)<br>0.014(1)	$\mathrm{C_2}$**Si**(H)Si
**4**	140.0(1)	107.7(1)<br>107.1(1)	1.872(2)<br>1.881(2)<br>av. 1.878[3]	3.208(1)	0.054(3)<br>0.178(3)	$\mathrm{C_2}$**Si**(H)Si
**5**	176.2(1)	111.6(1)<br>111.1(1)	1.861(3)<br>1.893(3)<br>av. 1.875[1]	3.506(1)	0.080(4)<br>0.164(4)	$\mathrm{C_2}$**Si**(H)Si
**6**	153.1(1)	109.5(1)<br>110.9(1)	1.863(2)<br>1.874(2)<br>av. 1.869[5]	3.409(1)	0.022(3)<br>0.128(3)	$\mathrm{C_2}$**Si**(H)Si<br>$\mathrm{C_2}$**Si**$\mathrm{H_2}$
**7**	169.1(2)	111.5(2)<br>112.3(2)	1.852(4)<br>1.878(4)<br>av. 1.87[11]	3.451(2)	0.013(5)<br>0.165(5)	$\mathrm{C_2}$**Si**(H/Me)Si<br>$\mathrm{C_2}$**Si**$\mathrm{H_2}$
[**10**]$^{2-}$	136.8(3)/137.1(3)<br>122.7(5)/123.5(7)	103.3(3)<br>103.6(3)	1.787(14)<br>1.978(18)<br>av. 1.89[5]	3.138(2)<br>3.167(3)	0.01(2)<br>0.285(9)	$\mathrm{C_2}$**Si**(H)B
**13**	141.9(2)	107.6(1)<br>107.4(1)	1.868(2)<br>1.875(2)<br>av. 1.88[1]	3.191(1)	0.022(1)<br>0.225(2)	$\mathrm{C_3}$**Si**H
[**15**]$^{-}$	112.6(2)	100.9(2)<br>103.4(2)	1.866(5)<br>1.890(5)<br>av. 1.88[1]	2.971(2)	0.018(7)<br>0.169(7)	$\mathrm{C_3}$**Si**H
**VI**	179.7(3)	112.6(2)<br>111.4(2)	1.861(5)<br>1.864(5)<br>av. 1.863[2]	3.490(2)	0.027(6)<br>0.127(6)	$\mathrm{C_2}$**Si**$\mathrm{H_2}$<br>$\mathrm{C_3}$**Si**H
**VII**	119.2(1)<br>121.5(1)	103.4(1)<br>103.6(6)<br>av. 103.8[5]	1.872(1)<br>1.878(2)<br>av. 1.876[3]	2.982(1)	0.011(2)<br>0.014(1)	$\mathrm{C_3}$**Si**H

[a] Geometric parameters for [**10**]$^{2-}$ are derived from the crystal structure of $[\mathrm{K(18\text{-}c\text{-}6)(THF)_2}][\mathrm{K(18\text{-}c\text{-}6)}]_3$[**10**]$_2$. The compound $\mathrm{K_2}$[**10**] is omitted due to severe disorder, which precludes a reliable determination of the geometric parameters of the DSA fragment.

[b] Dihedral angle between adjacent aryl rings of the DSA fragment, as defined in Figure S120. The slash (/) separates the values calculated for each position of the disordered aryl ring.

[c] C–Si–C bond angle, with the carbon atoms belonging to different aryl rings of the DSA fragment, as defined in Figure S120.

[d] Si–C bond length within the DSA fragment, i.e., between the silicon atom and the aryl carbon atom of the DSA framework.

[e] Interatomic distance between the two silicon atoms of the DSA fragment.

[f] Perpendicular deviation of the silicon atom from the least-squares plane of the adjacent aryl ring.

[g] Substitution pattern at the silicon atoms of the DSA fragment. If different substitution patterns occur within the same molecule, the corresponding χ angles are listed in the same order. The slash (/) denotes different disordered fragments overlapping at the same crystallographic position.

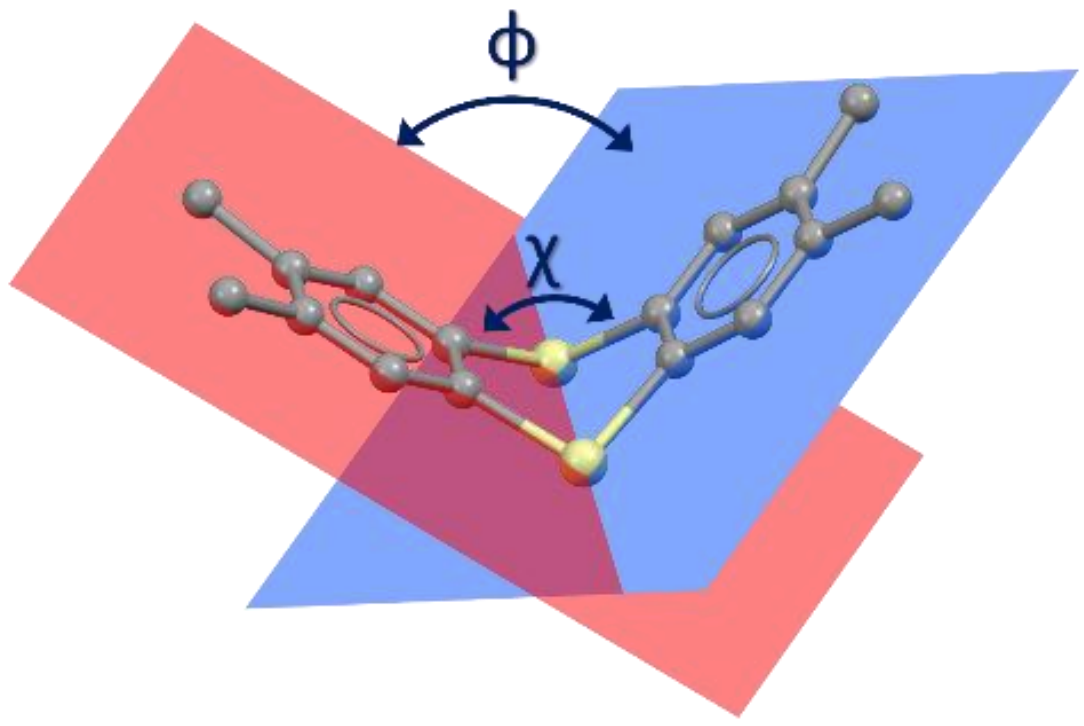

Figure S120. Definition of the C–Si–C bond angle χ at the silicon atoms in the DSA fragment and the dihedral angle ϕ between adjacent aryl rings. H atoms omitted for clarity. Si: yellow; C: grey.

4. References

[S1] M. Schmidt, N. H. H. Hürtgen, A. Virovets, H.-W. Lerner, M. Wagner, “Complete Series of 1,2-Bis(trihalogenosilyl)benzenes (F, Cl, Br, I): A Platform for Cooperating Lewis-Acidic Sites in Close Quarters” *Zeitschrift für Anorganische und Allgemeine Chemie* **652**, (2025): e202500154.

[S2] E. von Grotthuss, S. E. Prey, M. Bolte, H.-W. Lerner, M. Wagner, “Selective CO_2 Splitting by Doubly Reduced Aryl Boranes to Give CO and $[CO_3]^{2-}$” *Angewandte Chemie* **57**, (2018): 16491.

[S3] P. L. Lückert, J. Gilmer, A. Virovets, H.-W. Lerner, M. Wagner, “Donor-Free 9,10-Dihydro-9,10-Dialuminaanthracenes” *Chemical Science* **16**, (2025): 147.

[S4] B. Bogdanović, S.-T. Liao, R. Mynott, K. Schlichte, U. Westeppe, “Rate of Formation and Characterization of Magnesium Anthracene” *Chemische Berichte* **117**, (1984): 1378.

[S5] G. R. Fulmer, A. J. M. Miller, N. H. Sherden, H. E. Gottlieb, A. Nudelman, B. M. Stoltz, J. E. Bercaw, K. I. Goldberg, “NMR Chemical Shifts of Trace Impurities: Common Laboratory Solvents, Organics, and Gases in Deuterated Solvents Relevant to the Organometallic Chemist” *Organometallics* **29**, (2010): 2176.

[S6] E. A. Chernyshev, N. G. Komalenkova, L. N. Shamshin, S. A. Shchepinov, “Silicon-Containing Heterocyclic Compounds. IX. Preparation of Silicon-Containing Heterocycles by the Pyrolysis of Phenylchlorosilanes” *Zhurnal Obshchei Khimii* **41**, (1971): 843.

[S7] E. A. Chernyshev, N. G. Komalenkova, “The Thermal Gas-Phase Synthesis of Heterocyclic Compounds with One or Several Silicon Atoms in the Ring” *Russian Chemical Reviews* **58**, (1989): 559.

[S8] W. Z. McCarthy, J. Y. Corey, E. R. Corey, “Group 4 9,10-Dihydro-9,10-Diheteroanthracenes: Synthesis and Structure” *Organometallics* **3**, (1984): 255.

[S9] K. Thorshaug, O. Swang, I. M. Dahl, A. Olafsen, “An Experimental and Theoretical Study of Spin–Spin Coupling in Chlorosilanes” *The Journal of Physical Chemistry A* **110**, (2006): 9801.

[S10] P. Molinillo, A. Gálvez Del Postigo, M. Puyo, F. Vattier, A. M. Beltrán, N. Rendón, P. Lara, A. Suárez, “Selective H/D Exchange in E–H (E = Si, Ge, Sn) Bonds Catalyzed by 1,2,3-Triazolylidene-Stabilized Nickel Nanoparticles” *Inorganic Chemistry* **64**, (2025): 8125.

[S11] S. Bourg, B. Boury, F. H. Carré, R. J. P. Corriu, “Synthesis of New Silicon–Cobalt Clusters” *Organometallics* **17**, (1998): 167.

[S12] M. Itoh, K. Inoue, J. Ishikawa, K. Iwata, “Disproportionation Reactions of Organohydrosilanes in the Presence of Base Catalysts” *Journal of Organometallic Chemistry* **629**, (2001): 1.

[S13] M. A. Ring, D. M. Ritter, “Preparation and Reactions of Potassium Silyl” *Journal of the American Chemical Society* **83**, (1961): 802.

[S14] G. M. Sheldrick, “SHELXT – Integrated Space-Group and Crystal-Structure Determination” *Acta Crystallographica Section A: Foundations and Advances* **71**, (2015): 3.

[S15] G. M. Sheldrick, “A Short History of SHELX” *Acta Crystallographica Section A: Foundations of Crystallography* **64**, (2008): 112.

[S16] G. M. Sheldrick, “Crystal Structure Refinement with SHELXL” *Acta Crystallographica Section C: Structural Chemistry* **71**, (2015): 3.
